# Speciation atlas of polyoxometalates in aqueous solution (Part II): Molybdenum browns

**DOI:** 10.1126/sciadv.aea1910

**Published:** 2025-10-31

**Authors:** Ingrid Gregorovic, Nadiia I. Gumerova, Annette Rompel

**Affiliations:** ^1^ Universität Wien, Fakultät für Chemie, Institut für Biophysikalische Chemie, Josef-Holaubek-Platz 2, 1090 Wien, Austria.; ^2^ Vienna Doctoral School in Chemistry (DoSChem), Universität Wien, Währinger Straße 42, 1090 Vienna, Austria.

## Abstract

We present a validated, openly accessible workflow and spectral database charting the solution speciation of molybdenum browns. These redox-active polyoxometalates are central to catalysis and increasingly explored for bioinspired transformations and nanomedicine, making their solution behavior critical for both mechanistic understanding and practical use. Two archetypal Keplerate-type polyoxometalates, {Mo_72_V_30_} and {W_72_V_30_}, were investigated under 69 systematically varied aqueous conditions. Complementary ^51^V/^31^P nuclear magnetic resonance, resonance Raman, electrospray ionization mass spectrometry, and ultraviolet-visible spectroscopy interrogate the effects of pH, buffer identity, temperature, and incubation time on Keplerate integrity. Substituting Mo with W changes redox potential and hard/soft metal balance, yielding distinct stability patterns: {Mo_72_V_30_} breaks into fragments under all tested conditions whereas {W_72_V_30_} remains intact in fresh solutions and retains structural integrity up to pH 4 after 24 hours. All raw spectra, processed data, and stepwise protocols are deposited in a FAIR (findable, accessible, interoperable, reusable) repository, providing an open resource to accelerate catalytic and biological applications of polyoxometalates.

## Teaser

Metal-oxide nanocage stability depends on solution conditions, with an open dataset supporting future applications.

## INTRODUCTION

**Fig. 1.**
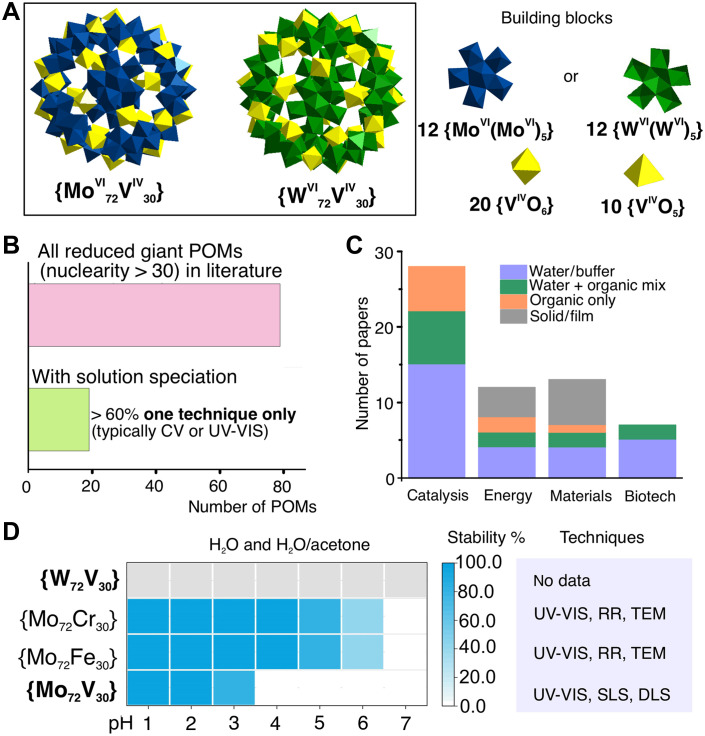
Literature overview of nanoscale POMs. (**A**) Structures of Keplerate-type POMs {Mo_72_V_30_} and {W_72_V_30_}. (**B**) Overview of numbers of all published giant POMs and their solution speciation. (**C**) Type of solvent media in applications of {Mo_72_V_30_} and {W_72_V_30_}. (**D**) Summary of stability studies of {M_72_M′_30_}–type Keplerates. Gray color corresponds to “no data.” The summarized data presented in (B) to (D) are described in detail in tables S3 and S4.

Polyoxometalates (POMs) are a class of metal-oxide inorganic compounds, mainly negatively charged, that exhibit a large range of structural diversity and a unique set of chemical and physical properties (*1*, *2*). Under highly acidic conditions (pH < 3) and in the presence of a reducing agent, molybdate salts self-assemble into partially reduced, nanoscale POMs known as molybdenum blues, extended wheel- or ball-shaped architectures composed of Mo(V)/Mo(VI) centers (*3*, *4*). A related family, often termed molybdenum browns (*5*), includes highly symmetrical Keplerate-type nanocages such as {Mo_132_} and {Mo_72_M′_30_} (where M′ = Fe^III^, V^IV^, or Cr^III^), which feature rigid, spherical frameworks with 132 or 72 Mo ions and carrying charges of up to −42 (Fig. 1A) (*6*–*9*). Despite being synthesized in solution, POMs’ structure characterization is primarily done in solid phase by x-ray diffraction techniques. More than 80% of their application, which include (photo)catalysis (*10*–*20*), medicine and biotechnology (*21*–*24*), host-guest chemistry (*25*–*27*), electrochemistry (*13*, *28*, *29*), and material science (*30*–*32*), take place in liquid media (Fig. 1). During dissolution in aqueous media, POM anions may undergo protonation (*33*), hydrolysis (*33*), and redox reactions (*34*), affecting the correct identification of active species. A review of the available literature shows that analytical studies (*35*, *36*) capable of determining which forms are present in aqueous media and when remain scarce for the largest, most reduced clusters (Fig. 1B). Of all nanosized POMs reported (table S3), only about 25% exhibit any data on their solution behavior, and > 60% of these reports rely on just a single analytical technique, typically ultraviolet-visible (UV-vis) spectroscopy or cyclic voltammetry (CV) (Fig. 1B and table S3). Reduced large POM structures such as Keplerates and molybdenum blues remain stable only within a narrow, highly acidic pH range, with molybdenum blues being present at pH levels as low as 1 to 1.5 (*37*). Keplerates are stable at slightly higher pH values than molybdenum blues, with their structural integrity observed up to about pH 4. Above this pH, the increased hydroxide concentration leads to deprotonation and destabilization of the Keplerate structure (*38*). The stability of Keplerates {Mo_72_M′_30_} (where M′ = Fe^III^, V^IV^, or Cr^III^) is typically assessed using UV-vis spectroscopy alone, while resonance Raman (RR), transmission electron microscopy (TEM), or light-scattering studies (SLS, DLS) have been used only in a few cases, primarily to investigate the formation of “blackberry” structures (Fig. 1D). Notably, there are no stability data or speciation studies available for {W_72_V_30_} (Fig. 1D and table S3) at all. The scarcity of solution-phase data, combined with the reliance on a narrow set of analytical techniques, leaves a critical gap between crystallographically defined Keplerate structures and their behavior under application-relevant conditions.

Building on our group’s previous work (*39*, *40*) on the speciation of the most common small POMs, including Keggin (*41*), Wells-Dawson (*42*), Anderson (*43*), Preyssler (*44*), and decavanadate-type structures (*45*), we now extend this open-access resource to encompass nanoscale, partially reduced POMs. In part I (*39*) of this study, we introduced a comprehensive “speciation atlas” for 10 widely used POMs across six structural archetypes and three addenda metal types, based on more than 1300 nuclear magnetic resonance (NMR) spectra collected under 54 aqueous conditions. This effort revealed key structure-stability relationships and provided a practical roadmap for selecting and applying POMs in catalysis, biology, and materials science, accompanied by a FAIR (findable, accessible, interoperable, and reusable) database to support future studies. The present work builds directly on that foundation by targeting the more structurally and electronically complex Keplerates and molybdenum blues. Understanding the solution speciation of Keplerates and molybdenum blues is key to unlocking their extraordinary redox, catalytic, and magnetic properties (tables S3 and S4), which stem from nanoscale hollow architectures, exceptionally high anionic charges [−20 to −42 per anion (*3*–*5*)] and dense arrays of paramagnetic mrtal centers (e.g., Mo^V^, V^IV^, and Fe^III^). In both Keplerates and molybdenum blues, structural complexity, strong paramagnetism, and chemical lability make it difficult to identify the true solution-phase species, hindering structure-function relationships and materials design. We have expanded our open speciation atlas, a FAIR, curated repository of stability maps and spectral fingerprints, to cover these complex, partially reduced clusters. With this addition, the atlas becomes a foundational resource for advanced catalytic, energy, and bioinspired applications.

The stability of Keplerate-type POMs is influenced by several factors, including pH, temperature, POM concentration, solvent type, and ionic strength (*38*). These variables notably affect their decomposition pathways and speciation in solution (*33*). When evaluating analytical methods for solution speciation of Keplerates, it is crucial to consider each technique’s advantages, limitations, and accessibility. NMR spectroscopy is a powerful tool, capable of detecting several NMR-active nuclei (^31^P, ^51^V, ^183^W, ^95^Mo, and, with enrichment, ^17^O) in a single instrument session (*33*, *46*, *47*). However, this approach requires a minimum solubility for reliable detection, and its effectiveness can be compromised by the presence of paramagnetic centers that broaden or quench resonances, obscuring weaker signals. In addition, there have been no systematic data on ^51^V NMR chemical shifts for polyoxovanadates, including isopolyvanadates (IPOVs) and mixed M–V small POMs (M = W^VI^ or Mo^V^). We compiled and present ^51^V NMR data of polyoxovanadates in figs. S5 to S7. RR spectroscopy, by contrast, is largely insensitive to paramagnetic interference and is highly valuable for characterizing both solution and solid-state samples of Keplerates (*48*). RR spectroscopy can detect vibrational modes associated with specific metal-oxygen bonds, such as Mo═O and Mo─O─Mo linkages, in theory enabling the identification of structural motifs, oxidation states, and the degree of reduction within the POM framework (*48*). Raman data exist for both small and nanoscale POMs across diverse, often incomparable experimental conditions (table S6).

Similarly, electrospray ionization mass spectrometry (ESI-MS) offers high sensitivity and can distinguish between species with similar mass/charge (*m/z*) ratios, although overlapping spectral envelopes can complicate interpretation (*33*, *49*–*51*). UV-vis spectroscopy, which is widely accessible and straightforward to use, excels at detecting reduced POMs through characteristic intervalence charge transfer (IVCT) absorption bands, such as Mo^V^ → Mo^VI^ transitions in {Mo_154_} near 750 nm (*33*, *49*), but UV-vis spectroscopy alone cannot verify whether the cage framework remains intact in solution. In summary, each analytical technique offers unique strengths and limitations; therefore, a combined analytical approach is necessary to achieve a comprehensive understanding of the solution speciation and structural features of Keplerates-type POMs.

For the present speciation study, we target the vanadium-containing Keplerate pair {Mo_72_V_30_} (*7*, *52*) and {W_72_V_30_} (*52*, *53*) (Fig. 1A). These ~3-nm nanocages share the same hollow framework motif as the archetypal molybdenum browns {Mo_132_} (*9*) and {Mo_72_Fe_30_} (*6*), and both compounds have been exploited in catalysis and energy-storage devices (table S4). Each cage hosts thirty V^IV^ centers (Fig. 1A) that, upon partial decondensation, can oxidize to V^V^ and thus become ^51^V-NMR–active, providing an intrinsic spectroscopic probe of solution behavior. A head-to-head comparison of the Mo and W variants therefore offers a controlled test of how simply swapping the addendum metal modulates speciation pathways and stability while size, charge, and heterometal content remain constant.

## RESULTS

### Experimental matrix and analytical toolbox

**Fig. 2.**
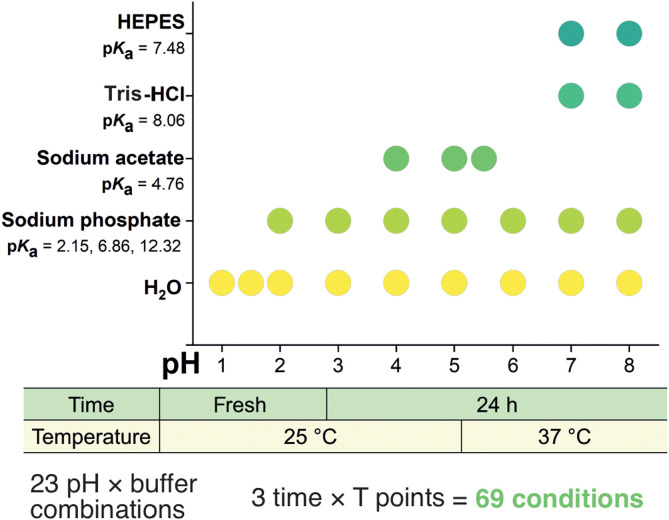
Experimental matrix for the Keplerate speciation study. The diagram summarizes the media tested: neat water and four 0.1 M buffers (sodium phosphate, acetate, tris-HCl, and Hepes) with their p*K*_a_ values at 25°C and operative pH ranges. The three sampling points analyzed: fresh (<10 min, T = 25°C), aged [24 hours (h), T = 25°C], and incubated (24 hours, T = 37°C).

We mapped the solution speciation of two vanadium-containing Keplerate POMs, {Mo_72_V_30_} (*7*) and {W_72_V_30_} (*53*) (Fig. 1A) across 69 distinct conditions—23 pH × buffer combinations, each examined at three time × temperature points (Fig. 2). POM solutions were prepared in water or in one of four widely used 0.1 M buffers (sodium phosphate, acetic acid–sodium acetate, tris-HCl, and Hepes), giving a pH window of 1 to 8 that spans the range for most catalytic and biological applications (table S4). The pH ranges included in this study can be divided into three regions: strongly acidic 1 ≤ pH ≤ 4, moderately acidic 5 ≤ pH ≤ 6, and neutral to moderately alkaline 7 ≤ pH ≤ 8. Each sample was characterized at three time × temperature points: fresh (< 60 min at 25°C), aged (24 hours at 25°C), and incubated (24 hours at 37°C). Speciation was probed by four complementary techniques: ^51^V NMR (^31^P NMR in sodium phosphate buffer), RR spectroscopy, ESI-MS, and UV-vis spectroscopy providing orthogonal views of both intact cages and decomposition products. A key challenge in monitoring vanadium speciation is the paramagnetic nature of all 30 V^IV^ centers in both Keplerates (Fig. 1A), which leads to broad or absent ^51^V NMR signals. The absence of a ^51^V NMR signal of NMR spectra of {W_72_V_30_} (fig. S45) indicates complete reduction and structural integrity, while broadened signals in ^51^V NMR of both Keplerates (figs. S9 and S48) suggest partial oxidation to V^V^ and interference from residual paramagnetic V^IV^ (*54*, *55*). We define a Keplerate as stable if its structural integrity is confirmed by all four techniques: NMR, RR, ESI-MS, and UV-vis. If ^51^V NMR, the most sensitive method, or RR spectra reveal signs of decomposition alongside evidence of an intact Keplerate, we classify the condition as partially stable. If only UV-vis preserves the IVCT band while all other methods indicate decomposition, the Keplerate is considered unstable, as the band merely reflects the presence of reduced centers rather than the intact structure. 

### Global trends in Keplerate stability and speciation

Before examining each pH × buffer × time × temperature combination in detail, we first outline the overarching speciation patterns that emerge across the full 75-condition matrix for both Keplerates.

#### pH window

**Fig. 3.**
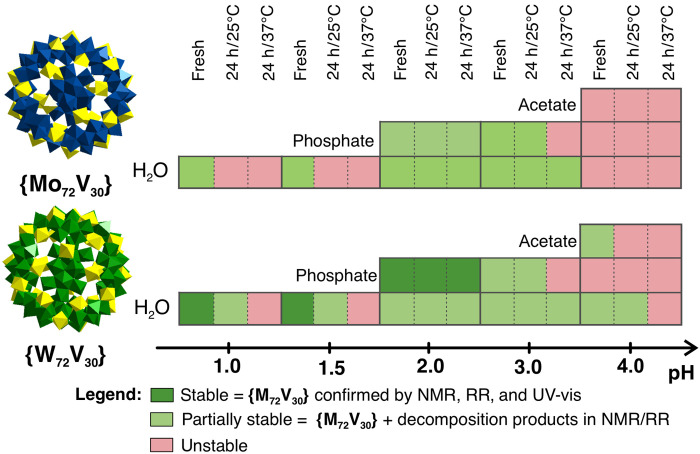
Stability map of Keplerate-type POMs: {Mo_72_V_30_} and {W_72_V_30_}. The stability map shows the presence of Keplerates in three different solution conditions: fresh, 24-hour aged at room temperature, and 24-hour incubated at 37°C. The stability conclusions were made by comparing the data collected by ^51^V NMR, RR, and UV-vis spectroscopy (tables S16 and S28). In the case of “full stability”—^51^V NMR showed no signal (presence of V^IV^), while RR spectroscopy confirmed the exclusive presence of the intact Keplerate, and UV-vis showed a stable IVCT absorption band. The figure shows only solutions with pH ≤ 4 as we detected no intact Keplerates in solutions at pH > 5. All buffers were prepared in concentration of 0.1 M. h, hours.

Both Keplerates survive only in a strongly acidic window (pH ≤ 4) (Fig. 3). Intact cages are detected by the absence of a ^51^V NMR signal (all V^IV^), a characteristic IVCT band, and a single RR fingerprint, and corroborating ESI-MS peaks for the intact anion. Above pH 4, ^51^V NMR peaks emerge, and a cascade of iso- and heteropoly-vanadate signals appears, indicating rapid decondensation. Above pH 5, the four-technique consensus required to confirm that an intact cage is lost: UV-vis, RR, or ESI-MS may still show cage-like cues, but ^51^V NMR reveals small vanadate signals, so we can no longer assign an intact Keplerate with confidence under any buffer or temperature chosen. The initial pH just in distilled water is ~3.8 for {Mo_72_V_30_} and ~3.6 for {W_72_V_30_}, with no measurable change over 72 hours without external pH adjustment.

#### Buffer effect

Below pH 4, the stability of {Mo_72_V_30_} depends only on proton activity: Phosphate and simple HCl/H_2_O give the same speciation, whereas {W_72_V_30_} is best preserved in phosphate around pH 2. In the moderately acidic to neutral zone (pH 5 to 8) the IVCT band endures only in neat water for both cages. Above pH 5, all investigated buffers accelerate breakdown and channels the addenda metals into buffer-specific Mo–V or W–V products.

#### Temperature effect

**Fig. 4.**
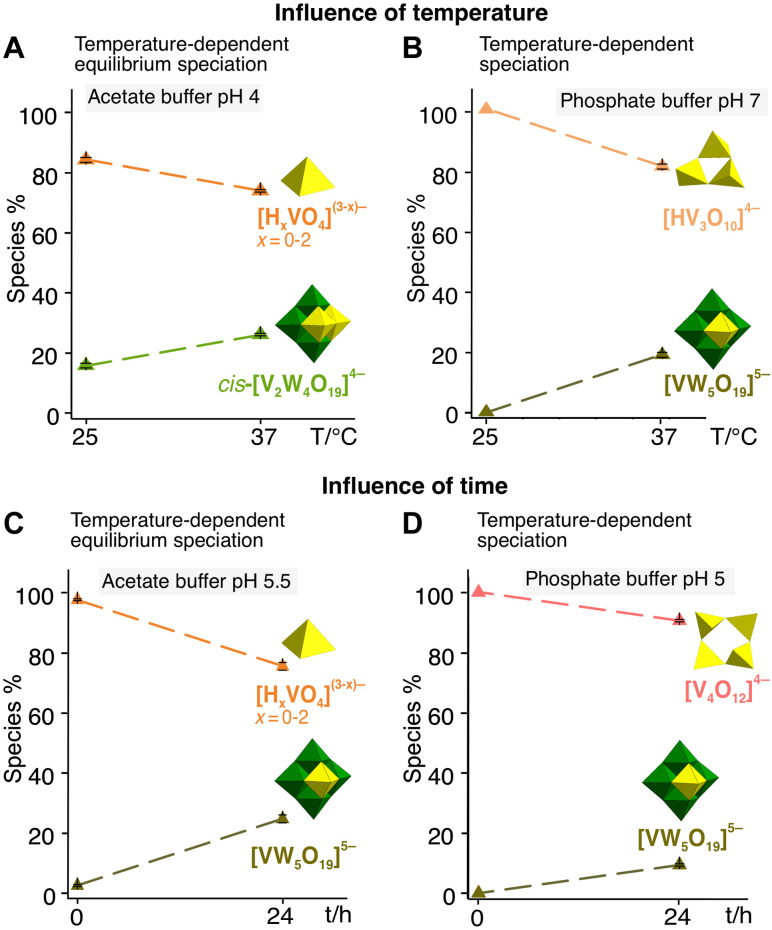
Influence of temperature and time on speciation of Keplerates in aqueous solutions. (**A**) Temperature-dependent equilibrium speciation: 0.1 M acetic acid–sodium acetate buffer solution of {W_72_V_30_} with pH 4 (**B**) temperature-dependent speciation: 0.1 M sodium phosphate buffer solution of {W_72_V_30_} with pH 7, (**C**) time-dependent equilibrium speciation: 0.1 M acetic acid–sodium acetate buffer solution of {W_72_V_30_} with pH 5.5, and (**D**) time-dependent speciation: 0.1 M sodium phosphate buffer solution of {W_72_V_30_} with pH 5.

Raising the solution temperature from ambient (~25°C) to physiological temperature (37°C) leaves both Keplerates ({Mo_72_V_30_} and {W_72_V_30_}) unchanged when the cage is still robust (pH ≤ 4), but it alters speciation once decomposition has already begun. Two behaviors emerge (Fig. 4, A and B): (i) Temperature-dependent equilibrium: When oxidation and hydrolysis products have already reached a steady distribution after 24 hours at 25°C, an additional 24 hours at 37°C does not create additional species but shifts their relative abundances. In this case ^51^V NMR shows Mo–V or W–V heteropoly anions growing at the expense of low-nuclearity vanadates. (ii) Temperature-dependent speciation: If the room-temperature sample is still evolving, heating opens additional breakdown pathways, so species appear or disappear and overall diversity increases. Thus, 37°C either biases an existing equilibrium or, under more labile conditions, accelerates for further decomposition.

#### Time effect

Prolonged incubation at 25°C produces the same two behavioral regimes seen for temperature (Fig. 4, C and D). (i) Time-dependent equilibrium: If the initial species are already stable within the first hour, aging for 24 hours does not generate additional POM types. Instead, ^51^V-NMR simply records a redistribution toward higher-nuclearity products, such as mixed Mo–V or W–V heteropolyanions and larger isopolyvanadates (IPOVs) such as decavanadate [V^V^_10_O_28_]^6−^ (V_10_, fig. S2). (ii) Time-dependent speciation: If the fresh solution is still evolving, 24-hour aging at room temperature unlocks additional hydrolysis and polymerization pathways, so smaller POMs appear and overall structural diversity rises. Thus, like heating, time either tilts an existing equilibrium toward larger clusters or, when the cage is fragile, drives further decomposition.

### Strongly acidic aqueous solutions (1 ≤ pH ≤ 4)

Each pH window opens with a one-sentence “key finding” and then follows a uniform three-part sequence: (i) speciation of {Mo_72_V_30_}, (ii) speciation of {W_72_V_30_} under identical conditions, and (iii) a concise side-by-side comparison that highlights the addenda-metal effect and points to the relevant supplementary figures and tables. We apply this template here for the strongly acidic regime (1 ≤ pH ≤ 4) and reuse it unchanged in the moderately acidic and neutral/alkaline sections, allowing readers to navigate and cross-reference trends quickly. Although broader pH ranges (e.g., pH 1 to 4) are used for narrative clarity, each pH point was analyzed and interpreted individually. Full-resolution data are provided in the Supplementary Materials.

#### Key finding

The Mo-based Keplerate persists only below pH 3 and is absent by pH 4 (and after warming up to 37°C even at pH 3), whereas the W-based analog remains the dominant species throughout pH 1 to 3 and still coexists with smaller POM fragments at pH 4.

#### {Mo_72_V_30_}

**Fig. 5.**
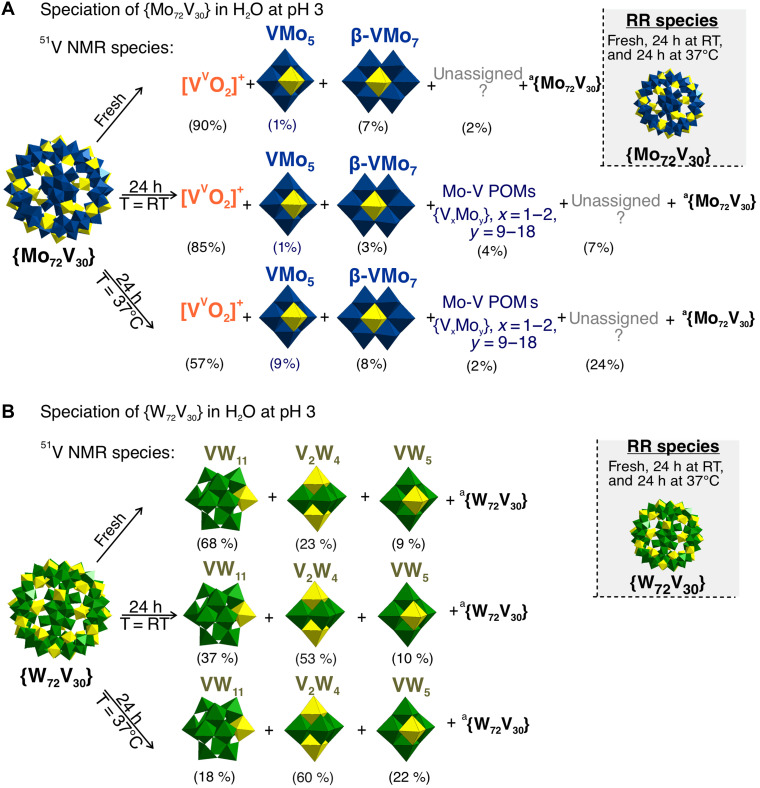
Speciation scheme of Keplerates in aqueous solutions at pH 3. (**A**) {Mo_72_V_30_} in H_2_O with pH 3 and (**B**) {W_72_V_30_} in H_2_O at pH 3 in fresh solutions, after 24-hour at 25°C, and after 24-hour incubation at 37°C based on ^51^V NMR, RR, and ESI-MS data. Only mixed M−V (M = Mo^VI^, W^VI^) POM species with > 5% are shown separately. Similar decomposition schemes for all Keplerates solutions with pH 1 to 4 are included in the Supplementary Materials (figs. S19 to S23, S26 to S28, S32, S55 to S59, S64 to S66, and S71). ^a^Broad signals in ^51^V NMR indicate the presence of paramagnetic V^IV^, suggesting the presence of the intact {M_72_V_30_}. h, hours.

*Acidified H_2_O (pH 1 to 4).* ^51^V NMR, RR, and UV-vis confirm that the intact cage {Mo_72_V_30_} is present in HCl-acidified H_2_O at pH 1 to 3 (Fig. 3, figs. S8, S9, and S38, and tables S8 to S10 and S15). The IVCT band at 510 nm, however, fades within 1 hour at pH ≤ 2, signaling rapid oxidation (*49*, *51*), and ESI-MS fails to detect the intact anion below pH 2, most likely because the high concentration of [H^+^] and [Cl^−^] suppress large ions (*56*). These strongly acidic solutions host the richest set of breakdown products: Lindqvist [V^V^Mo^VI^_5_O_19_]^3−^ (VMo_5_) dominates at pH 2, while simple V(V) species {[V^V^O_2_]^+^, [H_x_V^V^O_4_]^x-3^ (V_1_)} rise toward pH 3 and 4 (Fig. 5A, fig. S23, and tables S11). After 24 hours at 25°C or 37°C, RR (table S15) reveals Mo-based fragments—[Mo^VI^_3_O_10_]^2−^ (Mo_3_) and α-[Mo^VI^_8_O_26_]^4−^ (α-Mo_8_), and alongside V_1_–V_5_ {[H_x_V^V^O_4_]^x-3^ (V_1_), [V^V^_2_O_7_]^4−^ (V_2_), [V^V^_3_O_10_]^5−^ (V_3_), [V^V^_4_O_12_]^4−^ (V_4_), [V^V^_5_O_15_]^5−^ (V_5_)} vanadates, these Mo species remain undetectable by ^51^V NMR. All remaining peaks marked ‘?’ (Fig. 5A) account for ≤ 24% (usually ≤ 5%) of the integrated ^51^V signal and will be targeted in follow-up work using ^17^O-labeled two-dimensional heteronuclear multiple-quantum coherence, high-field cryo-NMR, and standard-addition controls. Updated spectra will be deposited in the FAIR PHAIDRA database as they become available.

*Phosphate (0.1 M) (pH 2 to 4).* ^51^V NMR and RR spectroscopy show that the Keplerate remains intact in this buffer at pH 2 to 3; however, it is already partially decomposed at pH 4 even in fresh or 24-hour aged at room temperature solutions, where [Mo^VI^_7_O_24_]^6−^ (Mo_7_) and V_1_–V_5_ vanadates are observed (figs. S12, S13, S26, S27, and S28 and tables S12 and S15). The IVCT band remains unchanged for 24 hours at 25°C across the pH 2 to 4 range, yet it fades when the pH 2 sample is incubated at 37°C (fig. S40). Occurring vanadium speciation is simple: orthovanadate V_1_ or H_2_V_2_ dominate, and ^51^V NMR detects no species beyond V_1_ and V_2_ in any sodium phosphate sample at pH from 2 to 4 (tables S8, S9, and S12). Heating the pH 2 solution brings in a modest amount (~13%) of [V^V^Mo^VI^_7_O_26_]^5−^ (β-VMo_7_) (tables S10 and S12). ^31^P NMR adds one extra clue: The [P_2_Mo^VI^_5_O_23_]^6−^ (P_2_Mo_5_) anion [1.24 parts per million (ppm)] is seen at pH 2 and in aged/incubated pH 3 samples but is absent at pH 4, where only the phosphate buffer resonance is observed.

*Acetate (0.1 M) (pH 4).* In acetate buffer, RR still shows {Mo_72_V_30_} signature bands in the 24 hour–aged sample, together with a single unassigned line at 695 cm^−1^ (table S15). The accompanying small-POM pattern resembles that in neat water and now includes two buffer-specific Mo–V clusters, α-[V^V^_2_Mo^VI^_18_O_62_]^6−^ (α-V_2_Mo_18_) and [HV^V^_2_Mo^VI^_8_O_32_]^5−^ (HV_2_Mo_8_) (tables S11 and S13). ^51^V NMR confirms orthovanadate V_1_ as the major vanadium species in both fresh and aged solutions, while decavanadate V_10_ rises slightly from ≈30% in the freshly prepared sample to ≈34% after 24 hours of incubation at 37°C, exactly offsetting the decline of V_1_ (table S13 and fig. S32). RR corroborates V_10_ formation. The Keplerate’s IVCT band at 510 nm remains unchanged (fig. S42A), indicating that the parent {Mo_72_V_30_} cage survives in acetate at pH 4.

#### {W_72_V_30_}

*Acidified H_2_O (pH 1 to 4).* ^51^V NMR is completely silent at pH 1 to 1.5, even after 24 hours at 37°C, confirming that all V centers remain paramagnetic V^IV^ and the {W_72_V_30_} cage is fully intact (tables S20 to S22 and fig. S45). RR spectroscopy, ESI-MS studies, and a steady 390-nm IVCT band seen in UV-vis spectra corroborate this stability (tables S27, S29, and S30, and figs. S77 and S83). From pH 2 upward, a single broad ^51^V peak appears (figs. S45 to S46), but all orthogonal probes still report an undamaged cage. Only trace breakdown products form: Lindqvist [V^V^W^VI^_5_O_19_]^3−^ (VW_5_) dominates the most acidic samples and is often the sole species detected in fresh pH 2 or aged/-heated to 37°C pH 1 to 2 solutions (figs. S55 to S57). Above pH 3, *cis*-*trans*-[V^V^_2_W^VI^_4_O_19_]^4−^ (V_2_W_4_) begin to appear (Fig. 5B and figs. S58 and S59). IPOVs are scarce (< 10% of total V) and are detected only under the most aggressive conditions (e.g., H_x_V_2_ in incubated pH 1 samples). By pH 4, simple orthovanadate V_1_ becomes the predominant vanadium species (fig. S59).

*Phosphate (0.1 M) (pH 2 to 4).* Phosphate does not destabilize the W-based Keplerate in the strongly acidic range: pH 2 samples remain cage-silent in ^51^V NMR even after 24 hours at 37°C (fig. S48), and UV-vis still shows the full 390-nm IVCT band (fig. S79B, tables S20 to S22, and fig. S64). Starting at pH 3, a single broad ^51^V signal emerges, indicating that while the paramagnetic cage is still present, partial dissociation into small vanadates has begun. V_1_–V_5_ vanadates account for the rest of the vanadium signal, and a minor VW_5_ component is seen only in the aged pH 4 sample (tables S20 to S22 and S24 and figs. S65 and S66). ^31^P NMR spectroscopic investigation detects no P-containing heteropolyanions at pH 2 to 4. Raman spectra for pH 2 to 4 buffers consistently show the cage together with [HW^VI^_6_O_21_]^5−^ (HW_6_), [W^VI^_7_O_24_]^6−^ (W_7_) and V_1_–V_5_ vanadates (table S27), while the IVCT band remains unchanged (figs. S79, C and D). Overall, phosphate sustains an intact W cage up to pH 3 but allows gradual exchange with simple vanadates as the medium approaches pH 4.

*Acetate (0.1 M) (pH 4).* V_1_ orthovanadate is identified as the predominant (between 74 and 100%) species under all three solution conditions—fresh, 24-hour aged at 25°C, or incubated at 37°C (table S25). In fresh pH 4 solutions, only V_1_ is detected by ^51^V NMR (table S20 and figs. S51 and S71). W-V mixed POMs (*cis*-V_2_W_4_) are present only in aged and incubated solutions (tables S21, S22, and S25 and fig. S71). RR analysis (table S27) detects the Keplerate signature in all samples except the incubated solutions, where HW_6_, W_7_, and vanadates are observed instead. UV-vis (fig. S81A) confirms complete IVCT band stability at pH 4.

#### Mo versus W—Bottom line

In HCl solutions, {Mo_72_V_30_} is detectable as an intact cage only up to pH 3 and already begins to oxidize below pH 2, giving a mixture of Lindqvist VMo_5_ and simple vanadates. Phosphate or acetate buffers mitigate but do not prevent this breakdown. By contrast, {W_72_V_30_} shows no ^51^V NMR signal (all V^IV^, intact cage) down to pH 2, retains a stable 390-nm IVCT band in every medium tested, and yields only VW_5_ or V_2_W_4_ fragments (Fig. 5).

### Moderately acidic aqueous solutions (pH 5-6)

#### Key finding

In this “transition zone” the Keplerate framework is no longer dominant: Vanadate ions take over, and time or mild heating shifts a small but growing share of vanadium into mixed-metal small POMs for both Mo and W systems.

#### {Mo_72_V_30_}

*Acidified H_2_O (pH 5 to 6).* At pH 5, RR, UV-vis, and ESI-MS still display fingerprints consistent with the reduced Keplerate, yet ^51^V NMR spectra (fig. S10 and tables S8 and S9) are dominated by free orthovanadate V_1_. This conflicting evidence prevents us from confirming that a notable fraction of intact cages remains. Trace mixed Mo–V anions and the first signs of decavanadate V_10_ also appear in the fresh spectrum (tables S9 to S11). After 24 hours at 25°C, the V_1_ signal declines while V_10_ grows, and an additional 24 hours at 37°C amplifies this shift and introduces Lindqvist VMo_5_ (tables S9 to S11). At pH 6, the vanadium pattern simplifies to V_1_ and V_3_, essentially unchanged by aging or heat, although an extra 695 cm^−1^ Raman band hints at partial cage loss (table S15).

*Phosphate (0.1 M) (pH 5 to 6).* The Keplerate undergoes substantial dissociation in this buffer. ^51^V NMR spectra are dominated by small vanadate oligomers (V_2_–V_4_), and only after 24 hours at 37°C at pH 6 mixed Mo–V species appear, contributing less than 10% of the total vanadium signal (figs. S13 and S14 and tables S8 to S10 and S12). RR data (table S15) still detect Keplerate bands in fresh and aged pH 5 samples, but at pH 6 or after heating, the spectra shift to molybdate fragments (β-Mo_8_, Mo_7_) and simple vanadates (V_1_–V_2_). The 510-nm IVCT band also vanishes upon heating, confirming oxidation of the reduced cage (fig. S41, A and B).

**Fig. 6.**
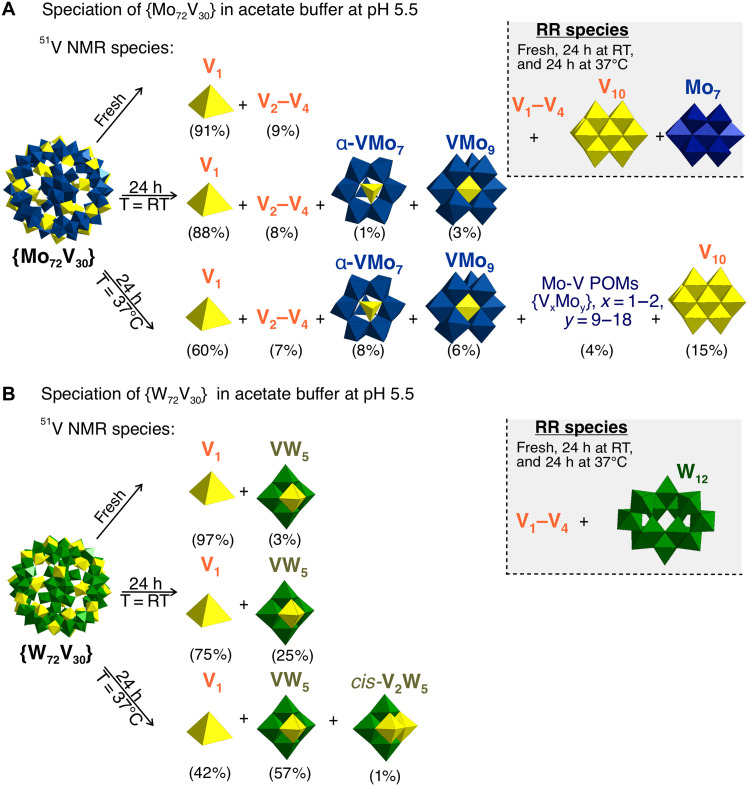
Speciation scheme of Keplerates in acetic acid–sodium acetate buffer at pH 5.5. (**A**) {Mo_72_V_30_} in 0.1 M acetate buffer at pH 5.5 and (**B**) {W_72_V_30_} in 0.1 M acetate buffer at pH 5.5 in fresh solutions, after 24-hour aging at 25°C and after 24 hour incubation at 37°C based on ^51^V NMR, and RR data. Only mixed M−V (M = Mo^VI^, W^VI^) POM species with >5% are shown separately. Decomposition schemes for all Keplerates solutions between pH 5 and 6 are included in the Supplementary Materials (figs. S24, S25, S28, S29, S33, S34, S60, S61, S67, S68, S72, and S73). h, hours.

*Acetate (0.1 M) (pH 5 to 5.5).* Orthovanadate V_1_ is still the major vanadium species at pH 5 (> 50% by ^51^V NMR), but 24 hours of aging or heating steadily converts part of it into decavanadate V_10_ and a small set of mixed Mo–V clusters (≤5%) (figs. S15 and S33 and tables S8 to S10 and S13). α-V_2_Mo_18_ appears in fresh and aged samples, while [HV^V^Mo^VI^_9_O_32_]^4−^ (HVMo_9_) is seen only after aging solutions (tables S8 to S10 and S13 and fig. S33). At pH 5.5, the fresh solution shows only V_1_–V_4_ vanadates. After 24 hours at 25°C, mixed Mo–V clusters rise to ~5%, and a 24-hour incubation at 37°C lifts them to ~18% and pushes V_10_ to ~16%, at the expense of V_1_ (Fig. 6A). At both pH values, RR (table S15) and UV-vis track the same trend: Raman shows bands for V_1_–V_5_ and Mo–V species, and the 510-nm IVCT band remains largely intact, dipping only slightly after the 37°C run (fig. S42, B and C).

#### {W_72_V_30_}

*Acidified H_2_O (pH 5-6).* At pH 5, ^51^V NMR is dominated by orthovanadate V_1_ and V_3_ (> 95% of total V) with only traces of mixed W–V POMs present (tables S20 to S23 and figs. S46 and S60). After 24 hours at 25°C, both V_3_ and the W–V fraction rise (V_3_ ≈ 10%), while V_1_ decreases. A further 24 hours at 37°C reduces the combined V_1_ + V_3_ fraction to below 70% and triples the mixed-W–V pool to more than 30%, yielding *cis*- and *trans*-V_2_W_4_. Raman and UV-vis spectroscopic investigations still show the Keplerate fingerprint and a stable 390-nm IVCT band under all tested conditions (table S27 and fig. S60). At pH 6, the fresh solution contains mostly V_1_ and HV_3_. The 24-hour aging removes ~20% of the vanadate signal in favor of W–V Lindqvist-type POMs (fig. S61 and tables S20-S23). RR measurements (table S27) detect {W_72_V_30_} together with V_1_–V_5_, W_7_, in fresh and aged samples and only {W_72_V_30_} after heating, while ESI-MS confirms intact Keplerate ions in all fresh pH 5 and pH 6 solutions (table S30).

*Phosphate (0.1 M) (pH 5-6).* In this buffer, the ^51^V NMR shows that tetravanadate V_4_ dominates at both pH values (fig. S49, S50). Upon aging, a trace amount of Lindqvist-type VW_5_ emerges, which increases to ~28% at pH 5 and only 9% at pH 6 (tables S21, S22, and S24 and fig. S67, S68). RR spectroscopy detects the Keplerate bands together with HW_6_ and V_1_–V_5_ (table S27), and the 390-nm IVCT band remains essentially intact, decreasing only slightly after 24 hours (fig. S80, A and B).

*Acetate (0.1 M) (pH 5-5.5).* In fresh buffer at pH 5, V_1_ orthovanadate accounts for > 95% of the vanadium signal and only traces of Lindqvist VW_5_ and V_2_W_4_ are present (tables S20 to S22 and S25 and figs. S51 and S72). After 24 hours (25°C), V_1_ falls to ~61% and the Lindqvist cluster fraction rises, a trend that strengthens on heating to 37°C, when VW_5_ becomes the major product. The Raman spectroscopic investigation consistently detects V_1_–V_5_ vanadates plus a paratungstate marker band [H_2_W^VI^_12_O_42_]^10−^ (H_2_W_12_) (table S27), and UV-vis shows an unchanged 390-nm IVCT band, indicating that the reduced W cage itself remains (fig. S81B). The behavior at pH 5.5 mirrors pH 5: Fresh and aged samples contain chiefly V_1_ with a minority of VW_5_, while 37°C incubation shifts the equilibrium almost entirely to Lindqvist species (Fig. 6B).

#### Mo versus W—Bottom line

The Mo cage essentially disappears, leaving V_1_/V_3_/V_4_ vanadates plus low-nuclearity Mo–V complexes. In contrast, the W cage remains detectable by RR and UV-vis spectroscopy and converts only partially, more slowly, and mainly to Lindqvist VW_5_/V_2_W_4_, making {W_72_V_30_} markedly more resilient under moderately acidic conditions.

### Neutral to moderately alkaline aqueous solutions (pH 7-8)

#### Key finding

In neutral water, both cages persist only as a minor reduced fraction that coexists with free vanadates, but in every buffer, the Keplerate signature collapses and speciation is dominated by buffer-dependent vanadate and mixed-metal fragments.

#### {Mo_72_V_30_}

*Neutralized H_2_O (pH 7-8).* At both pH values, ^51^V NMR shows a mixture of V_1_–V_4_ IPOVs (tables S8 to S11 and figs. S11 and S25). RR spectroscopy still picks up the Keplerate signature in every sample, although extra bands at 692 cm^−1^ (aged pH 7) and 689 cm^−1^ (fresh/aged pH 8) hint at additional, as-yet-unassigned fragments (table S15). ESI-MS detects the same vanadate series in fresh solutions (table S18), and the 510-nm IVCT band remains unchanged across pH 7 to 8, indicating that the reduced species persists alongside the free V_1_–V_4_ (figs. S39C, D).

*Phosphate (0.1 M)* (*pH 7-8).* In this buffer, the cage is fully decomposed. At pH 7, ^51^V NMR shows only tetravanadate V_4_, with V_5_ appearing after 37°C incubation (tables S8 to S10 and S12 and figs. S13 and S30). Raman spectroscopy confirms the absence of the cage and records Mo_7_ + V_1_ in the fresh sample, shifting to Mo_7_/[Mo^VI^_6_O_19_]^2−^ (Mo_6_) + V_4_ after aging and [Mo^VI^O_4_]^2−^ (Mo_1_) + V_1_ after incubation (table S15). The Keplerate IVCT band vanishes completely at 37°C (fig. S41C). At pH 8, a mixed [V^V^Mo^VI^_4_O_17_]^5−^ (VMo_4_) anion appears in fresh and aged samples, but aging/heating again reduces the spectrum to V_4_/V_5_ only (tables S8 to S10 and S14 and fig. S31). Raman investigation tracks the same trend, with Mo fragments progressing from Mo_7_ (fresh) to Mo_1_ (aged) and Mo_6_ (incubated) (table S15). The IVCT band survives just a few minutes (~4 min) in the fresh sample before fading completely (fig. S41D).

**Fig. 7.**
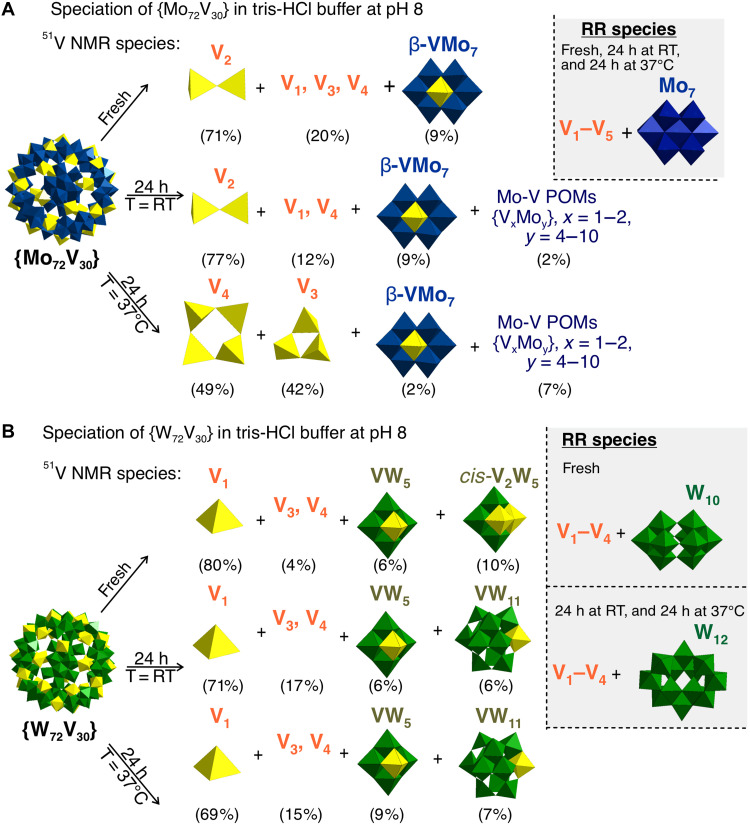
Speciation scheme of Keplerates in tris-HCl buffer at pH 8. (**A**) {Mo_72_V_30_} in tris-HCl at pH 8 and (**B**) {W_72_V_30_} in tris-HCl at pH 8 in fresh solutions, after 24 hours at 25°C after 24-hour incubation at 37°C based on ^51^V NMR and RR data. Only mixed M−V (M = Mo^VI^, W^VI^) POM species with > 5% are shown separately. Similar decomposition schemes for all Keplerates solutions with pH 7 to 8 are included in the Supplementary Materials (figs. S25, S30, S31, S35 to S37, S62, S63, S69, S70, and S74 to S76). h, hours.

*Tris-HCl (0.1 M) (pH 7-8).* At pH 7, ^51^V NMR is still dominated by simple vanadates V_1_–V_4_ (≥80%) (fig. S16). Over 24 hours at either temperatures, up to ~15% of the vanadium shifts into mixed Mo–V clusters, including β-VMo_7_, α-V_2_Mo_18_, HVMo_9_, and [V^V^_9_Mo^VI^O_28_]^5−^ (V_9_Mo) (tables S9, S10, and S14 and fig. S35). Raman data reflect the same change, showing Mo_7_ fragments together with an additional 778-cm^−1^ band (likely V_10_) (table S15). The 510-nm IVCT band completely fades after 22 hours at 37°C (fig. S43A). At pH 8, the fresh solution again contains > 85% V_1_–V_5_ vanadates, with β-VMo_7_ and [HV^V^2Mo^VI^_10_O_38_]^5−^ (HMo_10_V_2_) making up the balance (Fig. 7A, tables S8 to S10 and S14, and figs. S16 and S36). Aging removes most V_1_, enriches H_x_V_2_, and generates HMo_10_V_2_ and β-[H_2_V^V^_2_Mo^VI^_6_O_26_]^4−^ (β-H_2_V_2_Mo_6_), while incubation adds a few more IPOVs and Mo–V clusters (tables S9 and S14 and fig. S36). The IVCT band is visible only in freshly prepared samples (fig. S43B), and Raman spectra evolve from Mo_7_ + vanadates (fresh) to Mo_1_/Mo_7_ + vanadates after aging and heating (table S15).

*Hepes (0.1 M) (pH 7-8).* At pH 7, ^51^V NMR reveals only small vanadates V_1_–V_4_, with V_3_ as the dominant species (>60%), and POVs relative proportions shift slightly upon aging or 37°C incubation (tables S8 to S10 and S14 and figs. S17 and S37), in agreement with RR data (table S15). The IVCT band drops only slightly after 24 hours at 37°C (fig. S43C). At pH 8, the speciation remains similar: vanadates V_1_–V_5_ dominate (H_x_V_2_ fresh/aged, V_4_ after heating). The Raman data follow the same trend, shifting from Mo_7_ + vanadates in aged samples to Mo_1_ + vanadates after heating (table S15). The IVCT band is visible only in very fresh solution (fig. S43D).

#### {W_72_V_30_}

*Neutralized H_2_O (pH 7-8).* At pH 7, ^51^V NMR spectrum is almost entirely orthovanadate V_1_ (≈ 90%) with a little V_3_. Lindqvist VW_5_ is barely detectable in fresh or aged samples but rises to ~10% after 24 hours at 37°C (fig. S47, tables S20 to S23, and fig. S62). RR and UV-vis spectroscopy still display a Keplerate signature, and ESI-MS confirms intact cage ions in fresh solutions (table S27 and fig. S78C). At pH 8, V_1_–V_3_ again dominate and VW_5_ remains below 3% (fig. S47 and tables S20 to S23). Raman spectra now show the initial Keplerate, W_7_ POM, and V_1_–V_5_ IPOVs (table S27). The 390-nm IVCT band remains stable after 24 hours at 37°C (fig. S78D), indicating that some fraction of a reduced W-V cage persists alongside the free vanadates.

*Phosphate (0.1 M) (pH 7-8).* At pH 7, ^51^V NMR is restricted to HV_3_, with Lindqvist VW_5_ appearing only after 24 hours at 37°C (figs. S50 and S69 and tables S20 to S22 and S24). Raman data and UV-vis still show a fingerprint of W-based Keplerate in the fresh and aged samples, but incubation replaces the cage bands with tungstate fragments (HW_6_) plus V_1_–V_5_ vanadates (table S27 and fig. S80C). At pH 8, a single H_x_V_2_ signal dominates the fresh solution, while aging and heating introduce additional species—HV_3_ and V_4_ (tables S20 to S22 and S24 and figs. S50 and S70). The corresponding RR spectra evolve from W_7_ (fresh) to W_7_ + HW_6_ (aged) and finally H_2_W_12_ (incubated), always with the same V_1_–V_5_ vanadate series (table S27). The IVCT band at 390 nm fades gradually during aging and disappears after 24 hours at 37°C, indicating slow oxidation of metal centers (fig. S80D).

*Tris-HCl (0.1 M) (pH 7-8).* At pH 7, the vanadium pool consists almost entirely of V_1_–HV_3_, and only after 24 hours at 37°C does a small VW_5_ fraction (~5%) appear (fig. S52, tables S20 to S22 and S26, and fig. S74). RR investigations show HW_6_/W_7_ fragments in every sample and [W^VI^_10_O_32_]^4−^ (W_10_) only after aging (table S27), while the 390-nm IVCT band stays nearly constant (fig. S82A). At pH 8, speciation broadens: Fresh solutions contain ≈85% V_1_ + H_x_V_2_ and ≈15% W-V clusters (VW_5_, *cis*-H_2_V_2_W_4_) (Fig. 7B, fig. S75, and tables S20 and S26). Aging or heating increases the proportion of vanadates (H_x_V_1_, HV_3_, V_4_), slightly raises the W-V share (VW_5_, [H_2_V^V^W^VI^_11_O_40_]^7−^ (H_2_VW_11_) < 15%), and introduces a minor unknown at −533 ppm (fig. S75, and tables S21, S22 and S26). Raman spectroscopic investigations confirm the growing tungstate family {W_12_, [W^VI^O_4_]^2−^ (W_1_) and [W^VI^_2_O_7_]^2−^ (W_2_)} (table S27). UV-vis spectroscopy records a gradual fade of the IVCT band for 24 hours at 37°C (fig. S82B).

*Hepes (0.1 M) (pH 7-8).* At pH 7, ^51^V NMR shows only small vanadates V_1_–V_3_ (H_x_V_2_ dominates) (fig. S53, tables S20 to S22 and S26, and fig. S76), while RR data reveal a W_7_ signal and a 701-cm^−1^ shoulder in aged samples (table S27), and the 390-nm IVCT band remains stable (fig. S82 C). At pH 8, the NMR spectrum still contains just V_1_–V_5_ (mainly V_1_) (figs. S53 and S76 and tables S20 to S22 and S26) and RR investigations again show W_7_ plus a 602-cm^−1^ line in the fresh sample and W_12_ fragments after heating (table S27). The IVCT band fades gradually for over 24 hours at 37°C, as in tris buffer (fig. S82D).

#### Mo versus W—Bottom line

Above pH 7, the Mo cage is effectively gone outside neat H_2_O, whereas the W cage still leaves a traceable IVCT band and Raman fingerprint and breaks down more slowly, forming mainly VW_5_/V_2_W_4_ clusters rather than the richer Mo–V blend seen for its Mo analog.

## DISCUSSION

The global survey (69 conditions, pH range from 1 to 8, four buffers, and two temperatures) shows that acidity is the primary gatekeeper. When the medium is strongly acidic (pH ≤ 4), the driving force for metal-oxide condensation is low, V centers remain in the paramagnetic V^IV^ state, and the four orthogonal probes—“silent” ^51^V NMR, sharp IVCT band, single RR fingerprint, and intact-anion peaks by ESI-MS—concur that the cage is intact. Once the pH drifts above 4, that state becomes thermodynamically uphill and Keplerate cages break (Fig. 3). Throughout, one experimental advantage stands out: Dissolving either Keplerate does not measurably shift bulk pH of buffered solutions (tables S7 and S19), eliminating a common source of artefacts in POM chemistry (*39*).

The very first detectable fragment in every medium is orthovanadate V_1_, the lowest-energy monomer in the V^V^ acid–base series. Its immediate appearance reflects fast kinetics, as the V─O─V links at the cage surface hydrolyze almost as soon as the pH is high enough. What follows is slower, thermodynamically driven reassembly: Over 24 hours at 25°C and 37°C, V_1_ condenses into higher vanadates and then into buffer-templated mixed-metal clusters. The products mirror those found in classical, nonreduced Mo, W, and V systems (e.g., decavanadate V_10_, heptamolybdate Mo_7_, octamolybdate Mo_8_, and paratungstate W_7_), confirming that once the cages open and are getting reduced, the chemistry reenters the well-mapped acidified POM landscape (*39*).

Within this overarching pathway, the addendum metal dictates lifetime and speciation. This disparity can be attributed to the known chemistry of Mo- and W-based POMs (*1*, *2*, *33*). Tungsten’s high oxophilicity and reluctance to change oxidation state limit {W_72_V_30_} speciation to fewer but more stable structures, such as Lindqvist-type VW_5_ and extend the intact window to pH between 3 and 4. Molybdenum, by contrast, is redox-flexible, its cage fragments earlier and into a richer mixture of V–Mo species. Buffers modulate, but never invert, that trend. Phosphate stabilizes the W cage at pH ≈ 2 yet offers no extra protection to the Mo cage. Acetate preserves the IVCT band but pushes Mo toward the V_10_ + α-VMo_7_ pair, whereas tris and Hepes at pH 7 to 8 accelerate oxidation, imprinting their characteristic molybdate or tungstate fingerprints onto the spectrum—even as neat water continues to show faint signals of intact cage.

The divergent decomposition patterns observed across buffer systems reflect three complementary effects: weak chelation, ligand templating, and microenvironmental modulation. Acetate, tris, and Hepes contain donor atoms (O, N, or SO_3_^−^) capable of temporally coordinating V^IV/V^, Mo^VI^, or W^VI^ centers exposed after initial V─O─M bond cleavage. These weak interactions lower the effective metal–oxygen bond stability, shift hydrolysis equilibria toward smaller fragments. While acetate and tris are small and moderately nucleophilic, Hepes is bulkier and less prone to coordination yet still influences speciation by stabilizing the solution at pH 7 to 8, where cage decomposition is thermodynamically favored. In both Mo and W systems, Hepes-buffered solutions predominantly yield small vanadates (e.g., V_1_–V_5_), with no notable formation of mixed Mo–V or W–V species, suggesting that Hepes neither stabilizes the cage nor promotes recombination into heterometallic clusters. In contrast, phosphate (a hard, multidentate oxo ligand) can cap exposed addendum-metal sites and stabilize partially opened cages. Consistent with this role, ^31^P NMR reveals direct phosphate incorporation into [P_2_Mo^V^_5_O_23_]^6−^ species once the Mo-cage begins to decompose at pH 2 to 3 (figs. S26 and S27). Last, each buffer system subtly alters local proton activity and ionic strength even at identical nominal pH, which in turn modulates the driving force for V^IV^ → V^V^ oxidation and metal-oxide reassembly. While phosphate leaves a clear ligand fingerprint, acetate, tris, and Hepes steer decomposition without direct incorporation but generate characteristic product distributions (table S31).

Temperature and time act as kinetic selectors. When the cage is robust (pH ≤ 3), heating from 25° to 37°C does almost nothing. Under more basic conditions, however, the same increase either tilts an existing Mo/W–vanadate equilibrium or, if the system is still evolving, unlocks additional hydrolysis and condensation pathways, enriching Lindqvist clusters at the expense of low-nuclearity vanadates. Aging at 25°C produces the same dichotomy, proving that heat mainly accelerates processes that would occur anyway on a longer timescale.

The larger experimental lesson is methodological: Optical methods alone are insufficient, although previous stability studies on Keplerate-type POMs, such as {Mo_132_} and {Mo_72_Fe_30_}, primarily used UV-vis and sometimes RR spectroscopy (*57*–*63*). UV-vis and Raman can report a “living” IVCT band and Mo═O stretch long after ^51^V NMR and RR show that the cage has vanished. Accurate mechanistic work therefore requires at least one structure-sensitive probe such as NMR or RR in addition to redox-sensitive UV-vis.

The speciation atlas now serves three immediate purposes. First, it recalibrates past catalytic or bioassay reports: For example, studies that assumed an intact Keplerate at pH 5 in acetate buffer can be revisited knowing the exact speciation. Second, practitioners can prospectively design solution conditions: work below pH 3 in phosphate to preserve a W cage for photoredox storage, or target pH 8 in tris/Hepes to harvest VW_5_/V_2_W_4_ as oxide-precursors. Third, the dataset provides benchmark spectra that computational chemists and spectroscopists can use for fingerprint-matching, accelerating identification of unknown POM intermediates in complex media. As an illustration, {Mo_72_V_30_} has been applied in catalysis in neat water at elevated temperatures (*18*) and as a precursor for MoS_2_-based electrocatalysts (*32*). Under aqueous conditions close to those entries in our atlas (H_2_O, pH ≈ 4; fig. S23 and table S11), the solution may contain not only the intact cage but also small vanadates and mixed Mo–V fragments. The atlas thus provides a framework for considering how such coexisting species might contribute to, or be leveraged in, catalytic or materials synthesis processes.

In summary, Keplerate nanocages represent an instructive microcosm of metal-oxide chemistry: Subtle shifts in proton activity, ligand shell, or temperature can redirect a 3-nm, 102-metal assembly into the canonical monomers and oligomers that underpin the wider POM family. By following that transformation with four truly orthogonal analytical techniques, we show (i) that speciation is hierarchical, in which orthovanadate appears first, higher vanadates and mixed-metal clusters later, and (ii) that addenda-metal rigidity, not just charge density, governs how long the parent structure persists. These insights generalize well beyond the Keplerates. They explain why buffer identity can make or break catalytic selectivity and why single-wavelength “stability tests” are unreliable for any reduced metal-oxide cluster. The FAIR data package released here (PHAIDRA https://phaidra.univie.ac.at/o:2143725, DOI: 10.25365/phaidra.694) converts those qualitative lessons into a quantitative lookup table, enabling researchers to design solution conditions that either lock a cage in place for bioinspired charge storage or harvest its fragments as precursors for oxide materials, all while reporting the full pH buffer–temperature context so future studies can build on, rather than repeat, the same chemical ground.

## MATERIALS AND METHODS

All chemicals used for buffer preparations were purchased from Sigma-Aldrich and used without further purification.

### Buffer preparation

In addition to studying the Keplerate speciation in distilled water in the pH range from 1 to 8, four different buffers were selected covering the pH range from 2 to 8 to ensure stable pH and study the influence of commonly used buffers on Keplerates’ speciation. In the acidic region (pH 2 to 6), two anionic buffers have been studied: Sodium phosphate buffers in the pH range 2 to 6 and acetic acid–sodium acetate in the pH range 4 to 5.5. In the neutral to moderately alkaline range (pH 7 to 8), two organic buffers have been studied: Hepes and tris-HCl buffers, both in the pH range 7 to 8. All buffers were prepared at a concentration of 0.1 M.

### POM synthesis and characterization

Na_8_K_16_(VO)(H_2_O)_5_[K_10_ ⊂ {(Mo^VI^)Mo^VI^_5_O_21_(H_2_O)_3_(SO_4_)}_12_(VO)_30_(H_2_O)_20_]∙150H_2_O ({Mo_72_V_30_}) and K_8_Na_28_[(W^VI^)W_5_^VI^(SO_3_)(H_2_O)_3_O_21_}_21_{V^IV^O(H_2_O)}_30_]∙ ~ 90H_2_O ({W_72_V_30_}), were synthesized according to the already established protocols (*7*, *53*). After obtaining the crystalline samples of both Keplerates, they were further characterized in the solid-state using infrared spectroscopy (fig. S4 and table S1) and by single-crystal X-ray diffraction (SXRD) unit cell determination (table S2).

### Speciation studies

For this study, we selected two Keplerate-type POMs, {Mo_72_V_30_} and {W_72_V_30_}. These POMs contain enough of paramagnetic V^IV^ that can oxidize to diamagnetic V^V^ upon degradation to smaller POM structures. This transformation enhances the detection sensitivity and reliability of ^51^V NMR measurements, enabling efficient analysis of solutions containing the resulting V^V^ species. The POMs also exhibit suitable solubility between 0.15 and 0.50 mM ({W_72_V_30_}), making them compatible with various analytical techniques in solutions.

We cover 69 different conditions and have a POM concentration of 0.15 mM because it is the maximum concentration of {Mo_72_V_30_}, except for 0.20 mM POM concentration in acetic acid—sodium acetate buffers. All solutions were tested immediately after preparation of the solution or within 1 hour of preparation (NMR experiments), after 24-hour aging at room temperature, and after 24-hour incubation at 37°C. As stated before, all buffers were prepared at a concentration of 0.1 M. The pH value of buffers and POM solutions for speciation study were determined using Thermo Fisher Orion Star A211 benchtop pH meter with a Hamilton Biotrod electrode, which was calibrated before each series of measurements with three pH points using standard buffers from Sigma-Aldrich for calibration with pH 2.00 (citric acid/sodium hydroxide/hydrogen chloride), 4.01 (potassium hydrogen phthalate), and 7.00 (disodium hydrogen phosphate/potassium dihydrogen phosphate).

The speciation of Keplerates was primarily studied using NMR (^51^V and ^31^P), UV-vis spectroscopy and RR spectroscopy on WITec alpha 300A equipped with 532-nm laser. In addition, ESI-MS thermal ionization MS–time of flight (timsTOF) on Bruker ESI/matrix-assisted laser desorption/ionization–Qq-TOF with dual-TIMS analyzer was used to prove the presence of intact Keplerate anion in distilled water (pH 1 to 8) in fresh samples. ^31^P and ^51^V NMR spectra were measured on an Avance Neo 500-MHz FT-NMR spectrometer (Bruker, Rheinstetten, Germany) at 25°C. Chemical shifts were measured relative to 85% H_3_PO_4_ and VOCl_3_.

## Supplementary Materials

### The PDF file includes:

Supplementary Text Figs. S1 to S83 Tables S1, S2, S5 to S31 Legends for tables S3 and S4 References
