# Supplementary material for: Speciation atlas of polyoxometalates in aqueous solution (Part II): Molybdenum browns
Source: Sci Adv. 2025 Oct 31;11(44):eaea1910. doi: 10.1126/sciadv.aea1910 (PMC12577712; doi:10.1126/sciadv.aea1910)
Supplement: Supplementary file 1 — Supplementary Text Figs. S1 to S83 Tables S1, S2, S5 to S31 Legends for tables S3 and S4 References [file sciadv.aea1910_sm.pdf]

Science Advances
AAAS

Supplementary Materials for

Speciation atlas of polyoxometalates in aqueous solution (Part II): Molybdenum browns

Ingrid Gregorovic *et al.*

Corresponding author: Nadiia I. Gumerova, nadiia.gumerova@univie.ac.at;
Annette Rompel, annette.rompel@univie.ac.at

Sci. Adv. **11**, eaea1910 (2025)
DOI: 10.1126/sciadv.aea1910

The PDF file includes:

Figs. S1 to S83
Supplementary Text
Tables S1 and S2, and S5 to S31
Legends for tables S3 and S4
Abbreviations
References

Other Supplementary Material for this manuscript includes the following:

Tables S3 and S4

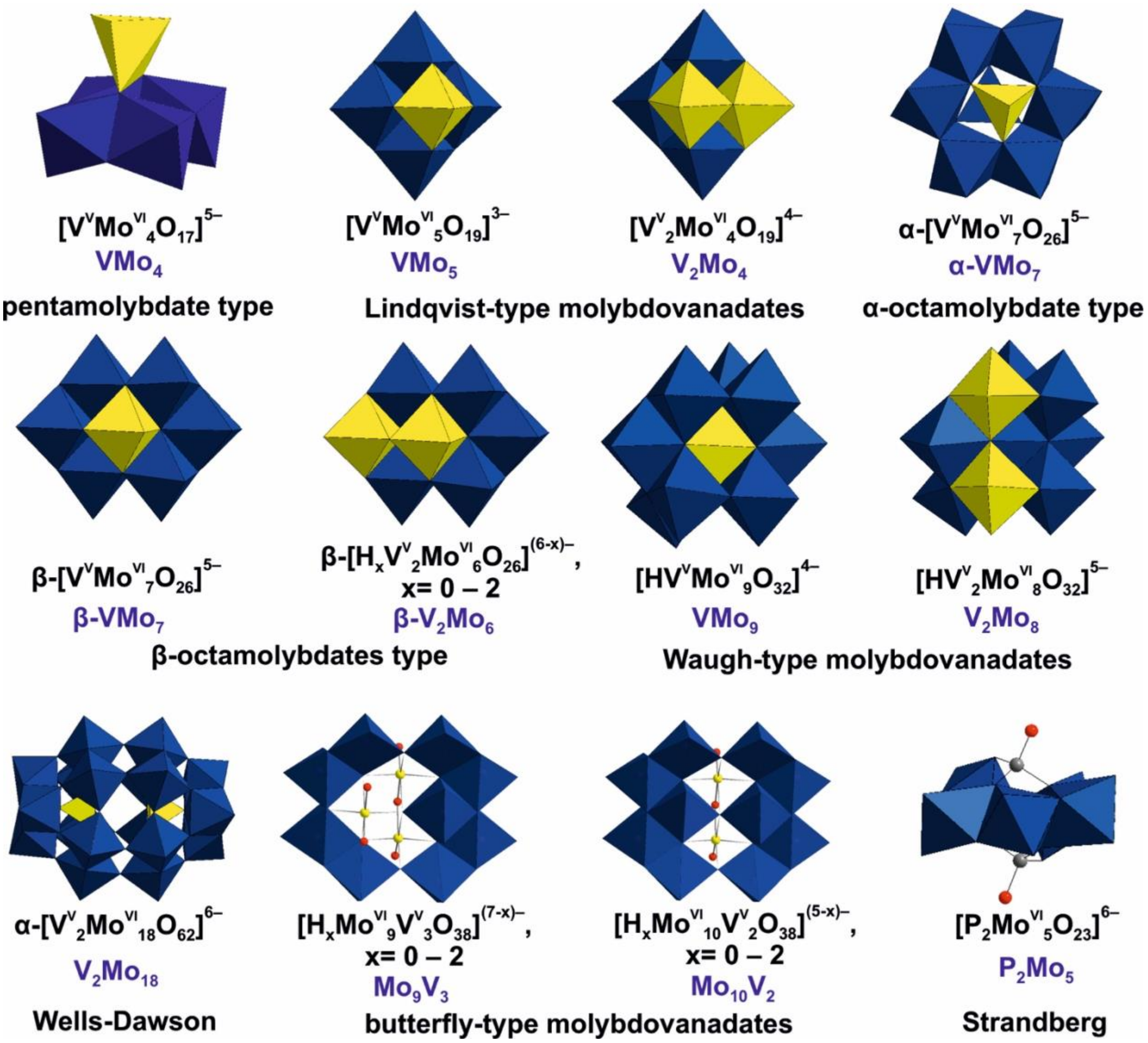

Fig. S1. Mo–based POMs with nuclearity M ≤ 36 where M = Mo and V. All Mo-V mixed-metal polyoxometalates identified by ^{51}V or ^{31}P-NMR formed in solution in this study after dissolving **$\{Mo_{72}V_{30}\}$** (0.15 mM) in H_2O (pH 1 – 8) and 0.1 M buffers (acetic acid – sodium acetate pH 4 – 5.5; sodium phosphate pH 2 – 8; Tris-HCl pH 7 – 8; HEPES pH 7 – 8). Color code: $\{MoO_6\}$, blue; $\{VO_6\}/\{VO_4\}$, yellow; P, gray; O, red.

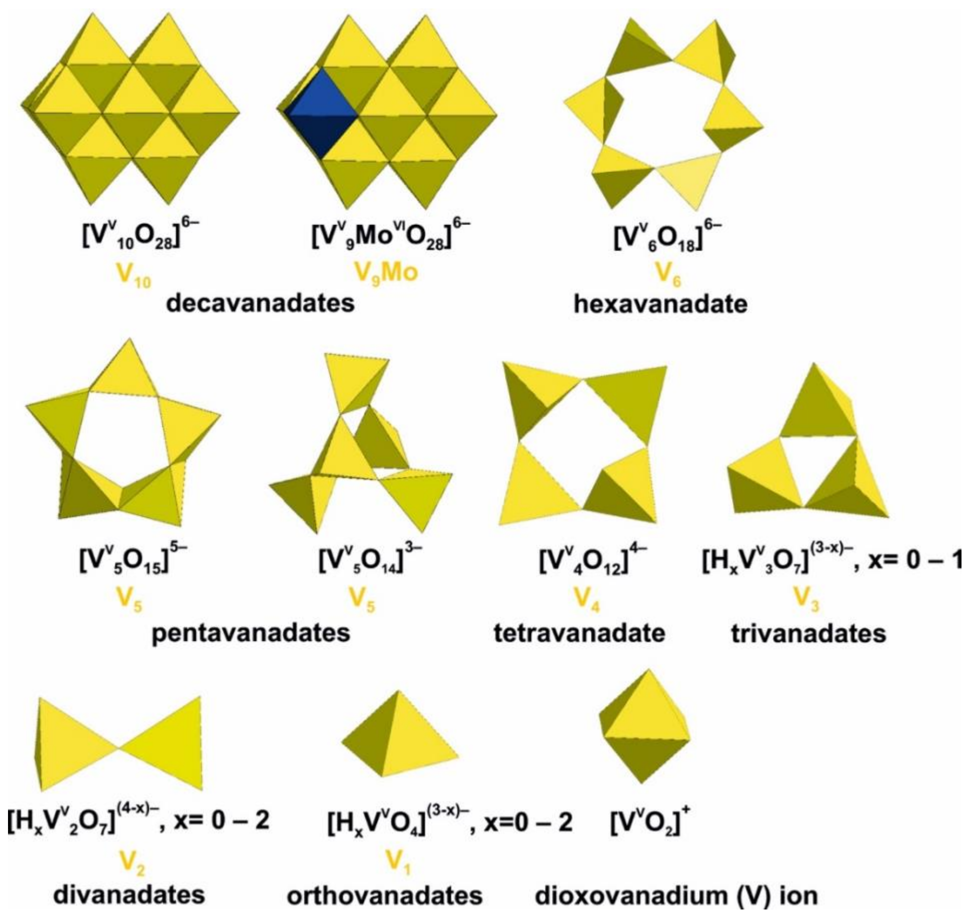

Fig. S2. V–based POMs with nuclearity M ≤ 36 where M = V. All V-based polyoxometalates identified by ^{51}V-NMR formed in solution after dissolving **{$Mo_{72}V_{30}$}** (0.15 mM) in H_2O (pH 1 – 8) and 0.1 M buffers (acetic acid – sodium acetate pH 4 – 5.5; sodium phosphate pH 2 – 8; Tris-HCl pH 7 – 8; HEPES pH 7 – 8). Color code: {MoO_6}, blue; {VO_6}/{VO_4}, yellow.

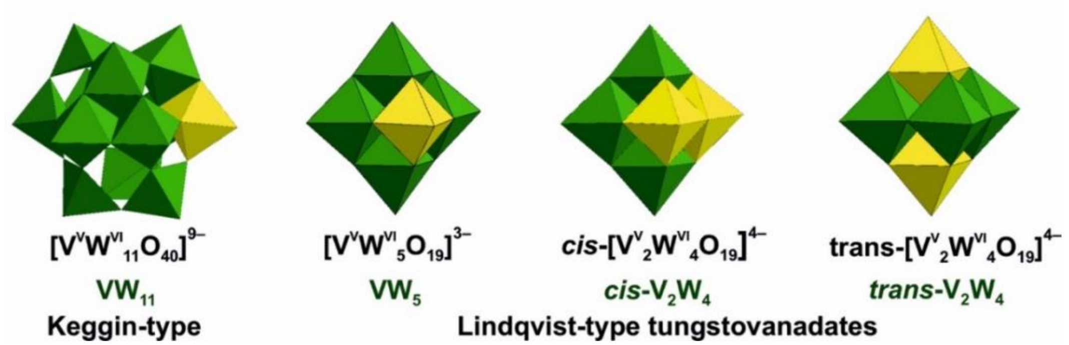

Fig. S3. W–based POMs with nuclearity M ≤ 36 where M = W and V. All W-based polyoxometalates identified by ^{51}V or ^{31}P-NMR in this study formed in solution after dissolving **{$W_{72}V_{30}$}** (0.15 mM) in H_2O (pH 1 – 8) and 0.1 M buffers (acetic acid – sodium acetate pH 4 – 5.5; sodium phosphate pH 2 – 8; Tris-HCl pH 7 – 8; HEPES pH 7 – 8). Color code: {WO_6}, green; {VO_6}, yellow.

2. Synthesis and characterization of polyoxometalates

$Na_8K_{16}(VO)(H_2O)_5[K_{10}\subset\{(Mo^{VI})Mo^{VI}{}_5O_{21}(H_2O)_3(SO_4)\}_{12}(V^{IV}O)_{30}(H_2O)_{20}]\cdot150H_2O$ (**{Mo72V30}**), (*7*) and $K_8Na_{28}[(W^{VI})W_5{}^{VI}(SO_3)(H_2O)_3O_{21}\}_{21}\{V^{IV}O(H_2O)\}_{30}]\cdot{\sim}90H_2O$ (**{W72V30}**), (*53*) were synthesized according to the procedures reported in the literature (*8, 53*). Both compounds were characterized in the solid state using infrared spectroscopy (IR, Section 2.1, **Table S1**) and SXRD for unit cell determination (**Table S2**).

2.1. IR spectroscopy

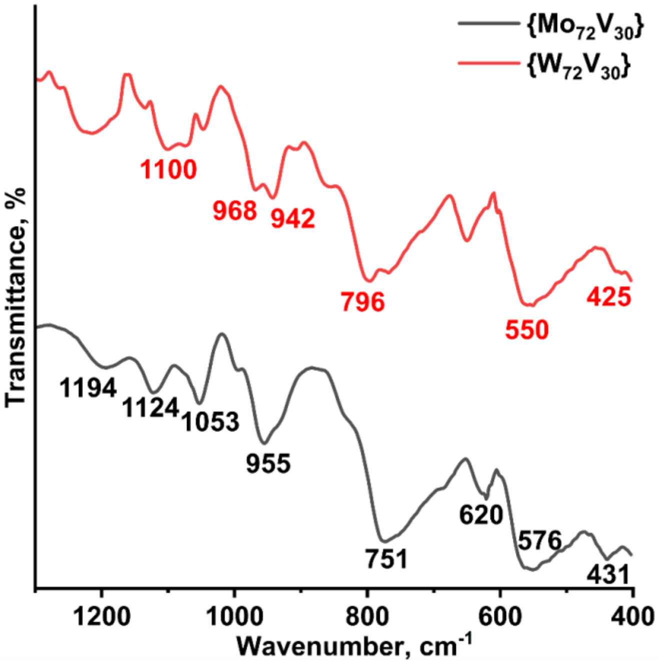

Fig. S4. IR spectra of Keplerate-type polyoxometalates. IR-spectra of two Keplerate type POMs – $Na_8K_{16}(VO)(H_2O)_5[K_{10}\subset\{(Mo^{V})Mo^{V}{}_5O_{21}(H_2O)_3(SO_4)\}_{12}(V^{IV}O)_{30}(H_2O)_{20}]\cdot150H_2O$ ({**Mo72V30**}) and $K_8Na_{28}[(W^{VI})W_5{}^{VI}(SO_3)(H_2O)_3O_{21}\}_{21}\{V^{IV}O(H_2O)\}_{30}]\cdot{\sim}90H_2O$ ({**W72V30**}) in the fingerprint range of 1200 – 400 cm^{-1} (**Table S1**).

Table S1. The positions and attribution of the observed band in the IR-spectra fingerprint region (1200 – 400 cm^{-1}) of Keplerate-type POMs included in the study.

The inconsistencies of some spectrum bands may be caused by different measurement conditions (e.g., using the KBr pellet in the literature vs. using the ATR module in our case), different hydrate forms, or the failure to indicate all the bands in the spectrum.

POM formula	Position and intensity of bands [cm^{-1}] (ATR module)	Position and intensity (if indicated) of bands [cm^{-1}] according to literature (KBr pellet)	Ref	Attribution
**{$Mo_{72}V_{30}$}**	1192, 1124, 1053	1198, 1128, 1054	(*7*)	^{v}S–O (SO_4)
	955	966		^{v}W=O_t/ ^{v}V=O_t
	751, 620, 546, 431	791, 631, 575, 449		^{v}Mo–O_b, ^{v}Mo–O_c
**{$W_{72}V_{30}$}**	1100	1101	(*53*)	^{v}S–O (SO_3)
	968	963		^{v}W=O_t/ ^{v}V=O_t
	942, 796, 550, 425	896, 766, 582, 446		^{v}W–O_b, ^{v}W–O_c

Legend: O_t – terminal oxygen, O_b – edge-bridging oxygen, O_c – corner-bridging oxygen.

2.2. <u>SXRD unit cell determination</u>

Table S2. SXRD unit cell determination: experimental results and literature unit cell of Keplerate-type POMs included in the study.

The inconsistencies between literature (checked at T = 200 K) and experimental (checked at room temperature) data may be caused by the difference in T, fragility of single crystals (data quality) and the different XRD devices used.

POM formula	Literature unit cell (T = 200 K)	Ref	Experimental unit cell (room temperature)
**{$Mo_{72}V_{30}$}**	C2/c<br>a= 47.177(3) Å<br>b= 42.460(3) Å<br>c= 26.4971(16) Å<br>α= 90°<br>β= 90.134(2)°<br>γ= 90°<br>Z= 4<br>V= 53078 Å^3	(*7*)	C2/c<br>a= 46.5696 Å<br>b= 42.0357 Å<br>c= 26.2231 Å<br>α= 90°<br>β= 90.0773°<br>γ= 90°<br>Z= 4<br>V= 52499 Å^3

**{$W_{72}V_{30}$}**	Cmca<br>a= 35.241(7) Å<br>b= 38.475(8) Å<br>c= 38.396(8) Å<br>α= 90°<br>β= 90°<br>γ= 90°<br>Z= 4<br>V= 52061 (18) $Å^3$	(*53*)	Cmca<br>a= 34.5098 Å<br>b= 37.7065 Å<br>c= 37.4363 Å<br>α= 90°<br>β= 89.9889°<br>γ= 90°<br>Z= 4<br>V= 50789 $Å^3$

3. Molybdenum Blues and Molybdenum Browns (Keplerates) and their solution-phase stability and speciation reported in the literature

Table S3. Literature-known large reduced POMs (with nuclearity > 50) organized by increasing nuclearity.

List of all reported large (with addenda nuclearity > 50) reduced POM structures and their stability and speciation in aqueous solution reported in the literature up to May 2025 (ref. *64–108*). The search was conducted using the Scopus document search engine (search words: giant polyoxometalates, molybdenum blues, molybdenum browns, Keplerates, wheel-type polyoxometalates, molybdenum wheels, $\{Mo_{154}\}$, $\{Mo_{132}\}$, reduced polyoxometalates, nanosized polyoxometalates, polyoxometalate capsules, high-nuclearity polyoxometalate, and molybdenum reds) and was limited to large POM structures containing Mo and W. When information is missing in the literature, it is marked as n.r. – not reported.

Table S3 is provided in the supplementary Excel file (Table S3.xlsx).

Table S4. Applications of Molybdenum Blues and Keplerate type POMs in solutions.

Application of all literature-known large reduced POM structures (with nucelearity > 50) in solutions. The search was conducted using the Scopus document search engine and was limited to large POM structures containing Mo (by combining search words from **Table S3** and following search words: applications, catalysis, sensor, electrochemistry, biomedical, biotechnology, analytical chemistry, material science, drug delivery, battery, multifunctional and biology). If POM is only mentioned in **Table S2**, no applications were reported in the literature by June 2025 (ref. *109–190*).

Table S4 is provided in the supplementary Excel file (Table S4.xlsx).

4. Literature-known NMR and Resonance Raman shifts for small polyoxometalates.

Table S5. List of all literature known ^{51}V NMR shifts for POMs with nuclearity ≤ 63 (as of October 2024).

If pH is not stated alongside a given ^{51}V NMR shift value, it indicates it was not reported in the original publication. The structures of POM anions identified during speciation study are shown in **Figures S1 – S3**. Underlined NMR shifts denote literature-referenced values used to assign our spectra. To enhance clarity and readability of the table, the graphical representation of ^{51}V NMR shifts listed in **Table S5** are provided in **Figures S5 – S7**.

**POM species**	**δ/ppm**	**Ref**
	**Polyoxovanadates**	
$[V^{V}O_4]^{3-}$	−541; −541.2; −536 (pH 14); one signal between −554 to −559 (pH 10 – 13); −537 (pH 9); or −556	(*54*), (*191*), (*192*), (*193*), (*201*)
$[V^{V}O_4H]^{2-}$	−538.8 (pK_a 13.4 or pK_a ≈ 12); −534 (pH 13); or one signal between −537 and −539 (pH 9.2–12.3)	(*54*), (*191*), (*192*), (*230*)
$[V^{V}O_4H_2]^{-}$	–560.4 (pK_a = 7.91 or pK_a = 7.1); −574 (pH 9, "VO_3^{-}"); or –560 (pH ≈ 7)	(*54*), (*191*), (*192*), (*194*)
$[H_xV^{V}O_4]^{(3-x)-}$, x=0-2	One signal between –560 to –548; –558; or one signal between −535 to −553 (pH 7–9);	(*195*), (*207*), (*211*)
$[V^{V}O_2]^{+}$	−545 (pH 0–1); –541 (pH < 3), or −544 (pH 1.3–2.8); −544.5; or −536.0	(*191*), (*196*), (*199*), (*201*), (*205*), (*206*)
$[V^{V}O_3]^{-}$ (NH_4^{+})	−605; or −573 (Na^{+}, pH 7.1)	(*197*), (*201*)
$[V^{V}{}_2O_7]^{4-}$	–561; one signal between –553 and –568 (pH 9–13); one signal between –527 to –534 (pH 9.5–13); –557 (pH 12); or −568	(*54*), (*191*), (*192*), (*198*), (*201*)
$[HV^{V}{}_2O_7]^{3-}$	–563.5 (pK_a = 9.74 or pK_a = 8.9); –562 (pH 9); or −564 (pH 9.2 – 9.6)	(*54*), (*191*), (*192*), (*230*)
$[H_2V^{V}{}_2O_7]^{2-}$	–572.7 (pK_a = 8.29 or pK_a = 7.2); −572.5 (pK_a = 7.2); or –572.5	(*191*), (*192*), (*194*)
$[H_xV_2O_7]^{(4-x)-}$, x=1 – 2	−570; or one signal between –558 and –568 (pH 7 – 9)	(*207*), (*211*)
$[V^{V}{}_3O_{10}]^{5-}$	–556.3 and –590.4 (pH 9–11); or one signal between–493 and –504 and one signal between –510 and –523 (pH 2.7–6.9)	(*54*), (*191*)
$[HV^{V}{}_3O_{10}]^{4-}$	*ca.* –570	(*192*)
$[V^{V}{}_4O_{12}]^{4-}$	−577.6 (pH 7); −577; one signal between −580 and −574; –573; −574; or −578 (pH 9.2–9.6)	(*54*), (*191*), (*194*), (*195*), (*198*), (*207*), (*230*)
$[V^{V}{}_4O_{13}]^{6-}$	One signal between –566 to –585 (pH 7 – 9); –569.1 and *ca.* –585; –569.1 (pH ≈ 9); −571 and −586 (pH 9.2–9.6)	(*191*), (*192*), (*230*)
$[HV^{V}{}_4O_{13}]^{5-}$	One signal between −566 and −585 (pK_a ≈ 8.9); or one signal between −560 and −590 (pK_a ≈ 9)	(*191*), (*192*)
$[H_2V^{V}{}_4O_{13}]^{4-}$	−597 and −605 (pH≤7.5)	(*192*)
$[V^{V}{}_5O_{15}]^{5-}$	−585; or −586	(*191*), (*192*), (*230*)
$[V^{V}{}_6O_{18}]^{6-}$	−589.4	(*192*)

$[V^{V}_{10}O_{28}]^{6-}$	–422, –496 and –513 (no V ratios);<br>–426 (2V), –508 (4V) and –527 (4V) (pH 2);<br>–427 (2V), –513 (4V) and –520 (4V) (pH 4);<br>one signal between –420 and –425, one signal between –500 and –514 and one signal between –510 and –519 (pH 3.5 – 5);<br>–426 (2V), –496 (4V) and –511 (4V) (pH 5.5);<br>–420 (2V), –495 (4V), –511 (4V) (pH 5.4);<br>–422 (2V), –496 (4V) and –513 (4V) (pH 8);<br>–418 (2V), –492 (4V) and –510 (4V) (pH 7);<br>−423 (2V), −495 (4V), −518 (4V)	(*54*), (*191*), (*198*), (*201*) (*207*)
$H[V^{V}_{10}O_{28}]^{5-}$	–424, –500 and –516 (pK_a 6.14, no V ratios);<br>one signal between –421 and –422, one signal between –495 and –500 and one signal between –512 and –517 (pH 4 – 5.8) (no V ratios)	(*54*), (*191*)
$H_2[V^{V}_{10}O_{28}]^{4-}$	–425, –506 and –524 (pK_a 3.86, no V ratios);<br>–422, –502 and –519 (pH 3) (no V ratios)	(*54*), (*191*)
$H_3[V^{V}_{10}O_{28}]^{3-}$	–427, –515 and –534 (pK_a 1.57) (no V ratios)	(*191*)
$[V^{V}_{15}O_{42}]^{9-}$	–507 (1V), –531 (2V), –584 (8V) and –597 (4V) (pH 3.5)	(*203*)
**Mixed vanadium-molybdenum POMs**		
$[V^{V}Mo^{VI}_5O_{19}]^{3-}$	–506 (pH 1 – 4); −505 (pH 4), −502 (pH 3); –506.4 (pK_a 3.74; pH 1.5–4.0)	(*196*), (*200*), (*201*), (*202*)
$[V^{V}_2Mo^{VI}_4O_{19}]^{4-}$	–494; −494.5; −495; –497.0 and –496.3 (pH 4); –498 (pH 1.4–7)	(*198*), (*199*), (*200*), (*201*), (*202*)
$[HV^{V}_2Mo^{VI}_4O_{19}]^{3-}$	–507; or −512.5 (pK_a 3.74)	(*201*), (*202*)
$[V^{V}Mo^{VI}_4O_{17}]^{5-}$	–567 (pH 1.4–7)	(*202*)
α-$[V^{V}Mo^{VI}_7O_{26}]^{5-}$	–502.9 (pH 3–4)	(*200*)
β-$[V^{V}Mo^{VI}_7O_{26}]^{5-}$	–534.2; or –537.2	(*200*), (*202*)
α-$[HV^{V}Mo^{VI}_7O_{26}]^{4-}$	–502.6	(*202*)
α-$[V^{V}_2Mo^{VI}_6O_{26}]^{6-}$	–487	(*199*)
α-$[HV^{V}_2Mo^{VI}_6O_{26}]^{5-}$	–500.2	(*202*)
$[V^{V}_2Mo^{VI}_6O_{26}]^{6-}$	–482 ± 3 (pH 5.5)	(*201*)
β-$[HV^{V}_2Mo^{VI}_6O_{26}]^{5-}$	–540.9 (pH 4.5–5.8)	(*202*)
β-$[H_2V^{V}_2Mo^{VI}_6O_{26}]^{4-}$	–539.3 (pH 1.7–3.0)	(*202*)
$[Mo^{VI}_4V^{V}_5O_{27}]^{5-}$	–440.0, –491.6, –501.6, –504.4 and –518.6	(*202*)
$[HV^{V}Mo^{VI}_9O_{32}]^{4-}$	–515	(*202*)
$[HV^{V}_2Mo^{VI}_8O_{32}]^{5-}$	–519	(*202*)
$[V^{V}Mo^{VI}_{12}O_{40}]^{3-}$	−554	(*196*)
$[V^{V}(V^{V}Mo^{VI}_{11})O_{40}]^{4-}$	–514 and –552	(*196*)
$[V^{V}(Mo_{10}V^{V}_2O_{40})]^{5-}$	–505, –525, –547.8 and –548.0	(*202*)
α-$[V^{V}_2Mo^{VI}_{18}O_{62}]^{6-}$	–527 (pH 1.3)	(*196*)

$[V^{V}_{9}Mo^{VI}O_{28}]^{5-}$	–422 (2V), –492 (2V), –501 (2V), –512 (2V), –522 or −521.5 (1V) (pH 5.5–6.25)	(*197*), (*201*)
$[V^{V}_{10-x}Mo^{VI}_{x}O_{28}]^{-6+x}$ (x = 1, 2)	–435 (2V), –486 (2V), –497 (2V), –500 (2V), –513 (2V); –449, –489, –498, –506, –514	(*201*)
$[V^{V}_{x}Mo^{VI}_{13-x}O_{40}]^{n-}$ (x = 1, 2)	−496, −498, −516, −522	(*201*)
$[Mo^{VI}_{9}V^{V}_{3}O_{38}]^{7-}$	–516	(*202*)
$[HMo^{VI}_{9}V^{V}_{3}O_{38}]^{6-}$	–522.6 (2V) and –525.8 (1V) (pK_a 3.2)	(*202*)
$[HMo^{VI}_{10}V^{V}_{2}O_{38}]^{5-}$	–526	(*202*)
$[H_3Mo_{57}V_6(NO)_6O_{189}(H_2O)_{12}(VO)_6]^{15-}$	–485.5 and –578.5	(*86*)
**Phosphovanadomolybdates and phosphovanadates**		
$[PV^{V}Mo^{VI}_{11}O_{40}]^{4-}$	–530; or –532 (pH 1.8)	(*204*), (*205*)
$[PV_2^{V}Mo^{VI}_{10}O_{40}]^{5-}$	–535;<br>–532, –542 and –546 (pH 1.8);	(*204*), (*205*)
$[PV^{V}_{3}Mo^{VI}_{9}O_{40}]^{6-}$	–538; –532, –542, –546, –554 and –585 (pH 1.8)	(*204*), (*205*)
$[PV_5^{V}Mo^{VI}_{7}O_{40}]^{8-}$	–532, –542, –546, –554, –585 and –595 (pH 1.8)	(*205*)
$[H_bPV^{V}_{14}O_{42}]^{(9-b)-}$	−529.3, −583.5 and −590.4	(*206*)
$[PV^{V}_{14}O_{42}]^{9-}$	−528.1, −580.0 and −593.8 (pH 1.7)	(*208*)
**Mixed vanadium-tungsten POMs**		
$[V^{V}W_5O_{19}]^{5-}$	–522 (pH 2); –509.6; −522; –509.7 (pH 5–7); –522 ± 0.3 (pH 2.8); or –526.4	(*198*), (*201*), (*208*), (*209*), (*211*), (*213*)
$[V^{V}_{2}W^{VI}_{4}O_{19}]^{4-}$	–507; –511; or –506 (pH 4, buffer)	(*198*), (*201*), (*214*)
cis-$[V^{V}_{2}W^{VI}_{4}O_{19}]^{4-}$	–510.5; –515.4 (pH 1.5–7); or –511.3	(*208*), (*210*), (*213*)
cis-$[HV^{V}_{2}W^{VI}_{4}O_{19}]^{3-}$	–527.8 (*pK$_a$* 2.23)	(*213*)
$[V^{V}_{3}W^{VI}_{3}O_{19}]^{5-}$	–497; or one signal between –494 and –499 (pH 7–9)	(*197*), (*210*)
trans-$[V^{V}_{2}W^{VI}_{4}O_{19}]^{4-}$	–518.4	(*213*)
trans-$[HV^{V}_{2}W^{VI}_{4}O_{19}]^{3-}$	ca. –528 (*pK$_a$* 2.5)	(*213*)
$[V^{V}_{4}W^{VI}_{9}O_{40}]^{6-}$	–506, −530 and –553 (pH 4)	(*211*), (*214*)
α-$[H_2V^{V}W^{VI}_{11}O_{40}]^{7-}$	–539 ± 1 (pH 4); or –544.3	(*212*), (*213*)
$[V^{V}_{3}W^{VI}_{10}O_{40}]^{5-}$	–506, –530 to –535, –558 (pH 2)	(*214*)
fac-$[V^{V}_{3}W^{VI}_{3}O_{19}]^{5-}$	–498.3	(*213*)
fac-$[HV^{V}_{3}W^{VI}_{3}O_{19}]^{4-}$	–506.0 (*pK$_a$* 6.99)	(*213*)
mer-$[V^{V}_{3}W^{VI}_{3}O_{19}]^{5-}$	–498.9 (1V) and –505.2 (2V)	(*213*)
mer-$[HV^{V}_{3}W^{VI}_{3}O_{19}]^{4-}$	–513.7 (1V) and –518.6 (2V) (*pK$_a$* 7.99)	(*213*)
$[V^{V}_{9}W^{VI}O_{28}]^{5-}$	–424 (2V), –492 (2V), –503 (2V), –511 (2V), –522 (1V); or –428.2 (2V), –496.2 (2V), –507.8 (2V), –515.4 (1V), –518.6 (1V) and –525.7 (1V)	(*201*), (*213*)

$[HV^{V}_{9}W^{VI}O_{28}]^{4-}$	–428.3 (2V), –514.0 (2V), –507.8 (2V), –520.9 (1V), –532.6 (1V) and –533.0 (1V) (*pK_a* 2.15)	(*213*)
$[H_xV^{V}_{5}W^{VI}_{4}O_{27}]^{x-5}$ (x=0 or 1)	–428.7 (1V), –509.2 (2V), –520.1 (1V) and–528.6 (1V) (pK_a 2.5–3.7)	(*213*)
**Phosphovanadotungstates**		
$[PV^{V}W^{VI}_{11}O_{40}]^{4-}$	−557.3; or −545 ± 2	(*208*), (*214*)
$[PV^{V}_{2}W^{VI}_{10}O_{40}]^{5-}$	−547 and −553 to −557	(*214*)
α-$[PV^{V}_{3}W^{VI}_{9}O_{40}]^{6-}$	−566.1	(*208*)
$[HPV^{V}_{4}W^{VI}_{8}O_{40}]^{6-}$	−561	(*214*)

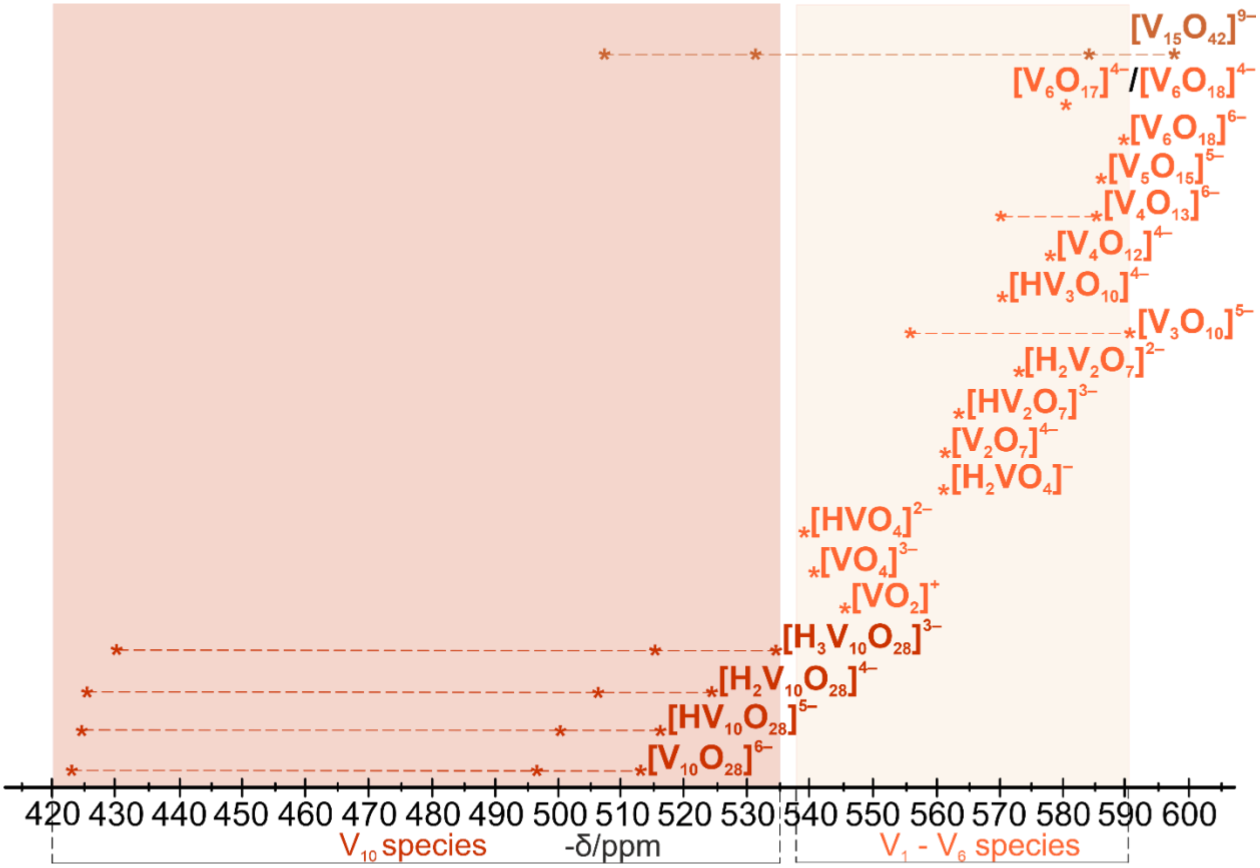

Fig. S5. Graphical summary of ^{51}V NMR chemical shifts for iso(poly)vanadates from Table S5.

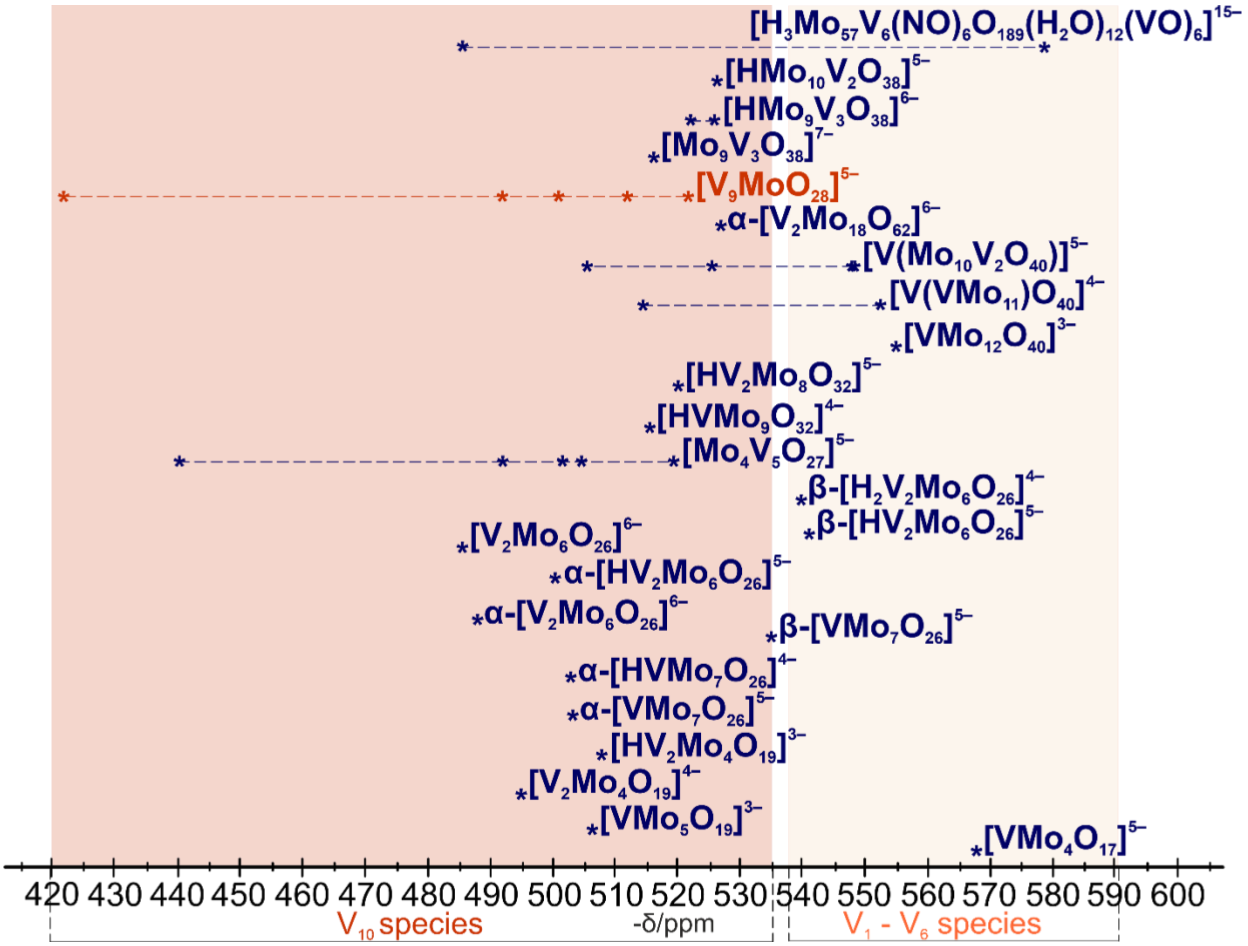

Fig. S6. Graphical summary of ^{51}V NMR chemical shifts for mixed V–Mo polyoxometalates from Table S5.

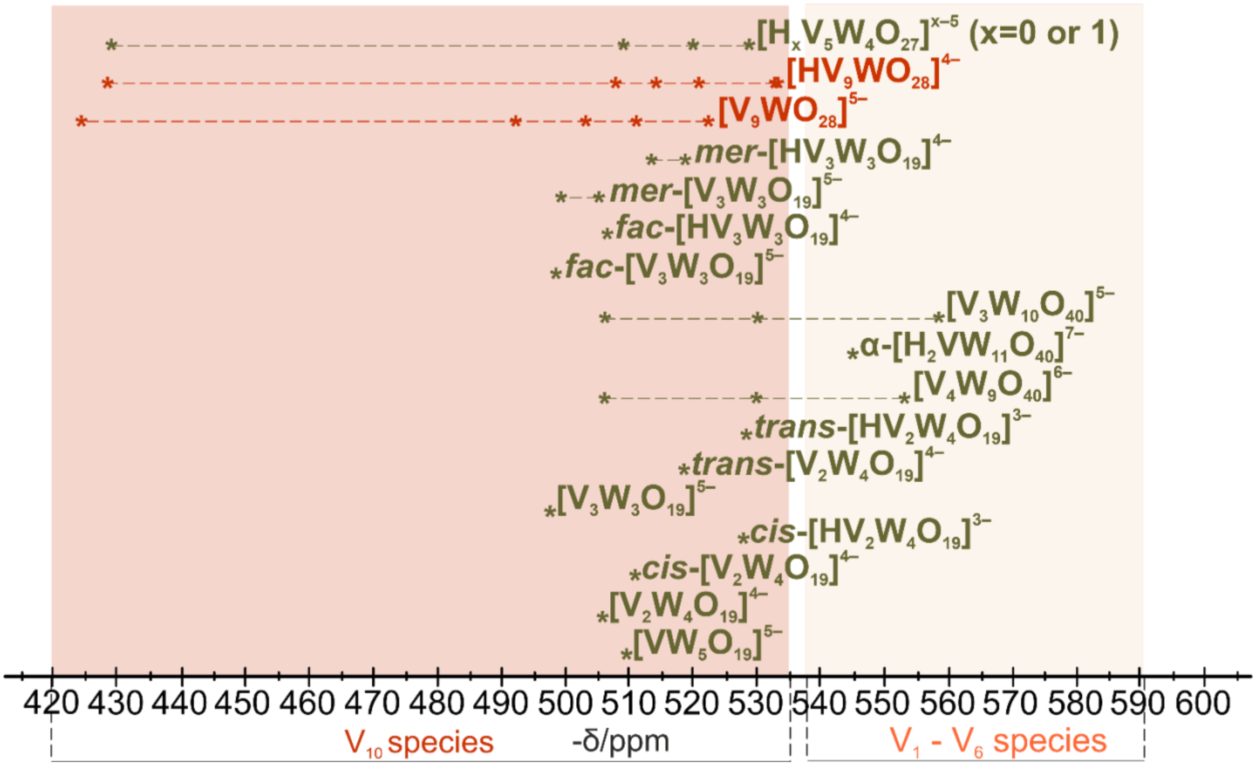

Fig. S7. Graphical summary of ^{51}V NMR chemical shifts for mixed V–W polyoxometalates from Table S5.

Table S6. List of literature-known Resonance Raman (RR) shifts for polyoxometalates (as of October 2024). Absence of solvent and pH information indicates that these parameters were not provided in the cited publication. RR measurements performed on solid samples are noted in the measurement conditions column. Main RR peaks specified in the literature are underlined in the table.

Species	Wavenumber/ cm^{-1}	Assignations	Measurement conditions	Ref.
**Isopolymolybdates**				
$[Mo^{VI}O_4]^{2-}$	318, 854, 896	O–Mo–O bend, Mo–O asymmetric stretch, Mo–O symmetric stretch	514.5 nm laser	(*215*)
	328, 850, 898		457.9 nm laser	(*216*)
	320, 830 – 855, 890 – 903		spectrophotometer with Cary 81 laser (λ not specified, but probably 785 nm laser), 0.1 – 1 M $[MoO_4]^{2-}$ solutions, pH 7 – 14	(*217*)
	317, 896	O–Mo–O bend, Mo–O symmetric stretch	785 nm laser, t = 30 s, pH 7 – 9	(*218*)
$H_2Mo^{VI}O_4$	919	n. r.	pH < 1	(*218*)
	225, 250, 313, 403, 919, 958	Mo–O stretch	spectrophotometer with Cary 81 laser (λ not specified, but probably 785 nm laser), $[MoO_4]^{2-}$ in conc. HCl	(*219*)
$[Mo^{VI}_3O_{10}]^{2-}$	874, 901, 918, 950	Mo=O asymmetric stretch	488 nm laser, pH 3.5 – 5	(*220*)
	946 – 950	Mo=O asymmetric stretch	785 nm laser, pH 4 – 5	(*218*)
$Mo^{V/VI}_4O_{11}$	183, 206, 229, 264, 306, 340, 380, 431, 452, 498, 568, 744(sh), 790, 835, 843, 907, 985	O–Mo–O asymmetric, O–Mo–O asymmetric, O–Mo–O symmetric, O–Mo–O symmetric, Mo–O_t asymmetric, Mo–O_t symmetric	632.8 nm laser, solid state	(*221*)
$[Mo^{VI}_5O_{17}]^{4-}$	812, 868, 908, 960	Mo–O asymmetric stretch	532 nm laser, 80 mW, solid state	(*222*)
$[Mo^{VI}_6O_{19}]^{2-}$	224/306, 620, 991	O_b–Mo–O_b bend, O_b–Mo–O_t bend, Mo–O_t symmetric stretch	785 nm laser, solid state	(*223*)
	285, 598, 817, 986	O_b–Mo–O_b bend, O_b–Mo–O_t bend, Mo–O_t asymmetric, Mo–O_t symmetric stretch	514.5 nm laser (in DMF)	(*224*)
	958, 985	Mo=O asymmetric stretch	488 nm laser	(*220*)
$[Mo^{VI}_7O_{24}]^{6-}$	939	Mo=O asymmetric stretch	488 nm laser, pH 4 – 6	(*220*)
	180, 208, 450, 939	Mo–O–Mo deformations, Mo–O–Mo deformations, Mo–O–Mo symmetric stretch, Mo=O stretch	514.5 laser, E(laser) = 100 mW, pH 7.0	(*215*)
	360, 898, 950	Mo–O–Mo bend, Mo–O–Mo asymmetric	457.9 nm laser	(*216*)

		stretch, Mo−O$_t$ symmetric stretch		
	170, 210 – 219, 361 – 362, 420 – 430, 555 – 560, 790, 943	Mo–O–Mo deformations, Mo–O–Mo deformations, Mo=O bend, Mo–O–Mo symmetric stretch, Mo–O–Mo symmetric stretch, Mo–O–Mo asymmetric stretch Mo=O stretch	spectrophotometer with Cary 81 laser (λ not specified, but probably 785 nm laser), pH 8, 0.1 – 1 M $[MoO_4]^{2-}$ solutions	(*217*)
	939	Mo=O asymmetric stretch	785 nm laser, t = 30 s, pH 5 – 6	(*218*)
	115, 215, 245, 358, 444, 550, 628, 893, 940	Mo–O–Mo deformations, Mo=O bend, Mo–O–Mo symmetric stretch, Mo–O–Mo symmetric stretch, Mo–O$_t$ symmetric stretch, Mo=O asymmetric stretch	spectrophotometer with Cary 81 laser (λ not specified, but probably 785 nm laser)	(*219*)
	895, 939	Mo–O$_t$ symmetric stretch, Mo=O asymmetric stretch	785 nm laser, t = 1 – 10 min	(*225*)
$[HMo^{VI}{}_7O_{24}]^{5-}$	948	Mo=O asymmetric stretch	785 nm laser, t = 30 s, pH 4	(*218*)
$[Mo^{VI}{}_8O_{26}]^{4-}$	200, 363, 844, 913, 958, 973	Mo–O–Mo deformations, Mo=O bend, Mo–O–Mo asymmetric stretch, Mo=O stretch, Mo=O stretch	514.5 nm laser, E(laser) = 100 mW, pH 1.8 – 2.2	(*215*)
	210, 370, 855, 920 – 925, 960 − 965	Mo–O–Mo deformations, Mo=O bend, Mo–O–Mo asymmetric stretch, Mo=O stretch, Mo=O stretch	spectrophotometer with Cary 81 laser (λ not specified, but probably 785 nm laser), pH 1.7 – 2.2	(*217*)
	200, 360, 850, 916, 961	Mo–O stretch	spectrophotometer with Cary 81 laser (λ not specified, but probably 785 nm laser)	(*219*)
	910 – 918, 956, 969	Mo–O$_t$ asymmetric stretch, Mo=O asymmetric stretch	785 nm laser, t = 1 – 10 min	(*225*)
α–$[Mo^{VI}{}_8O_{26}]^{4-}$	918, 959	Mo=O asymmetric stretch	488 nm laser, pH 2 – 5	(*220*)
	958	Mo=O asymmetric stretch	785 nm laser, t = 30 s, pH < 4	(*218*)
	915, 950 – 952	Mo–O$_t$ asymmetric stretch, Mo–O$_t$ symmetric stretch	in mixed MoV solutions, 785 nm laser, t = 1 – 10 min	(*225*)
β–$[Mo^{VI}{}_8O_{26}]^{4-}$	904, 915, 943, 971	Mo=O asymmetric stretch	488 nm laser, pH 1.5 – 4	(*220*)
	969	Mo=O asymmetric stretch	785 nm laser, t = 30 s, pH < 4	(*218*)
	966 – 968	Mo–O$_t$ asymmetric stretch	in mixed MoV solutions, 785 nm laser, t = 1 – 10 min	(*225*)
γ–$[Mo^{VI}{}_8O_{26}]^{4-}$	840, 912, 922, 943, 963	Mo=O asymmetric stretch	488 nm laser	(*220*)
$[Mo^{VI}{}_{36}O_{118}]^{8-}$	210, 363, 450, 827, 933, 953	Mo–O–Mo deformations, Mo=O bend, Mo–O–Mo	514.5 nm laser, E(laser) = 100 mW, pH 0.3	(*215*)

		asymmetric stretch, Mo=O stretch, Mo=O stretch		
	899, 957, 983	Mo=O asymmetric stretch	488 nm laser, pH 0.5 – 1.5	(*220*)
	849, 895, 955, 981	Mo–O stretches	785 nm laser, t = 30 s, pH<1	(*218*)
$\{Mo^{VI/V}_{154}\}$	221, 326, 462, 536, 806	n. r.	1064 nm laser, solid state	(*73*)
	1 band between 800 - 1000, 4 sharp bands between 200 - 550	$Mo-O_t$ symmetric stretch, Mo−O−Mo stretch	1064 nm laser	(*48*)
$\{Mo^{VI/V}_{132}\}$	316, 376, 842 – 875, 945	$Mo-O_t$ bend, Mo−O−Mo symmetric stretch, $O-Mo-O_t$ symmetric + asymmetric stretch, $Mo-O_t$ symmetric + asymmetric stretch	633 nm laser, solid state	(*226*)
	211, 321, 374, 468, 564, 876, 951	n.r.	1064 nm laser, E(laser) = 174 mW, solid state	(*15*)
$\{Mo^{VI/V}_{138}\}$	322 – 372, 439 – 532, 653 – 717, 820, 961	$Mo-O_t$ bend, Mo−O−Mo symmetric stretch, Mo–O−Mo asymmetric stretch, $O-Mo-O_t$ stretching, $Mo-O_t$ symmetric + asymmetric stretch	488 nm laser, solid state	(*226*)
$\{Mo^{VI}_{72}Fe^{III}_{30}\}$	379, 452 – 586, 717, 842 – 952, 969	$Mo-O_t$ bend, Mo−O−Mo symmetric stretch, Mo−O−Mo asymmetric stretch, $O-Mo-O_t$ stretch, $Mo-O_t$ symmetric + asymmetric stretch	633 nm laser, solid state	(*226*)
$\{Mo^{VI}_{72}V^{V}_{30}\}$	872, 941	V=O/ Mo=O stretch	1064 nm laser, solid state	(*227*)
	841, 911, 925	n. r.	n. r.	(*18*)
	870	V=O/ Mo=O stretch	in mixed MoV solutions, 785 nm laser, t = 1 – 10 min	(*225*)
**Isopolyvanadates**				
$[V^{V}_{10}O_{28}]^{6-}$	251, 322, 470, 534, 591, 749, 839, 973, 1001	O–V–O bend, breathing, O–V–O bends and V rocks, V–O–V asymmetric stretch, O–V–O and V–O–V asymmetric stretch, V–O symmetric stretch, V–O symmetric stretch	$[V_{10}]$ = 3 mM, 485 nm laser, E(laser) = 30 mW	(*228*)
	261, 331, 478, 541, 617, 748, 835, 997, 1053			
	315, 593, 967, 995	O–V–O bends and V rocks, V–O symmetric stretch	pH 4 – 8.59, $[VO_3^-]$ = 50 mM, 514.5 nm laser, E(laser) = 50 mW	(*229*)
	185, 210, 251, 324, 458, 547,	V=O bend, V–O–V asymmetric and	Spectrophotometer with Cary 81 laser (λ not	(*217*)

	600, 840, 920, 960, 970, 994	symmetric stretches, V=O stretch	specified, but probably 785 nm laser), pH 4.0	
$[V^{V}O_4]^{3-}$	820	V–O symmetric stretch	$[VO_3^-]$ = 50 mM, 514.5 nm laser, E(laser) = 50 mW	(*229*), (*230*)
$[V^{V}O_3]^{-}$	994	V=O stretch	in mixed MoV solutions, 785 nm laser, t = 1 – 10 min	(*225*)
$[HV^{V}O_4]^{2-}$	834	V=O stretch	$[VO_4^{2-}]$ = 50 mM, 514.5 nm laser, E(laser) = 100 – 200 mW	(*230*)
$[V^{V}{}_2O_7]^{4-}$	228, 351, 503, 810, 850, 877	V=O bend, V–O–V asymmetric and symmetric stretches, V=O stretch	Spectrophotometer with Cary 81 laser (λ not specified, but probably 785 nm laser), pH 7 – 14	(*217*)
	875	V–O symmetric stretch	$[VO_3^-]$ = 50 mM, 514.5 nm laser, E(laser) = 50 mW	(*229*)
	870	*$HV_1 + V_2$, V=O symmetric stretch	$[VO_4^{2-}]$ = 50 mM, 514.5 nm laser, E(laser) = 100 – 200 mW, pH 9 – 12	(*230*)
$[V^{V}{}_3O_{10}]^{5-}$	851	V–O symmetric stretch	$[VO_4^{2-}]$ = 50 mM, 514.5 nm laser, E(laser) = 100 – 200 mW	(*230*)
$[V^{V}{}_4O_{12}]^{4-}$	947	V–O symmetric stretch	$[VO_3^-]$ = 50 mM, 514.5 nm laser, E(laser) = 50 mW	(*229*)
	945	V–O symmetric stretch, *could also be mixture of $[V^{V}{}_4O_{12}]^{4-}$, $[V^{V}{}_5O_{15}]^{5-}$ and $[V^{V}{}_6O_{18}]^{6-}$ (pH < 9.2)	$[VO_4^{2-}]$ = 50 mM, 514.5 nm laser, E(laser) = 100 – 200 mW	(*230*)
	900	V–O symmetric stretch	$[VO_4^{2-}]$ = 50 mM, 514.5 nm laser, E(laser) = 100 – 200 mW	(*230*)
$[V^{V}{}_4O_{13}]^{6-}$	861	V–O symmetric stretch	$[VO_4^{2-}]$ = 50 mM, 514.5 nm laser, E(laser) = 100 – 200 mW	(*230*)
$[V^{V}{}_5O_{15}]^{5-}$	947	V–O symmetric stretch	$[VO_3^-]$ = 50 mM, 514.5 nm laser, E(laser) = 50 mW	(*229*)
	945	V–O symmetric stretch, *could also be mixture of $[V^{V}{}_4O_{12}]^{4-}$, $[V^{V}{}_5O_{15}]^{5-}$ and $[V^{V}{}_6O_{18}]^{6-}$ (pH < 9.2)	$[VO_4^{2-}]$ = 50 mM, 514.5 nm laser, E(laser) = 100 – 200 mW	(*230*)
	900	V–O symmetric stretch	$[VO_4^{2-}]$ = 50 mM, 514.5 nm laser, E(laser) = 100 – 200 mW	(*230*)
$[V^{V}{}_6O_{18}]^{6-}$	945	V–O symmetric stretch, *(*228*): could also be mixture of $[V^{V}{}_4O_{12}]^{4-}$, $[V^{V}{}_5O_{15}]^{5-}$ and $[V^{V}{}_6O_{18}]^{6-}$ (pH < 9.2)	$[VO_4^{2-}]$ = 50 mM, 514.5 nm laser, E(laser) = 100 – 200 mW	(*229*)
	900	V–O symmetric stretch	$[VO_4^{2-}]$ = 50 mM, 514.5 nm laser, E(laser) = 100 – 200 mW	(*230*)
**Isopolytungstates**				

$[W^{VI}O_4]^{2-}$	325 / 330, 838 / 834, 931 / 930	W–O bend, W–O asymmetric stretch, W–O symmetric stretch	514.5 nm laser, E(laser) = 100 mW	(*231*), (*215*)
	931	W=O symmetric stretch	514.5 nm laser	(*232*), (*233*), (*234*)
$W^{VI}O_3 - nH_2O$	300 – 550	H_2O libration	514.5 nm, E= 200 mW	(*233*)
$[W^{VI}{}_2O_7]^{2-}$	930	W=O symmetric stretch	514.5 nm laser	(*232*)
$[W^{VI}{}_6O_{19}]^{2-}$	965 – 975	W–O stretch	514.5 nm laser	(*232*), (*235*)
	234, 992	W–O stretch	514.5 nm, E(laser) = 200 mW	(*233*)
	996	W–O stretch	514.5 nm laser, in DMF	(*234*)
$[W^{VI}{}_6O_{20}(OH)]^{5-}$	170, 310, 358, 485, 571, 640, 902, 961	W–O bend, W–O bend, W–O asymmetric stretch	514.5 nm laser	(*231*)
$[HW^{VI}{}_6O_{21}]^{5-}$	960	W–O stretch	514.5 nm laser	(*232*), (*235*)
	420, 941, 980	W=O stretch	514.5 nm laser, 0.1 M solution, pH 5 – 6	(*215*)
$[W^{VI}{}_7O_{24}]^{6-}$	960	W–O stretch	514.5 nm laser	(*232*), (*236*)
	903, 967	W–O symmetric stretch, W–O asymmetric stretches	514.5 nm laser, pH 5.8 and 6.8	(*234*)
$[HW^{VI}{}_7O_{24}]^{5-}$	965 – 975	W–O stretch	514.5 nm laser	(*232*), (*236*)
$[W^{VI}{}_{10}O_{32}]^{4-}$	995	W–O stretch	514.5 nm laser	(*232*), (*233*)
$[W^{VI}{}_{12}O_{36}(OH)_{10}]^{10-}$ [a]	200, 310, 360, 400, 625, 900, 927, 957	W–O bend, W=O stretch	514.5 nm laser, 0.1 M solution, pH 5 – 6	(*215*)
$[W^{VI}{}_{12}O_{38}(OH)_2]^{6-}$	652, 885, 934, 960, 976	W–O symmetric stretch, W–O asymmetric stretches	514.5 nm laser	(*232*)
$[W^{VI}{}_{12}O_{39}]^{6-}$	961 – 980	W–O asymmetric stretch	514.5 nm laser	(*232*), (*233*)
$[W^{VI}{}_{12}O_{40}]^{8-}$ [b]	109, 133, 161, 217, 238, 311, 360, 656, 892, 912, 965	W=O wagging (out-of-plane), O–W–O twist (out-of-plane), O_t–W–O (in-plane), W–O–W bend (out-of-plane), W–O–W bend (in-plane), W=O scissoring (in-plane), W=O bend (out-of-plane), W–O–W symmetric stretch, W=O_t stretch (symmetric), W=O_t stretch (asymmetric)	785 nm laser, E(laser) = 10 mW	(*237*)
$[W^{VI}{}_{12}O_{41}]^{10-}$	900	W–O symmetric stretch	514.5 nm laser	(*232*), (*233*)
$[W^{VI}{}_{12}O_{42}]^{12-}$	197, 225, 320, 560, 615, 906, 962	W–O bend, W–O stretch	spectrophotometer with Cary 81 laser (λ not	(*217*)

			specified, but probably 785 nm laser), pH 7.8	
$[H_2W^{VI}_{12}O_{42}]^{10-}$	356, 403, 562, 580, 722, 857, 873, 883, 899, 911, 934, 942, 956	O–W–O bend, O–W–O bend, W–O–W stretch (edge, corner), W–O–W stretch (edge, corner), W–O–W stretch (corner), W–O–W stretch (edge), W–O–W stretch (edge), W–O–W stretch (edge), W–O–W stretch (edge), W–O–W stretch (edge), W=O_t stretch, W=O_t stretch, W=O_t stretch	532 nm laser	(*238*)
	350, 359, 405, 497, 557, 580, 644, 724, 857, 874, 886, 891, 908, 936, 955	O–W–O bend, O–W–O bend, O–W–O bend, W–O–W stretch (edge, corner), W–O–W stretch (edge, corner), W–O–W stretch (edge, corner), W–O–W stretch (edge, corner), W–O–W stretch (corner), W–O–W stretch (edge), W–O–W stretch (edge), W–O–W stretch (edge), W–O–W stretch (edge), W–O–W stretch (edge), W=O_t stretch, W=O_t stretch	785 nm laser	(*238*)
	164, 196, 225, 310, 366, 560, 615, 906, 945, 962	n. r.	spectrophotometer with Cary 81 laser (λ not specified, but probably 785 nm laser), pH 5.7	(*217*)
$[W^{VI}_{24}O_{72}(OH)_{12}]^{12-}$	209, 362, 516, 645, 886, 963	W–O bend, W–O asymmetric stretch	514.5 nm laser	(*231*)
$\{W^{VI}_{72}V^{V}_{30}\}$	876, 987	W=O stretch	514 nm laser	(*53*)

Legend:
a – $[W^{VI}_{12}O_{36}(OH)_{10}]^{10-}$ is referred as paratungstate B in publication, but it is not correct formula for paratungstate B.
b – the SXRD structure in publication shows structure of paratungstate B, but sum formula must be $[W_{12}O_{40}(OH)_2]^{10-}$. The formulas in Table S6. are shown in the same way as authors wrote in publication.
Explanation of RR M–O assignation abbreviations: O_t – terminal oxygen, O_b – edge-bridging oxygen, O_c – corner-bridging oxygen.

5. Spectroscopic study of $\{Mo_{72}V_{30}\}$ Keplerate-type polyoxometalate

5.1. pH of $\{Mo_{72}V_{30}\}$ solutions

Table S7. pH in $\{Mo_{72}V_{30}\}$ solutions.

pH measured in triplicate of 0.15 mM **$\{Mo_{72}V_{30}\}$** dissolved in 10% D_2O/H_2O (pH 1 – 8) and 0.1 M buffers (acetic acid – sodium acetate pH 4 – 5.5; sodium phosphate pH 2 – 8 (while phosphate does not buffer at pH range from 3.5 – 5.5, experiments were conducted at this pH to provide comparisons to previously published studies (*239*)); Tris-HCl pH 7 – 8; HEPES pH 7 – 8). The initial pH just in distilled water is ~3.8 for **$\{Mo_{72}V_{30}\}$**, with no measurable change over 72 h without external pH adjustment.

**pH**	**$\{Mo_{72}V_{30}\}$ (0.15 mM) in Solvent / 0.1M Buffer**	**pH after dissolving $\{M_{72}V_{30}\}$**				**pH after 24 h aging of $\{Mo_{72}V_{30}\}$ at RT[b]**				**pH after 24 h incubation of $\{Mo_{72}V_{30}\}$ at 37 °C**			
		**Sample**			**Mean of 1 to 3 ± SD[a]**	**Sample**			**Mean of 1 to 3 ± SD[a]**	**Sample**			**Mean of 1 to 3 ± SD[a]**
		#1	#2	#3		#1	#2	#3		#1	#2	#3	
Strongly acidic environment 1 ≤ pH ≤ 4													
1	10% D_2O/H_2O pH 1	1.08	1.06	1.07	1.07 ± 0.01	1.04	1.13	1.04	1.07 ± 0.05	1.25	1.08	0.98	1.10 ± 0.14
1.5	10% D_2O/H_2O pH 1.5	1.57	1.59	1.54	1.57 ± 0.03	1.63	1.58	1.68	1.63 ± 0.05	1.54	1.55	1.62	1.57 ± 0.04
2	10% D_2O/H_2O pH 2	2.06	2.07	2.07	2.07 ± 0.01	2.11	2.07	2.15	2.11 ± 0.04	2.11	2.07	2.22	2.13 ± 0.08
	sodium phosphate ($H_2PO_4^-$ /H_3PO_4) pH 2	2.09	2.09	2.09	2.09 ± 0.00	2.13	2.14	2.13	2.13 ± 0.01	2.11	2.12	2.11	2.11 ± 0.01
3	10% D_2O/H_2O pH 3	2.99	3.07	3.05	3.04 ± 0.04	3.01	3.10	3.04	3.05 ± 0.05	3.18	3.06	3.03	3.09 ± 0.08
	sodium phosphate pH 3	3.09	3.07	3.07	3.08 ± 0.01	3.15	3.10	3.11	3.12 ± 0.03	3.05	3.08	3.08	3.07 ± 0.02
4	10% D_2O/H_2O pH 4	3.96	4.04	3.94	3.98 ± 0.05	3.92	4.05	3.91	3.96 ± 0.08	4.40	4.09	4.08	4.19 ± 0.18
	sodium phosphate ($H_2PO_4^-$ /H_3PO_4) pH 4	3.97	4.01	4.01	4.00 ± 0.02	4.06	4.06	4.07	4.06 ± 0.01	4.04	4.04	4.04	4.04 ± 0.00
	acetic acid – sodium acetate (OAc^-/HOAc) pH 4	4.02	4.07	4.02	4.04 ± 0.03	4.05	4.10	4.05	4.07 ± 0.03	4.05	4.07	4.03	4.05 ± 0.02
Moderately acidic environment 5 ≤ pH ≤ 6													
5	10% D_2O/H_2O pH 5	5.02	5.03	5.00	5.02 ± 0.02	4.99	4.96	4.97	4.97 ± 0.02	4.66	4.51	4.68	4.62 ± 0.09
	sodium phosphate ($H_2PO_4^-$ /H_3PO_4) pH 5	4.91	5.02	5.05	4.99 ± 0.07	4.85	4.97	4.91	4.91 ± 0.06	4.76	4.79	4.79	4.78 ± 0.02
	acetic acid – sodium acetate	4.98	4.99	5.00	4.99 ± 0.01	4.95	4.97	4.97	4.96 ± 0.01	4.91	4.97	4.95	4.94 ± 0.03

	(OAc^-/HOAc) pH 5												
5.5	acetic acid – sodium acetate (OAc^-/HOAc) pH 5.5	5.47	5.49	5.50	5.49 ± 0.02	5.45	5.45	5.43	5.44 ± 0.01	5.47	5.42	5.41	5.43 ± 0.03
6	10% D_2O/H_2O pH 6	6.07	6.07	6.05	6.06 ± 0.01	6.00	5.97	6.01	5.99 ± 0.02	5.38	5.57	5.40	5.45 ± 0.10
	sodium phosphate ($H_2PO_4^-$/H_3PO_4) pH 6	6.00	6.01	6.00	6.00 ± 0.01	5.82	5.89	5.89	5.87 ± 0.04	5.85	5.93	5.90	5.89 ± 0.04
Neutral to moderately alkaline environment 7 ≤ pH ≤ 8													
7	10% D_2O/H_2O pH 7	7.07	7.10	7.05	7.07 ± 0.03	6.89	6.92	6.89	6.90 ± 0.02	6.12	6.10	6.08	6.10 ± 0.02
	sodium phosphate ($H_2PO_4^-$/H_3PO_4) pH 7	6.89	6.90	6.90	6.90 ± 0.01	6.75	6.80	6.80	6.78 ± 0.03	6.50	6.53	6.52	6.52 ± 0.02
	HEPES[c] pH 7	7.00	7.00	6.98	6.99 ± 0.01	7.00	6.99	6.98	6.99 ± 0.01	6.88	6.90	6.88	6.89 ± 0.01
	Tris[d]-HCl pH 7	6.95	6.99	6.95	6.96 ± 0.02	5.46	5.47	5.47	5.47 ± 0.01	5.57	5.57	5.58	5.57 ± 0.01
8	10% D_2O/H_2O pH 8	7.97	7.99	8.02	7.99 ± 0.03	6.36	6.37	6.44	6.39 ± 0.04	5.73	5.77	5.74	5.75 ± 0.02
	Sodium phosphate ($H_2PO_4^-$/H_3PO_4) pH 8	7.95	7.95	7.93	7.94 ± 0.01	7.77	7.80	7.74	7.77 ± 0.03	7.45	7.45	7.38	7.43 ± 0.04
	HEPES[c] pH 8	7.99	7.99	8.03	8.00 ± 0.02	7.62	7.62	7.63	7.62 ± 0.01	7.53	7.54	7.60	7.56 ± 0.04
	Tris[d]-HCl pH 8	7.95	7.95	7.95	7.95 ± 0.00	7.95	7.95	7.89	7.93 ± 0.03	7.20	7.20	7.18	7.19 ± 0.01

[a]SD – standard deviation; [b]RT- room temperature; [c]HEPES – 4-(2-hydroxyethyl)-1-piperazineethanesulfonic acid, $C_8H_{18}N_2O_4S$; [d]tris – tris(hydroxymethyl)aminomethane, $C_4H_{11}NO_3$.

5.2. ^{51}V spectroscopic studies of {$Mo_{72}V_{30}$} solutions

All ^{51}V NMR peaks were assigned based on the literature data from **Table S5**. In some ^{51}V spectra, several chemical shifts have not yet been described in the literature and, therefore, cannot be assigned in this work.

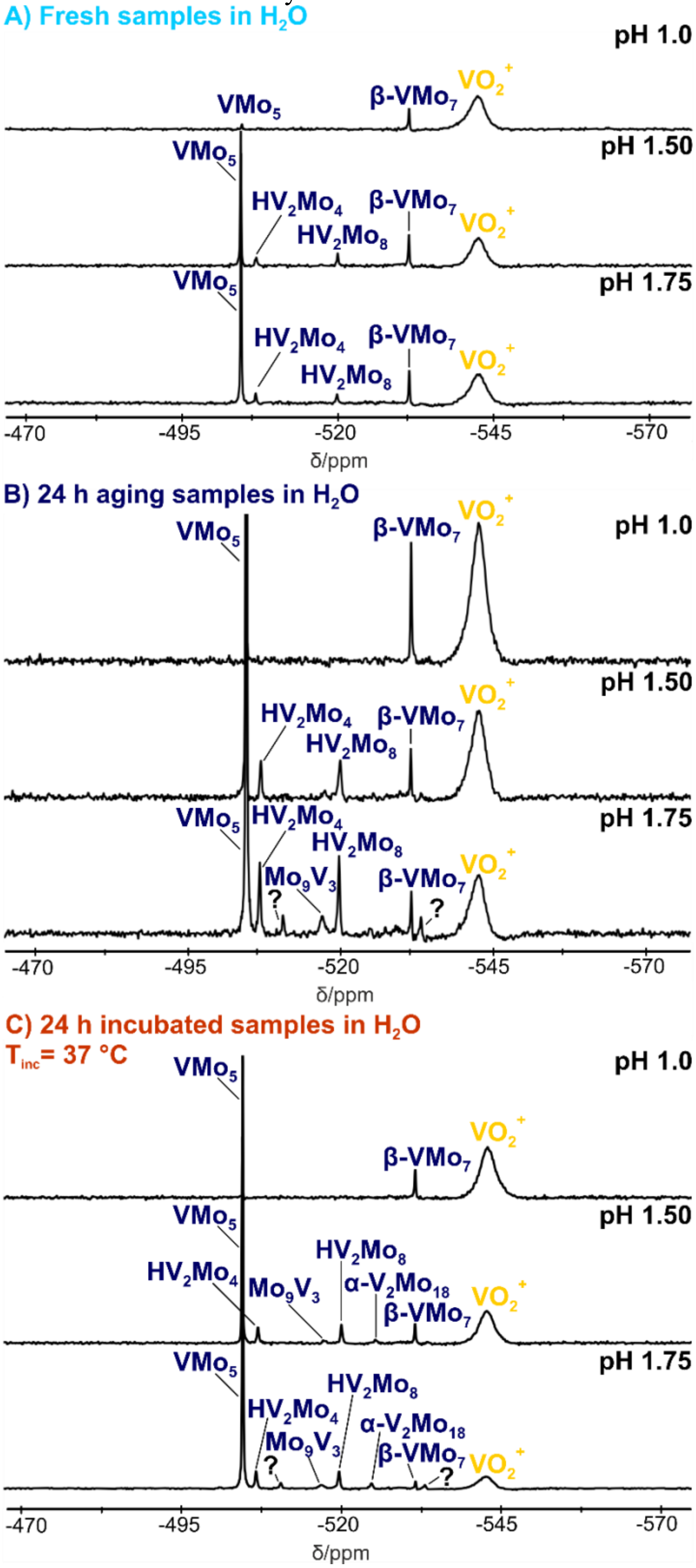

Fig. S8. ^{51}V NMR spectra of {$Mo_{72}V_{30}$} in H_2O (pH 1.0, pH 1.50, and pH 1.75): A) fresh solutions; B) 24 h aging at room temperature, and C) 24 h incubation at 37 °C.

^{51}V NMR spectra for 0.15 mM fresh solutions (**A**) of {$\mathbf{Mo_{72}V_{30}}$} in H_2O (pH 1.0 – 1.75) that were recorded approximately one hour after preparation. Aliquots of fresh solutions were taken for 24 h aging experiments at room temperature (**B**) and 24 h incubation at 37 °C (**C**) and were then recorded approximately 1 h after the end of 24 h experiments. The structures of all POMs forming during the experiments are shown in **Figures S1 – S2**. The chemical shifts and percentages of POM species are given in **Tables S8 – S10**.

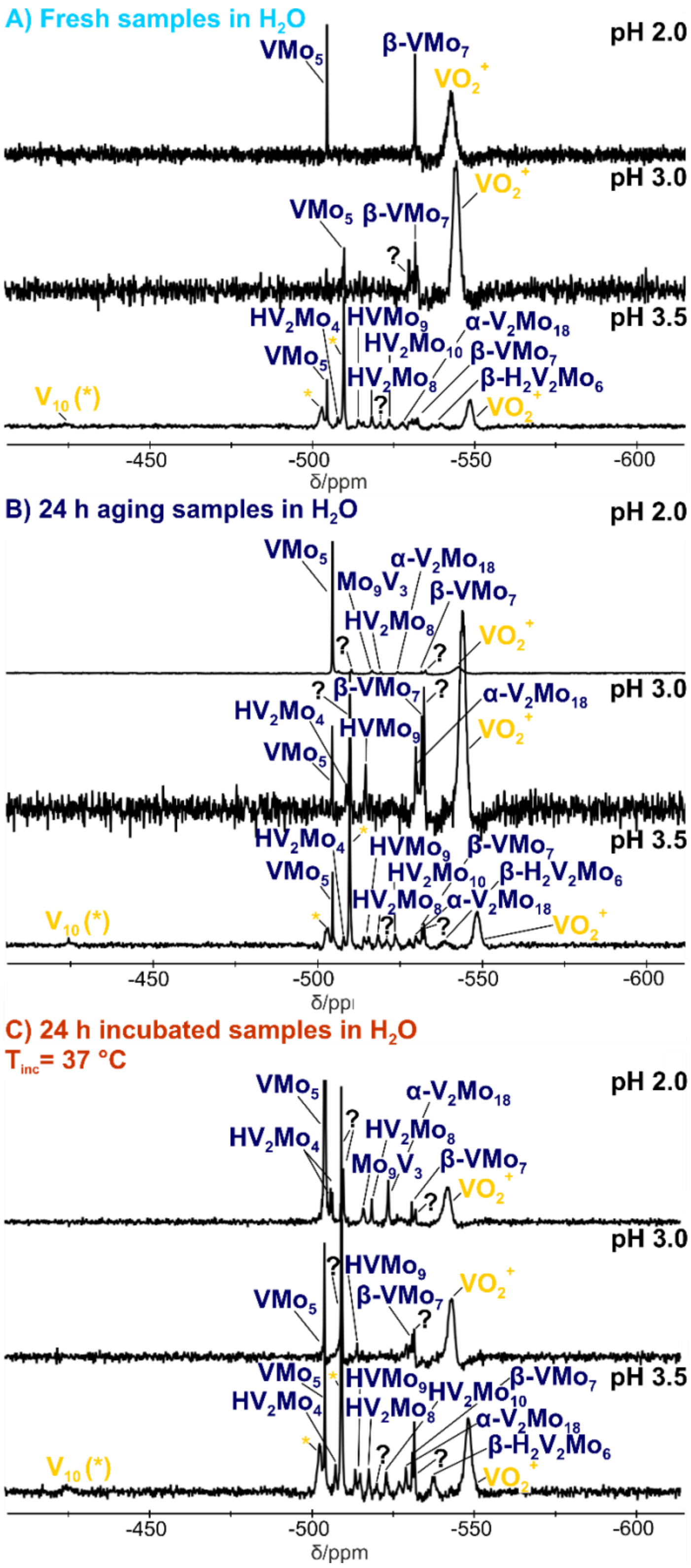

Fig. S9. ^{51}V NMR spectra of {Mo$_{72}$V$_{30}$} in H_2O (pH 2.0, pH 3.0, and pH 3.5): A) fresh solutions; B) 24 h aging at room temperature, and C) 24 h incubation at 37 °C.

^{51}V NMR spectra for 0.15 mM fresh solutions (**A**) of {**Mo$_{72}$V$_{30}$**} in H_2O (pH 2.0 – 3.5) that were recorded approximately one hour after preparation. Aliquots of fresh solutions were taken for 24 h aging experiments at room temperature (**B**) and 24 h incubation at 37 °C (**C**) and were then recorded approximately 1 h after the end of 24 h experiments. The structures of all POMs forming during the experiments are shown in **Figures S1 – S2**. The chemical shifts and percentages of POM species are given in **Tables S8 – S10**.

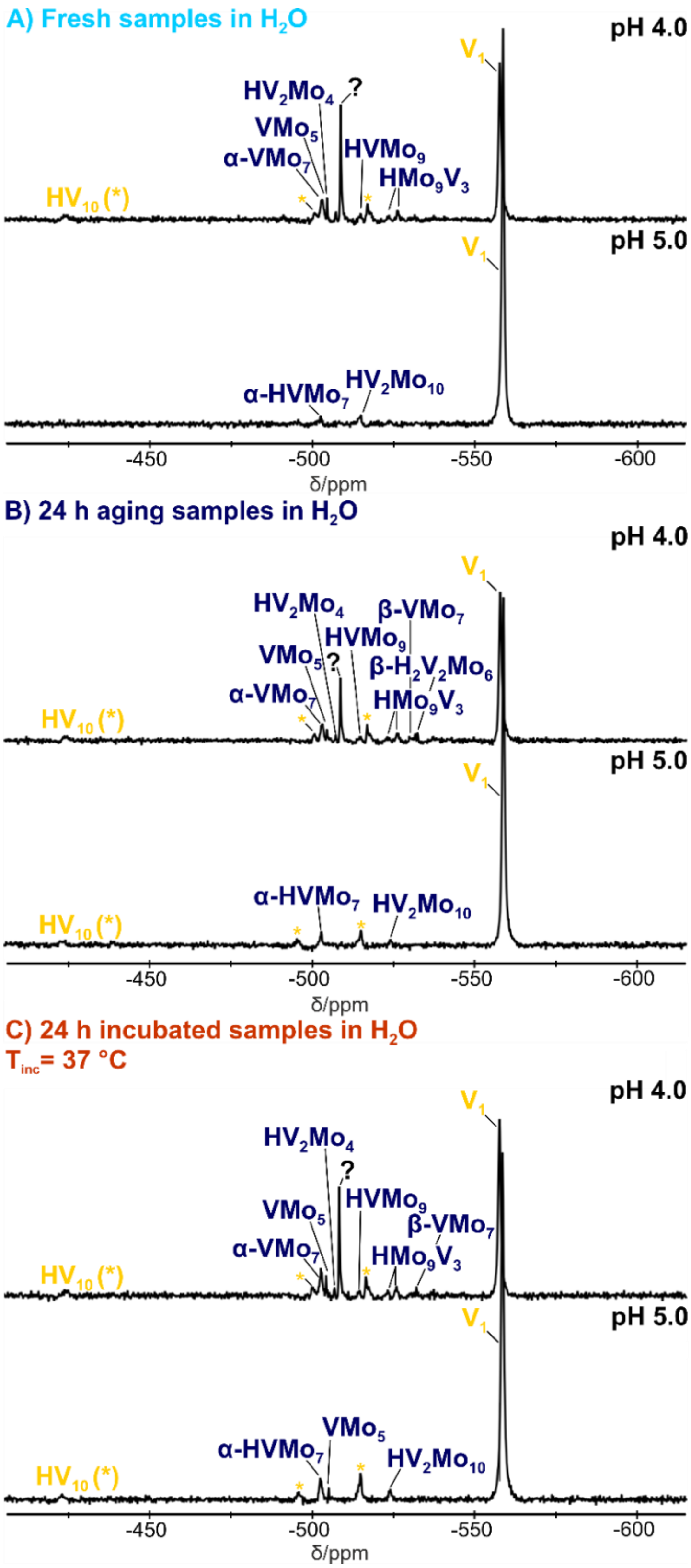

Fig. S10. ^{51}**V NMR spectra of {$Mo_{72}V_{30}$} in H_2O (pH 4.0, and pH 5.0): A) fresh solutions; B) 24 h aging at room temperature, and C) 24 h incubation at 37 °C.**

^{51}V NMR spectra for 0.15 mM fresh solutions (**A**) of {**$Mo_{72}V_{30}$**} in H_2O (pH 4.0 – 5.0) that were recorded approximately one hour after preparation. Aliquots of fresh solutions were taken for 24 h aging experiments at room temperature (**B**) and 24 h incubation at 37 °C (**C**) and were then recorded approximately 1 h after the end of 24 h experiments. The structures of all POMs forming during the experiments are shown in **Figures S1 – S2**. The chemical shifts and percentages of parent (if still present) and formed POM species are given in **Tables S8 – S10**.

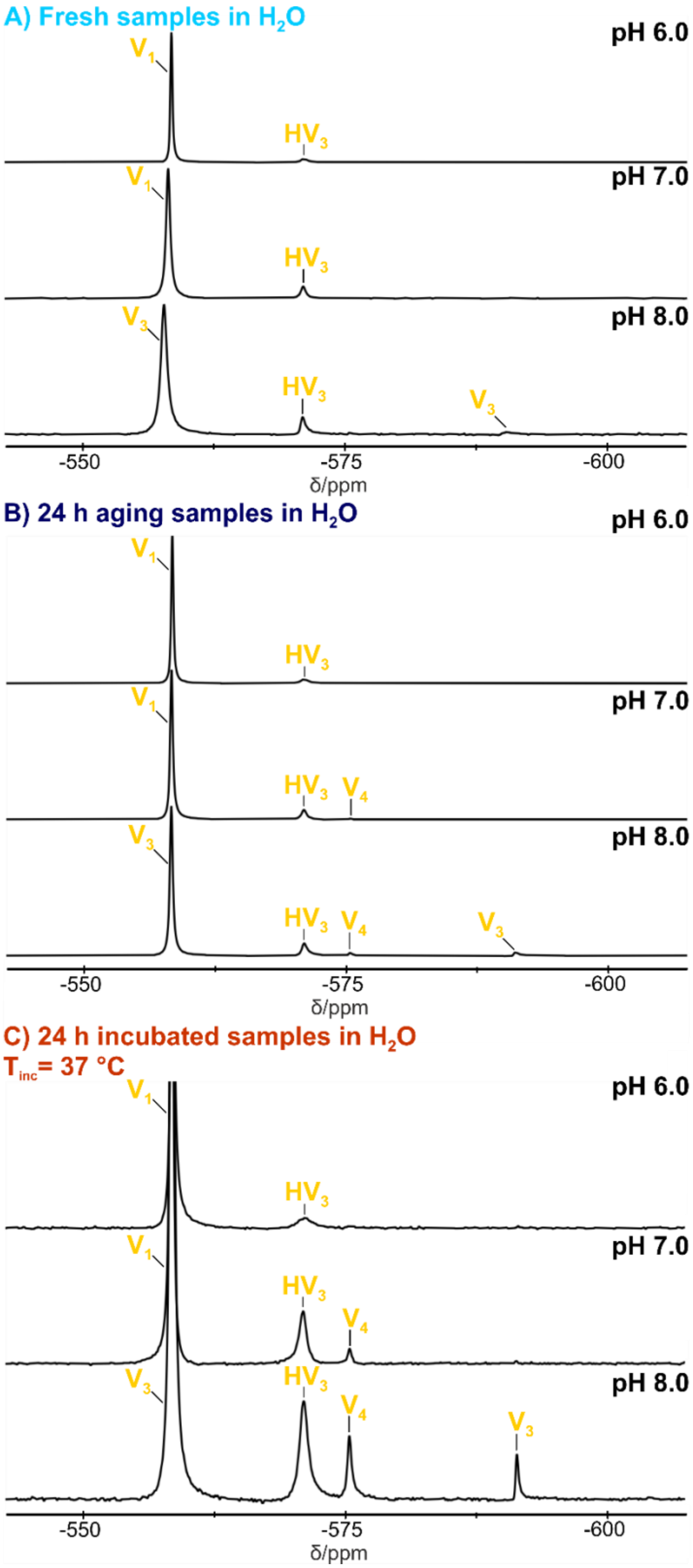

Fig. S11. ^{51}**V NMR spectra of {Mo$_{72}$V$_{30}$} in H$_2$O (pH 6.0, pH 7.0, and pH 8.0): A) fresh solutions; B) 24 h aging at room temperature, and C) 24 h incubation at 37 °C.**

^{51}V NMR spectra for 0.15 mM fresh solutions (**A**) of {**Mo$_{72}$V$_{30}$**} in H$_2$O (pH 6.0 – 8.0) that were recorded approximately one hour after preparation. Aliquots of fresh solutions were taken for 24 h aging experiments at room temperature (**B**) and 24 h incubation at 37 °C (**C**) and were then recorded approximately 1 h after the end of 24 h experiments. The structures of all POMs forming during the experiments are shown in **Figures S1 – S2**. The chemical shifts and percentages of parent (if still present) and formed POM species are given in **Tables S8 – S10**.

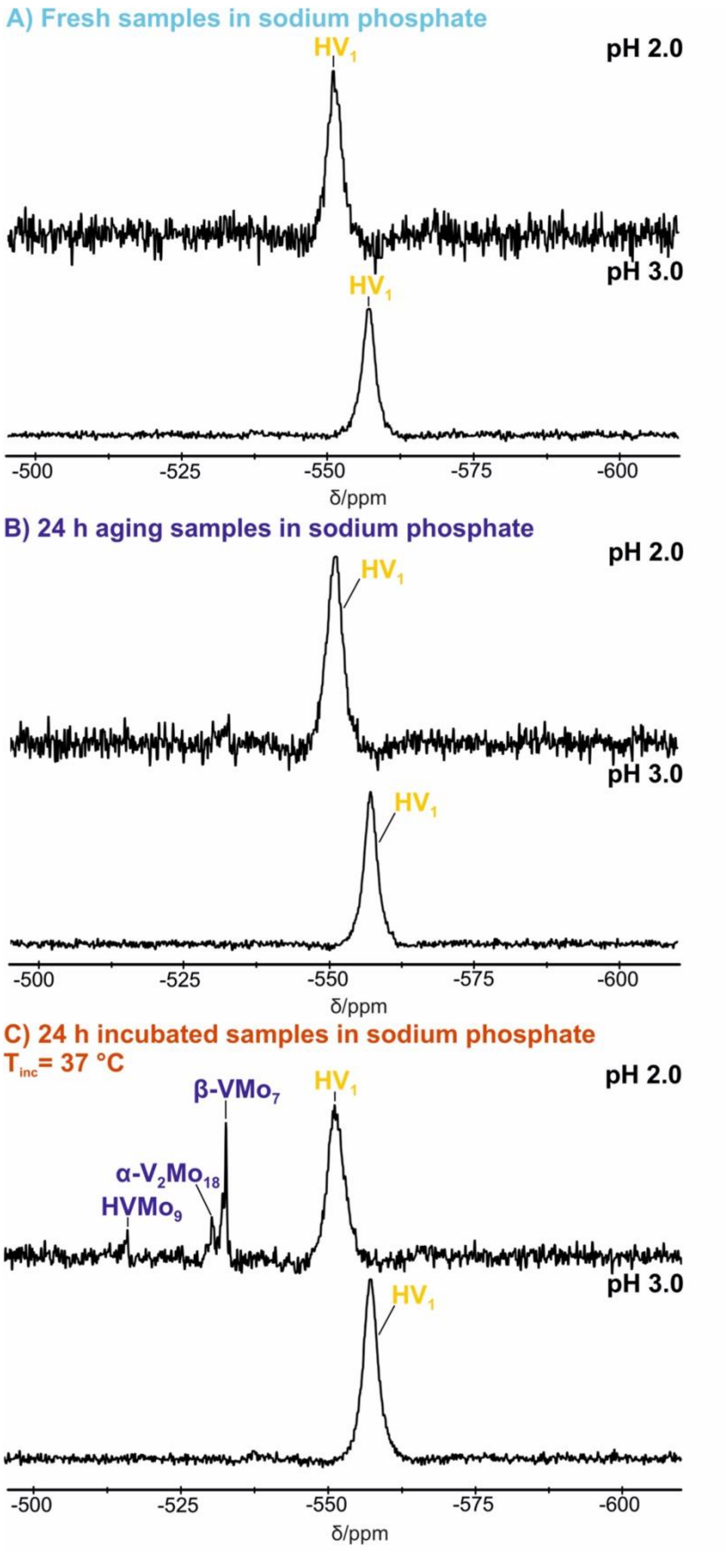

Fig. S12. ^{51}**V NMR spectra of {Mo$_{72}$V$_{30}$} in 0.1 M sodium phosphate buffers (pH 2.0 and pH 3.0): A) fresh solutions; B) 24 h aging at room temperature, and C) 24 h incubation at 37 °C.** ^{51}V NMR spectra for 0.15 mM fresh solutions (**A**) of **{Mo$_{72}$V$_{30}$}** in 0.1 M sodium phosphate buffers (pH 2.0 – 3.0) that were recorded approximately one hour after preparation. Aliquots of fresh solutions were taken for 24 h aging experiments at room temperature (**B**) and 24 h incubation at 37 °C (**C**) and were then recorded approximately 1 h after the end of 24 h experiments. The structures of all POMs forming during the experiments are shown in **Figures S1 – S2**. The chemical shifts and percentages of parent (if still present) and formed POM species are given in **Tables S8 – S10**.

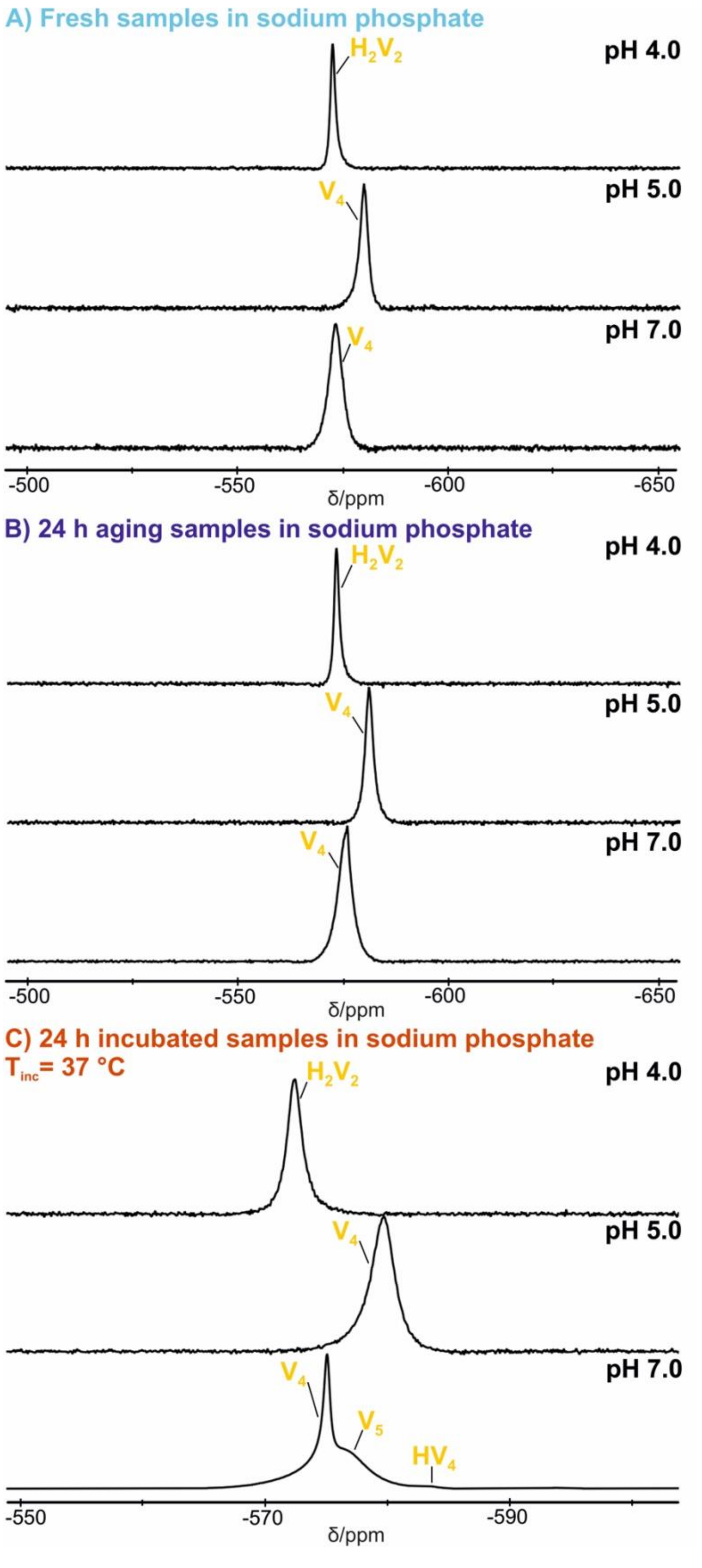

Fig. S13. ^{51}V NMR spectra of {$Mo_{72}V_{30}$} in 0.1 M sodium phosphate buffers (pH 4.0, pH 5.0, and pH 7.0): A) fresh solutions; B) 24 h aging at room temperature, and C) 24 h incubation at 37 °C.

^{51}V NMR spectra for 0.15 mM fresh solutions (**A**) of {$\mathbf{Mo_{72}V_{30}}$} in 0.1 M sodium phosphate buffers (pH 4.0, 5.0 and 7.0) that were recorded approximately one hour after preparation. Aliquots of fresh solutions were taken for 24 h aging experiments at room temperature (**B**) and 24 h incubation at 37 °C (**C**) and were then recorded approximately 1 h after the end of 24 h experiments. The structures of all POMs forming during the experiments are shown in **Figures S1 – S2**. The chemical shifts and percentages of parent (if still present) and formed POM species are given in **Tables S8 – S10**.

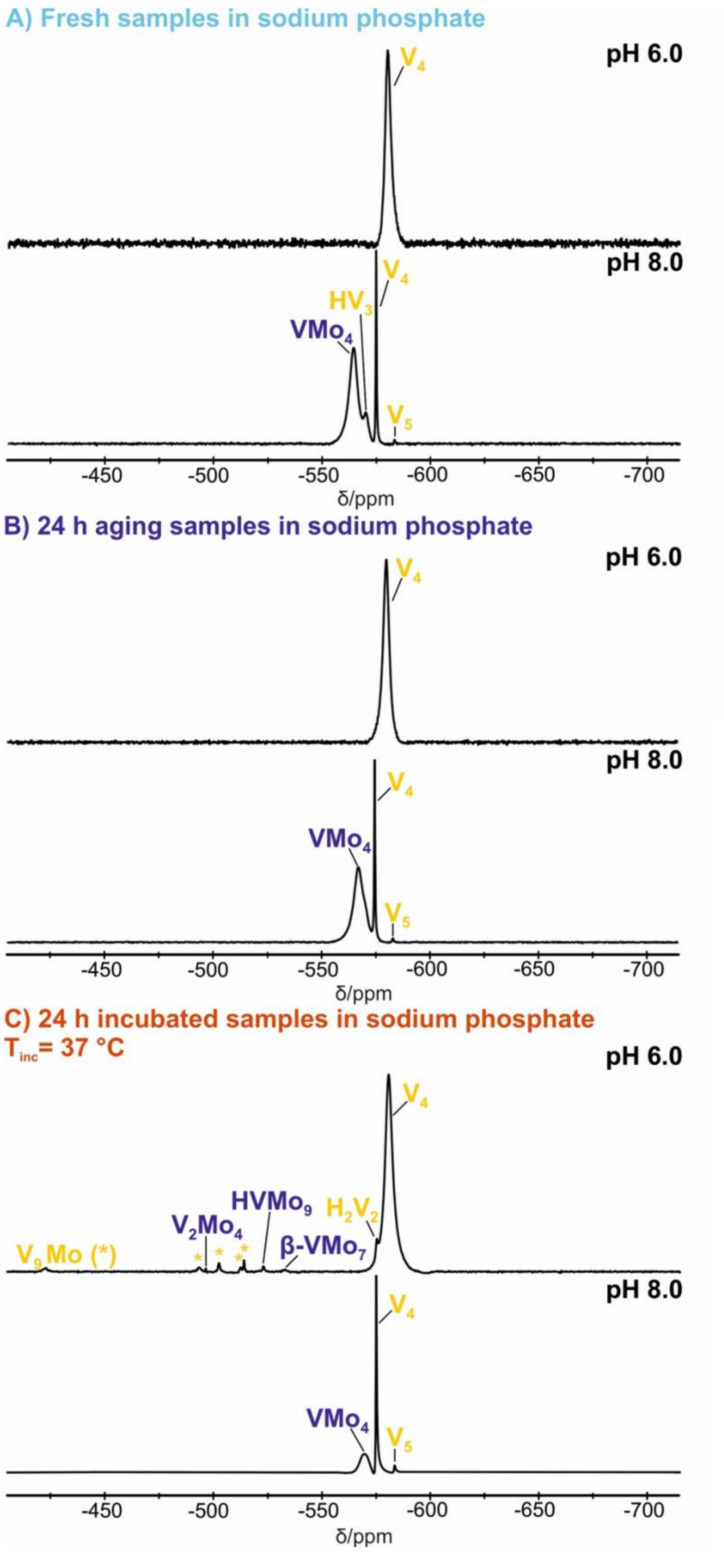

Fig. S14. ^{51}**V NMR spectra of {**$Mo_{72}V_{30}$**} in 0.1 M sodium phosphate buffers (pH 6.0 and pH 8.0): A) fresh solutions; B) 24 h aging at room temperature, and C) 24 h incubation at 37 °C.**

^{51}V NMR spectra for 0.15 mM fresh solutions (**A**) of **{**$Mo_{72}V_{30}$**}** in 0.1 M sodium phosphate buffers (pH 6.0 and 8.0) that were recorded approximately one hour after preparation. Aliquots of fresh solutions were taken for 24 h aging experiments at room temperature (**B**) and 24 h incubation at 37 °C (**C**) and were then recorded approximately 1 h after the end of 24 h experiments. The structures of all POMs forming during the experiments are shown in **Figures S1 – S2**. The chemical shifts and percentages of parent (if still present) and formed POM species are given in **Tables S8 – S10**.

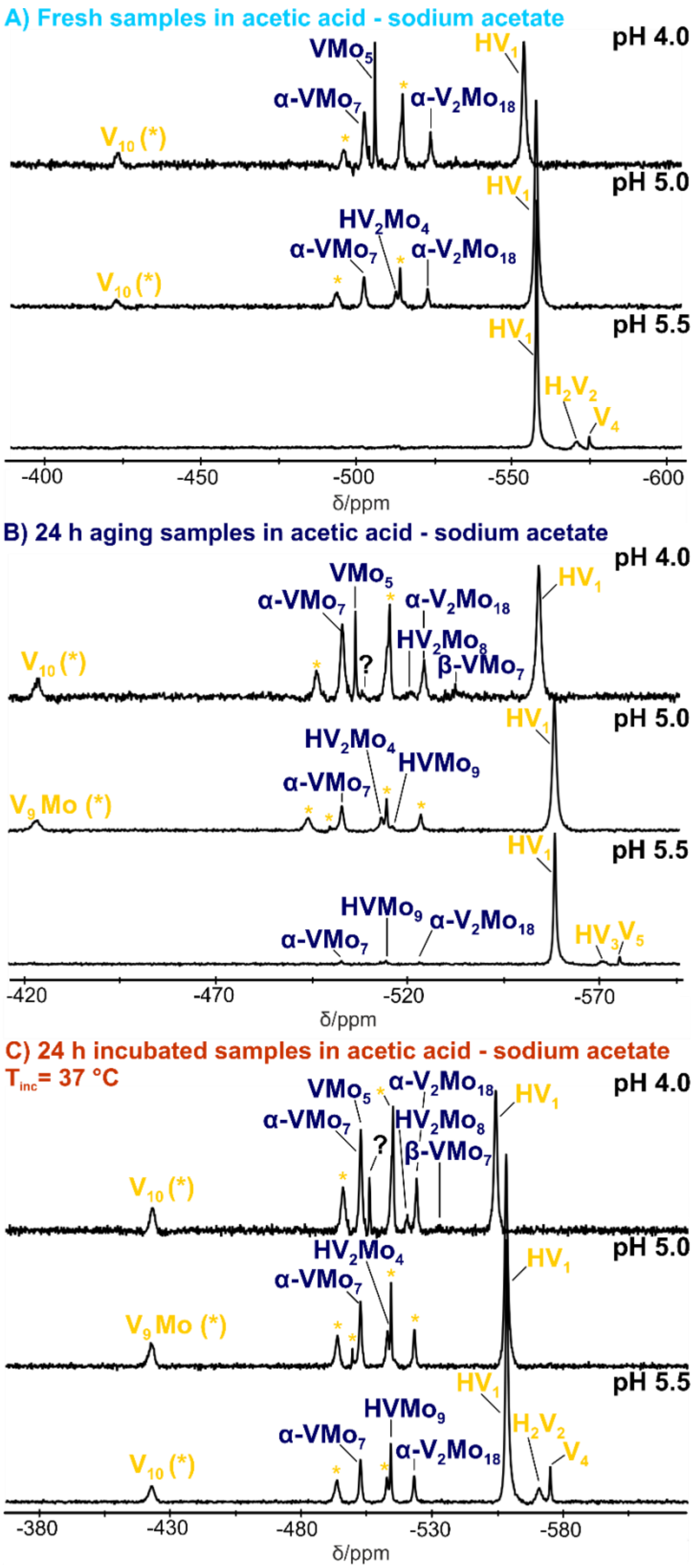

Fig. S15. [51]V NMR spectra of {Mo72V30} in 0.1 M acetic acid – sodium acetate buffers: A) fresh solutions; B) 24 h aging at room temperature and C) 24 h incubation at 37 °C.

[51]V NMR spectra for 0.15 mM fresh solutions (**A**) of {**Mo72V30**} in 0.1 M acetic acid – sodium acetate buffers (pH 4 – 5.5) that were recorded approximately one hour after preparation. Aliquots of fresh solutions were taken for 24 h aging experiments at room temperature (**B**) and 24 h incubation at 37 °C (**C**) and were then recorded approximately 1 h after the end of 24 h experiments. The structures of all POMs forming during the experiments are shown in **Figures S1 – S2**. The chemical shifts and percentages of parent (if still present) and formed POM species are given in **Tables S8 – S10**.

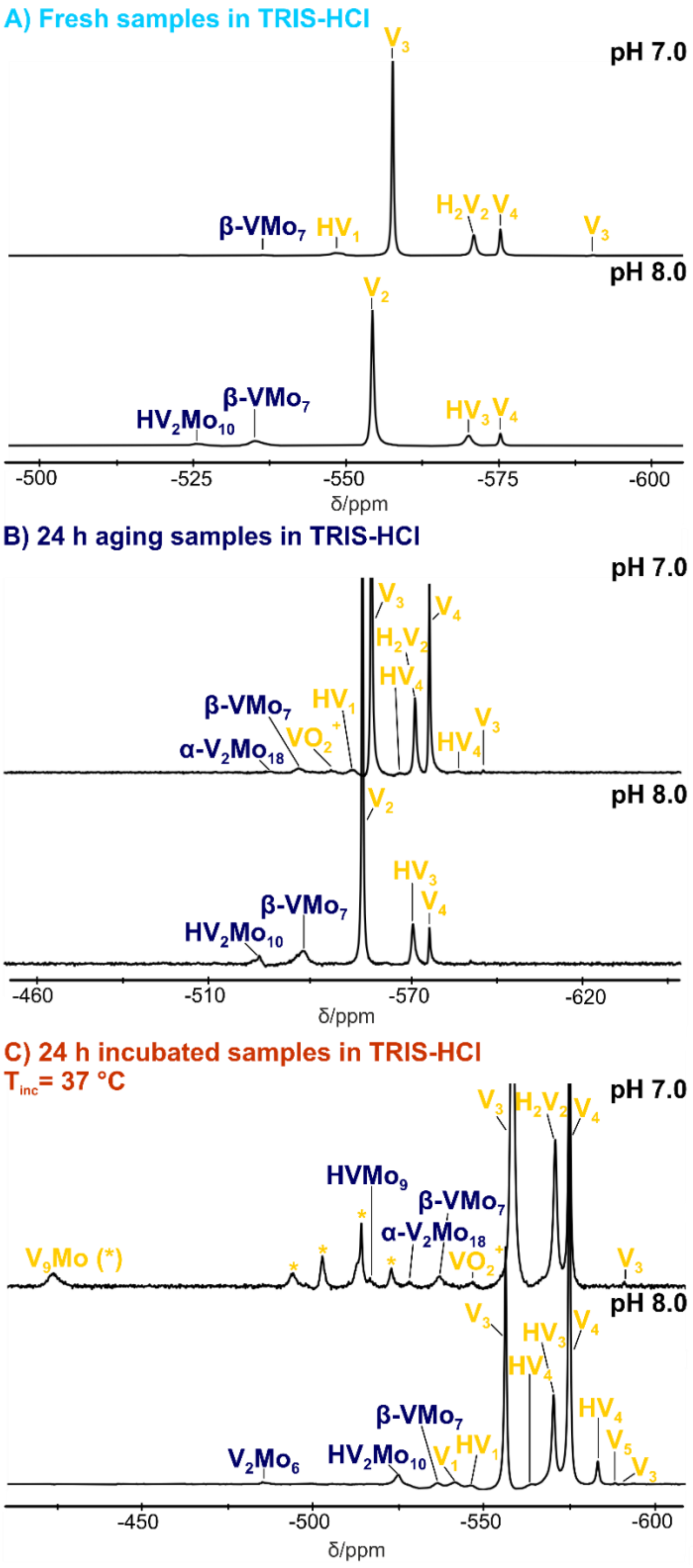

Fig. S16. ^{51}V NMR spectra of $\{Mo_{72}V_{30}\}$ in 0.1 M Tris - HCl buffers (pH 7.0 – 8.0): A) fresh solutions; B) 24 h aging at room temperature and C) 24 h incubation at 37 °C.

^{51}V NMR spectra for 0.15 mM fresh solutions (**A**) of **$\{Mo_{72}V_{30}\}$** in 0.1 M Tris - HCl buffers (pH 7.0 – 8.0) that were recorded approximately one hour after preparation. Aliquots of fresh solutions were taken for 24 h aging experiments at room temperature (**B**) and 24 h incubation at 37 °C (**C**) and were then recorded approximately 1 h after the end of 24 h experiments. The structures of all POMs forming during the experiments are shown in **Figures S1 – S2**. The chemical shifts and percentages of parent (if still present) and formed POM species are given in **Tables S8 – S10**.

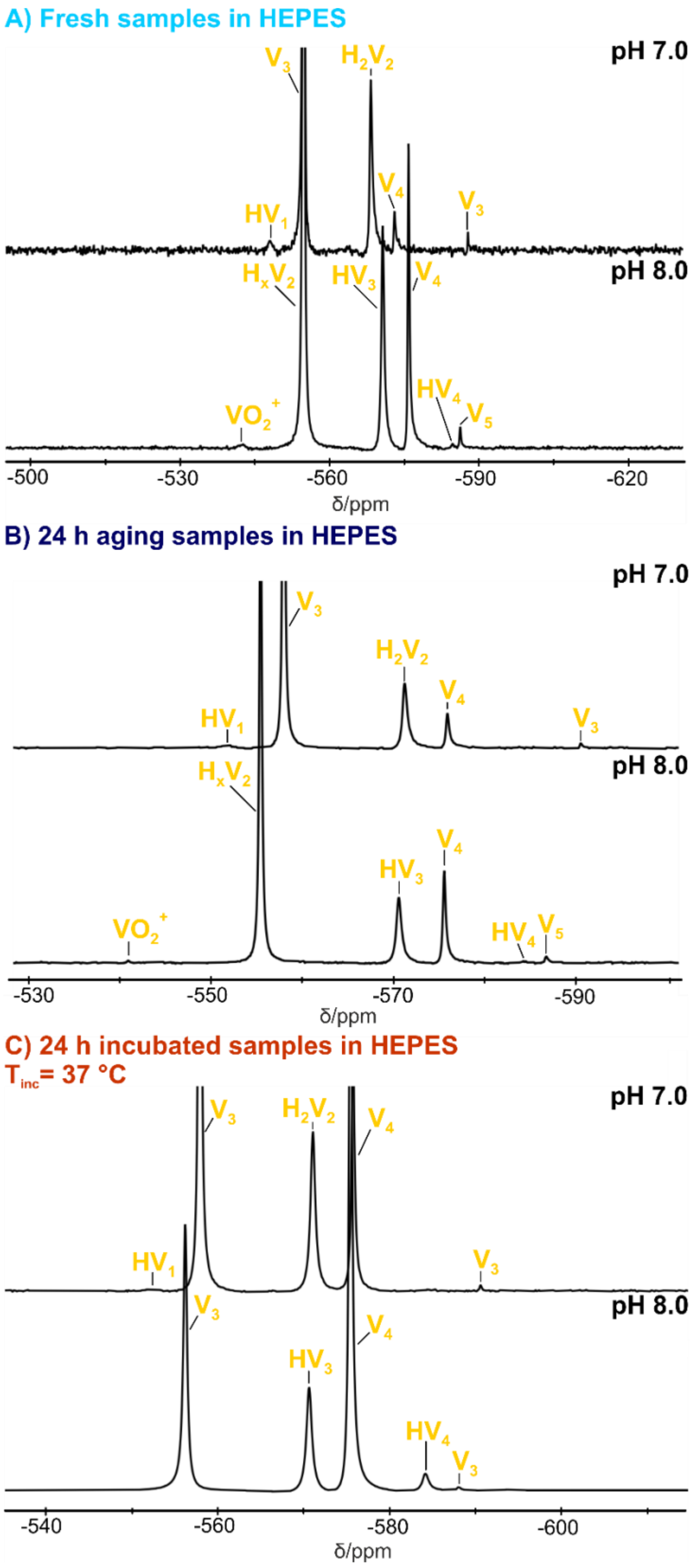

Fig. S17. ^{51}V NMR spectra of {$Mo_{72}V_{30}$} in 0.1 M HEPES buffers (pH 7.0 – 8.0): A) fresh solutions; B) 24 h aging at room temperature and C) 24 h incubation at 37 °C.

^{51}V NMR spectra for 0.15 mM fresh solutions (**A**) of {**$Mo_{72}V_{30}$**} in 0.1 M HEPES buffers (pH 7 – 8) that were recorded approximately one hour after preparation. Aliquots of fresh solutions were taken for 24 h aging experiments at room temperature (**B**) and 24 h incubation at 37 °C (**C**) and were then recorded approximately 1 h after the end of 24 h experiments. The structures of all POMs forming during the experiments are shown in **Figures S1 – S2**. The chemical shifts and percentages of parent (if still present) and formed POM species are given in **Tables S8 – S10**.

Table S8. Analysis of NMR spectroscopic data recorded in {$Mo_{72}V_{30}$} in fresh solutions.

Chemical shifts in ^{51}V NMR spectra measured in triplicate of **{$Mo_{72}V_{30}$}** (0.15 mM) dissolved in D_2O and 0.1 M buffers (acetic acid – sodium acetate pH 4 – 5.5; sodium phosphate pH 2 – 8 (while phosphate does not buffer at pH range from 3.5 – 5.5, experiments were conducted at this pH to provide comparisons to previously published studies (*239*); Tris-HCl pH 7 – 8; HEPES pH 7 – 8). The content of species was calculated based on the integration of ^{51}V signals. Signals were assigned based on the literature data summarized in **Table S5**. Percentages are normalized within each pH row and sum to ~100 % (small deviations reflect rounding or trace unassigned signals).

**pH**	**{$Mo_{72}V_{30}$} (0.15 mM) in Solvent / Buffer**	**% of all mixed V-Mo small POMs (Tables S11 – S14) or/and *unassigned* species in fresh solution**				**% of all iso(poly)vanadate species (Tables S11 – S14) in fresh solution**			
		**Sample**			**Mean of 1 to 3 ± SD[a]**	**Sample**			**Mean of 1 to 3 ± SD[a]**
		**#1**	**#2**	**#3**		**#1**	**#2**	**#3**	
1	10% D_2O/H_2O pH 1	−δ (^{51}V) [ppm]: 504.7, 531.6				−δ (^{51}V) [ppm]: 542.7			
		6.8	6.7	6.5	6.7 ± 0.2	93.2	93.3	93.5	93.3 ± 0.2
1.5	10% D_2O/H_2O pH 1.5	−δ (^{51}V) [ppm]: 504.6, 507.1, 520.2, 531.6				−δ (^{51}V) [ppm]: 542.8			
		29.2	31.6	33.3	31.4 ± 2.1	70.7	68.5	66.7	68.6 ± 2.1
1.75	10% D_2O/H_2O pH 1.75	−δ (^{51}V) [ppm]: 504.6, 506.9, 520.0, 531.6				−δ (^{51}V) [ppm]: 542.6			
		38.0	39.5	42.9	40.1 ± 2.5	62.0	60.5	57.1	59.9 ± 2.5
2	10% D_2O/H_2O pH 2	−δ (^{51}V) [ppm]: 504.5, 531.5				−δ (^{51}V) [ppm]: 542.6			
		41.2	33.5	45.8	40.2 ± 6.2	58.8	66.5	54.2	59.8 ± 6.2
	0.1 M sodium phosphate ($H_2PO_4^-$/H_3PO_4) pH 2	−δ (^{51}V) [ppm]: –				−δ (^{51}V) [ppm]: 551.2			
		–	–	–	–	100	100	100	100 ± 0.0
3	10% D_2O/H_2O pH 3	−δ (^{51}V) [ppm]: 504.4, 531.6				−δ (^{51}V) [ppm]: 544.1			
		7.5	10.0	6.0	7.8 ± 2.0	90.6	87.5	91.5	89.9 ± 2.1
		unassigned −δ (^{51}V) [ppm]: 509.8							
		2.0	2.5	2.5	2.3 ± 0.3				
	0.1 M sodium phosphate ($H_2PO_4^-$/H_3PO_4) pH 3	−δ (^{51}V) [ppm]: –				−δ (^{51}V) [ppm]: 556.9			
		–	–	–	–	100	100	100	100 ± 0.0
3.5	10% D_2O/H_2O pH 3.5	−δ (^{51}V) [ppm]: 504.5, 507.8, 514.1, 518.2, 523.5, 527.7, 531.2, 539.8				−δ (^{51}V) [ppm]: 423.7, 502.9, 509.7, 544.7			
		19.1	14.9	16.7	16.9 ± 2.1	80.9	85.1	83.3	83.1 ± 2.1
4	10% D_2O/H_2O pH 4	−δ (^{51}V) [ppm]: 502.9, 504.4, 507.5, 514.8, 523.6, 526.0,				−δ (^{51}V) [ppm]: 423.7, 500.8, 516.7, 557.6			
		17.5	17.6	15.1	16.7 ± 1.4	68.5	68.9	70.9	69.4 ± 1.3
		unassigned −δ (^{51}V) [ppm]: 508.5							
		14.0	13.5	14.1	13.9 ± 0.3				

	0.1 M sodium phosphate ($H_2PO_4^-$/H_3PO_4) pH 4	−δ (^{51}V) [ppm]: −				−δ (^{51}V) [ppm]: 572.4			
		−	−	−	−	100	100	100	
	0.1 M acetic acid – sodium acetate (OAc^-/HOAc) pH 4	−δ (^{51}V) [ppm]: 502.9, 504.5, 506.3, 524.3				−δ (^{51}V) [ppm]: 423.1, 496.2, 515.2, 554.2			
		30.1	29.9	27.8	29.3 ± 1.3	69.5	69.5	72.1	70.7 ± 1.2
5	10% D_2O/H_2O pH 5	−δ (^{51}V) [ppm]: 502.5, 523.8				−δ (^{51}V) [ppm]: 514.8, 558.6			
		2.0	2.0	2.0	2.0 ± 0	97.5	97.0	98.0	97.5 ± 0.5
	0.1 M sodium phosphate ($H_2PO_4^-$/H_3PO_4) pH 5	−δ (^{51}V) [ppm]: −				−δ (^{51}V) [ppm]: 580.2			
		−	−	−	−	100	100	100	100 ± 0.0
	0.1 M acetic acid – sodium acetate (OAc^-/HOAc) pH 5	−δ (^{51}V) [ppm]: 502.8, 513.2, 523.3				−δ (^{51}V) [ppm]: 423.5, 494.1, 514.4, 558.2			
		14.3	16.1	16.3	15.6 ± 1.1	86.1	86.4	83.7	84.4 ± 1.1
5.5	0.1 M acetic acid – sodium acetate (OAc^-/HOAc) pH 5.5	−δ (^{51}V) [ppm]: −				−δ (^{51}V) [ppm]: 559.3, 571.1, 575.2			
		−	−	−	−	100	100	100	100 ± 0.0
6	10% D_2O/H_2O pH 6	−δ (^{51}V) [ppm]: −				−δ (^{51}V) [ppm]: 558.4, 571.1			
		−	−	−	−	100	100	100	100 ± 0.0
	0.1 M sodium phosphate (HPO_4^{2-}/$H_2PO_4^-$) pH 6	−δ (^{51}V) [ppm]: −				−δ (^{51}V) [ppm]: 580.2			
		−	−	−	−	100	100	100	100 ± 0.0
7	10% D_2O/H_2O pH 7	−δ (^{51}V) [ppm]: −				−δ (^{51}V) [ppm]: 558.3, 571.1			
		−	−	−	−	100	100	100	100 ± 0.0
	0.1 M sodium phosphate (HPO_4^{2-}/$H_2PO_4^-$) pH 7	−δ (^{51}V) [ppm]: −				−δ (^{51}V) [ppm]: 573.3			
		−	−	−	−	100	100	100	100 ± 0.0
	0.1 M HEPES[c] pH 7	−δ (^{51}V) [ppm]: −				−δ (^{51}V) [ppm]: 551.3, 557.8, 571.2, 575.9, 590.5			
		−	−	−	−	100	100	100	100 ± 0.0
	0.1 M Tris[d]-HCl pH 7	−δ (^{51}V) [ppm]: 536.8				−δ (^{51}V) [ppm]: 548.5, 557.7, 571.0, 575.4			
		0.8	0.5	0.7	0.7 ± 0.2	97.8	98.0	99.3	99.3 ± 0.2
8	10% D_2O/H_2O pH 8	−δ (^{51}V) [ppm]: −				−δ (^{51}V) [ppm]: 557.8, 570.9, 590.3			
		−	−	−	−	100	100	100	100 ± 0.0
	0.1 M sodium phosphate (HPO_4^{2-}/$H_2PO_4^-$) pH 8	−δ (^{51}V) [ppm]: −				−δ (^{51}V) [ppm]: 564.7, 570.5, 575.0, 583.6			
		−	−	−	−	100	100	100	100 ± 0.0
	0.1 M HEPES[c] pH 8	−δ (^{51}V) [ppm]: −				−δ (^{51}V) [ppm]: 542.7, 554.5, 570.2, 575.4, 584.2, 585.7			
		−	−	−	−	100	100	100	100 ± 0.0

	0.1 M Tris[d]-HCl pH 8	−δ (^{51}V) [ppm]: 525.6, 535.3				−δ (^{51}V) [ppm]: 545.9, 554.5, 570.3, 575.4, 582.9			
		12.7	12.0	10.4	11.7 ± 1.2	87.3	88.0	89.6	88.3 ± 1.2

[a]SD – standard deviation; [b]RT- room temperature; [c]HEPES – 4-(2-hydroxyethyl)-1-piperazineethanesulfonic acid, $C_8H_{18}N_2O_4S$; [d]tris – tris(hydroxymethyl)aminomethane, $C_4H_{11}NO_3$.Iso(poly)vanadates include V_{10} and V_9Mo_1 decavanadate species.

Table S9. Analysis of NMR spectroscopic data recorded in {$Mo_{72}V_{30}$} solutions after 24 h aging at room temperature.

Chemical shifts in ^{51}V NMR spectra measured in triplicate of **{$Mo_{72}V_{30}$}** (0.15 mM) dissolved in D_2O and 0.1 M buffers (acetic acid – sodium acetate pH 4 – 5.5; sodium phosphate pH 2 – 8 (while phosphate does not buffer at pH range from 3.5 – 5.5, experiments were conducted at this pH to provide comparisons to previously published studies (*239*); Tris-HCl pH 7 – 8; HEPES pH 7 – 8) and measured after 24 h kept at room temperature. The content of species was calculated based on the integration of ^{51}V signals. Signals were assigned based on the literature data summarized in **Table S5**. Percentages are normalized within each pH row and sum to ~100 % (small deviations reflect rounding or trace unassigned signals).

**pH**	**{$Mo_{72}V_{30}$} (0.15 mM) in solvent / buffer**	**% of all mixed V-Mo small POMs (Tables S11 – S14) or/and *unassigned* species in solution after 24 h at RT**				**% of all iso(poly)vanadate species (Tables S11 – S14) after 24 h at RT**			
		**Sample**			**Mean of 1 to 3 ± SD[a]**	**Sample**			**Mean of 1 to 3 ± SD[a]**
		**#1**	**#2**	**#3**		**#1**	**#2**	**#3**	
1	10% D_2O/H_2O pH 1	−δ (^{51}V) [ppm]: 504.7, 531.6				−δ (^{51}V) [ppm]: 542.7			
		8.3	6.4	9.2	8.0 ± 1.4	91.7	93.6	90.8	92.0 ± 1.4
1.5	10% D_2O/H_2O pH 1.5	−δ (^{51}V) [ppm]: 504.5, 507.0, 520.0, 531.5				−δ (^{51}V) [ppm]: 542.6			
		40.8	45	30.1	38.6 ± 6.9	59.2	55.0	69.9	61.4 ± 7.7
1.75	10% D_2O/H_2O pH 1.75	−δ (^{51}V) [ppm]: 504.6, 506.8, 517.0, 519.8, 531.6				−δ (^{51}V) [ppm]: 542.4			
		63.5	65.2	63.2	64.0 ± 1.1	34.2	32.8	35.0	34.0 ± 1.1
		unassigned −δ (^{51}V) [ppm]: 510.6, 533.2							
		2.3	2.0	1.8	2.0 ± 0.3				
2	10% D_2O/H_2O pH 2	−δ (^{51}V) [ppm]: 504.4, 506.3, 516.5, 519.2, 524.3, 531.5				−δ (^{51}V) [ppm]: 542.7			
		73.2	79.3	70.8	74.4 ± 4.4	22.6	15.8	24.9	21.1 ± 4.7
		unassigned −δ (^{51}V) [ppm]: 510.3, 532.8							
		4.3	4.8	4.3	4.5 ± 0.3				
	0.1 M sodium phosphate ($H_2PO_4^-$/H_3PO_4) pH 2	−δ (^{51}V) [ppm]: −				−δ (^{51}V) [ppm]: 551.0			
		−	−	−	−	100	100	100	100 ± 0.0
3	10% D_2O/H_2O pH 3	−δ (^{51}V) [ppm]: 504.4, 514.6, 529.8, 531.7				−δ (^{51}V) [ppm]: 544.1			
		9.5	9.0	4.0	7.5 ± 3.0	81.5	82.0	90.1	84.5 ± 4.8
		unassigned −δ (^{51}V) [ppm]: 509.8, 532.4							
		9.0	9.0	6.0	8.0 ± 1.8				
	0.1 M sodium phosphate ($H_2PO_4^-$/H_3PO_4) pH 3	−δ (^{51}V) [ppm]: −				−δ (^{51}V) [ppm]: 557.0			
		−	−	−	−	100	100	100	100 ± 0.0

3.5	10% D_2O/H_2O pH 3.5	−δ (^{51}V) [ppm]: 503.5, 504.5, 507.9, 515.4, 518.3, 523.5, 530.9, 538.9				−δ (^{51}V) [ppm]: 424.3, 509.7, 527.8, 544.8			
		20.7	19.9	20.8	20.5 ± 0.5	79.0	79.7	78.5	79.1 ± 0.6
		unassigned −δ (^{51}V) [ppm]: 520.9							
		0.3	0.3	0.7	0.4 ± 0.2				
4	10% D_2O/H_2O pH 4	−δ (^{51}V) [ppm]: 502.7, 504.4, 507.3, 514.7, 525.9, 532.0, 536.8				−δ (^{51}V) [ppm]: 424.3, 500.5, 516.7, 522.9, 557.6			
		17.0	15.2	14.3	15.5 ± 1.4	73.0	73.6	76.0	74.2 ± 1.6
		unassigned −δ (^{51}V) [ppm]: −δ (^{51}V) [ppm]: 508.5							
		10.0	11.2	9.7	10.3 ± 0.8				
	0.1 M sodium phosphate ($H_2PO_4^-$/H_3PO_4) pH 4	−δ (^{51}V) [ppm]: −				−δ (^{51}V) [ppm]: 572.4			
		−	−	−	−	100	100	100	100 ± 0.0
	0.1 M acetic acid – sodium acetate (OAc^-/HOAc) pH 4	−δ (^{51}V) [ppm]: 502.9, 504.5, 506.3, 524.4				−δ (^{51}V) [ppm]: 423.1, 496.2, 515.2, 554.3			
		30.5	30.5	34.7	31.9 ± 2.4	69.5	69.5	65.3	68.1 ± 2.4
5	10% D_2O/H_2O pH 5	−δ (^{51}V) [ppm]: 502.6, 524.0				−δ (^{51}V) [ppm]: 423.6, 495.5, 514.9, 558.5			
		2.5	3.0	3.0	2.8 ± 0.3	97.5	97.0	97.0	97.2 ± 0.3
	0.1 M sodium phosphate ($H_2PO_4^-$/H_3PO_4) pH 5	−δ (^{51}V) [ppm]: −				−δ (^{51}V) [ppm]: 580.2			
		−	−	−	−	100	100	100	100 ± 0.0
	0.1 M acetic acid – sodium acetate (OAc^-/HOAc) pH 5	−δ (^{51}V) [ppm]: 502.8, 513.1, 523.4				−δ (^{51}V) [ppm]: 423.0, 494.0, 499.5, 514.5, 558.4			
		13.9	13.6	15.0	14.2 ± 0.7	86.1	86.4	85.0	85.8 ± 0.7
5.5	0.1 M acetic acid – sodium acetate (OAc^-/HOAc) pH 5.5	−δ (^{51}V) [ppm]: 502.6, 512.8, 514.3				−δ (^{51}V) [ppm]: 558.3, 571.0, 575.2			
		4.5	5.0	4.0	4.5 ± 0.5	95.5	95.0	96.0	95.5 ± 0.5
6	10% D_2O/H_2O pH 6	−δ (^{51}V) [ppm]: −				−δ (^{51}V) [ppm]: 558.4, 571.1			
		−	−	−	−	100	100	100	100 ± 0.0
	0.1 M sodium phosphate (HPO_4^{2-}/$H_2PO_4^-$) pH 6	−δ (^{51}V) [ppm]: −				−δ (^{51}V) [ppm]: 580.4			
		−	−	−	−	100	100	100	100 ± 0.0
7	10% D_2O/H_2O pH 7	−δ (^{51}V) [ppm]: −				−δ (^{51}V) [ppm]: 558.3, 571.0			
		−	−	−	−	100	100	100	100 ± 0.0
	0.1 M sodium phosphate (HPO_4^{2-}/$H_2PO_4^-$) pH 7	−δ (^{51}V) [ppm]: −				−δ (^{51}V) [ppm]: 575.1			
		−	−	−	−	100	100	100	100 ± 0.0
	0.1 M HEPES[c] pH 7	−δ (^{51}V) [ppm]: −				−δ (^{51}V) [ppm]: 551.5, 557.8, 571.1, 575.9, 590.5			
		−	−	−	−	100	100	100	100 ± 0.0
	0.1 M Tris[d]-HCl pH 7	−δ (^{51}V) [ppm]: 527.6, 536.8, 553.0				−δ (^{51}V) [ppm]: 546.5, 558.3, 565.5, 571.2, 575.4, 583.9, 591.2			

		2.2	2.0	2.2	2.1 ± 0.1	97.8	98.0	97.8	97.9 ± 0.1
8	10% D_2O/H_2O pH 8	−δ (^{51}V) [ppm]: −				−δ (^{51}V) [ppm]: 558.3, 571.0, 575.4, 591.2			
		–	–	–	–	100	100	100	100 ± 0.0
	0.1 M sodium phosphate (HPO_4^{2-}/$H_2PO_4^{-}$) pH 8	−δ (^{51}V) [ppm]: 567.5				−δ (^{51}V) [ppm]: 575.0, 583.5			
		79.0	78.6	79.4	79.0 ± 0.4	21.0	21.4	20.6	21.0 ± 0.4
	0.1 M HEPES^c pH 8	−δ (^{51}V) [ppm]: −				−δ (^{51}V) [ppm]: 540.7, 544.0, 555.3, 570.4, 575.5, 584.2, 586.6			
		–	–	–	–	100	100	100	100 ± 0.0
	0.1 M Tris^d-HCl pH 8	−δ (^{51}V) [ppm]: 525.2, 538.0				−δ (^{51}V) [ppm]: 555.6, 570.6, 575.4, 587.4			
		10.8	10.9	11.3	11.0 ± 0.3	89.2	89.1	88.7	89.0 ± 0.3

^{a}SD – standard deviation; ^{b}RT- room temperature; ^{c}HEPES – 4-(2-hydroxyethyl)-1-piperazineethanesulfonic acid, $C_8H_{18}N_2O_4S$; ^{d}tris – tris(hydroxymethyl)aminomethane, $C_4H_{11}NO_3$.

Table S10. Analysis of NMR spectroscopic data recorded in {$Mo_{72}V_{30}$} solutions after 24 h incubation at 37 °C.

Chemical shifts in ^{51}V NMR spectra measured in triplicate of **{$Mo_{72}V_{30}$}** (0.15 mM) dissolved in D_2O and 0.1 M buffers (acetic acid – sodium acetate pH 4 – 5.5; sodium phosphate pH 2 – 8 (while phosphate does not buffer at pH range from 3.5 – 5.5, experiments were conducted at this pH to provide comparisons to previously published studies (*239*); Tris-HCl pH 7 – 8; HEPES pH 7 – 8) and measured after 24 h kept at 37 °C. The content of species was calculated based on the integration of ^{51}V signals. Signals were assigned based on the literature data summarized in **Table S5**. Percentages are normalized within each pH row and sum to ~100 % (small deviations reflect rounding or trace unassigned signals).

**pH**	**{$Mo_{72}V_{30}$} (0.15 mM) in Solvent / Buffer**	**% of all mixed V-Mo small POMs (Tables S11 – S14) or/and *unassigned* species in solution after 24 h incubation at 37 °C**				**% of all iso(poly)vanadate species (Tables S11 – S14) after 24 h incubation at 37 °C**			
		**Sample**			**Mean of 1 to 3 ± SD^a**	**Sample**			**Mean of 1 to 3 ± SD^a**
		**#1**	**#2**	**#3**		**#1**	**#2**	**#3**	
1	10% D_2O/H_2O pH 1	−δ (^{51}V) [ppm]: 504.7, 531.6				−δ (^{51}V) [ppm]: 542.7			
		6.1	8.1	9.5	7.9 ± 1.7	93.9	91.9	90.5	92.1 ± 1.7
1.5	10% D_2O/H_2O pH 1.5	−δ (^{51}V) [ppm]: 504.2, 507.2, 520.0, 525.4, 531.5				−δ (^{51}V) [ppm]: 543.0			
		35.1	36.0	30.9	34.0 ± 2.7	61.4	60.0	62.5	61.3 ± 1.3
		unassigned −δ (^{51}V) [ppm]: 517.4, 533.6							
		3.5	4.0	6.6	4.7 ± 1.7				
1.75	10% D_2O/H_2O pH 1.75	−δ (^{51}V) [ppm]: 504.6, 506.7, 516.9, 519.7, 524.7, 531.6				−δ (^{51}V) [ppm]: 542.5			
		67.1	68.2	68.3	67.9 ± 0.7	30.2	29.1	29.5	29.6 ± 0.5
		unassigned −δ (^{51}V) [ppm]: 509.2, 510.5, 533.3							
		2.7	2.7	2.3	2.6 ± 0.2				

2	10% D_2O/H_2O pH 2	−δ (^{51}V) [ppm]: 504.5, 506.3, 508.8, 516.4, 519.1, 524.2, 531.5				−δ (^{51}V) [ppm]: 542.7			
		85.0	85.5	86.5	85.6 ± 0.8	11.1	10.8	10.0	10.6 ± 0.6
		unassigned −δ (^{51}V) [ppm]: 509.4, 510.2, 532.7							
		3.9	3.8	3.5	3.7 ± 0.2				
	0.1 M sodium phosphate ($H_2PO_4^-$/H_3PO_4) pH 2	−δ (^{51}V) [ppm]: 516.0, 530.3, 532.7				−δ (^{51}V) [ppm]: 551.0			
		18.7	17.4	19.1	18.4 ± 0.9	81.3	82.6	80.9	81.6 ± 0.9
3	10% D_2O/H_2O pH 3	−δ (^{51}V) [ppm]: 504.4, 514.5, 531.7				−δ (^{51}V) [ppm]: 544.4			
		15.8	22.0	18.1	18.6 ± 3.1	60.4	50.8	60.8	57.3 ± 5.7
		unassigned −δ (^{51}V) [ppm]: 509.8, 532.3							
		23.8	27.2	21.1	24.1 ± 3.1				
	0.1 M sodium phosphate ($H_2PO_4^-$/H_3PO_4) pH 3	−δ (^{51}V) [ppm]: –				−δ (^{51}V) [ppm]: 557.4			
		–	–	–	–	100	100	100	100 ± 0.0
3.5	10% D_2O/H_2O pH 3.5	−δ (^{51}V) [ppm]: 504.5, 507.8, 514.6, 518.2, 523.5, 529.6, 532.3, 538.6				−δ (^{51}V) [ppm]: 423.9, 502.9, 509.6, 547.9			
		23.1	24.6	19.0	22.2 ± 2.9	76.9	75.4	81.0	77.8 ± 2.9
4	10% D_2O/H_2O pH 4	−δ (^{51}V) [ppm]: 502.7, 504.4, 507.0, 516.5, 523.3, 526.3, 532.2				−δ (^{51}V) [ppm]: 423.8, 500.2, 514.8, 557.7			
		17.5	17.6	15.1	16.7 ± 1.4	68.5	68.9	70.9	69.4 ± 1.3
		unassigned −δ (^{51}V) [ppm]: 508.4							
		14.0	13.5	14.1	13.8 ± 0.3				
	0.1 M sodium phosphate ($H_2PO_4^-$/H_3PO_4) pH 4	−δ (^{51}V) [ppm]:				−δ (^{51}V) [ppm]: 572.5			
		–	–	–	–	100	100	100	100 ± 0.0
	0.1 M acetic acid – sodium acetate (OAc^-/HOAc) pH 4	−δ (^{51}V) [ppm]: 502.9, 504.5, 506.4, 524.2, 521.0, 532.6				−δ (^{51}V) [ppm]: 424.0, 496.3, 515.2, 554.9			
		32.8	30.8	30.4	31.3 ± 1.3	67.1	69.1	69.5	68.6 ± 1.3
		unassigned −δ (^{51}V) [ppm]: 508.6							
		0.1	0.2	0.1	0.1 ± 0.1				
5	10% D_2O/H_2O pH 5	−δ (^{51}V) [ppm]: 502.7, 505.3, 523.8				−δ (^{51}V) [ppm]: 423.8, 495.7, 515.0, 558.6			
		7.5	7.0	6.5	7.0 ± 0.5	92.5	93.0	93.5	93.0 ± 0.5
	0.1 M sodium phosphate ($H_2PO_4^-$/H_3PO_4) pH 5	−δ (^{51}V) [ppm]: –				−δ (^{51}V) [ppm]: 579.8			
		–	–	–	–	100	100	100	100 ± 0.0
	0.1 M acetic acid – sodium acetate (OAc^-/HOAc) pH 5	−δ (^{51}V) [ppm]: 502.8, 513.0, 523.4				−δ (^{51}V) [ppm]: 423.0, 494.0, 499.5, 514.5, 558.4			
		15.6	18.0	17.6	17.1 ± 1.3	84.4	82.0	82.4	82.9 ± 1.3
5.5	0.1 M acetic acid – sodium acetate	−δ (^{51}V) [ppm]: 502.8, 514.4, 523.4				−δ (^{51}V) [ppm]: 423.4, 493.9, 512.8, 558.5, 571.3, 575.2			

	(OAc⁻/HOAc) pH 5.5	18.7	17.5	18.3	18.2 ± 0.6	81.3	82.5	81.7	81.8 ±0.6
6	10% D_2O/H_2O pH 6	−δ (^{51}V) [ppm]: –				−δ (^{51}V) [ppm]: 558.2, 571.1			
		–	–	–	–	100	100	100	100 ± 0.0
	0.1 M sodium phosphate (HPO_4^{2-}/$H_2PO_4^-$) pH 6	−δ (^{51}V) [ppm]: 496.8, 514.4, 533.2				−δ (^{51}V) [ppm]: 423.3, 493.9, 502.9, 512.8, 523.3, 575.3, 580.7			
		1.5	1.4	1.6	1.5 ± 0.1	98.5	98.6	98.4	98.5 ± 0.1
7	10% D_2O/H_2O pH 7	−δ (^{51}V) [ppm]: –				−δ (^{51}V) [ppm]: 558.4, 571.0, 575.5			
		–	–	–	–	100	100	100	100 ± 0.0
	0.1 M sodium phosphate (HPO_4^{2-}/$H_2PO_4^-$) pH 7	−δ (^{51}V) [ppm]: –				575.1, 583.3, 594.5, 603.9			
		–	–	–	–	100	100	100	100 ± 0.0
	0.1 M HEPES[c] pH 7	−δ (^{51}V) [ppm]: –				−δ (^{51}V) [ppm]: 552.2, 558.0, 571.2, 575.8, 584.9, 590.7			
		–	–	–	–	100	100	100	100 ± 0.0
	0.1 M Tris[d]-HCl pH 7	−δ (^{51}V) [ppm]: 516.7, 528.0, 537.2				−δ (^{51}V) [ppm]: 422.8, 493.6, 502.5, 514.1, 523.0, 546.5, 558.5, 571.2, 575.4, 591.5			
		3.6	4.3	3.4	3.8 ± 0.5	96.4	95.7	96.6	96.2 ± 0.5
8	10% D_2O/H_2O pH 8	−δ (^{51}V) [ppm]: –				−δ (^{51}V) [ppm]: 558.4, 571.1, 575.4, 591.5			
		–	–	–	–	100	100	100	100 ± 0.0
	0.1 M sodium phosphate (HPO_4^{2-}/$H_2PO_4^-$) pH 8	−δ (^{51}V) [ppm]: 569.5				−δ (^{51}V) [ppm]: 575.1, 583.5, 594.6			
		38.5	37.1	38.0	37.9 ± 0.7	61.5	62.9	62.0	62.1 ± 0.7
	0.1 M HEPES[c] pH 8	−δ (^{51}V) [ppm]: –				−δ (^{51}V) [ppm]: 545.8, 556.3, 570.7, 575.5, 584.2, 588.1, 594.9, 604.2			
		–	–	–	–	100	100	100	100 ± 0.0
	0.1 M Tris[d]-HCl pH 8	−δ (^{51}V) [ppm]: 485.0, 509.2, 525.1, 536.6, 541.7,				−δ (^{51}V) [ppm]: 546.4, 556.6, 564.1, 570.7, 575.3, 583.7, 588.5, 594.6			
		7.1	5.9	4.2	5.7 ± 1.5	92.9	94.1	95.8	94.3 ± 1.5

[a]SD – standard deviation; [b]RT- room temperature; [c]HEPES – 4-(2-hydroxyethyl)-1-piperazineethanesulfonic acid, $C_8H_{18}N_2O_4S$; [d]tris – tris(hydroxymethyl)aminomethane, $C_4H_{11}NO_3$.Iso(poly)vanadates include V_{10} and V_9Mo_1 decavanadate species.

Table S11. Overview of Mo-V and V-based species detected by ^{51}V NMR (based on data in Tables S8 – S10) in H_2O with pH 1 – 8 after dissolution of $\{Mo_{72}V_{30}\}$ at three different conditions: fresh solutions, 24 h at room temperature, and 24 h at 37 °C. Column totals may be < 100 % due to unassigned signals not itemized in this table.

POM anion structure	solutions	pH 1	pH 1.5	pH 1.75	pH 2	pH 3	pH 3.5	pH 4	pH 5	pH 6	pH 7	pH 8
$[V^{V}Mo^{VI}_5O_{19}]^{3-}$	fresh	1.1 ± 0.0 %	1.2 ± 0.1 %	29.9 ± 4.5 %	25.0 ± 7.1 %	0.7 ± 0.3 %	4.7 ± 1.2 %	1.5 ± 0.0 %	1.3 ± 0.3 %	-	-	-
	RT	0.9 ± 0.2 %	0.9 ± 0.1 %	50.5 ± 3.7 %	67.9 ± 3.6 %	1.2 ± 0.6 %	6.3 ± 1.3 %	1.0 ± 0.2 %	-	-	-	-
	inc.	0.7 ± 0.0 %	0.7 ± 0.1 %	54.7 ± 1.6 %	77.7 ± 0.7 %	8.9 ± 1.3 %	8.2 ± 1.0 %	1.9 ± 0.5 %	0.7 ± 0.3 %	-	-	-
$[HV^{V}_2Mo^{VI}_4O_{19}]^{3-}$	fresh	-	-	1.7 ± 0.5 %	-	-	2.0 ± 0.5 %	1.2 ± 0.6 %	-	-	-	-
	RT	-	-	4.3 ± 0.7 %	1.2 ± 0.2 %	-	1.3 ± 0.7 %	0.7 ± 0.3 %	-	-	-	-
	inc.	-	-	3.1 ± 0.8 %	2.2 ± 0.5 %	-	2.5 ± 0.9 %	1.2 ± 0.8 %	-	-	-	-
α-$[V^{V}Mo^{VI}_7O_{26}]^{5-}$	fresh	-	-	-	-	-	-	6.5 ± 0.5 %	-	-	-	-
	RT	-	-	-	-	-	-	6.2 ± 0.3 %	-	-	-	-
	inc.	-	-	-	-	-	-	7.4 ± 0.9 %	-	-	-	-
α-$[HV^{V}Mo^{VI}_7O_{26}]^{4-}$	fresh	-	-	-	-	-	-	-	-	-	-	-
	RT	-	-	-	-	-	-	-	2.0 ± 0.0 %	-	-	-
	inc.	-	-	-	-	-	-	-	4.7 ± 0.8 %	-	-	-
β-$[V^{V}Mo^{VI}_7O_{26}]^{5-}$	fresh	5.5 ± 0.0 %	5.4 ± 0.2 %	6.8 ± 3.4 %	15.1 ± 4.2 %	7.2 ± 1.8 %	5.9 ± 1.1 %	-	-	-	-	-
	RT	7.1 ± 1.6 %	6.8 ± 1.1 %	1.9 ± 1.0 %	0.7 ± 0. 1%	2.5 ± 1.3 %	7.7 ± 1.6 %	2.2 ± 0.7 %	-	-	-	-
	inc.	12.1 ± 8.9 %	11.7 ± 3.4 %	1.5 ± 0.7 %	0.6 ± 0.1 %	8.4 ± 1.8 %	4.9 ± 1.3 %	0.8 ± 0.3 %	-	-	-	-
β-$[H_2V^{V}_2Mo^{VI}_6O_{26}]^{4-}$	fresh	-	-	-	-	-	0.8 ± 0.3 %	-	-	-	-	-
	RT	-	-	-	-	-	1.2 ± 0.5 %	1.0 ± 0.5 %	-	-	-	-
	inc.	-	-	-	-	-	1.9 ± 0.4 %	-	-	-	-	-
$[HV^{V}_2Mo^{VI}_7O_{38}]^{5-}$	fresh	-	-	-	-	-	-	-	0.7 ± 0.3 %	-	-	-
	RT	-	-	-	-	-	-	-	0.8 ± 0.3 %	-	-	-
	inc.	-	-	-	-	-	-	-	1.7 ± 0.3 %	-	-	-
$[HV^{V}_3Mo^{VI}_6O_{38}]^{6-}$	fresh	-	-	-	-	-	-	2.5 ± 0.5 %	-	-	-	-
	RT	-	-	-	-	-	-	3.0 ± 0.5 %	-	-	-	-
	inc.	-	-	-	-	-	-	3.5 ± 1.3 %	-	-	-	-
$[HV^{V}Mo^{VI}_9O_{32}]^{4-}$	fresh	-	-	-	-	-	0.9 ± 0.4 %	1.5 ± 0.0 %	-	-	-	-

	RT	-	-	-	-	1.5 ± 0.5 %	1.3 ± 0.6 %	1.4 ± 0.3 %	-	-	-	-
	inc.	-	-	-	-	1.3 ± 0.4 %	2.5 ± 1.4 %	1.9 ± 0.3 %	-	-	-	-
	fresh	-	-	1.8 ± 0.6 %	-	-	1.5 ± 0.5 %	-	-	-	-	-
**$[HV^{V}{}_{2}Mo^{VI}{}_{8}O_{32}]^{5-}$**	RT	-	-	4.5 ± 0.1 %	0.8 ± 0.1 %	-	1.0 ± 0.3 %	-	-	-	-	-
	inc.	-	-	4.5 ± 0.2 %	1.1 ± 0.1 %	-	0.4 ± 0.2 %	-	-	-	-	-
	fresh	-	-	-	-	-	0.6 ± 0.1 %	-	-	-	-	-
**$[HV^{V}{}_{2}Mo^{VI}{}_{10}O_{38}]^{5-}$**	RT	-	-	-	-	-	1.2 ± 0.4 %	-				
	inc.	-	-	-	-	-	0.4 ± 0.2 %	-				
	fresh	-	-	-	-	-	-	-				
**$[V^{V}{}_{3}Mo^{VI}{}_{9}O_{38}]^{7-}$**	RT	-	-	2.8 ± 0.3 %	2.8 ± 0.5 %	-	-	-				
	inc.	-	-	2.7 ± 0.5 %	1.7 ± 0.3 %	-	-	-				
	fresh	-	-	-	-	-	0.4 ± 0.1 %	-				
**α-$[V^{V}{}_{2}Mo^{VI}{}_{18}O_{62}]^{6-}$**	RT	-	-	-	1.2 ± 0.1 %	2.3 ± 0.8 %	0.3 ± 0.0 %	-				
	inc.	-	-	1.4 ± 0.1 %	2.3 ± 0.4 %	-	1.4 ± 0.6 %	-				
	fresh	-	-	-	-	-	36.4 ± 4.3 %	9.4 ± 1.1 %	1.7 ± 0.3 %	-	-	-
**$[V^{V}{}_{10}O_{28}]^{6-}$**	RT	-	-	-	-	-	38.0 ± 3.6 %	5.9 ± 0.6 %	5.0 ± 0.5 %	-	-	-
	inc.	-	-	-	-	-	42.0 ± 2.5 %	9.3 ± 3.1 %	8.7 ± 0.6 %	-	-	-
	fresh	-	-	-	-	-	-	-	-	-	-	-
**$[V^{V}{}_{4}O_{12}]^{4-}$**	RT	-	-	-	-	-	-	-	-	-	-	1.3 ± 0.1 %
	inc.	-	-	-	-	-	-	-	-	-	2.1 ± 0.3 %	4.6 ± 0.3 %
	fresh	-	-	-	-	-	-	-	-	-	-	91.4 ± 0.1 %
**$[V^{V}{}_{3}O_{10}]^{5-}$**	RT	-	-	-	-	-	-	-	-	-	-	
	inc.	-	-	-	-	-	-	-	-	-	-	
	fresh	-	-	-	-	-	-	-	-	5.7 ± 0.4 %	8.2 ± 0.2 %	8.6 ± 0.1 %
**$[HV^{V}{}_{3}O_{10}]^{4-}$**	RT	-	-	-	-	-	-	-	-	6.5 ± 0.5 %	9.3 ± 0.9 %	10.6 ± 0.7 %
	inc.	-	-	-	-	-	-	-	-	6.5 ± 0.3 %	10.4 ± 1.7 %	13.8 ± 0.8 %
	fresh			59.9 ± 2.5 %	59.8 ± 6.2 %		46.7 ± 6.0 %	-	-	-	-	-
**$[V^{V}O_{2}]^{+}$**	RT			34.0 ± 1.1 %	21.1 ± 4.7 %		41.1 ± 3.6 %	-	-	-	-	-
	inc.			29.6 ± 0.5 %	10.6 ± 0.6 %	57.3 ± 5.7 %	35.7 ± 4.6 %	-	-	-	-	-

$[H_xVO_4]^{x-3}$ x=0-2	fresh	-	-	-	-	-	-	60.9 ± 1.9 %	96.3 ± 0.3 %	94.3 ± 0.4 %	91.8 ± 0.2 %	-
	RT	-	-	-	-	-	-	64.8 ± 0.7 %	92.2 ± 0.7 %	93.5 ± 0.5 %	90.7 ± 0.9 %	-
	inc.	-	-	-	-	-	-	60.2 ± 2.9 %		93.5 ± 0.3 %		-

Table S12. Overview of Mo-V and V-based species detected by ^{51}V NMR (based on data in Tables S8 – S10) in 0.1 M sodium phosphate buffer solutions with pH 2 – 8 after dissolution of $\{Mo_{72}V_{30}\}$ at three different conditions: fresh solutions, 24 h at room temperature, and 24 h at 37 °C. Percentages are normalized within each pH column and sum to ~100 % (small deviations reflect rounding or trace unassigned signals).

POM anion structure	solutions	pH 2	pH 3	pH 4	pH 5	pH 6	pH 7	pH 8
$[V^{V}Mo^{VI}_4O_{17}]^{5-}$	fresh	-	-	-	-	-	-	74.1 ± 1.2 %
	RT	-	-	-	-	-	-	79.0 ± 0.4 %
	inc.	-	-	-	-	-	-	37.9 ± 0.7 %
$[V^{V}_2Mo^{VI}_4O_{19}]^{4-}$	fresh	-	-	-	-	-	-	-
	RT	-	-	-	-	-	-	-
	inc.	-	-	-	-	0.5 ± 0.1 %	-	-
β-$[V^{V}Mo^{VI}_7O_{26}]^{5-}$	fresh	-	-	-	-	-	-	-
	RT	-	-	-	-	-	-	-
	inc.	13.0 ± 0.9 %	-	-	-	0.5 ± 0.1 %	-	-
$[HV^{V}Mo^{VI}_9O_{32}]^{4-}$	fresh	-	-	-	-	-	-	-
	RT	-	-	-	-	-	-	-
	inc.	2.2 ± 0.9 %	-	-	-	-	-	-
α-$[V^{V}_2Mo^{VI}_{18}O_{62}]^{6-}$	fresh	-	-	-	-	-	-	-
	RT	-	-	-	-	-	-	-
	inc.	3.2 ± 0.9 %	-	-	-	-	-	-
$[V^{V}_9Mo^{VI}O_{28}]^{5-}$	fresh	-	-	-	-	-	-	-
	RT	-	-	-	-	-	-	-
	inc.	-	-	-	-	3.7 ± 0.3 %	-	-
$[V^{V}_5O_{15}]^{5-}$	fresh	-	-	-	-	-	-	0.3 ± 0.1 %
	RT	-	-	-	-	-	-	0.5 ± 0.1 %
	inc.	-	-	-	-	-	2.6 ± 1.9 %	2.6 ± 0.3 %

**$[V^{V}_{4}O_{12}]^{4-}$**	fresh	-	-	-	100 ± 0.0 %	100 ± 0.0 %	100 ± 0.0 %	14.2 ± 1.9 %
	RT	-	-	-	100 ± 0.0 %	100 ± 0.0 %	100 ± 0.0 %	20.6 ± 0.4 %
	inc.	-	-	-	100 ± 0.0 %	88.3 ± 0.5 %	96.6 ± 2.0 %	59.4 ± 0.9 %
**$[H_2V^{V}_{4}O_{13}]^{4-}$**	fresh	-	-	-	-	-	-	-
	RT	-	-	-	-	-	-	-
	inc.	-	-	-	-	-	0.9 ± 0.1 %	-
**$[H_xV^{V}_{3}O_{10}]^{4-}$ x=1-2**	fresh	-	-	-	-	-	-	11.4 ± 0.9 %
	RT	-	-	-	-	-	-	-
	inc.	-	-	-	-	-	-	-
**$[H_2V^{V}_{2}O_7]^{2-}$**	fresh	-	-	100 ± 0.0 %	-	-	-	-
	RT	-	-	100 ± 0.0 %	-	-	-	-
	inc.	-	-	100 ± 0.0 %	-	6.4 ± 0.2 %	-	-
**$[H_xVO_4]^{x-3}$ x=0-2**	fresh	100 ± 0.0 %	100 ± 0.0 %	-	-	-	-	-
	RT	100 ± 0.0 %	100 ± 0.0 %	-	-	-	-	-
	inc.		100 ± 0.0 %	-	-	-	-	-

Table S13. Overview of Mo-V and V-based species detected by ^{51}V NMR (based on data in Tables S8 – S10) in 0.1 M acetic acid – sodium acetate buffer solutions with pH 4 – 5.5 after dissolution of {$Mo_{72}V_{30}$} at three different conditions: fresh solutions, 24 h at room temperature, and 24 h at 37 °C. Percentages are normalized within each pH column and sum to ~100 % (small deviations reflect rounding or trace unassigned signals).

POM anion structure	solutions	pH 4	pH 5	pH 5.5
**$[V^{V}Mo^{VI}_{5}O_{19}]^{3-}$**	fresh	9.7 ± 0.4 %	-	-
	RT	9.2 ± 2.6 %	-	-
	inc.	4.3 ± 0.3 %	-	-
**$[HV^{V}_{2}Mo^{VI}_{4}O_{19}]^{3-}$**	fresh	-	4.0 ± 0.3 %	-
	RT	-	4.7 ± 0.3 %	-
	inc.	-	5.8 ± 0.9 %	-
**α-$[V^{V}Mo^{VI}_{7}O_{26}]^{5-}$**	fresh	13.5 ± 0.6 %	8.0 ± 1.2 %	-
	RT	15.5 ± 0.5 %	8.2 ± 0.8 %	1.2 ± 0.3 %
	inc.	17.9 ± 1.0 %	11.3 ± 0.4 %	7.9 ± 0.4 %

**β-$[V^{V}Mo^{VI}_{7}O_{26}]^{5-}$**	fresh	-	-	-
	RT	-	-	-
	inc.	0.5 ± 0.1 %	-	-
**$[HV^{V}Mo^{VI}_{9}O_{32}]^{4-}$**	fresh	-	-	-
	RT	-	1.3 ± 0.3 %	2.7 ± 0.3 %
	inc.	-	-	6.4 ± 0.6 %
**$[HV^{V}_{2}Mo^{VI}_{8}O_{32}]^{5-}$**	fresh	-	-	-
	RT	-	-	-
	inc.	1.3 ± 0.5 %	-	-
**α-$[V^{V}_{2}Mo^{VI}_{18}O_{62}]^{6-}$**	fresh	6.5 ± 0.6 %	3.6 ± 0.2 %	-
	RT	7.2 ± 0.3 %	-	0.7 ± 0.3 %
	inc.	7.3 ± 0.1 %	-	3.9 ± 0.4%
**$[V^{V}_{9}Mo^{VI}O_{28}]^{5-}$**	fresh	-	-	-
	RT	-	24.5 ± 0.1 %	-
	inc.	-	29.6 ± 0.5 %	-
**$[V^{V}_{10}O_{28}]^{6-}$**	fresh	29.6 ± 1.8 %	12.6 ± 1.1 %	-
	RT	31.3 ± 2.2 %	-	-
	inc.	34.4 ± 1.3 %	-	15.9 ± 1.3 %
**$[V^{V}_{4}O_{12}]^{4-}$**	fresh	-	-	3.3 ± 0.6 %
	RT	-	-	2.8 ± 0.3 %
	inc.	-	-	3.3 ± 0.3 %
**$[H_{x}V^{V}_{3}O_{10}]^{4-}$ x=1-2**	fresh	-	-	-
	RT	-	-	4.7 ± 1.5 %
	inc.	-	-	-
**$[H_{2}V^{V}_{2}O_{7}]^{2-}$**	fresh	-	-	5.9 ± 0.5%
	RT	-	-	-
	inc.	-	-	3.9 ± 0.3 %
**$[H_{x}VO_{4}]^{x-3}$ x=0-2**	fresh	40.7 ± 2.2 %	71.9 ± 1.3 %	90.8 ± 1.1 %

	RT	36.8 ± 1.7 %	61.3 ± 0.7 %	88.0 ± 1.7 %
	inc.	34.2 ± 2.5 %	53.3 ± 1.6 %	59.4 ± 0.7 %

Table S14. Overview of Mo-V and V-based species detected by ^{51}V NMR (based on data in Tables S8 – S10) in 0.1 M Tris-HCl buffer and 0.1 M HEPES buffer solutions with pH 7 – 8 after dissolution of {$Mo_{72}V_{30}$}at three different conditions: fresh solutions, 24 h at room temperature, and 24 h at 37 °C. Percentages are normalized within each pH column and sum to ~100 % (small deviations reflect rounding or trace unassigned signals).

		**Tris-HCl**		**HEPES**	
**POM anion structure**	**solutions**	**pH 7**	**pH 8**	**pH 7**	**pH 8**
**$[HV^{V}_2Mo^{VI}_4O_{19}]^{3-}$**	fresh	-	-	-	-
	RT	-	-	-	-
	inc.	-	0.1 ± 0.0 %	-	-
**β-$[V^{V}Mo^{VI}_7O_{26}]^{5-}$**	fresh	0.7 ± 0.0 %	8.7 ± 0.7 %	-	-
	RT	1.8 ± 0.1 %	8.9 ± 0.1 %	-	-
	inc.	1.5 ± 0.3 %	1.6 ± 0.8 %	-	-
**β-$[H_2V^{V}_2Mo^{VI}_6O_{26}]^{4-}$**	fresh	-	-	-	-
	RT	-	-	-	-
	inc.	-	-	-	-
**$[V^{V}_2Mo^{VI}_6O_{26}]^{6-}$**	fresh	-	-	-	-
	RT	-	-	-	-
	inc.	-	0.4 ± 0.0 %	-	-
**$[HV^{V}Mo^{VI}_9O_{32}]^{4-}$**	fresh	-	-	-	-
	RT	-	-	-	-
	inc.	1.8 ± 0.2 %	-	-	-
**$[HV^{V}_2Mo^{VI}_{10}O_{38}]^{5-}$**	fresh	-	3.0 ± 0.5 %	-	-
	RT	-	2.2 ± 0.2 %	-	-
	inc.	-	3.6 ± 0.7 %	-	-
**α-$[V^{V}_2Mo^{VI}_{18}O_{62}]^{6-}$**	fresh	-	-	-	-
	RT	0.3 ± 0.0 %	-	-	-
	inc.	0.5 ± 0.2 %	-	-	-
**$[V^{V}_9Mo^{VI}O_{28}]^{5-}$**	fresh	-	-	-	-

	RT	-	-	-	-
	inc.	13.2 ± 0.2 %	-	-	-
**$[V^{V}_{6}O_{18}]^{6-}$**	fresh	-	-	-	-
	RT	-	-	-	-
	inc.	-	-	-	0.3 ± 0.0 %
**$[V^{V}_{5}O_{15}]^{5-}$**	fresh	-	-	-	0.8 ± 0.1 %
	RT	-	-	-	0.9 ± 0.1 %
	inc.	-	0.1 ± 0.0 %	-	-
**$[V^{V}_{4}O_{12}]^{4-}$**	fresh	11.6 ± 0.4 %	6.4 ± 0.4 %	1.4 ± 0.1 %	13.4 ± 0.8 %
	RT	26.7 ± 1.4 %	3.5 ± 1.0 %	5.9 ± 0.5 %	12.2 ± 3.3 %
	inc.	19.8 ± 0.2 %	45.0 ± 2.4 %	20.8 ± 0.8 %	53.9 ± 2.3 %
**$[H_{x}V^{V}_{4}O_{13}]^{(5-x)-}$, x= 0 – 1**	fresh	-	-	-	0.2 ± 0.1 %
	RT	1.0 ± 0.7 %	-	-	0.2 ± 0.1 %
	inc.	-	4.0 ± 0.4 %	0.2 ± 0.0 %	2.2 ± 0.0 %
**$[H_{2}V^{V}_{4}O_{13}]^{4-}$**	fresh	-	-	-	-
	RT	-	-	-	-
	inc.	-	-	-	0.2 ± 0.0 %
**$[V^{V}_{3}O_{10}]^{5-}$**	fresh	68.8 ± 2.3 %	-	87.5 ± 0.5 %	-
	RT	53.0 ± 2.1 %	-	77.6 ± 1.2 %	-
	inc.	47.2 ± 0.1 %	26.6 ± 2.1 %	58.7 ± 0.8 %	-
**$[HV^{V}_{3}O_{10}]^{4-}$**	fresh	-	11.0 ± 0.4 %	-	15.9 ± 0.3 %
	RT	-	9.1 ± 0.9 %	-	15.4 ± 1.2 %
	inc.	-	15.1 ± 1.8 %	-	13.9 ± 0.8 %
**$[H_{x}V^{V}_{2}O_{7}]^{(4-x)-}$ x=0-2**	fresh	13.8 ± 0.5 %	70.9 ± 1.1 %	10.3 ± 0.2 %	69.4 ± 1.0 %
	RT	15.0 ± 0.7 %	76.3 ± 2.2 %	15.9 ± 0.9 %	70.9 ± 4.5 %
	inc.	15.4 ± 0.8 %	-	19.8 ± 0.6 %	29.5 ± 1.5 %
**$[H_{x}V^{V}O_{4}]^{x-3}$ x=0-2**	fresh	5.1 ± 0.1 %	3.2 ± 0.5 %	0.9 ± 0.2 %	0.4 ± 0.1 %
	RT	1.5 ± 0.7 %	-	0.6 ± 0.2 %	0.2 ± 0.1 %
	inc.	-	2.7 ± 0.7 %	0.5 ± 0.1 %	-
**$[V^{V}O_{2}]^{+}$**	fresh	-	-	-	-

	RT	0.7 ± 0.1 %	-	-	0.2 ± 0.0 %
	inc.	0.6 ± 0.1 %	-	-	0.2 ± 0.0 %

5.3. Resonance Raman spectroscopic studies of {$Mo_{72}V_{30}$} solutions

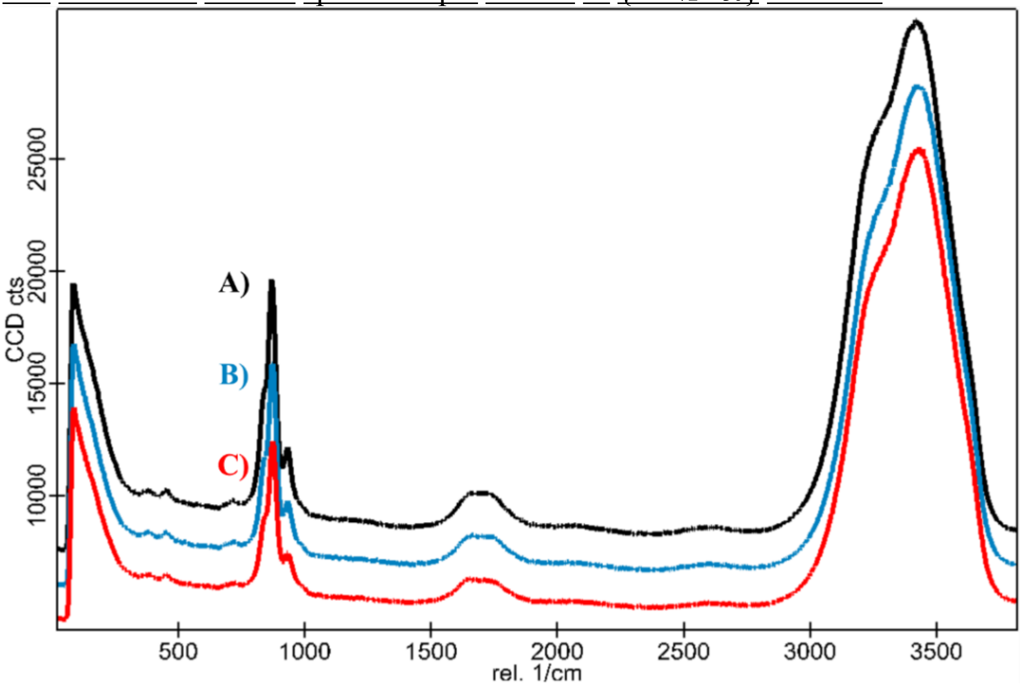

Fig. S18. Resonance Raman spectra of {$Mo_{72}V_{30}$} in H_2O with pH 2: A) fresh solutions; B) 24 h aging at room temperature and C) 24 h incubation at 37 °C.

Resonance Raman spectra for 0.15 mM fresh solutions (**A**) of $Na_8K_{16}(VO)(H_2O)_5[K_{10}\subset\{(Mo)Mo_5O_{21}(H_2O)_3(SO_4)\}_{12}(VO)_{30}(H_2O)_{20}]\cdot 150H_2O$ ({$Mo_{72}V_{30}$}) in H_2O that were recorded approximately 30 min after preparation. Aliquots of fresh solutions were taken for 24 h aging experiments at room temperature (**B**) and 24 h incubation at 37 °C (**C**) and were then recorded approximately 30 min after the end of 24 h experiments. The RR shifts of formed POM species are given in **Table S15**.

Table S15. Analysis of Resonance Raman spectroscopic data recorded of {$Mo_{72}V_{30}$} solutions at three different conditions: fresh solutions, 24 h at room temperature, and 24 h at 37 °C.

Resonance Raman (λ_e = 532 nm, E = 25 mW) shifts measured in duplicate of {$Mo_{72}V_{30}$} (0.15 mM) dissolved in H_2O (pH 1 – 8) or 0.1 M buffers (acetic acid – sodium acetate pH 4 – 5.5; sodium phosphate pH 2 – 8; Tris-HCl pH 7 – 8; HEPES pH 7 – 8). The resonance Raman shifts were assigned based on the literature data summarized in **Table S6**. The RR spectra of stable conditions (H_2O, pH 2) is shown on **Figure S18.** RR shifts of the intact Keplerates are are shown in bold.

**Sample condition**		**Resonance Raman shifts/ cm^{-1}**	**Species detected**	**Assignment according to literature**
		**H_2O, c($Mo_{72}V_{30}$)= 0.15 mM**		
**pH 1**	fresh	**452 (w, mb), 718 (w, b), 842 (sh), 873 (st, s), 934 (m, s), 992 (sh)**	**{$Mo_{72}V_{30}$}**	ν_{as+s}(O−V−O), ν_s(V−O)/ ν_s(Mo−O), ν(V=O)/ ν(Mo=O)
	24 h at RT	950 (w, mb), 1091 (m, mb)	$[Mo_3O_{10}]^{2-}$	ν_{as}(Mo=O)
			$V_1 - V_5$ isopolyvanadates	ν_{as}(V=O) bands between 900 – 1100 cm^{-1}
	24 h at 37 °C	923 (w, mb), 949 (w, mb)	α-$[Mo_8O_{26}]^{4-}$	ν_{as}(Mo−O_t) = 915, ν_s(Mo−O_t) =950 in Mo-V solutions
**pH 1.5**	fresh	**452 (w, mb), 718 (w, b), 842 (sh), 873 (st, s), 935 (m, s), 993 (sh)**	**{$Mo_{72}V_{30}$}**	ν_{as+s}(O−V−O), ν_s(V−O)/ ν_s(MoO, ν(V=O)/ ν(Mo=O)
	24 h at RT	872 (w, mb), 947 (w, mb), 1094 (w, b)	$[Mo_3O_{10}]^{2-}$	ν_s(Mo−O), ν_{as}(Mo=O)
			$V_1 - V_5$ isopolyvanadates	ν_{as}(V=O) bands between 900 – 1100 cm^{-1}
	24 h at 37 °C	863 (w, b), 913 (w, mb), 948 (w, mb)	α-$[Mo_8O_{26}]^{4-}$	ν_{as}(Mo−O_t), ν_s(Mo−O_t)
**pH 2**	fresh	**451 (w, mb), 717 (w, mb), 843 (sh), 871 (st, s), 933 (m, s), 993 (sh)**	**{$Mo_{72}V_{30}$}**	ν_{as+s}(O−V−O), ν_s(V−O)/ ν_s(Mo−O), ν(V=O)/ ν(Mo=O)
	24 h at RT	**451 (w, mb), 718 (w, mb), 842 (sh), 873 (st, s), 933 (m, s), 993 (sh)**	**{$Mo_{72}V_{30}$}**	ν_{as+s}(O−V−O), ν_s(V−O)/ ν_s(Mo−O), ν(V=O)/ ν(Mo=O)
	24 h at 37 °C	**452 (w, mb), 717 (w, mb), 842 (sh), 874 (st, s), 934 (m, s), 993 (sh)**	**{$Mo_{72}V_{30}$}**	ν_{as+s}(O−V−O), ν_s(V−O)/ ν_s(Mo−O), ν(V=O)/ ν(Mo=O)
**pH 3**	fresh	**451 (w, mb), 718 (w, mb), 842 (sh), 871 (st, s), 933 (m, s), 993 (sh)**	**{$Mo_{72}V_{30}$}**	ν_{as+s}(O−V−O), ν_s(V−O)/ ν_s(Mo−O), ν(V=O)/ ν(Mo=O)
	24 h at RT	**451 (w, mb), 720 (w, mb), 843 (sh), 873 (st, s), 933 (m, s), 993 (sh)**	**{$Mo_{72}V_{30}$}**	ν_{as+s}(O−V−O), ν_s(V−O)/ ν_s(Mo−O), ν(V=O)/ ν(Mo=O)
	24 h at 37 °C	**452 (w, mb), 719 (w, mb), 842 (sh), 873 (st, s), 933 (m, s), 993 (sh)**	**{$Mo_{72}V_{30}$}**	ν_{as+s}(O−V−O), ν_s(V−O)/ ν_s(Mo−O), ν(V=O)/ ν(Mo=O)
**pH 4**	fresh	**451 (w, mb),** 697 (sh), **721 (w, mb), 842 (sh), 871 (st, s), 934 (m, s), 993 (sh)**	**{$Mo_{72}V_{30}$}**	ν_{as+s}(O−V−O), ν_s(V−O)/ ν_s(Mo−O), ν(V=O)/ ν(Mo=O)
			unassigned ? (697 (sh))	ν_{as}(Mo−O−Mo)
	24 h at RT	**451 (w, mb), 718 (w, mb), 842 (sh), 870 (st, s), 933 (m, s), 993 (sh)**	**{$Mo_{72}V_{30}$}**	ν_{as+s}(O−V−O), ν_s(V−O)/ ν_s(Mo−O), ν(V=O)/ ν(Mo=O)
	24 h at 37 °C	**451 (w, mb), 718 (w, mb), 842 (sh), 871 (st, s), 933 (m, s), 993 (sh)**	**{$Mo_{72}V_{30}$}**	ν_{as+s}(O−V−O), ν_s(V−O)/ ν_s(Mo−O), ν(V=O)/ ν(Mo=O)
**pH 5**	fresh	**452 (w, mb), 717 (w, mb), 843 (sh), 872 (st, s), 934 (m, s), 992 (sh)**	**{$Mo_{72}V_{30}$}**	ν_{as+s}(O−V−O), ν_s(V−O)/ ν_s(Mo−O), ν(V=O)/ ν(Mo=O)
	24 h at RT	**451 (w, mb), 718 (w, mb), 842 (sh), 871 (st, s), 933 (m, s), 993 (sh)**	**{$Mo_{72}V_{30}$}**	ν_{as+s}(O−V−O), ν_s(V−O)/ ν_s(Mo−O), ν(V=O)/ ν(Mo=O)
	24 h at 37 °C	**452 (w, mb), 715 (w, mb), 842 (sh), 872 (st, s), 933 (m, s), 991 (sh)**	**{$Mo_{72}V_{30}$}**	ν_{as+s}(O−V−O), ν_s(V−O)/ ν_s(Mo−O), ν(V=O)/ ν(Mo=O)

**pH 6**	fresh	**452 (w, mb), 718 (w, mb), 842 (sh), 872 (st, s), 934 (m, s), 991 (sh)**	**{Mo$_{72}$V$_{30}$}**	ν_{as+s}(O−V−O), ν_s(V−O)/ ν_s(Mo−O), ν(V=O)/ ν(Mo=O)
	24 h at RT	**450 (w, mb),** 695 (sh), **718 (w, mb), 842 (sh), 871 (st, s), 933 (m, s), 993 (sh)**	**{Mo$_{72}$V$_{30}$}**	ν_{as+s}(O−V−O), ν_s(V−O)/ ν_s(Mo−O), ν(V=O)/ ν(Mo=O)
			unassigned ? (695 (sh))	ν_{as}(Mo–O−Mo)
	24 h at 37 °C	**452 (w, mb), 718 (w, mb), 842 (sh), 871 (st, s), 933 (m, s), 990 (sh)**	**{Mo$_{72}$V$_{30}$}**	ν_{as+s}(O−V−O), ν_s(V−O)/ ν_s(Mo−O), ν(V=O)/ ν(Mo=O)
**pH 7**	fresh	**451 (w, mb), 717 (w, mb), 842 (sh), 872 (st, s), 934 (m, s), 992 (sh)**	**{Mo$_{72}$V$_{30}$}**	ν_{as+s}(O−V−O), ν_s(V−O)/ ν_s(Mo−O), ν(V=O)/ ν(Mo=O)
	24 h at RT	**451 (w, mb).** 692 (sh), **718 (w, mb), 842 (sh), 871 (st, s), 933 (m, s), 991 (sh)**	**{Mo$_{72}$V$_{30}$}**	ν_{as+s}(O−V−O), ν_s(V−O)/ ν_s(Mo−O), ν(V=O)/ ν(Mo=O)
			unassigned ? (692 (sh))	ν_{as}(Mo–O−Mo)
	24 h at 37 °C	**451 (w, mb), 718 (w, mb), 844 (sh), 870 (st, s), 933 (m, s), 990 (sh)**, 1001 (sh)	**{Mo$_{72}$V$_{30}$}**	ν_{as+s}(O−V−O), ν_s(V−O)/ ν_s(Mo−O), ν(V=O)/ ν(Mo=O)
			V_1 − V_5 isopolyvanadates	ν_{as}(V=O) bands between 900 – 1100 cm^{-1}
**pH 8**	fresh	**451 (w, mb),** 689 (sh), **718 (w, mb), 841 (sh), 873 (st, s), 933 (m, s), 992 (sh)**	**{Mo$_{72}$V$_{30}$}**	ν_{as+s}(O−V−O), ν_s(V−O)/ ν_s(Mo−O), ν(V=O)/ ν(Mo=O)
			unassigned ? (689 (sh))	ν_{as}(Mo–O−Mo)
	24 h at RT	**451 (w, mb),** 689 (sh), **718 (w, mb), 842 (sh), 871 (st, s), 933 (m, s), 992 (sh)**	**{Mo$_{72}$V$_{30}$}**	ν_{as+s}(O−V−O), ν_s(V−O)/ ν_s(Mo−O), ν(V=O)/ ν(Mo=O)
			unassigned ? (689 (sh))	ν_{as}(Mo–O−Mo)
	24 h at 37 °C	**451 (w, mb), 717 (s, mb), 844 (sh), 872 (st, s), 934 (m, s), 993 (sh)**	**{Mo$_{72}$V$_{30}$}**	ν_{as+s}(O−V−O), ν_s(V−O)/ ν_s(Mo−O), ν(V=O)/ ν(Mo=O)

*H_2O shifts / cm^{-1}: 1650 – 3420 (st, b)

0.1 M sodium phosphate buffers, c(Mo$_{72}$V$_{30}$)= 0.15 mM

**pH 2**	fresh	451 (w, mb), 720 (w, b), 847 (st, s), 936 (m, s), 998 (sh)	$[Mo_7O_{24}]^{6-}$	ν_s(Mo–O−Mo), ν_{as}(Mo–O−Mo), ν_{as}(Mo=O), ν_s(Mo=O)
			$[HVO_4]^{2-}$	ν(V=O)
	24 h at RT	450 (w, mb), 500 (w, b), 718 (w, b), 838 (sh), 877 (st, s), 937 (m, s), 983 (sh)	$[Mo_7O_{24}]^{6-}$	ν_s(Mo–O−Mo), ν_{as}(Mo–O−Mo), ν_{as}(Mo=O), ν_s(Mo=O)
			$[HVO_4]^{2-}$	ν(V=O)
	24 h at 37 °C	**450 (w, mb), 716 (w, b), 842 (sh), 874 (st, s), 933 (m, s), 989 (sh)**	**{Mo$_{72}$V$_{30}$}**	ν_{as+s}(O−V−O), ν_s(V−O)/ ν_s(Mo−O), ν(V=O)/ ν(Mo=O)
**pH 3**	fresh	**453 (w, mb), 722 (w, b), 846 (sh), 876 (st, s), 935 (m, s), 993 (sh)**	**{Mo$_{72}$V$_{30}$}**	ν_{as+s}(O−V−O), ν_s(V−O)/ ν_s(Mo−O), ν(V=O)/ ν(Mo=O)
	24 h at RT	**450 (w, mb), 715 (w, mb), 842 (sh), 872 (st, s), 932 (m, s), 989 (sh)**	**{Mo$_{72}$V$_{30}$}**	ν_{as+s}(O−V−O), ν_s(V−O)/ ν_s(Mo−O), ν(V=O)/ ν(Mo=O)
	24 h at 37 °C	**451 (w, mb), 717 (w, mb), 840 (sh), 876 (st, s), 935 (m, s), 983 (sh)**	**{Mo$_{72}$V$_{30}$}**	ν_{as+s}(O−V−O), ν_s(V−O)/ ν_s(Mo−O), ν(V=O)/ ν(Mo=O)
**pH 4**	fresh	**450 (w, mb), 717 (w, mb), 841 (sh), 876 (st, s), 935 (m, s), 996 (sh),** 1054 (w, mb)	**{Mo$_{72}$V$_{30}$}**	ν_{as+s}(O−V−O), ν_s(V−O)/ ν_s(Mo−O), ν(V=O)/ ν(Mo=O)
			V_1 − V_5 isopolyvanadates	ν_{as}(V=O) bands between 900 – 1100 cm^{-1}
	24 h at RT	**450 (w, mb), 718 (w, mb), 846 (sh), 873 (st, s), 933 (m, s), 989 (sh)**	**{Mo$_{72}$V$_{30}$}**	ν_{as+s}(O−V−O), ν_s(V−O)/ ν_s(Mo−O), ν(V=O)/ ν(Mo=O)
	24 h at 37 °C	**450 (w, mb), 722 (w, mb), 842 (sh), 876 (st, s), 935 (m, s), 989 (sh)**	**{Mo$_{72}$V$_{30}$}**	ν_{as+s}(O−V−O), ν_s(V−O)/ ν_s(Mo−O), ν(V=O)/ ν(Mo=O)

**pH 5**	fresh	**450 (w, mb), 718 (w, b), 842 (sh), 875 (st, s), 935 (m, s), 989 (sh)**	**$\{Mo_{72}V_{30}\}$**	ν_{as+s}(O–V–O), ν_s(V–O)/ ν_s(Mo–O), ν(V=O)/ ν(Mo=O)
	24 h at RT	**451 (w, mb), 716 (w, mb), 844 (sh), 872 (st, s), 933 (m, s), 989 (sh)**	**$\{Mo_{72}V_{30}\}$**	ν_{as+s}(O–V–O), ν_s(V–O)/ ν_s(Mo–O), ν(V=O)/ ν(Mo=O)
	24 h at 37 °C	455 (w, mb), 718 (w, mb), 844 (sh), 873 (st, s), 933 (m, s), 979 (sh)	β-$[Mo_8O_{26}]^{4-}$	ν_s(Mo–O–Mo), ν_{as}(Mo–O–Mo), ν_{as}(Mo=O), ν_s(Mo=O)
			$[Mo_7O_{24}]^{6-}$	ν_s(Mo–O–Mo), ν_{as}(Mo–O–Mo), ν_{as}(Mo=O)
			$[HVO_4]^{2-}$ or $[V_2O_7]^{4-}$	ν_s(V–O)
**pH 6**	fresh	453 (w, mb), 716 (w, mb), 838 (sh), 872 (st, s), 934 (m, s), 977 (sh)	β-$[Mo_8O_{26}]^{4-}$	ν_s(Mo–O–Mo), ν_{as}(Mo–O–Mo), ν_{as}(Mo=O), ν_s(Mo=O)
			$[Mo_7O_{24}]^{6-}$	ν_{as}(Mo=O)
			$[HVO_4]^{2-}$ or $[V_2O_7]^{4-}$	ν_s(V–O)
	24 h at RT	450 (w, mb), 718 (w, mb), 842 (sh), 873 (st, s), 933 (m, s), 987 (sh)	$[Mo_7O_{24}]^{6-}$	ν_s(Mo–O–Mo), ν_{as}(Mo–O–Mo), ν_{as}(Mo=O), ν_s(Mo=O)
			$[HVO_4]^{2-}$	ν(V=O)
	24 h at 37 °C	449 (w, mb), 715 (w, mb), 842 (sh), 873 (st, s), 933 (m, s), 979 (sh)	β-$[Mo_8O_{26}]^{4-}$	ν_s(Mo–O–Mo), ν_{as}(Mo–O–Mo), ν_{as}(Mo=O), ν_s(Mo=O)
			$[Mo_7O_{24}]^{6-}$	ν_{as}(Mo=O)
			$[HVO_4]^{2-}$ or $[V_2O_7]^{4-}$	ν_s(V–O)
**pH 7**	fresh	449 (w, mb), 718 (w, b), 844 (sh), 873 (st, s), 933 (m, s), 987 (w, s)	$[Mo_7O_{24}]^{6-}$	ν_s(Mo–O–Mo), ν_{as}(Mo–O–Mo), ν_{as}(Mo=O), ν_s(Mo=O)
			$[HVO_4]^{2-}$	ν(V=O)
	24 h at RT	846 (sh), 873 (st, s), 930 (m, s), 949 (sh), 985 (w, mb)	$[Mo_7O_{24}]^{6-}$	ν_{as}(Mo=O)
			$[Mo_6O_{19}]^{2-}$	ν_{as+s}(Mo–O_t)
			$[V_4O_{12}]^{4-}$	ν_{as+s}(V–O)
	24 h at 37 °C	875 (w, mb), 891 (w, mb), 986 (w, s)	$V_1 - V_5$ isopolyvanadates	ν_{as}(V=O) bands between 900 – 1100 cm^{-1}
			$[MoO_4]^{2-}$	ν_s(Mo–O)
**pH 8**	fresh	718 (w, b), 838 (sh), 871 (st, s), 932 (m, s), 987 (m, s)	$[Mo_7O_{24}]^{6-}$	ν_{as}(Mo=O), ν_s(Mo=O)
			$[HVO_4]^{2-}$	ν(V=O)
	24 h at RT	879 (w, b), 892 (w, s), 986 (w, s)	$V_1 - V_5$ isopolyvanadates	ν_{as}(V=O) bands between 900 – 1100 cm^{-1}
			$[MoO_4]^{2-}$	ν_s(Mo–O)
	24 h at 37 °C	547 (m, b), 893 (w, s), 988 (w, s)	$[Mo_6O_{19}]^{2-}$	ν_{as}(Mo=O), ν_s(Mo=O)
			$V_1 - V_5$ isopolyvanadates	ν_{as}(V=O) bands between 900 – 1100 cm^{-1}
*sodium phosphate buffers shift / cm^{-1}: 1075 (w, mb), 1651 (m, b), 2084 (w, b), 2616 (w, b), 3426 (st, b)				
**0.1 M acetic acid – sodium acetate buffers, c($Mo_{72}V_{30}$)= 0.20 mM**				
**pH 4**	fresh	453 (w, mb), 722 (w, mb), 842 (sh), 875 (st, s), 931 (m, s), 979 (w, s)	$[V_{10}O_{28}]^{6-}$ or $[V_9MoO_{28}]^{5-}$	ν_{as+s}(O–V–O), ν_s(V–O)/ ν_s(Mo–O, ν(V=O)/ ν(Mo=O)
			$[HVO_4]^{2-}$ or $[V_2O_7]^{4-}$	ν_s(V–O)

			$[Mo_7O_{24}]^{6-}$	ν_{as}(Mo=O)
			unassigned ? (722 (sh))	ν_{as}(Mo–O–Mo)
	24 h at RT	**452 (w, mb),** 695 (sh), **717 (w, mb), 842 (sh), 872 (st, s), 932 (m, s), 989 (sh)**	**$\{Mo_{72}V_{30}\}$**	ν_{as+s}(O–V–O), ν_s(V–O)/ ν_s(Mo–O), ν(V=O)/ ν(Mo=O)
			unassigned ? (695 (sh))	ν_{as}(Mo–O–Mo)
	24 h at 37 °C	452 (w, mb), 726 (sh), 842 (sh), 874 (st, s), 931 (m, s), 978 (w, s)	$[V_{10}O_{28}]^{6-}$ or $[V_9MoO_{28}]^{5-}$	ν_{as+s}(O–V–O), ν_s(V–O)/ ν_s(Mo–O), ν(V=O)/ ν(Mo=O)
			$[HVO_4]^{2-}$ or $[V_2O_7]^{4-}$	ν_s(V–O)
			$[Mo_7O_{24}]^{6-}$	ν_{as}(Mo=O)
**pH 5**	fresh	455 (w, mb), 720 (w, mb), 780 (sh), 840 (sh), 873 (st, s), 932 (m, s), 977 (sh)	$[V_{10}O_{28}]^{6-}$ or $[V_9MoO_{28}]^{5-}$	ν_{as+s}(O–V–O), ν_s(V–O)/ ν_s(Mo–O), ν(V=O)/ ν(Mo=O)
			$[HVO_4]^{2-}$ or $[V_2O_7]^{4-}$	ν_s(V–O)
			$[Mo_7O_{24}]^{6-}$	ν_{as}(Mo=O)
	24 h at RT	**449 (w, mb), 718 (w, mb), 842 (sh), 875 (st, s), 931 (m, s), 989 (sh)**	**$\{Mo_{72}V_{30}\}$**	ν_{as+s}(O–V–O), ν_s(V–O)/ ν_s(Mo–O), ν(V=O)/ ν(Mo=O)
	24 h at 37 °C	454 (w, mb), 716 (w, b), 780 (sh), 844 (sh), 875 (st, s), 926 (m, s), 977 (sh)	$[V_{10}O_{28}]^{6-}$ or $[V_9MoO_{28}]^{5-}$	ν_{as+s}(O–V–O), ν_s(V–O)/ ν_s(Mo–O), ν(V=O)/ ν(Mo=O)
			$[HVO_4]^{2-}$ or $[V_2O_7]^{4-}$	ν_s(V–O)
			$V_1 - V_5$ isopolyvanadates	ν_{as}(V=O) bands between 900 – 1100 cm^{-1}
**pH 5.5**	fresh	453 (w, mb), 717 (w, mb), 774 (sh), 846 (sh), 873 (st, s), 930 (m, s), 979 (sh)	$[V_{10}O_{28}]^{6-}$ or $[V_9MoO_{28}]^{5-}$	ν_{as+s}(O–V–O), ν_s(V–O)/ ν_s(Mo–O), ν(V=O)/ ν(Mo=O)
			$V_1 - V_5$ isopolyvanadates	ν_{as}(V=O) bands between 900 – 1100 cm^{-1}
			$[Mo_7O_{24}]^{6-}$	ν_{as}(Mo=O)
	24 h at RT	450 (w, mb), 718 (w, mb), 774 (sh), 844 (sh), 873 (st, s), 931 (m, s), 973 (sh)	$[V_{10}O_{28}]^{6-}$ or $[V_9MoO_{28}]^{5-}$	ν_{as+s}(O–V–O), ν_s(V–O)/ ν_s(Mo–O), ν(V=O)/ ν(Mo=O)
			$V_1 - V_5$ isopolyvanadates	ν_{as}(V=O) bands between 900 – 1100 cm^{-1}
			$[Mo_7O_{24}]^{6-}$	ν_{as}(Mo=O)
	24 h at 37 °C	450 (w, mb), 718 (w, mb), 780 (sh), 844 (sh), 872 (st, s), 931 (m, s), 975 (sh)	$[V_{10}O_{28}]^{6-}$ or $[V_9MoO_{28}]^{5-}$	ν_{as+s}(O–V–O), ν_s(V–O)/ ν_s(Mo–O), ν(V=O)/ ν(Mo=O)
			$V_1 - V_5$ isopolyvanadates	ν_{as}(V=O) bands between 900– 1100 cm^{-1}
			$[Mo_7O_{24}]^{6-}$	ν_{as}(Mo=O)

*acetic acid – sodium acetate buffers shifts / cm^{-1}: 1352 (w, mb), 1411 (w, mb), 1696 (m ,b), 2936 (w, s), 3441 (st, b)

0.1 M Tris – HCl buffers, c($Mo_{72}V_{30}$)= 0.15 mM

**pH 7**	Fresh	718 (w, b), 760 (w, s), 840 (sh), 871 (st, s), 933 (m, s), 979 (sh)	$[Mo_7O_{24}]^{6-}$	ν_{as}(Mo–O–Mo), ν_{as}(Mo=O), ν_s(Mo=O)
			$HV_1 - V_6$ isopolyvanadates	ν_{as}(V=O) bands between 800– 1000 cm^{-1}
	24 h at RT	760 (w, s), 838 (sh), 871 (st, s), 932 (m, s), 975 (sh)	$[Mo_7O_{24}]^{6-}$	ν_{as}(Mo–O–Mo), ν_{as}(Mo=O), ν_s(Mo=O)
			$HV_1 - V_6$ isopolyvanadates	ν_{as}(V=O) bands between 800– 1000 cm^{-1}

	24 h at 37 °C	760 (w, s), 778 (w, s), 842 (sh), 871 (st, s), 933 (m, s), 979 (sh)	$[Mo_7O_{24}]^{6-}$	ν_{as}(Mo=O), ν_s(Mo=O)
			$HV_1 - V_6$ isopolyvanadates	ν_{as}(V=O) bands between 800–1000 cm^{-1}
			Possible $[V_{10}O_{28}]^{6-}$	ν_{as+s}(O−V−O)
**pH 8**	Fresh	764 (w, mb), 840 (sh), 870 (st, s), 927 (m, mb), 979 (sh)	$[Mo_7O_{24}]^{6-}$	ν_{as}(Mo=O), ν_s(Mo=O)
			$HV_1 - V_6$ isopolyvanadates	ν_{as}(V=O) bands between 800–1000 cm^{-1}
	24 h at RT	762 (w, mb), 893 (w, s)	$[MoO_4]^{2-}$	ν_s(Mo–O)
			$V_3 - V_6$ isopolyvanadates	ν_{as}(V=O) bands between 800–900 cm^{-1}
	24 h at 37 °C	760 (w, mb), 894 (w, s)	$[MoO_4]^{2-}$	ν_s(Mo–O)
			$V_3 - V_6$ isopolyvanadates	ν_{as}(V=O) bands between 800–900 cm^{-1}

*Tris – HCl buffers shifts / cm^{-1}: 1064 (w, mb), 1468 (w, s), 1732 (m, b), 2604 (w, b), 2898 (m, s), 2961 (m, s), 3435 (st, b)

0.1 M HEPES buffers, c($Mo_{72}V_{30}$)= 0.15 mM

**pH 7**	Fresh	732 (sh), 873 (st, s), 936 (w, mb), 999 (w, mb)	$[Mo_7O_{24}]^{6-}$	ν_{as}(Mo=O)
			$[HVO_4]^{2-}$ or $[V_2O_7]^{4-}$	ν_s(V–O)
			$V_1 - V_5$ isopolyvanadates	ν_{as}(V=O) bands between 900–1100 cm^{-1}
	24 h at RT	732 (sh), 874 (st, s), 936 (w, mb), 999 (w, mb)	$[Mo_7O_{24}]^{6-}$	ν_{as}(Mo=O)
			$[HVO_4]^{2-}$ or $[V_2O_7]^{4-}$	ν_s(V–O)
			$V_1 - V_5$ isopolyvanadates	ν_{as}(V=O) bands between 900–1100 cm^{-1}
	24 h at 37 °C	734 (sh), 874 (st, s), 936 (w, mb), 999 (w, mb)	$[Mo_7O_{24}]^{6-}$	ν_{as}(Mo=O)
			$[HVO_4]^{2-}$ or $[V_2O_7]^{4-}$	ν_s(V–O)
			$V_1 - V_5$ isopolyvanadates	ν_{as}(V=O) bands between 900–1100 cm^{-1}
**pH 8**	fresh	872 (st, s), 933 (m, s)	$[Mo_7O_{24}]^{6-}$	ν_{as}(Mo=O)
			$[HVO_4]^{2-}$ or $[V_2O_7]^{4-}$	ν_s(V–O)
	24 h at RT	882 (w, mb), 931 (w, b)	$[Mo_7O_{24}]^{6-}$	ν_{as}(Mo=O)
			$HV_1 - V_6$ isopolyvanadates	ν_{as}(V=O) bands between 800–1000 cm^{-1}
	24 h at 37 °C	893 (w, s), 1011 (m, s)	$[MoO_4]^{2-}$	ν_s(Mo–O)
			$V_1 - V_5$ isopolyvanadates	ν_{as}(V=O) bands between 900–1100 cm^{-1}

*HEPES buffers shifts / cm-^{1}: 765 (w, mb), 840 (sh), 1035 (m, s), 1195 (w, b), 1305 (w, mb), 1429 (sh), 1451 (w, mb), 1464 (w, mb), 1749 (m, b), 2841 (m, mb), 2944 (sh), 2970 (st, s), 3480 (st, b)

*Intensity: st – strong, m – medium, w – weak. Shape: b – broad, mb – medium broad, s – sharp, sh – shoulder.

5.4. {$Mo_{72}V_{30}$} rearrangement schemes

5.4.1. {$Mo_{72}V_{30}$} rearrangement schemes in H_2O (pH 1 – 8)

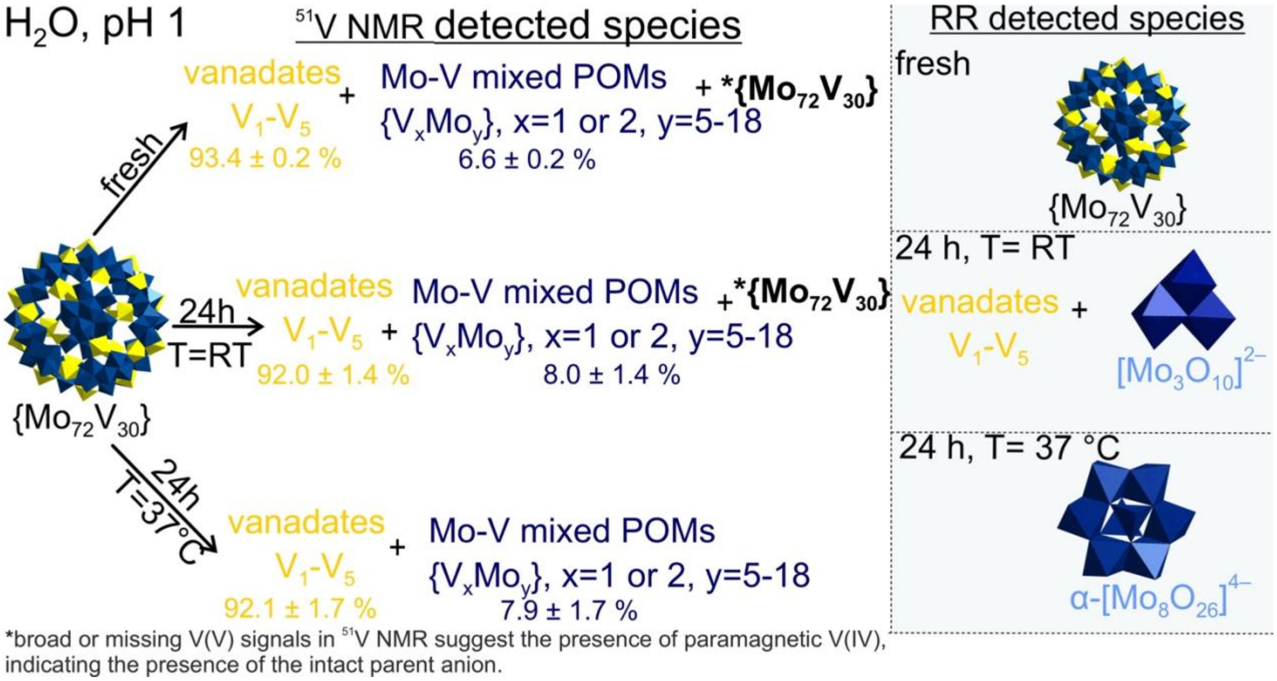

Fig. S19. Rearrangement scheme of {$Mo_{72}V_{30}$} in H_2O (pH 1).

The scheme is based on the collected and analyzed ^{51}V NMR and resonance Raman data (gray field). The structures of all POMs are shown in **Figures S1** and **S2**. **Tables S8 – S10** give all ^{51}V NMR chemical shifts and percentages of species detectable by ^{51}V NMR (**Table S11**) formed and **Table S15** gives resonance Raman shifts, and their assignation based on literature data (**Tables S5 – S6**). Only species with a percentage > 10 % are shown separately; other species are separated by the type of addenda metals in the structure. Color code: {MoO_6}, blue; {VO_6}, yellow.

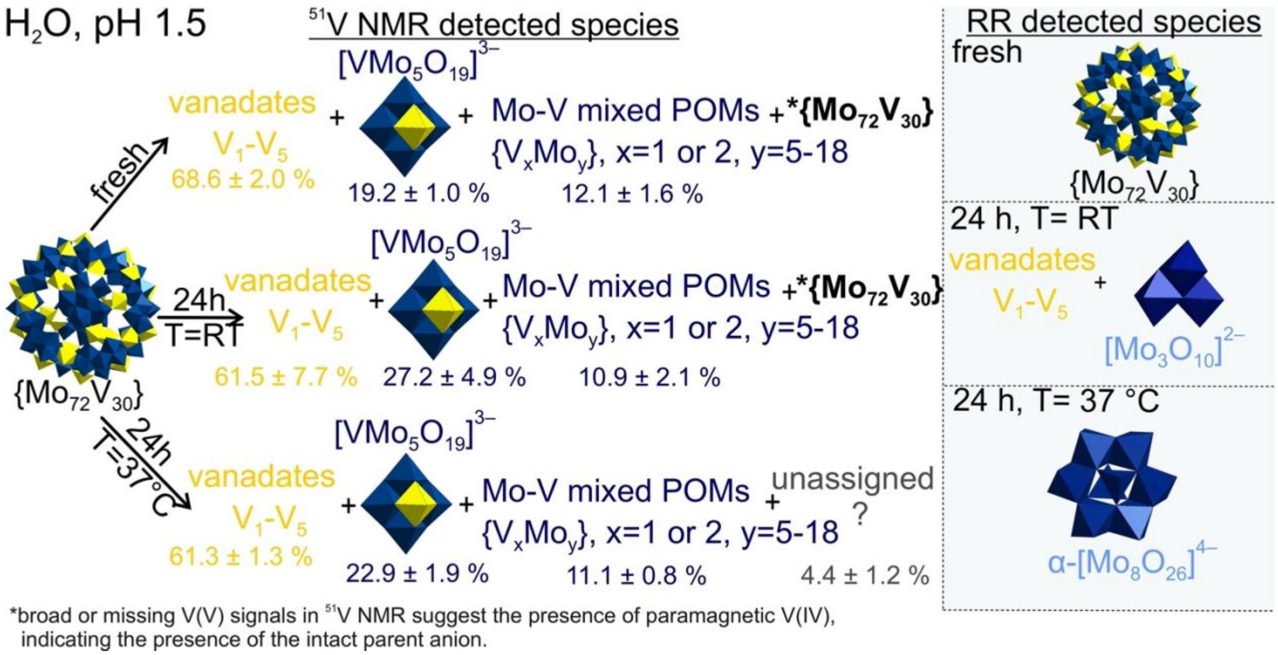

Fig. S20. Rearrangement scheme of {$Mo_{72}V_{30}$} in H_2O (pH 1.5).

The scheme is based on the collected and analyzed ^{51}V-NMR and resonance Raman data (gray field). The structures of all POMs are shown in **Figures S1** and **S2**. **Tables S8 – S10** give all ^{51}V NMR chemical shifts and percentages of species detectable by ^{51}V NMR (**Table S11**) formed and **Table S15** gives resonance Raman shifts, and their assignation based on literature data (**Tables S5 – S6**). Only species with a percentage > 10 % are shown separately; other species are separated by the type of addenda metals in the structure. Color code: {MoO_6}, blue; {VO_6}, yellow.

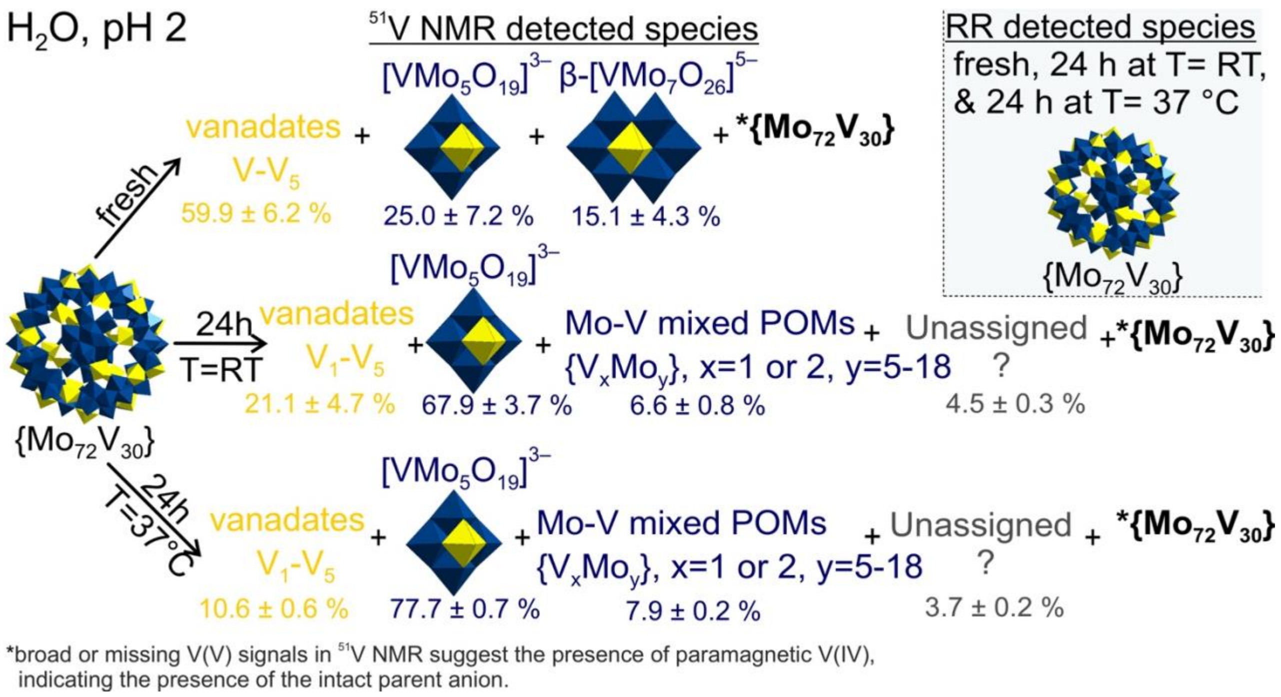

Fig. S21. Rearrangement scheme of {$Mo_{72}V_{30}$} in H_2O (pH 2).

The scheme is based on the collected and analyzed ^{51}V NMR and resonance Raman data (gray field). The structures of all POMs are shown in **Figures S1** and **S2**. **Tables S8 – S10** give all ^{51}V NMR chemical shifts and percentages of species detectable by ^{51}V NMR (**Table S11**) formed and **Table S15** gives resonance Raman shifts, and their assignation based on literature data (**Tables S5 – S6**). Only species with a percentage > 10 % are shown separately; other species are separated by the type of addenda metals in the structure. Color code: {MoO_6}, blue; {VO_6}, yellow.

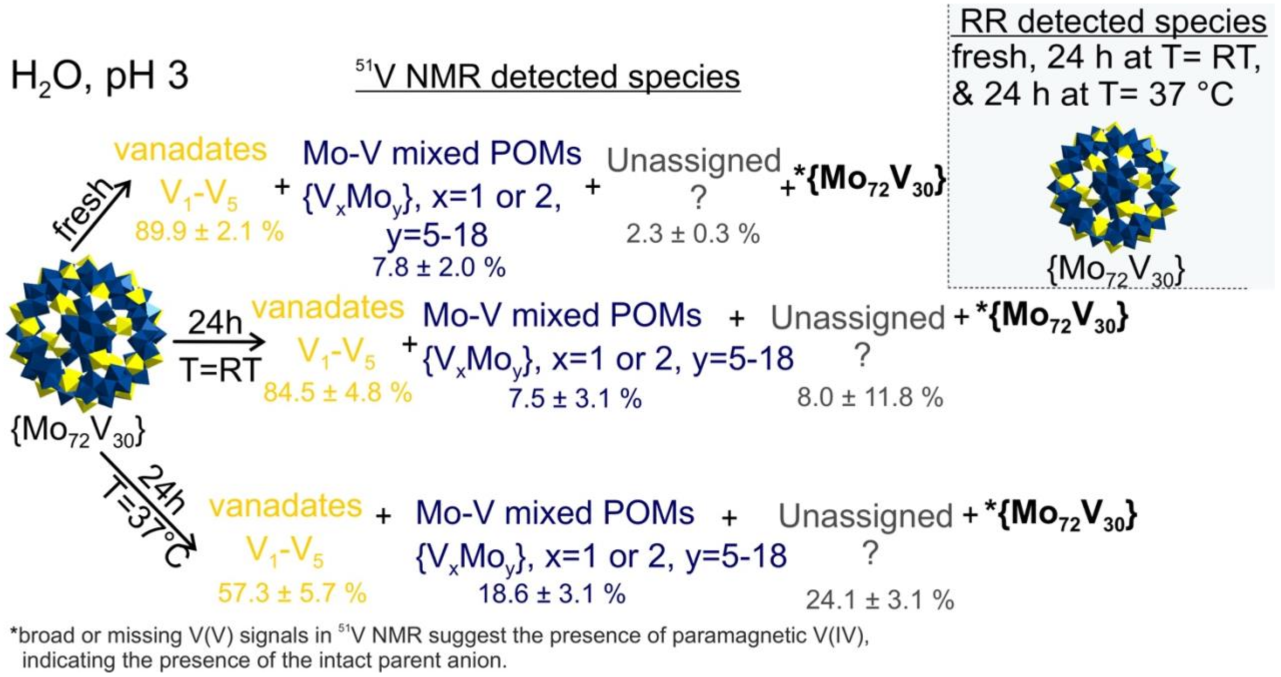

Fig. S22. Rearrangement scheme of {$Mo_{72}V_{30}$} in H_2O (pH 3).

The scheme is based on the collected and analyzed ^{51}V NMR and resonance Raman data (gray field). The structures of all POMs are shown in **Figures S1** and **S2**. **Tables S8 – S10** give all ^{51}V NMR chemical shifts and percentages of species detectable by ^{51}V NMR (**Table S11**) formed and **Table S15** gives resonance Raman shifts, and their assignation based on literature data (**Tables S5 – S6**). Only species with a percentage > 10 % are shown separately; other species are separated by the type of addenda metals in the structure. Color code: {MoO_6}, blue; {VO_6}, yellow.

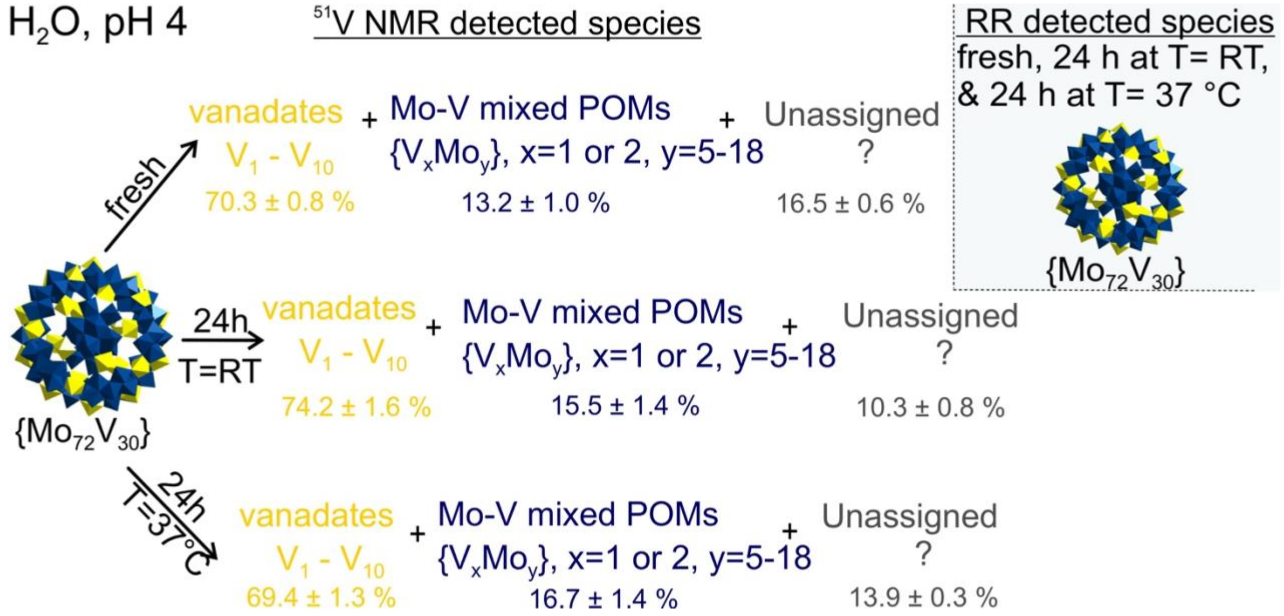

Fig. S23. Rearrangement scheme of $\{Mo_{72}V_{30}\}$ in H_2O (pH 4).

The scheme is based on the collected and analyzed ^{51}V NMR and resonance Raman data (gray field). The structures of all POMs are shown in **Figures S1** and **S2**. **Tables S8 – S10** give all ^{51}V NMR chemical shifts and percentages of species detectable by ^{51}V NMR (**Table S11**) formed and **Table S15** gives resonance Raman shifts, and their assignation based on literature data (**Tables S5 – S6**). Only species with a percentage > 10 % are shown separately; other species are separated by the type of addenda metals in the structure. Color code: $\{MoO_6\}$, blue; $\{VO_6\}$, yellow.

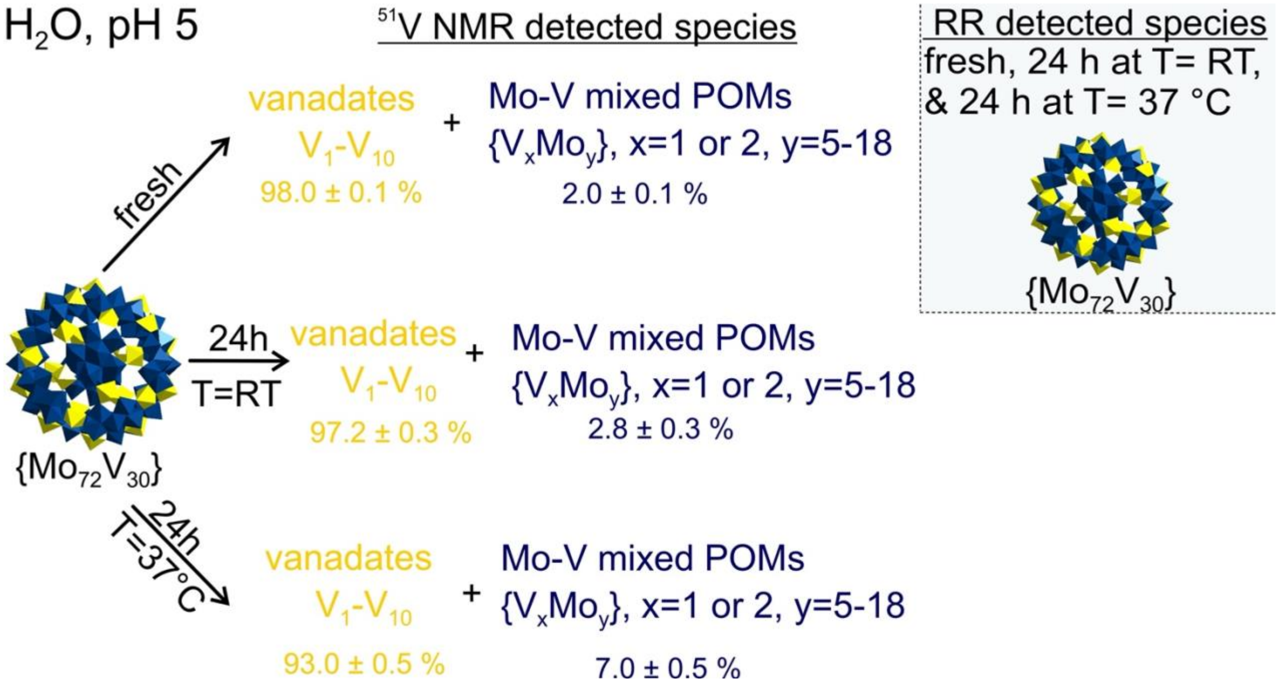

Fig. S24. Rearrangement scheme of {$Mo_{72}V_{30}$} in H_2O (pH 5).

The scheme is based on the collected and analyzed ^{51}V NMR and resonance Raman data (gray field). The structures of all POMs are shown in **Figures S1** and **S2**. **Tables S8 – S10** give all ^{51}V NMR chemical shifts and percentages of species detectable by ^{51}V NMR (**Table S11**) formed and **Table S15** gives resonance Raman shifts, and their assignation based on literature data (**Tables S5 – S6**). Only species with a percentage > 10 % are shown separately; other species are separated by the type of addenda metals in the structure. Color code: {MoO_6}, blue; {VO_6}, yellow.

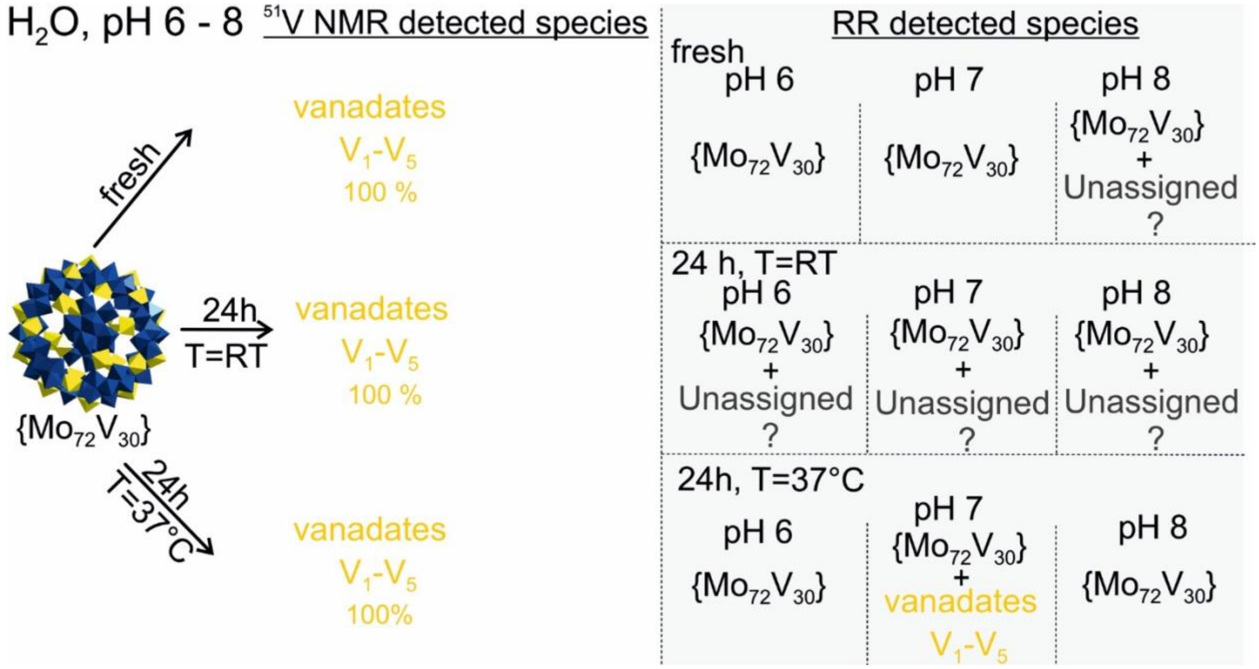

Fig. S25. Rearrangement scheme of $\{Mo_{72}V_{30}\}$ in H_2O (pH 6 – 8).

The scheme is based on the collected and analyzed ^{51}V NMR and resonance Raman data (gray field). The structures of all POMs are shown in **Figures S1** and **S2**. **Tables S8 – S10** give all ^{51}V NMR chemical shifts and percentages of species detectable by ^{51}V NMR (**Table S11**) formed and **Table S15** gives resonance Raman shifts, and their assignation based on literature data (**Tables S5 – S6**). Only species with a percentage > 10 % are shown separately; other species are separated by the type of addenda metals in the structure. Color code: $\{MoO_6\}$, blue; $\{VO_6\}$, yellow.

5.4.2. $\{Mo_{72}V_{30}\}$ rearrangement schemes in 0.1 M sodium phosphate buffers.

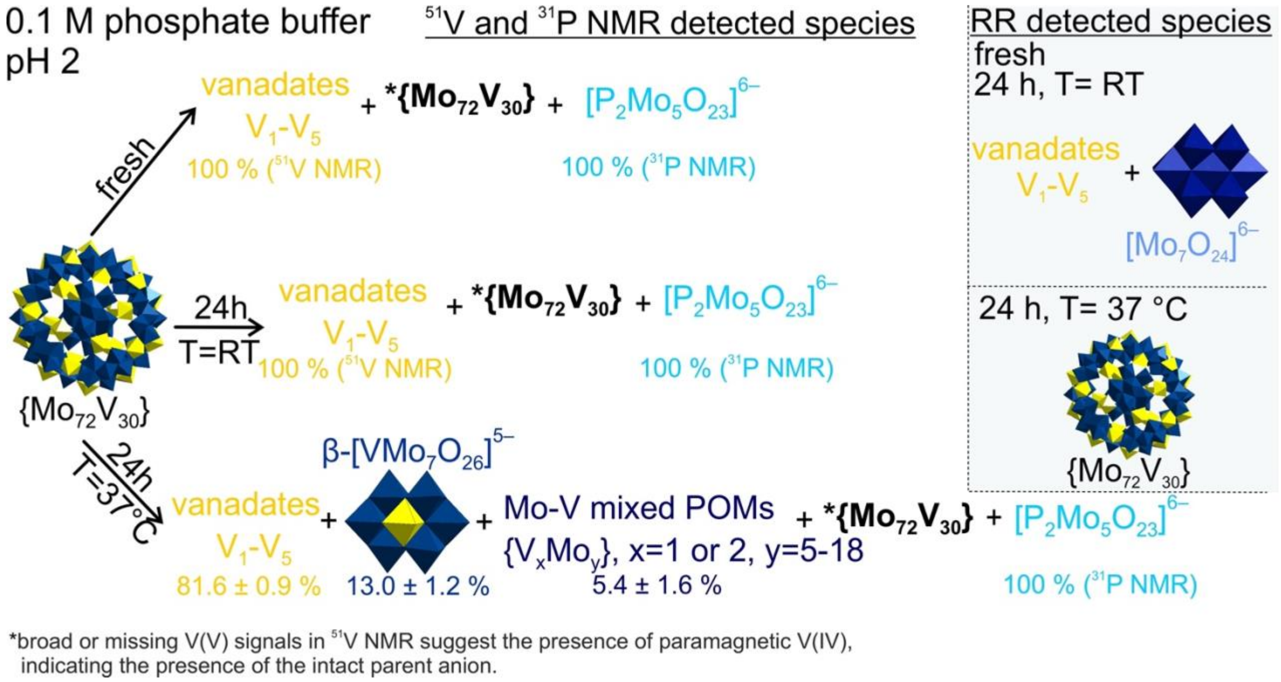

Fig. S26. Rearrangement scheme of $\{Mo_{72}V_{30}\}$ in 0.1 M sodium phosphate buffer (pH 2). The scheme is based on the collected and analyzed ^{51}V and ^{31}P NMR and resonance Raman data (gray field). The structures of all POMs are shown in **Figures S1** and **S2**. **Tables S8 – S10** give all ^{51}V NMR chemical shifts and percentages of species detectable by ^{51}V NMR (**Table S12**) formed and **Table S15** gives resonance Raman shifts, and their assignation based on literature data (**Tables S5 – S6**). Only species with a percentage > 10 % are shown separately; other species are separated by the type of addenda metals in the structure. Color code: $\{MoO_6\}$, blue; $\{VO_6\}$, yellow.

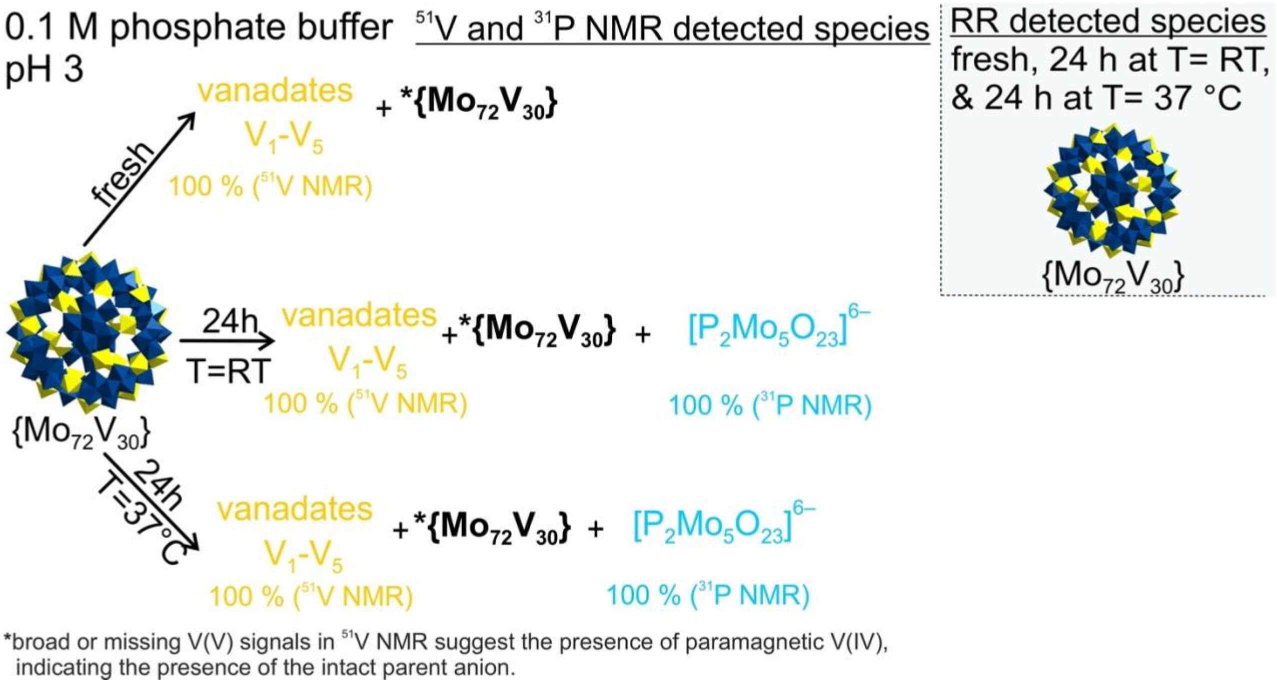

Fig. S27. Rearrangement scheme of $\{Mo_{72}V_{30}\}$ in 0.1 M sodium phosphate buffer (pH 3). The scheme is based on the collected and analyzed ^{51}V and ^{31}P NMR and resonance Raman data (gray field). The structures of all POMs are shown in **Figures S1** and **S2**. **Tables S8 – S10** give all ^{51}V NMR chemical shifts and percentages of species detectable by ^{51}V NMR (**Table S12**) formed and **Table S15** gives resonance Raman shifts, and their assignation based on literature data (**Tables S5 – S6**). Only species with a percentage > 10 % are shown separately; other species are separated by the type of addenda metals in the structure. Color code: $\{MoO_6\}$, blue; $\{VO_6\}$, yellow.

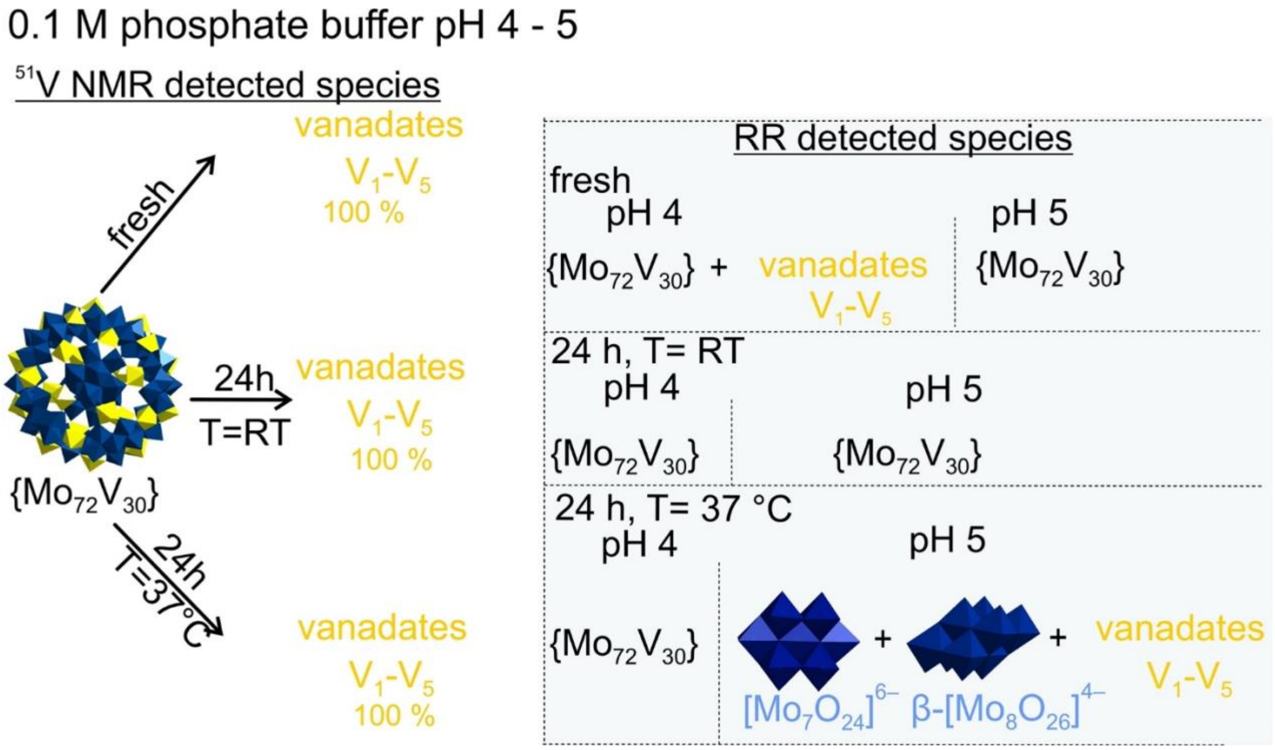

Fig. S28. Rearrangement scheme of $\{Mo_{72}V_{30}\}$ in 0.1 M sodium phosphate buffers (pH 4 and pH 5).

The scheme is based on the collected and analyzed ^{51}V-NMR and resonance Raman data (gray field). The structures of all POMs are shown in **Figures S1** and **S2**. **Tables S8 – S10** give all ^{51}V NMR chemical shifts and percentages of species detectable by ^{51}V NMR (**Table S12**) formed and **Table S15** gives resonance Raman shifts, and their assignation based on literature data (**Tables S5 – S6**). Only species with a percentage > 10 % are shown separately; other species are separated by the type of addenda metals in the structure. Color code: $\{MoO_6\}$, blue; $\{VO_6\}$, yellow.

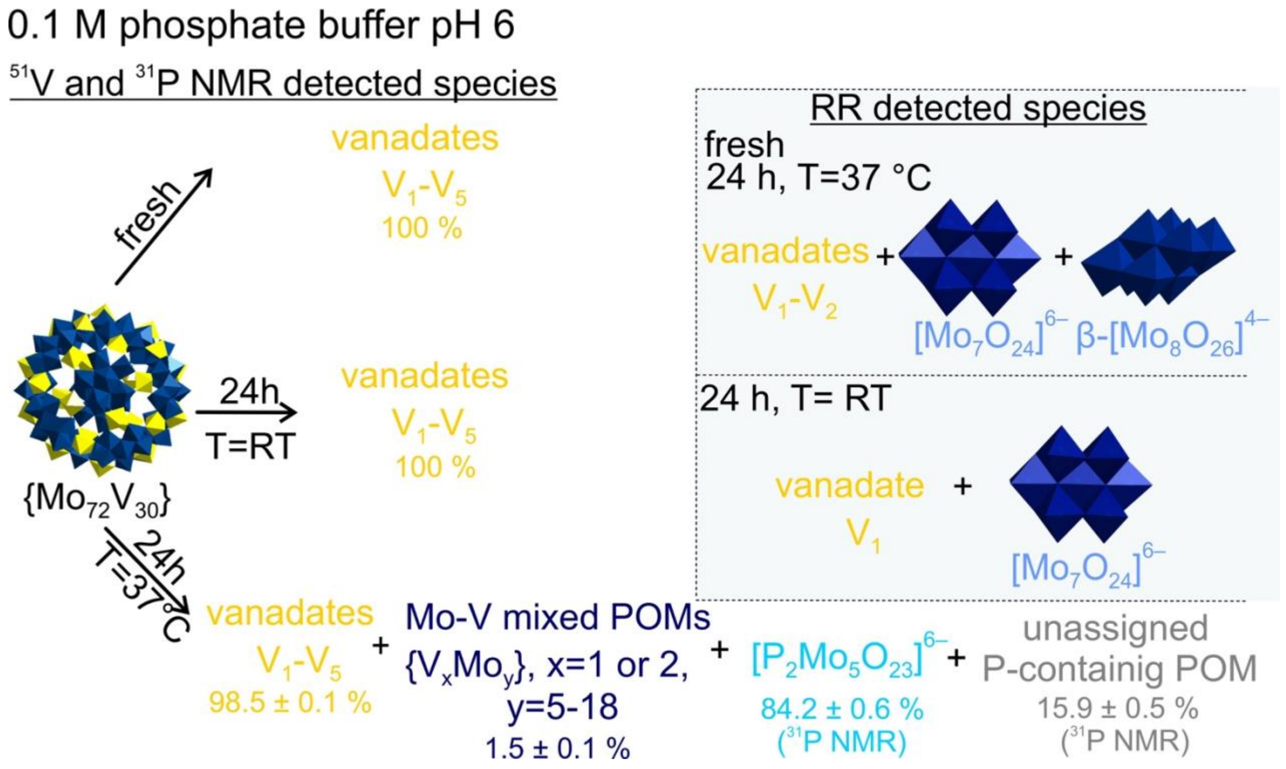

Fig. S29. Rearrangement scheme of $\{Mo_{72}V_{30}\}$ in 0.1 M sodium phosphate buffer (pH 6). The scheme is based on the collected and analyzed ^{51}V-NMR and resonance Raman data (gray field). The structures of all POMs are shown in **Figures S1** and **S2**. **Tables S8 – S10** give all ^{51}V NMR chemical shifts and percentages of species detectable by ^{51}V NMR (**Table S12**) formed and **Table S15** gives resonance Raman shifts, and their assignation based on literature data (**Tables S5 – S6**). Only species with a percentage > 10 % are shown separately; other species are separated by the type of addenda metals in the structure. Color code: $\{MoO_6\}$, blue; $\{VO_6\}$, yellow.

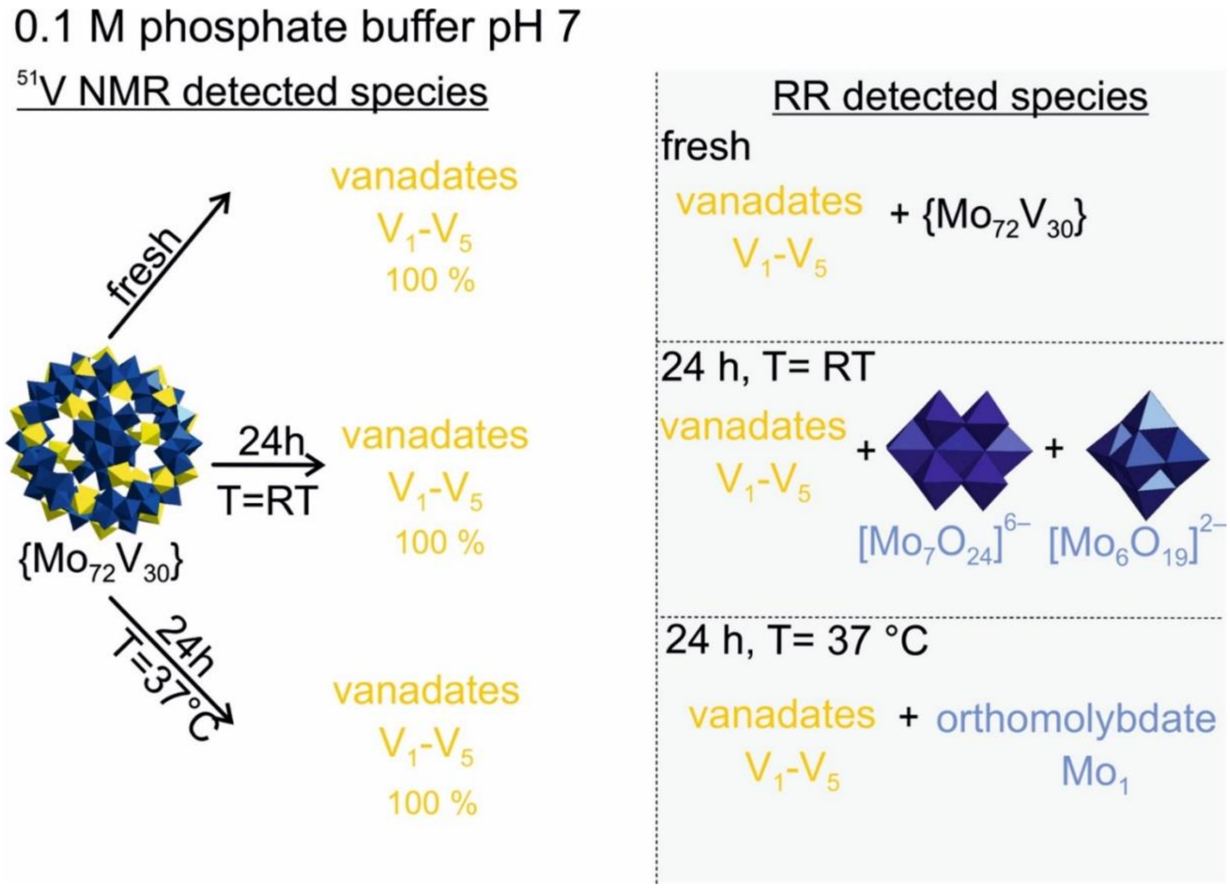

Fig. S30. Rearrangement scheme of $\{Mo_{72}V_{30}\}$ in 0.1 M sodium phosphate buffer (pH 7). The scheme is based on the collected and analyzed ^{51}V NMR and resonance Raman data (gray field). The structures of all POMs are shown in **Figures S1** and **S2**. **Tables S8 – S10** give all ^{51}V NMR chemical shifts and percentages of species detectable by ^{51}V NMR (**Table S12**) formed and **Table S15** gives resonance Raman shifts, and their assignation based on literature data (**Tables S5 – S6**). Only species with a percentage > 10 % are shown separately; other species are separated by the type of addenda metals in the structure. Color code: $\{MoO_6\}$, blue; $\{VO_6\}$, yellow.

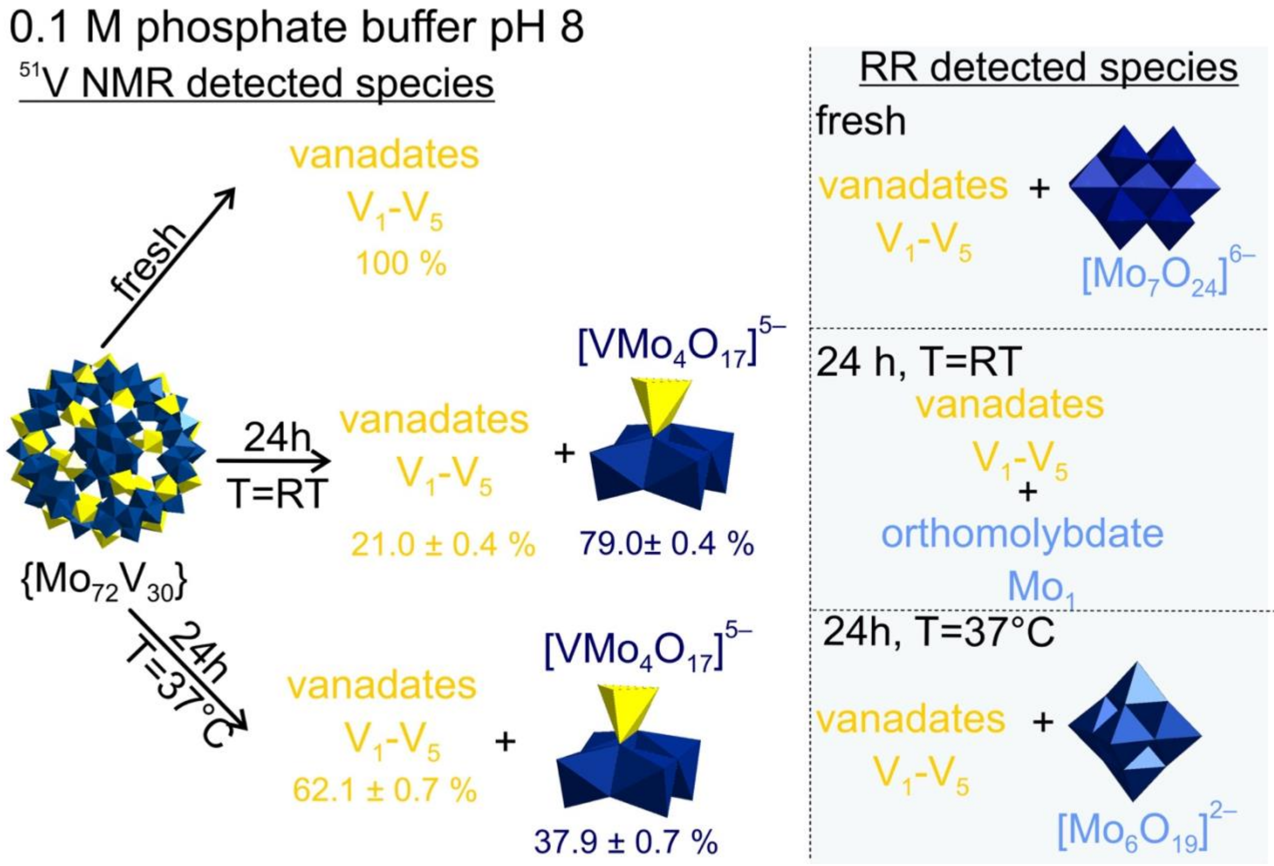

Fig. S31. Rearrangement scheme of {$Mo_{72}V_{30}$} in 0.1 M sodium phosphate buffer (pH 8). The scheme is based on the collected and analyzed ^{51}V-NMR and resonance Raman data (gray field). The structures of all POMs are shown in **Figures S1** and **S2**. **Tables S8 – S10** give all ^{51}V NMR chemical shifts and percentages of species detectable by ^{51}V NMR (**Table S12**) formed and **Table S15** gives resonance Raman shifts, and their assignation based on literature data (**Tables S5 – S6**). Only species with a percentage > 10 % are shown separately; other species are separated by the type of addenda metals in the structure. Color code: {MoO_6}, blue; {VO_6}, yellow.

5.4.3. {$Mo_{72}V_{30}$} rearrangement schemes in 0.1 M acetic acid – sodium acetate buffers

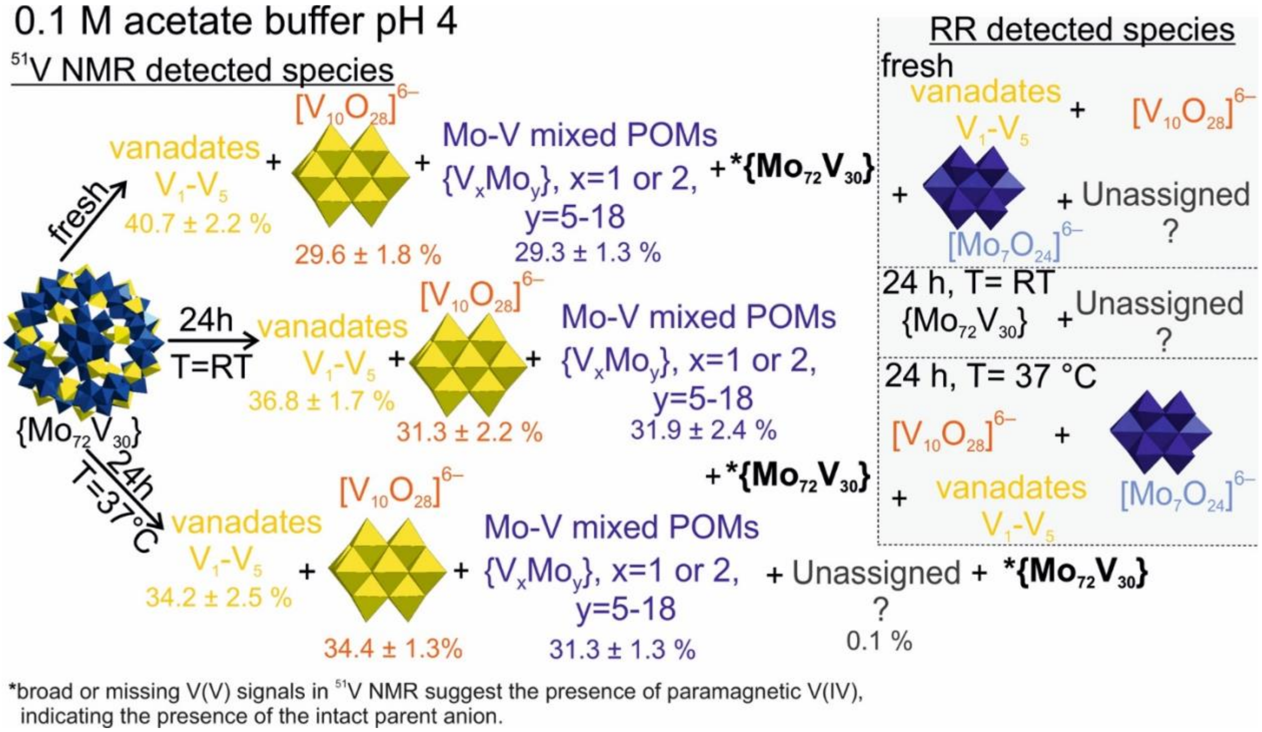

Fig. S32. Rearrangement scheme of {$Mo_{72}V_{30}$} in 0.1 M sodium acetate – acetic acid buffer (pH 4).

The scheme is based on the collected and analyzed ^{51}V-NMR and resonance Raman data (gray field). The structures of all POMs are shown in **Figures S1** and **S2**. **Tables S8 – S10** give all ^{51}V NMR chemical shifts and percentages of species detectable by ^{51}V NMR (**Table S13**) formed and **Table S15** gives resonance Raman shifts, and their assignation based on literature data (**Tables S5 – S6**). Only species with a percentage > 10 % are shown separately; other species are separated by the type of addenda metals in the structure. Color code: {MoO_6}, blue; {VO_6}, yellow.

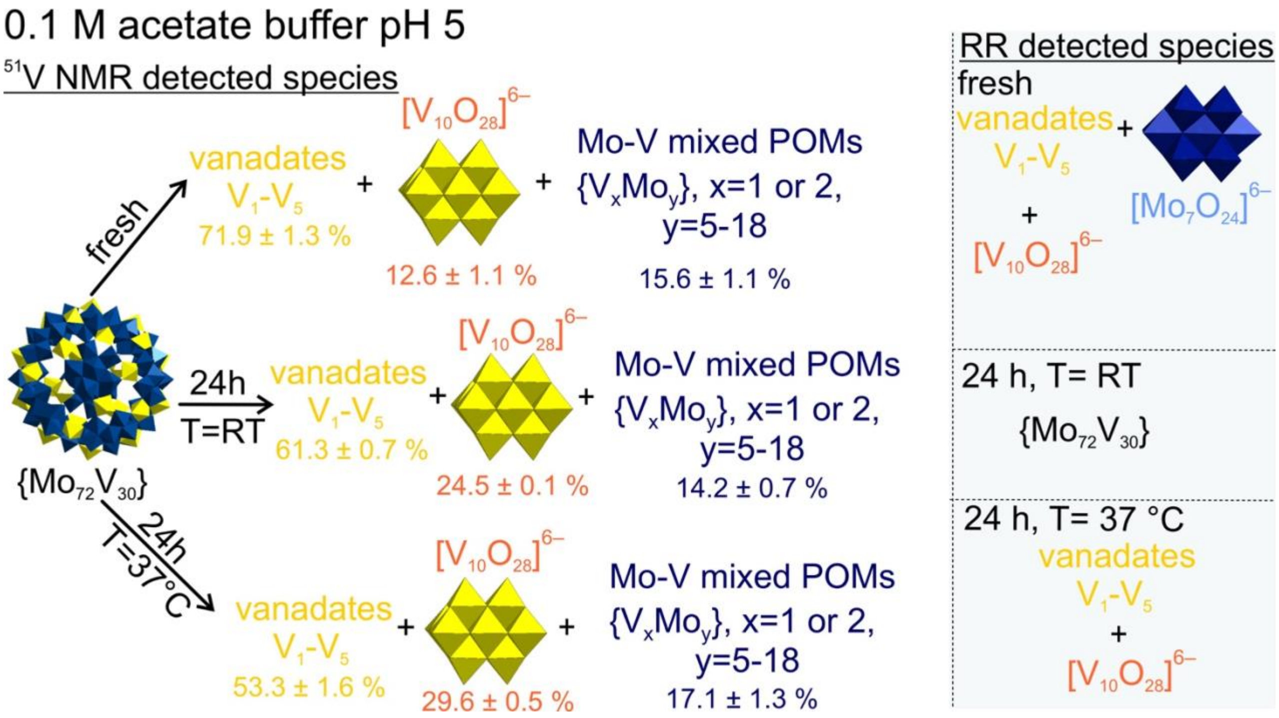

Fig. S33. Rearrangement scheme of {$Mo_{72}V_{30}$} in 0.1 M sodium acetate – acetic acid buffer (pH 5).

The scheme is based on the collected and analyzed ^{51}V NMR and resonance Raman data (gray field). The structures of all POMs are shown in **Figures S1** and **S2**. **Tables S8 – S10** give all ^{51}V NMR chemical shifts and percentages of species detectable by ^{51}V NMR (**Table S13**) formed and **Table S15** gives resonance Raman shifts, and their assignation based on literature data (**Tables S5 – S6**). Only species with a percentage > 10 % are shown separately; other species are separated by the type of addenda metals in the structure. Color code: {MoO_6}, blue; {VO_6}, yellow.

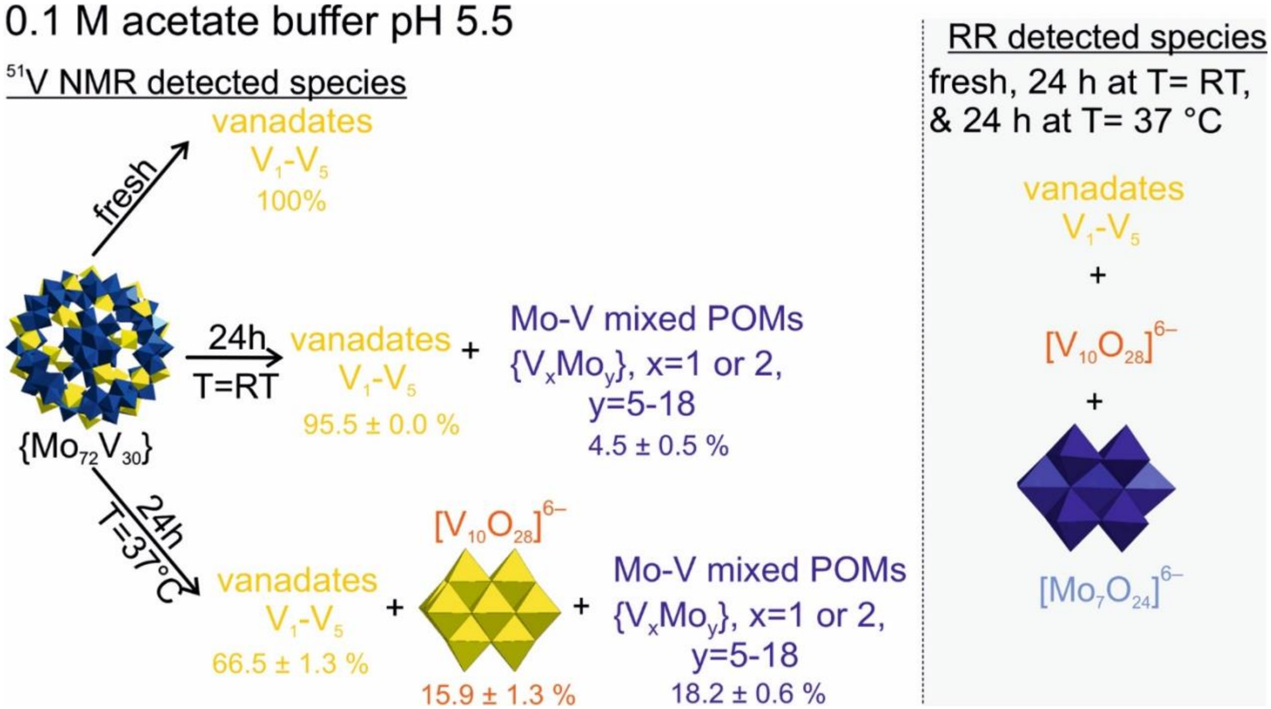

Fig. S34. Rearrangement scheme of {$Mo_{72}V_{30}$} in 0.1 M sodium acetate – acetic acid buffer (pH 5.5).

The scheme is based on the collected and analyzed ^{51}V NMR and resonance Raman data (gray field). The structures of all POMs are shown in **Figures S1** and **S2**. **Tables S8 – S10** give all ^{51}V NMR chemical shifts and percentages of species detectable by ^{51}V NMR (**Table S13**) formed and **Table S15** gives resonance Raman shifts, and their assignation based on literature data (**Tables S5 – S6**). Only species with a percentage > 10 % are shown separately; other species are separated by the type of addenda metals in the structure. Color code: {MoO_6}, blue; {VO_6}, yellow.

5.4.4. {$Mo_{72}V_{30}$} rearrangement schemes in 0.1 M Tris – HCl buffers

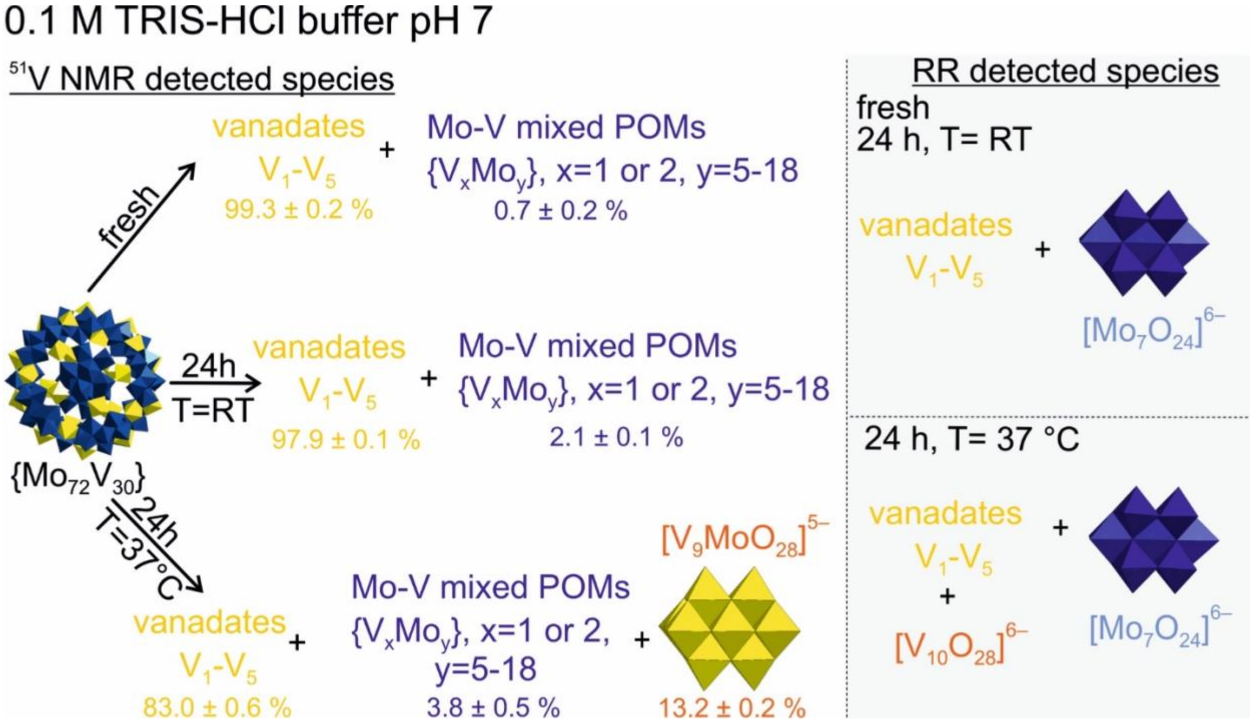

Fig. S35. Rearrangement scheme of {$Mo_{72}V_{30}$} in 0.1 M Tris–HCl buffer (pH 7).
The scheme is based on the collected and analyzed ^{51}V NMR and resonance Raman data (gray field). The structures of all POMs are shown in **Figures S1** and **S2**. **Tables S8 – S10** give all ^{51}V NMR chemical shifts and percentages of species detectable by ^{51}V NMR (**Table S 14**) formed and **Table S15** gives resonance Raman shifts, and their assignation based on literature data (**Tables S5 – S6**). Only species with a percentage > 10 % are shown separately; other species are separated by the type of addenda metals in the structure. Color code: {MoO_6}, blue; {VO_6}, yellow.

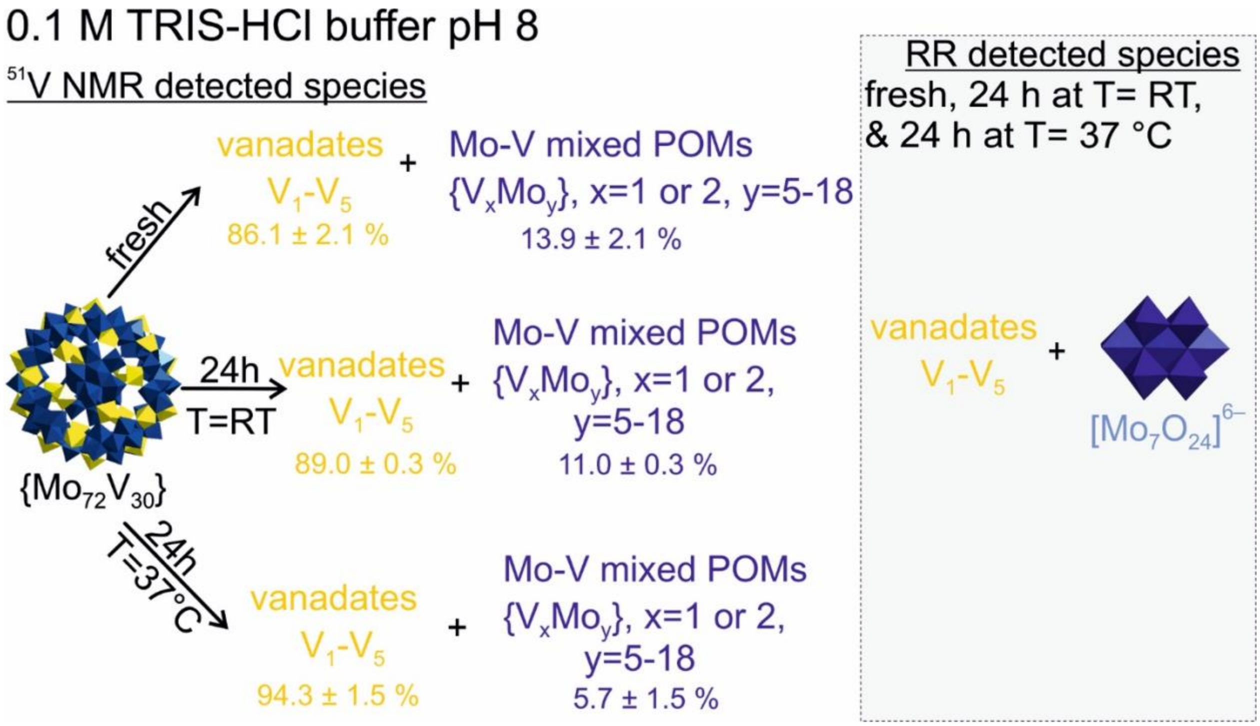

Fig. S36. Rearrangement scheme of $\{Mo_{72}V_{30}\}$ in 0.1 M Tris–HCl buffer (pH 8).

The scheme is based on the collected and analyzed ^{51}V NMR and resonance Raman data (gray field). The structures of all POMs are shown in **Figures S1** and **S2**. **Tables S8 – S10** give all ^{51}V NMR chemical shifts and percentages of species detectable by ^{51}V NMR (**Table S14**) formed and **Table S15** gives resonance Raman shifts, and their assignation based on literature data (**Tables S5 – S6**). Only species with a percentage > 10 % are shown separately; other species are separated by the type of addenda metals in the structure. Color code: $\{MoO_6\}$, blue; $\{VO_6\}$, yellow.

5.4.5. {$Mo_{72}V_{30}$} rearrangement schemes in 0.1 M HEPES buffers

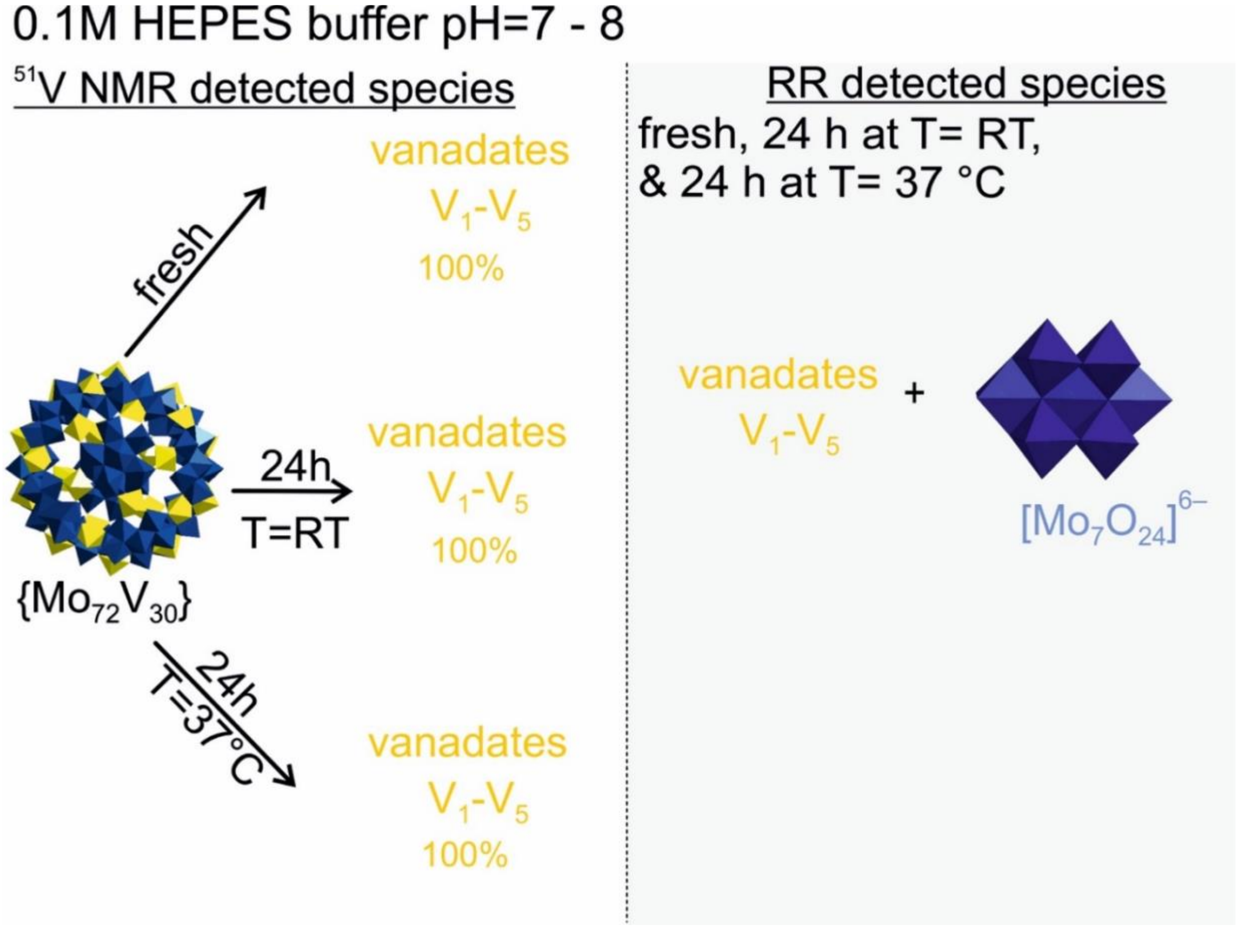

Fig. S37. Rearrangement scheme of {$Mo_{72}V_{30}$} in 0.1 M HEPES buffers (pH 7 and 8).
The scheme is based on the collected and analyzed ^{51}V- and resonance Raman data (gray field). The structures of all POMs are shown in **Figures S1** and **S2**. **Tables S8 – S10** give all ^{51}V NMR chemical shifts and percentages of species detectable by ^{51}V NMR (**Table S14**) formed and **Table S15** gives resonance Raman shifts, and their assignation based on literature data (**Tables S5 – S6**). Only species with a percentage > 10 % are shown separately; other species are separated by the type of addenda metals in the structure. Color code: {MoO_6}, blue; {VO_6}, yellow.

5.5. UV-vis spectroscopy of {$Mo_{72}V_{30}$} in aqueous solutions

UV-vis data was collected every 30 min for 24 h. The “fresh solutions” were measured after 3 min of stirring until the {$Mo_{72}V_{30}$} was dissolved entirely and 1 min of incubation in the UV-VIS spectrometer in total 4 min).

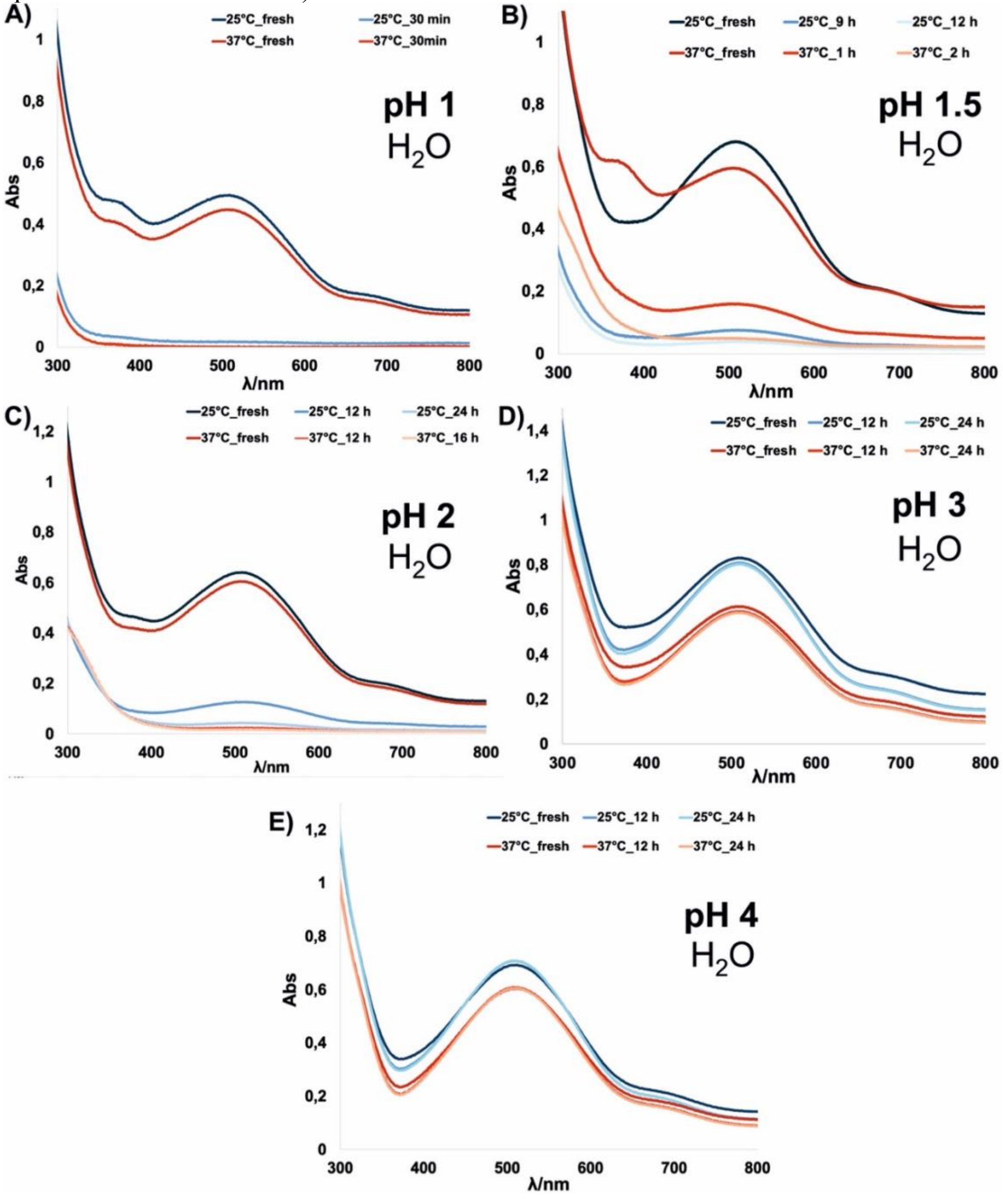

Fig. S38. UV-vis spectra of {$Mo_{72}V_{30}$} in H_2O.

UV-vis spectra of 10 µM {$Mo_{72}V_{30}$} in H_2O at T = 25 °C and T = 37 °C either until complete oxidation (loss of absorbance band) or 0 – 12 – 24 h period: A) H_2O pH 1, B) H_2O pH 1.5, C) H_2O pH 2 and D) H_2O pH 3, E) H_2O pH 4.

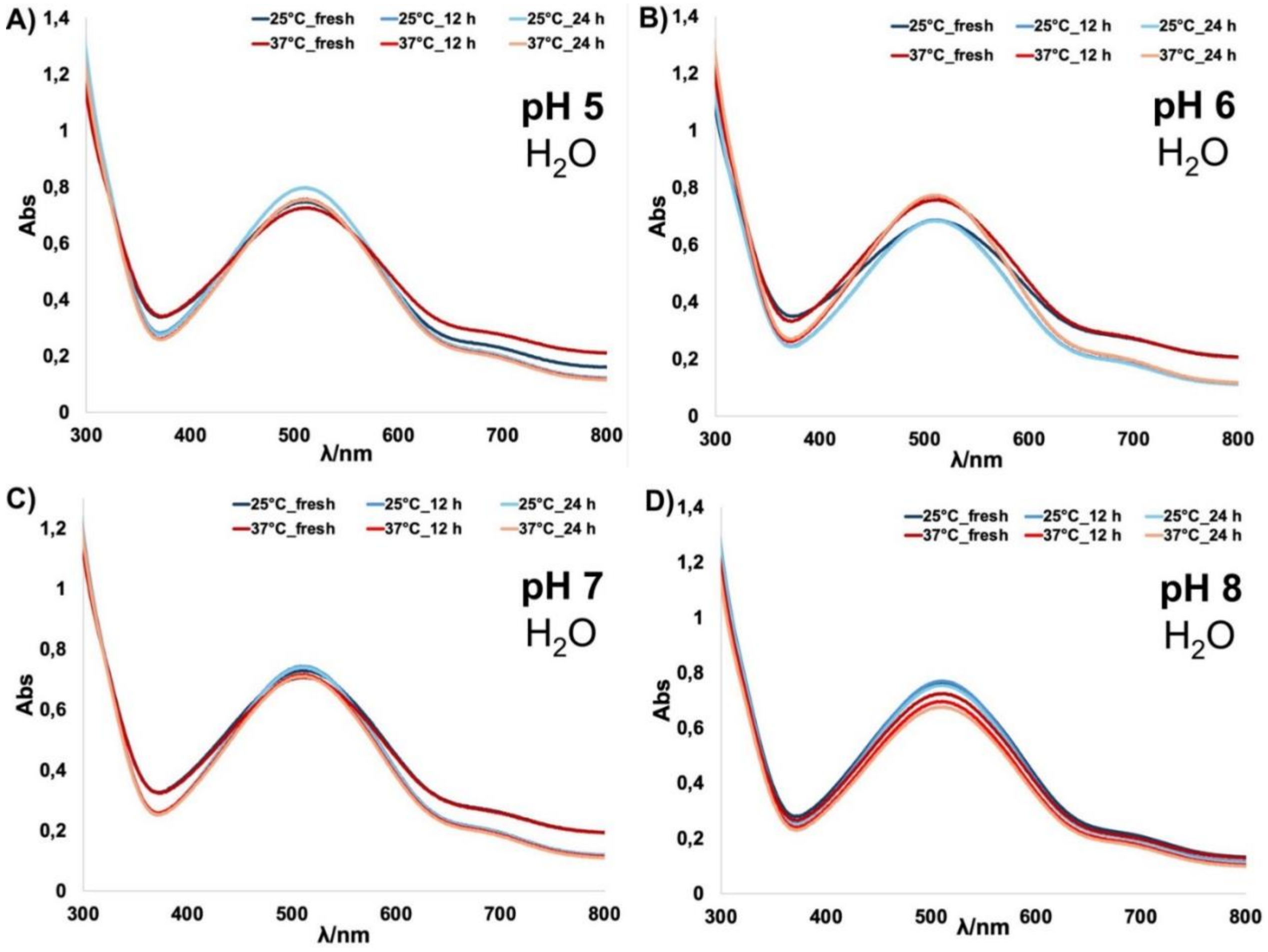

Fig. S39. UV-vis spectra of {$Mo_{72}V_{30}$} in H_2O.

UV-vis spectra of 10 μM {$Mo_{72}V_{30}$} in H_2O at T = 25 °C and T = 37 °C either until complete oxidation (loss of absorbance band) or 0 – 12 – 24 h period: A) H_2O pH 5, B) H_2O pH 6, C) H_2O pH 7, and D) H_2O pH 8.

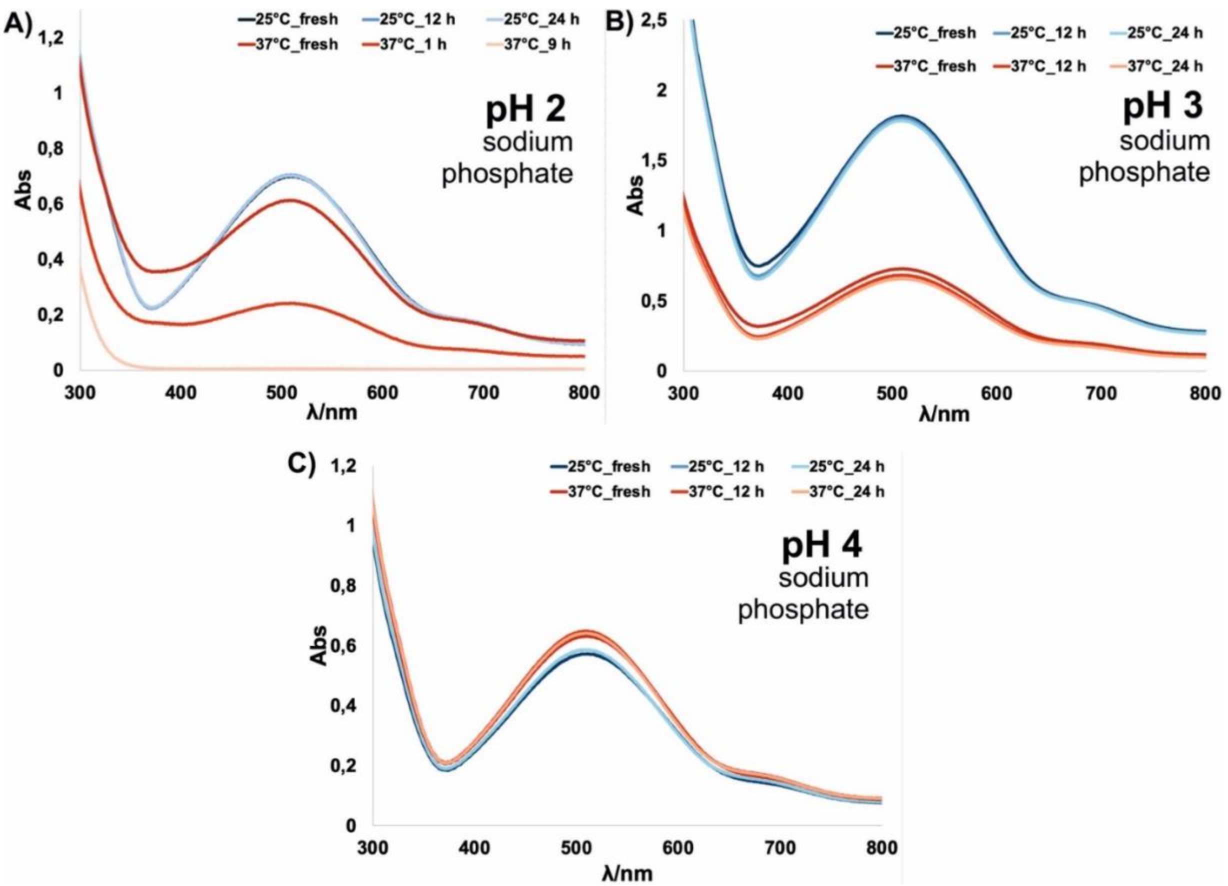

Fig. S40. UV-vis spectra of {$Mo_{72}V_{30}$} in sodium phosphate buffers.

UV-VIS spectra of 10 μM {$Mo_{72}V_{30}$} in 0.1 M sodium phosphate buffers at T = 25 °C and T = 37 °C either until complete oxidation (loss of absorbance band) or 0 – 12 – 24 h period: A) 0.1 M sodium phosphate buffer pH 2, C) 0.1 M sodium phosphate buffer pH 3, and C) 0.1 M sodium phosphate buffer pH 4.

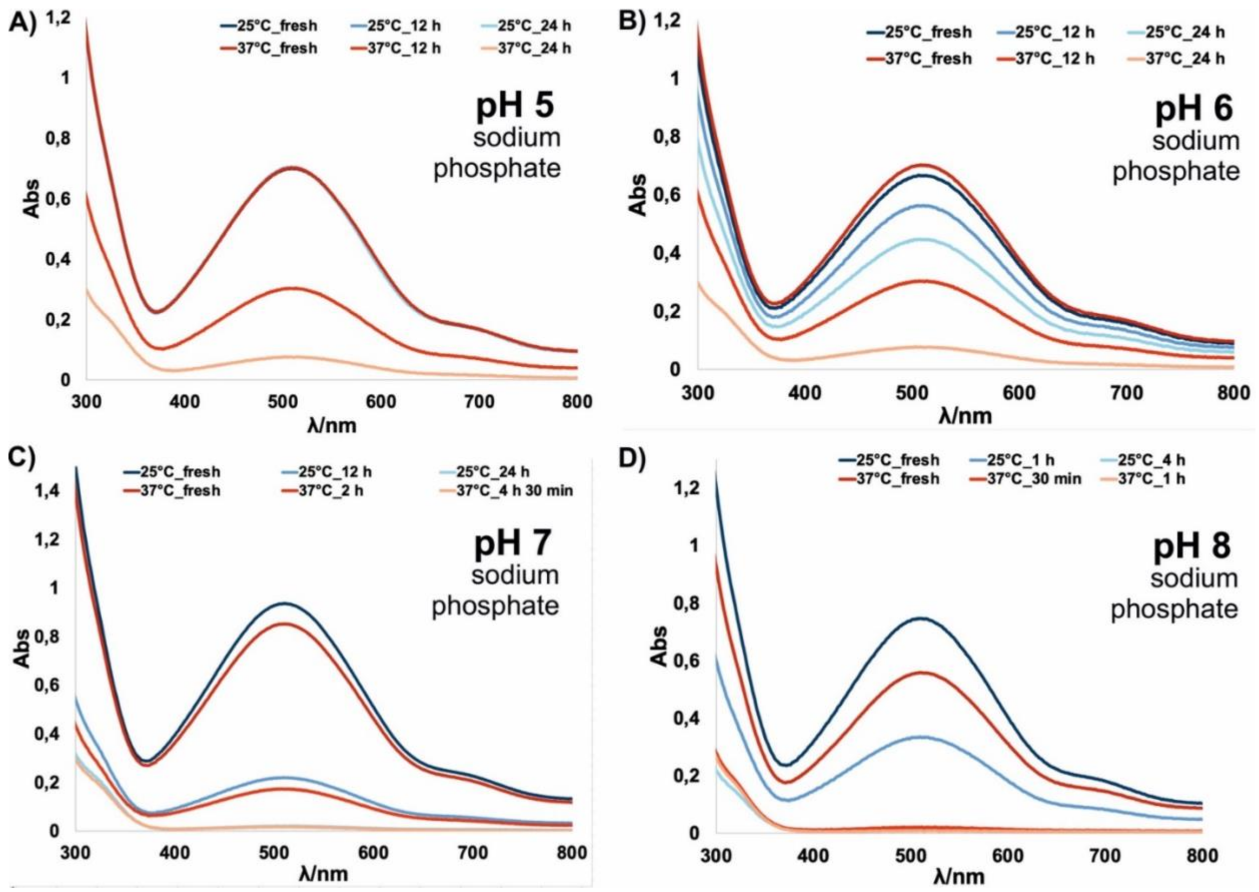

Fig. S41. UV-vis spectra of {$Mo_{72}V_{30}$} in sodium phosphate buffers.

UV-vis spectra of 10 μM {$Mo_{72}V_{30}$} in 0.1 M sodium phosphate buffers at T = 25 °C and T = 37 °C either until complete oxidation (loss of absorbance band) or 0 – 12 – 24 h period: A) 0.1 M sodium phosphate buffer pH 5, B) 0.1 M sodium phosphate buffer pH 6, C) 0.1 M sodium phosphate buffer pH 7 and D) 0.1 M sodium phosphate buffer pH 8.

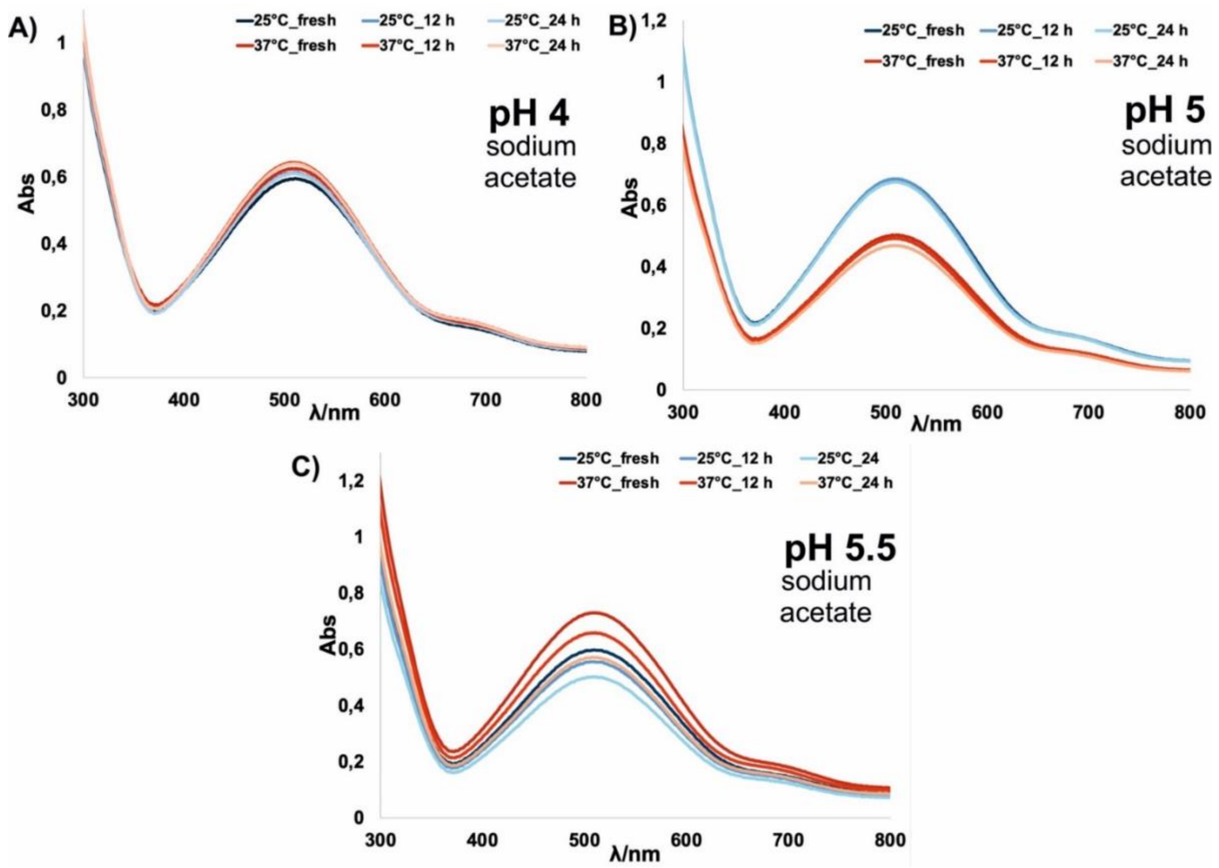

Fig. S42. UV-vis spectra of {$Mo_{72}V_{30}$} in acetic acid – sodium acetate buffers.

UV-Vis spectra of 10 μM {$Mo_{72}V_{30}$} in 0.1 M acetic acid – sodium acetate buffers at T = 25 °C and T = 37 °C either until complete oxidation (loss of absorbance band) or 0 – 12 – 24 h period: A) 0.1 M acetic acid – sodium acetate buffer pH 4, B) 0.1 M acetic acid – sodium acetate buffer pH 5, and C) 0.1 M acetic acid – sodium acetate buffer pH 5.5.

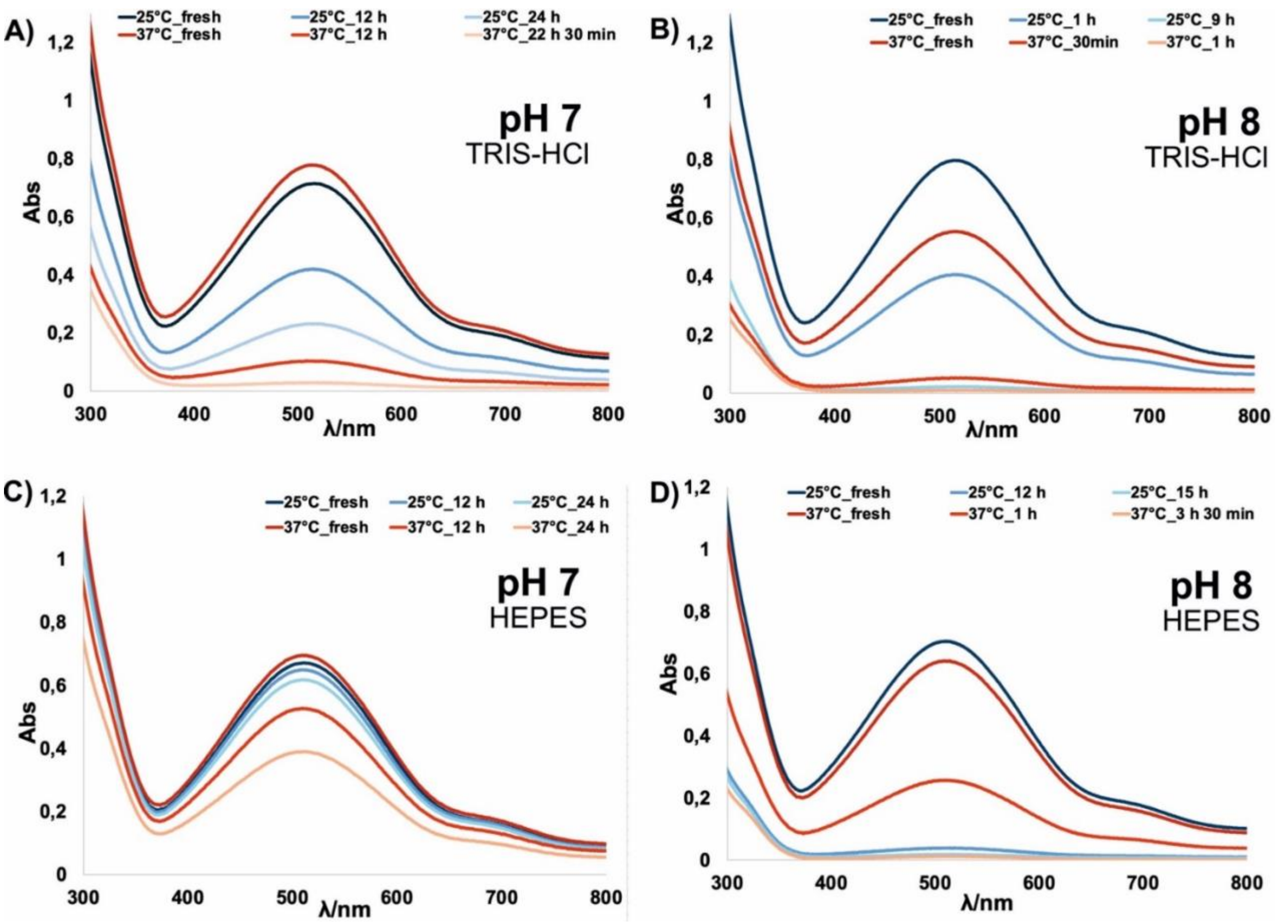

Fig. S43. UV-vis spectra of {$Mo_{72}V_{30}$} in Tris-HCl and HEPES buffers.

UV-vis spectra of 10 μM {$Mo_{72}V_{30}$} in 0.1 M Tris – HCl and 0.1 M HEPES buffers at T = 25 °C and T = 37 °C either until complete oxidation (loss of absorbance band) or 0 – 12 – 24 h period: A) 0.1 M Tris – HCl buffer pH 7, B) 0.1 M Tris – HCl buffer pH 8, C) 0.1 M HEPES buffer pH 7 and D) 0.1 M HEPES buffer pH 8.

5.6. Summary of ^{51}V-NMR, resonance Raman and UV-VIS spectroscopy experiments

Table S16. Summarized comparison of results of three analytical methods: ^{51}V-NMR, Resonance Raman, UV – nir – vis and ESI-MS (fresh {$Mo_{72}V_{30}$} non-buffered solutions with pH 1 – 8).

H_2O							
		NMR (0.15 mM {$Mo_{72}V_{30}$})		Resonance Raman (0.15 mM {$Mo_{72}V_{30}$})		UV – VIS (10 µM {$Mo_{72}V_{30}$})	ESI-MS (1 mg/mL {$Mo_{72}V_{30}$})
Sample condition		Intact {$Mo_{72}V_{30}$}	Post-decomposition POM species	Intact {$Mo_{72}V_{30}$}	Post-decomposition POM species	Reduced/ Not reduced	Detection of {$Mo_{72}V_{30}$} anion
pH 1	Fresh	*Yes	Yes	Yes	No	Yes	No
	24 h at RT	*Yes	Yes	No	Yes	No	-
	24 h at 37 °C	Yes	Yes	No	No	No	-
pH 1.5	Fresh	*Yes	Yes	Yes	No	Yes	No
	24 h at RT	*Yes	Yes	No	Yes	No	-
	24 h at 37 °C	No	Yes	No	Yes	No	-
pH 2	Fresh	*Yes	Yes	Yes	No	Yes	Yes
	24 h at RT	*Yes	Yes	Yes	No	Yes	-
	24 h at 37 °C	*Yes	Yes	Yes	No	No	-
pH 3	Fresh	*Yes	Yes	Yes	No	Yes	Yes
	24 h at RT	*Yes	Yes	Yes	No	Yes	-
	24 h at 37 °C	*Yes	Yes	Yes	No	Yes	-
pH 4	Fresh	No	Yes	Yes	Yes	Yes	Yes
	24 h at RT	No	Yes	Yes	No	Yes	-
	24 h at 37 °C	No	Yes	Yes	No	Yes	-
pH 5	Fresh	No	Yes	Yes	No	Yes	Yes
	24 h at RT	No	Yes	Yes	No	Yes	-
	24 h at 37 °C	No	Yes	Yes	No	Yes	-
pH 6	Fresh	No	Yes	Yes	No	Yes	Yes
	24 h at RT	No	Yes	Yes	Yes	Yes	-
	24 h at 37 °C	No	Yes	Yes	No	Yes	-
pH 7	Fresh	No	Yes	Yes	No	Yes	Yes
	24 h at RT	No	Yes	Yes	Yes	Yes	-
	24 h at 37 °C	No	Yes	No	Yes	Yes	-
	Fresh	No	Yes	Yes	Yes	Yes	Yes

pH 8	24 h at RT	No	Yes	Yes	Yes	Yes	-
	24 h at 37 °C	No	Yes	Yes	No	Yes	-

0.1 M sodium phosphate buffers

		NMR (0.15 mM $\{Mo_{72}V_{30}\}$)		Resonance Raman (0.15 mM $\{Mo_{72}V_{30}\}$)		UV – VIS (10 µM $\{Mo_{72}V_{30}\}$)	ESI-MS (1 mg/mL $\{Mo_{72}V_{30}\}$)
Sample condition		Intact $\{Mo_{72}V_{30}\}$	Post-decomposition POM species	Intact $\{Mo_{72}V_{30}\}$	Post-decomposition POM species	Reduced/ Not reduced	Detection of $\{Mo_{72}V_{30}\}$ anion
pH 2	Fresh	*Yes	Yes	No	Yes	Yes	-
	24 h at RT	*Yes	Yes	No	Yes	Yes	-
	24 h at 37 °C	*Yes	Yes	Yes	No	No	-
pH 3	Fresh	*Yes	Yes	Yes	No	Yes	-
	24 h at RT	*Yes	Yes	Yes	No	Yes	-
	24 h at 37 °C	*Yes	Yes	No	Yes	Yes	-
pH 4	Fresh	No	Yes	Yes	Yes	Yes	-
	24 h at RT	No	Yes	Yes	No	Yes	-
	24 h at 37 °C	No	Yes	Yes	No	Yes	-
pH 5	Fresh	No	Yes	Yes	No	Yes	-
	24 h at RT	No	Yes	Yes	No	Yes	-
	24 h at 37 °C	No	Yes	No	Yes	Yes	-
pH 6	Fresh	No	Yes	No	Yes	Yes	-
	24 h at RT	No	Yes	No	Yes	Yes	-
	24 h at 37 °C	No	Yes	No	Yes	Yes	-
pH 7	Fresh	No	Yes	No	Yes	Yes	-
	24 h at RT	No	Yes	No	Yes	No	-
	24 h at 37 °C	No	Yes	No	Yes	No	-
pH 8	Fresh	No	Yes	No	Yes	Yes	-
	24 h at RT	No	Yes	No	Yes	No	-
	24 h at 37 °C	No	Yes	No	Yes	No	-

0.1 M acetic acid – sodium acetate buffers

		NMR (0.20 mM $\{Mo_{72}V_{30}\}$)		Resonance Raman (0.20 mM $\{Mo_{72}V_{30}\}$)		UV – VIS (10 µM $\{Mo_{72}V_{30}\}$)	ESI-MS (1 mg/mL $\{Mo_{72}V_{30}\}$)
Sample condition		Intact $\{Mo_{72}V_{30}\}$	Post-decomposition POM species	Intact $\{Mo_{72}V_{30}\}$	Post-decomposition POM species	Reduced/ Not reduced	Detection of $\{Mo_{72}V_{30}\}$ anion
	Fresh	*Yes	Yes	No	Yes	Yes	-

pH 4	24 h at RT	*Yes	Yes	Yes	Yes	Yes	-
	24 h at 37 °C	*Yes	Yes	No	Yes	Yes	-
pH 5	Fresh	No	Yes	No	Yes	Yes	-
	24 h at RT	No	Yes	Yes	No	Yes	-
	24 h at 37 °C	No	Yes	No	Yes	Yes	-
pH 5.5	Fresh	No	Yes	No	Yes	Yes	-
	24 h at RT	No	Yes	No	Yes	Yes	-
	24 h at 37 °C	No	Yes	No	Yes	Yes	-
**0.1 M Tris – HCl buffers**							
		NMR (0.15 mM $\{Mo_{72}V_{30}\}$)		Resonance Raman (0.15 mM $\{Mo_{72}V_{30}\}$)		UV – VIS (10 µM $\{Mo_{72}V_{30}\}$)	ESI-MS (1 mg/mL $\{Mo_{72}V_{30}\}$)
Sample condition		Intact $\{Mo_{72}V_{30}\}$	Post-decomposition POM species	Intact $\{Mo_{72}V_{30}\}$	Post-decomposition POM species	Reduced/ Not reduced	Detection of $\{Mo_{72}V_{30}\}$ anion
pH 7	Fresh	No	Yes	No	Yes	Yes	-
	24 h at RT	No	Yes	No	Yes	Yes	-
	24 h at 37 °C	No	Yes	No	Yes	No	-
pH 8	Fresh	No	Yes	No	Yes	Yes	-
	24 h at RT	No	Yes	No	Yes	No	-
	24 h at 37 °C	No	Yes	No	Yes	No	-
**0.1 M HEPES buffers**							
		NMR (0.15 mM $\{Mo_{72}V_{30}\}$)		Resonance Raman (0.15 mM $\{Mo_{72}V_{30}\}$)		UV – VIS (10 µM $\{Mo_{72}V_{30}\}$)	ESI-MS (1 mg/mL $\{Mo_{72}V_{30}\}$)
Sample condition		Intact $\{Mo_{72}V_{30}\}$	Post-decomposition POM species	Intact $\{Mo_{72}V_{30}\}$	Post-decomposition POM species	Reduced/ Not reduced	Detection of $\{Mo_{72}V_{30}\}$ anion
pH 7	Fresh	No	Yes	No	Yes	Yes	-
	24 h at RT	No	Yes	No	Yes	Yes	-
	24 h at 37 °C	No	Yes	No	Yes	Yes	-
pH 8	Fresh	No	Yes	No	Yes	Yes	-
	24 h at RT	No	Yes	No	Yes	No	-
	24 h at 37 °C	No	Yes	No	Yes	No	-

*presence of Keplerate anion due to either shape of ^{51}V NMR spectra or no ^{51}V-NMR signal in 0.15 mM aqueous solutions because of paramagnetic V(IV) in solution

5.7. Electrospray ionization-mass spectrometry experiments

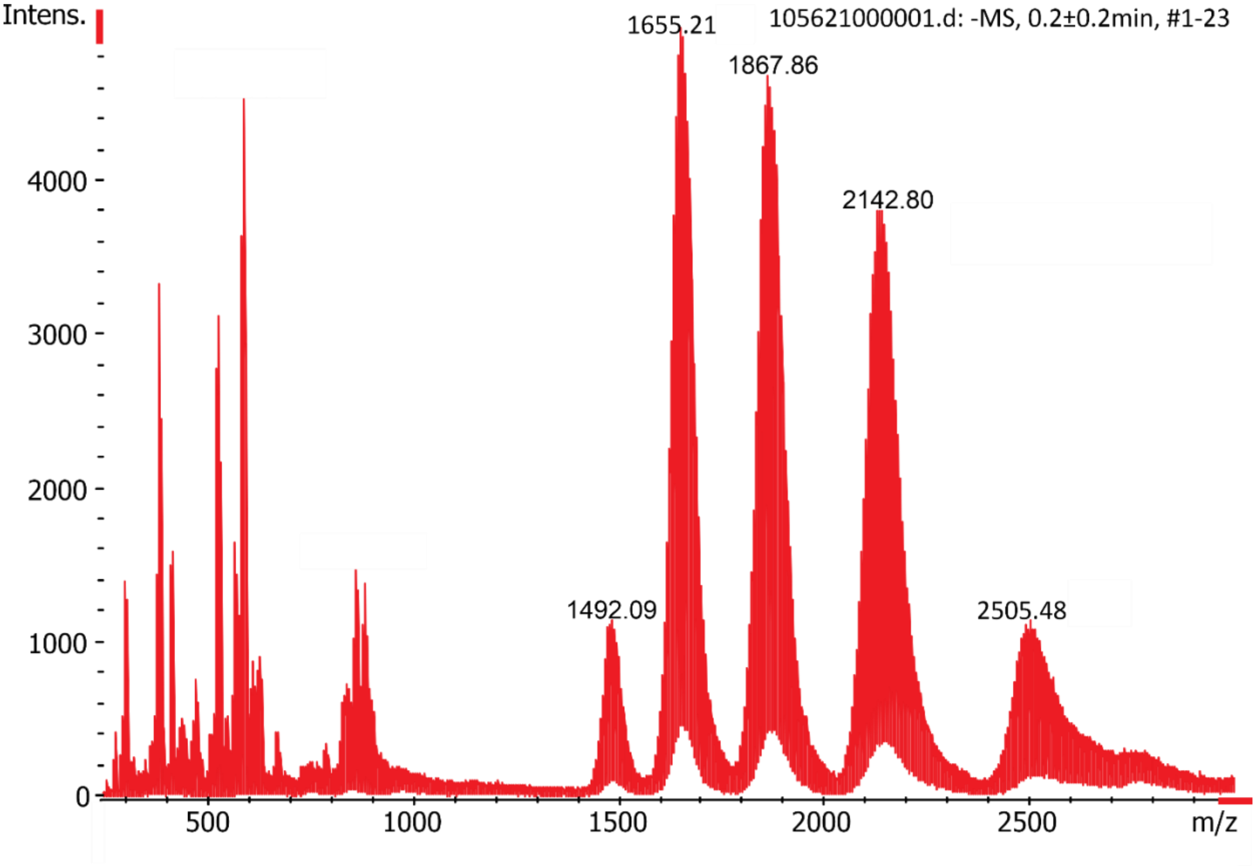

Fig. S44. ESI-MS spectra of 1 mg/mL {$Mo_{72}V_{30}$} fresh solution in H_2O at pH= 3.

The m/z envelopes that come from the large {$Mo_{72}V_{30}$} anions have also visible average m/z(experimental) of differently charge large anions listed in **Tables S17** and **S18**. The formula of Keplerate anions detected by ESI-MS are listed in **Table S17** together with their m/z values.

Table S17. ESI-MS data of 1 mg/ mL {$Mo_{72}V_{30}$} fresh solution in H_2O with pH 3

ESI-MS data of {$Mo_{72}V_{30}$} in H_2O with pH 3. The pH of solutions was adjusted using acid (HCl for pH ≤ 2 and formic acid for pH 3 – 6) or NH_3(aq) (pH > 7). The spectra were measured ~30 min after sample preparation. The MS spectra of {$Mo_{72}V_{30}$} solutions at pH ≤ 2 showed no detectable Keplerate anions, possibly due to the high concentration of Cl^- ions interfering with the detection of large anions (high m/z) by the mass spectrometer.

**pH 3**				
**Anion charge**	**POM anion formula**	**m/z (experimental)**	**m/z (calculated)**	**m/z (average)**
**-10**	**{$Na_xK_yH_{102-x-y}Mo_{72}V_{30}S_{10}O_{362}$}$^{10-}$, x=0 – 8, y=4 – 8**	1484.3 – 1513.3	1484.3 – 1513.3	**1492.1**
	**{$Na_xK_yH_{98-x-y}Mo_{72}V_{30}S_{10}O_{360}$}$^{10-}$, x=0 – 8, y=0 – 3**	1461.7 – 1486.9	1461.7 – 1486.9	

	**$\{Na_xK_yH_{92-x-y}Mo_{72}V_{30}S_{11}O_{360}\}^{10-}$, x=0 – 8, y=0 – 1**	1464.3 – 1481.8	1464.3 – 1481.8	
	**$\{Na_xH_{90-x}Mo_{72}V_{30}S_{12}O_{362}\}^{10-}$, x= 0 – 8**	1470.5 – 1488.0	1470.5 – 1488.0	
	**$\{Na_xK_yH_{90-x-y}Mo_{72}V_{30}S_{12}O_{362}\}^{10-}$, x= 0 – 4, y=1**	1474.3 – 1483.1	1474.3 – 1483.1	
	**$\{Na_xK_yH_{88-x-y}Mo_{72}V_{30}S_{11}O_{358}\}^{10-}$, x=4 or 6, y=0**	1473.8 – 1469.4	1473.8 – 1469.4	
**-9**	**$\{Na_xK_yH_{103-x-y}Mo_{72}V_{30}S_{10}O_{362}\}^{9-}$, x=0 – 8, y=1 – 9**	1657.8 – 1681.6	1657.8 – 1681.6	**1655.2**
	**$\{Na_xK_yH_{99-x-y}Mo_{72}V_{30}S_{10}O_{360}\}^{9-}$, x=0 – 8, y=0 – 4**	1624.2 – 1656.4	1624.2 – 1656.4	
	**$\{Na_xK_yH_{97-x-y}Mo_{72}V_{30}S_{11}O_{362}\}^{9-}$, x=0 – 8, y=0 – 4**	1631.1 – 1663.3	1631.1 – 1663.3	
	**$\{Na_xK_yH_{91-x-y}Mo_{72}V_{30}S_{12}O_{362}\}^{9-}$, x=0 – 7, y=0**	1634.0 – 1651.1	1634.0 – 1651.1	
	**$\{Na_xK_yH_{89-x-y}Mo_{72}V_{30}S_{11}O_{358}\}^{9-}$, x=0 – 7, y=0**	1638.1 – 1640.2	1638.1 – 1640.2	
**-8**	**$\{Na_xK_yH_{104-x-y}Mo_{72}V_{30}S_{10}O_{362}\}^{8-}$, x=0 – 8, y=1 – 9**	1860.4 – 1887.1	1860.4 – 1887.1	**1867.9**
	**$\{Na_xK_yH_{100-x-y}Mo_{72}V_{30}S_{10}O_{360}\}^{8-}$, x=0 – 8, y=0 – 4**	1827.3 – 1860.8	1827.3 – 1860.8	
	**$\{Na_xK_yH_{98-x-y}Mo_{72}V_{30}S_{11}O_{362}\}^{8-}$, x=0 – 8, y=0 – 3**	1839.1 – 1866.6	1839.1 – 1866.6	
	**$\{Na_xK_yH_{92-x-y}Mo_{72}V_{30}S_{12}O_{362}\}^{8-}$, x=0 – 5, y=0**	1838.3 – 1852.1	1838.3 – 1852.1	
**-7**	**$\{Na_xK_yH_{105-x-y}Mo_{72}V_{30}S_{10}O_{362}\}^{7-}$, x=0 – 8, y=4 – 9**	2115.4 – 2153.7	2115.4 – 2153.7	**2142.8**
	**$\{Na_xK_yH_{101-x-y}Mo_{72}V_{30}S_{10}O_{360}\}^{7-}$, x=0 – 8, y=2 – 5**	2099.4 – 2135.4	2099.4 – 2135.4	
	**$\{Na_xK_yH_{99-x-y}Mo_{72}V_{30}S_{11}O_{362}\}^{7-}$, x=0 – 8, y=0 – 5**	2109.1 – 2144.3	2109.1 – 2144.3	
	**$\{Na_xK_yH_{93-x-y}Mo_{72}V_{30}S_{12}O_{362}\}^{7-}$, x=0 – 8, y=0 – 2**	2101.1 – 2131.7	2101.1 – 2131.7	

	**$\{Na_xK_yH_{91-x-y}Mo_{72}V_{30}S_{11}O_{358}\}^{7-}$, x=0 – 1, y=0**	2087.1 – 2090.2	2087.1 – 2090.2	
**-6**	**$\{Na_xK_yH_{106-x-y}Mo_{72}V_{30}S_{10}O_{362}\}^{6-}$, x=1 – 8, y=1 – 6**	2478.2 – 2497.5	2478.2 – 2497.5	**2505.5**
	**$\{Na_xK_yH_{102-x-y}Mo_{72}V_{30}S_{10}O_{360}\}^{6-}$, x=1 – 8, y=1 – 4**	2456.8 – 2481.5	2456.8 – 2481.5	
	**$\{Na_xK_yH_{100-x-y}Mo_{72}V_{30}S_{11}O_{362}\}^{6-}$, x=0 – 8, y=0 – 2**	2447.1 – 2479.1	2447.1 – 2479.1	
	**$\{Na_xK_yH_{94-x-y}Mo_{72}V_{30}S_{12}O_{362}\}^{6-}$, x=0 – 8, y=0 – 1**	2451.4 – 2480.8	2451.4 – 2480.8	

Table S18. ESI-MS data overview of 1 mg/ mL $\{Mo_{72}V_{30}\}$ fresh solution in H_2O with pH 1 – 8. Overview of all Keplerate anions detected by ESI-MS in fresh solutions of 1 mg/ mL $\{Mo_{72}V_{30}\}$ in H_2O with pH 1 – 8. The m/z of all POM anions formula (for pH 3) are listed in **Table S17**. Legend: – anion not detected; ✓ anion detected.

Anion charge	POM anion formula	pH 1	pH 1.5	pH 2	pH 3	pH 4	pH 5	pH 6	pH 7	pH 8
**-10**	**$\{Na_xK_yH_{102-x-y}Mo_{72}V_{30}S_{10}O_{362}\}^{10-}$, x=0 – 8, y=4 – 9**	–	–	–	✓	✓	✓	✓	✓	✓
	**$\{Na_xK_yH_{98-x-y}Mo_{72}V_{30}S_{10}O_{360}\}^{10-}$, x=0 – 8, y=0 – 7**	–	–	–	✓	✓	✓	✓	✓	✓
	**$\{Na_xK_yH_{92-x-y}Mo_{72}V_{30}S_{11}O_{360}\}^{10-}$, x=0 – 8, y=0 – 1**	–	–	–	✓	✓	✓	✓	✓	✓
	**$\{Na_xH_{90-x}Mo_{72}V_{30}S_{12}O_{362}\}^{10-}$, x= 0 – 8**	–	–	–	✓	✓	✓	✓	✓	✓
	**$\{Na_xK_yH_{90-x-y}Mo_{72}V_{30}S_{12}O_{362}\}^{10-}$, x= 0 – 7, y= 1 – 3**	–	–	–	✓	✓	✓	✓	✓	✓
	**$\{Na_xK_yH_{88-x-y}Mo_{72}V_{30}S_{11}O_{358}\}^{10-}$, x=0 – 8, y=0 – 2**	–	–	–	✓	✓	✓	✓	✓	✓
**-9**	**$\{Na_xK_yH_{103-x-y}Mo_{72}V_{30}S_{10}O_{362}\}^{9-}$, x=0 – 8, y=1 – 9**	–	–	–	✓	✓	✓	✓	✓	✓
	**$\{Na_xK_yH_{99-x-y}Mo_{72}V_{30}S_{10}O_{360}\}^{9-}$, x=0 – 8, y=0 – 4**	–	–	–	✓	✓	✓	✓	✓	✓
	**$\{Na_xK_yH_{97-x-y}Mo_{72}V_{30}S_{11}O_{362}\}^{9-}$, x=0 – 8, y=0 – 4**	–	–	–	✓	✓	✓	✓	✓	✓
	**$\{Na_xK_yH_{91-x-y}Mo_{72}V_{30}S_{12}O_{362}\}^{9-}$, x=0 – 7, y=0**	–	–	–	✓	✓	✓	✓	✓	✓
	**$\{Na_xK_yH_{89-x-y}Mo_{72}V_{30}S_{11}O_{358}\}^{9-}$, x=0 – 7, y=0**	–	–	–	✓	✓	✓	✓	✓	✓
**-8**	**$\{Na_xK_yH_{104-x-y}Mo_{72}V_{30}S_{10}O_{362}\}^{8-}$, x=0-8, y=1-9**	–	–	–	✓	✓	✓	✓	✓	✓
	**$\{Na_xK_yH_{100-x-y}Mo_{72}V_{30}S_{10}O_{360}\}^{8-}$, x=0 – 8, y=0 – 6**	–	–	–	✓	✓	✓	✓	✓	✓
	**$\{Na_xK_yH_{98-x-y}Mo_{72}V_{30}S_{11}O_{362}\}^{8-}$, x=0 – 8, y= 0 – 6**	–	–	–	✓	✓	✓	✓	✓	✓
	**$\{Na_xK_yH_{92-x}Mo_{72}V_{30}S_{12}O_{362}\}^{8-}$, x=0 – 8, y=0 – 2**	–	–	–	✓	✓	✓	✓	✓	✓

	**$\{Na_xK_yH_{90-x-y}Mo_{72}V_{30}S_{11}O_{358}\}^{8-}$, x=0 − 8, y=0**	–	–	–	–	✓	✓	✓	✓	✓
**-7**	**$\{Na_xK_yH_{105-x-y}Mo_{72}V_{30}S_{10}O_{362}\}^{7-}$, x=0 − 8, y=4 − 9**	–	–	✓	✓	–	–	–	–	–
	**$\{Na_xK_yH_{101-x-y}Mo_{72}V_{30}S_{10}O_{360}\}^{7-}$, x=0 − 8, y=2 − 5**	–	–	✓	✓	–	–	–	–	–
	**$\{Na_xK_yH_{99-x-y}Mo_{72}V_{30}S_{11}O_{362}\}^{7-}$, x=0 − 8, y= 0 − 5**	–	–	✓	✓	–	–	–	–	–
	**$\{Na_xK_yH_{93-x-y}Mo_{72}V_{30}S_{12}O_{362}\}^{7-}$, x=0−8, y= 0−2**	–	–	✓	✓	–	–	–	–	–
	**$\{Na_xK_yH_{91-x-y}Mo_{72}V_{30}S_{11}O_{358}\}^{7-}$, x=0 − 1, y=0**	–	–	✓	✓	–	–	–	–	–
**-6**	**$\{Na_xK_yH_{106-x-y}Mo_{72}V_{30}S_{10}O_{362}\}^{6-}$, x=1 − 8, y=1 − 6**	–	–	✓	✓	–	–	–	–	–
	**$\{Na_xK_yH_{102-x-y}Mo_{72}V_{30}S_{10}O_{360}\}^{6-}$, x=1 − 8, y=1 − 4**	–	–	✓	✓	–	–	–	–	–
	**$\{H_{100-x-y}Na_xK_yMo_{72}V_{30}S_{11}O_{362}\}^{6-}$, x=0 − 8, y= 0 − 2**	–	–	✓	✓	–	–	–	–	–
	**$\{Na_xK_yH_{94-x-y}Mo_{72}V_{30}S_{12}O_{362}\}^{6-}$, x=0 − 8, y= 0 − 1**	–	–	✓	✓	–	–	–	–	–

6. Spectroscopic study of $\{W_{72}V_{30}\}$ Keplerate-type polyoxotungstate

6.1. pH of $\{W_{72}V_{30}\}$ solutions

Table S19. pH in $\{W_{72}V_{30}\}$ solutions.

pH measured in triplicate of 0.15 mM **$\{W_{72}V_{30}\}$** dissolved in 10% D_2O/H_2O (pH 1 – 8) and 0.1 M buffers (acetic acid – sodium acetate pH 4 – 5.5; sodium phosphate pH 2 – 8 (while phosphate does not buffer at pH range from 3.5 – 5.5, experiments were conducted at this pH to provide comparisons to previously published studies (*239*)); Tris-HCl pH 7 – 8; HEPES pH 7 – 8). The initial pH just in distilled water is ~3.6 for **$\{W_{72}V_{30}\}$**, with no measurable change over 72 h without external pH adjustment. The initial pH just in distilled water is ~3.6 for **$\{W_{72}V_{30}\}$**, with no measurable change over 72 h without external pH adjustment.

**pH**	**$\{W_{72}V_{30}\}$ (0.15 mM) in Solvent / 0.1 M Buffer**	**pH after dissolving $\{W_{72}V_{30}\}$**				**pH after 24 h aging of $\{W_{72}V_{30}\}$ at RT[b]**				**pH after 24 h incubation of $\{W_{72}V_{30}\}$ at 37 °C**			
		**Sample**			**Mean of 1 to 3 ± SD[a]**	**Sample**			**Mean of 1 to 3 ± SD[a]**	**Sample**			**Mean of 1 to 3 ± SD[a]**
		#1	#2	#3		#1	#2	#3		#1	#2	#3	
Strongly acidic environment 1 ≤ pH ≤ 4													
1	10% D_2O/H_2O pH 1	1.08	1.01	1.10	1.06 ± 0.05	1.15	0.99	1.07	1.07 ± 0.08	1.04	0.99	1.03	1.02 ± 0.03
1.5	10% D_2O/H_2O pH 1.5	1.54	1.52	1.48	1.51 ± 0.03	1.55	1.55	1.51	1.54 ± 0.02	1.48	1.40	1.37	1.42 ± 0.06
2	10% D_2O/H_2O pH 2	2.06	2.02	2.02	2.02 ± 0.02	2.06	2.00	2.05	2.04 ± 0.03	2.10	2.12	2.07	2.10 ± 0.03
	sodium phosphate ($H_2PO_4^-/H_3PO_4$) pH 2	2.00	2.00	2.01	2.00 ± 0.01	2.01	2.01	2.01	2.01 ± 0.00	1.99	2.01	2.01	2.00 ± 0.01
3	10% D_2O/H_2O pH 3	3.12	3.08	3.10	3.10 ± 0.02	3.07	3.03	3.04	3.05 ± 0.02	3.03	2.97	3.00	3.00 ± 0.03
	sodium phosphate pH 3	3.01	3.17	3.03	3.07 ± 0.09	3.07	3.05	3.05	3.06 ± 0.01	3.07	3.05	3.07	3.06 ± 0.01
4	10% D_2O/H_2O pH 4	4.07	3.86	3.93	3.95 ± 0.11	4.01	3.90	3.87	3.93 ± 0.07	3.88	3.73	3.76	3.79 ± 0.08
	sodium phosphate ($H_2PO_4^-/H_3PO_4$) pH 4	4.04	4.01	3.96	4.00 ± 0.04	3.99	3.99	3.95	3.98 ± 0.02	3.87	3.91	3.80	3.86 ± 0.06
	acetic acid – sodium acetate ($OAc^-/HOAc$) pH 4	4.06	4.06	4.07	4.06 ± 0.01	4.02	4.00	4.01	4.01 ± 0.01	3.95	3.96	3.97	3.96 ± 0.01
Moderately acidic environment 5 ≤ pH ≤ 6													
5	10% D_2O/H_2O pH 5	5.11	5.04	4.94	5.03 ± 0.09	4.13	4.17	4.10	4.13 ± 0.04	4.11	3.99	3.83	3.98 ± 0.14
	sodium phosphate ($H_2PO_4^-/H_3PO_4$) pH 5	5.01	5.00	5.00	5.00 ± 0.01	4.87	4.90	4.91	4.89 ± 0.02	4.80	4.82	4.80	4.81 ± 0.01
	acetic acid – sodium acetate ($OAc^-/HOAc$) pH 5	5.00	5.00	5.01	5.00 ± 0.01	4.96	4.98	4.98	4.97 ± 0.01	4.92	4.90	4.90	4.91 ± 0.01

5.5	acetic acid – sodium acetate ($OAc^-/HOAc$) pH 5.5	5.50	5.50	5.49	5.50 ± 0.01	5.46	5.50	5.49	5.48 ± 0.02	5.42	5.43	5.43	5.43 ± 0.01
6	10% D_2O/H_2O pH 6	6.07	5.94	6.02	6.01 ± 0.07	5.17	4.99	5.15	5.10 ± 0.10	4.96	4.90	4.99	4.95 ± 0.05
	sodium phosphate ($H_2PO_4^-/H_3PO_4$) pH 6	6.03	6.02	6.02	6.02 ± 0.01	6.05	6.05	6.04	6.05 ± 0.01	5.97	6.00	5.99	5.99 ± 0.02
Neutral to moderately alkaline environment 7 ≤ pH ≤ 8													
7	10% D_2O/H_2O pH 7	7.00	6.98	6.80	6.93 ± 0.11	6.62	6.55	6.40	6.52 ± 0.11	6.44	6.40	6.33	6.39 ± 0.06
	sodium phosphate ($H_2PO_4^-/H_3PO_4$) pH 7	6.93	6.99	6.99	6.97 ± 0.03	6.93	6.95	6.99	6.96 ± 0.03	6.90	6.90	6.89	6.90 ± 0.01
	HEPES[c] pH 7	6.99	6.99	6.98	6.99 ± 0.01	6.97	6.99	6.97	6.98 ± 0.01	6.93	6.93	6.94	6.93 ± 0.01
	Tris[d]-HCl pH 7	6.79	6.87	6.87	6.84 ± 0.05	6.64	6.71	6.71	6.69 ± 0.04	6.60	6.60	6.62	6.61 ± 0.01
8	10% D_2O/H_2O pH 8	8.08	8.13	7.94	8.05 ± 0.10	7.69	7.48	7.33	7.50 ± 0.18	7.42	7.11	7.01	7.18 ± 0.21
	sodium phosphate ($H_2PO_4^-/H_3PO_4$) pH 8	8.06	8.02	8.02	8.03 ± 0.02	7.96	7.99	7.99	7.98 ± 0.02	7.80	7.81	7.83	7.81 ± 0.02
	HEPES[c] pH 8	7.96	7.97	7.97	7.97 ± 0.01	7.92	7.95	7.95	7.94 ± 0.02	7.87	7.85	7.85	7.86 ± 0.01
	Tris[d]-HCl pH 8	7.96	7.96	7.99	7.97 ± 0.02	7.76	7.77	7.76	7.76 ± 0.01	7.62	7.60	7.60	7.61 ± 0.01

[a]SD – standard deviation; [b]RT- room temperature; [c]HEPES – 4-(2-hydroxyethyl)-1-piperazineethanesulfonic acid, $C_8H_{18}N_2O_4S$; [d]tris – tris(hydroxymethyl)aminomethane, $C_4H_{11}NO_3$.

6.2. ^{51}V spectroscopic studies of {$W_{72}V_{30}$} solutions

All ^{51}V peaks were assigned based on the literature data from **Table S5**. In some ^{51}V spectra, several chemical shifts have not yet been described in the literature and, therefore, cannot be assigned in this work.

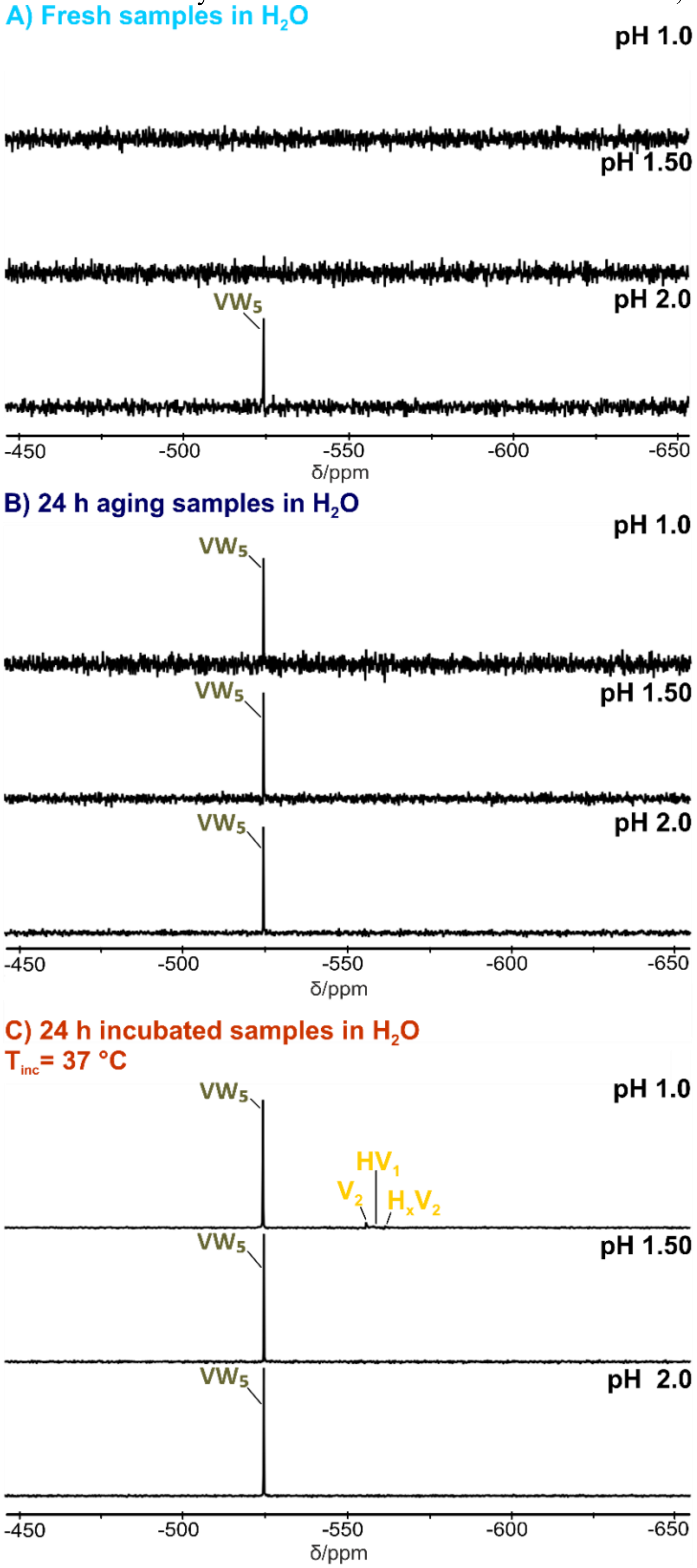

Fig. S45. ^{51}V NMR spectra of {$W_{72}V_{30}$} in H_2O (pH 1.0, pH 1.50, and pH 2.0): A) fresh solutions; B) 24 h aging at room temperature, and C) 24 h incubation at 37 °C.

^{51}V NMR spectra for 0.15 mM fresh solutions (**A**) of **{$W_{72}V_{30}$}** in H_2O (pH 1.0 – 2.0) that were recorded approximately one hour after preparation. Aliquots of fresh solutions were taken for 24 h aging experiments at room temperature (**B**) and 24 h incubation at 37 °C (**C**) and were then recorded approximately 1 h after the end of 24 h experiments. The structures of all POMs forming during the experiments are shown in **Figures S2 – S3**. The chemical shifts and percentages of parent (if still present) and formed POM species are given in **Tables S20 – S22**.

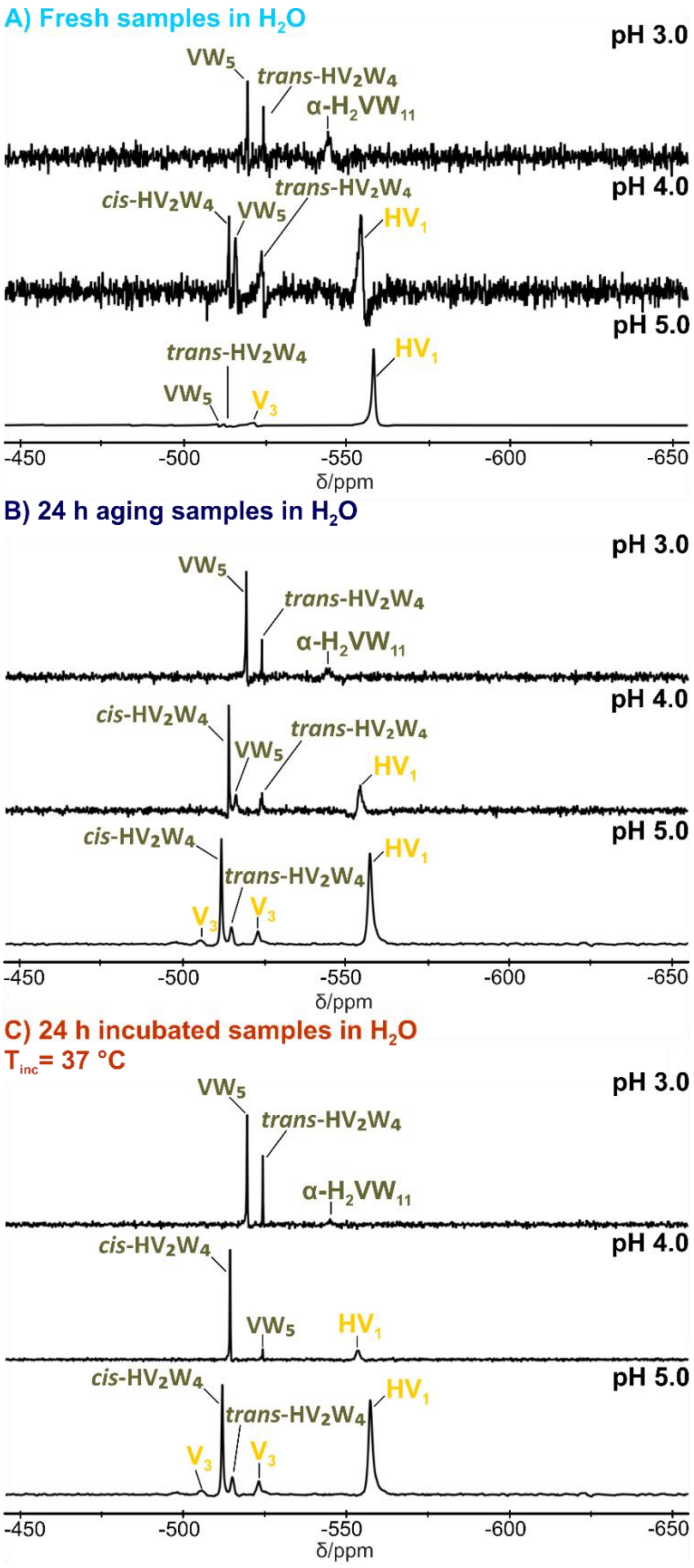

Fig. S46. ^{51}**V NMR spectra of** $\{W_{72}V_{30}\}$ **in** H_2O **(pH 3.0, pH 4.0, and pH 5.0): A) fresh solutions; B) 24 h aging at room temperature, and C) 24 h incubation at 37 °C.**

^{51}V NMR spectra for 0.15 mM fresh solutions (**A**) of $\{W_{72}V_{30}\}$ in H_2O (pH 3.0 – 5.0) that were recorded approximately one hour after preparation. Aliquots of fresh solutions were taken for 24 h aging experiments at room temperature (**B**) and 24 h incubation at 37 °C (**C**) and were then recorded approximately 1 h after the end of 24 h experiments. The structures of all POMs forming during the experiments are shown in **Figures S2 – S3**. The chemical shifts and percentages of parent (if still present) and formed POM species are given in **Tables S20 – S22**.

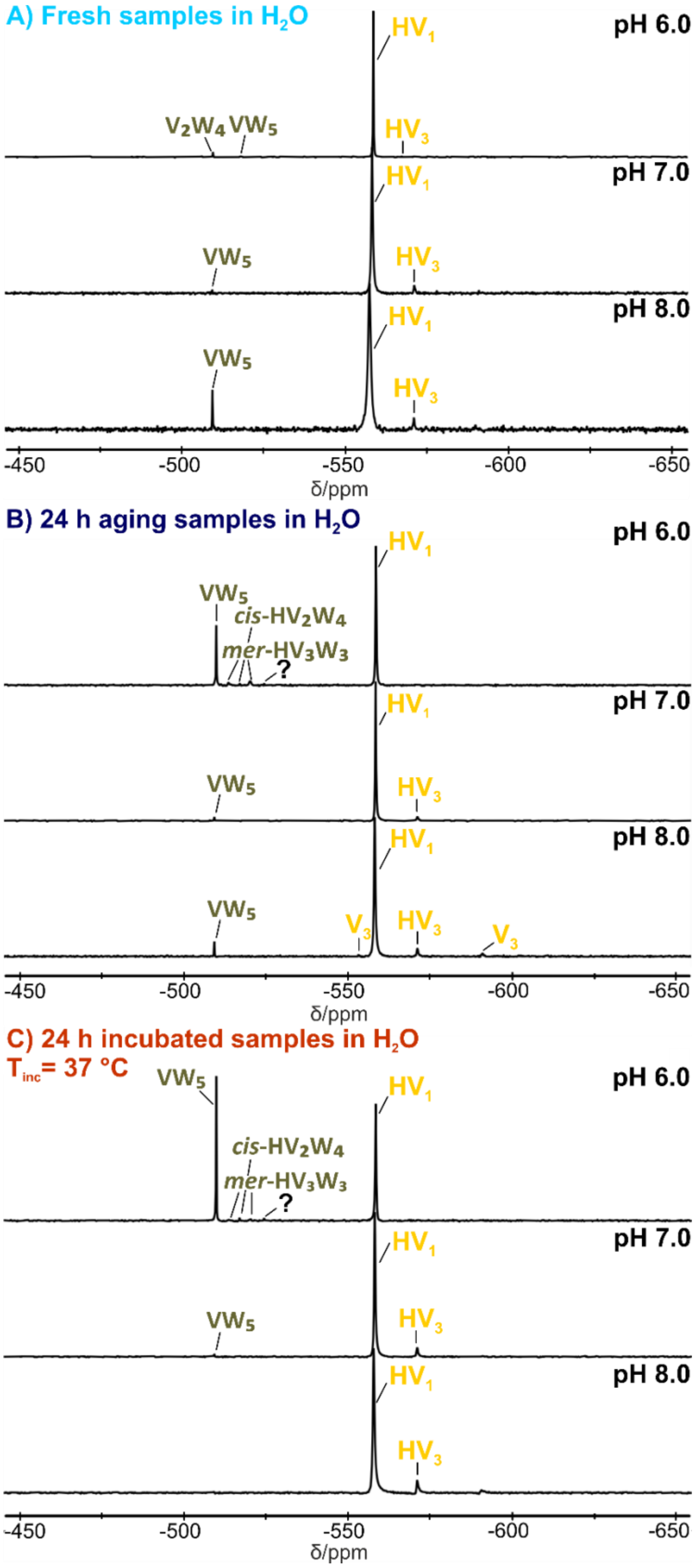

Fig. S47. ^{51}V NMR spectra of {$W_{72}V_{30}$} in H_2O (pH 6.0, pH 7.0, and pH 8.0): A) fresh solutions; B) 24 h aging at room temperature, and C) 24 h incubation at 37 °C.

^{51}V NMR spectra for 0.15 mM fresh solutions (**A**) of {$\mathbf{W_{72}V_{30}}$} in H_2O (pH 6.0 – 8.0) that were recorded approximately one hour after preparation. Aliquots of fresh solutions were taken for 24 h aging experiments at room temperature (**B**) and 24 h incubation at 37 °C (**C**) and were then recorded approximately 1 h after the end of 24 h experiments. The structures of all POMs forming during the experiments are shown in **Figures S2** – **S3**. The chemical shifts and percentages of parent (if still present) and formed POM species are given in **Tables S20** – **S22**.

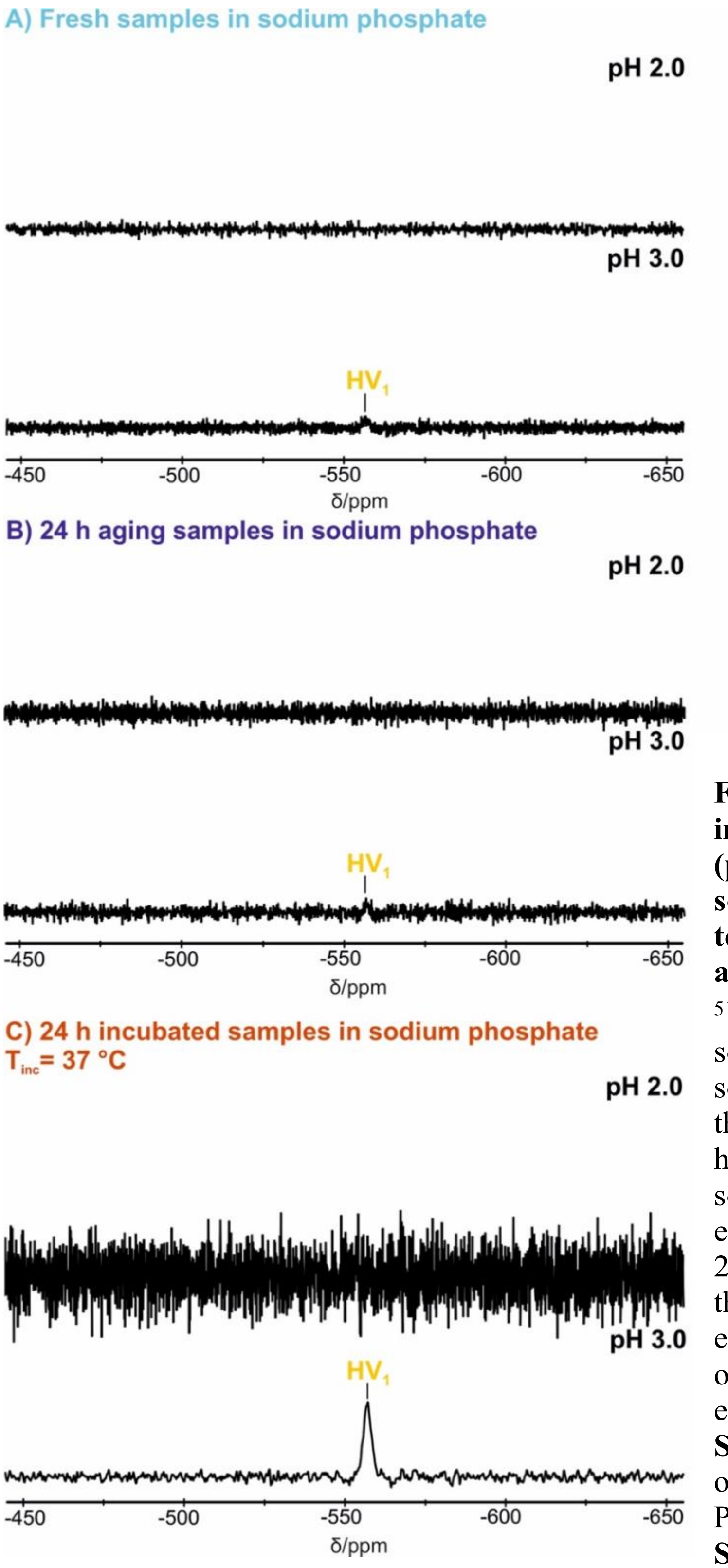

Fig. S48. ^{51}V NMR spectra of {$W_{72}V_{30}$} in 0.1 M sodium phosphate buffers (pH 2.0 and pH 3.0): A) fresh solutions; B) 24 h aging at room temperature, and C) 24 h incubation at 37 °C.

^{51}V NMR spectra for 0.15 mM fresh solutions (**A**) of {$\mathbf{W_{72}V_{30}}$} in 0.1 M sodium phosphate buffers (pH 2.0 – 3.0) that were recorded approximately one hour after preparation. Aliquots of fresh solutions were taken for 24 h aging experiments at room temperature (**B**) and 24 h incubation at 37 °C (**C**) and were then recorded approximately 1 h after the end of 24 h experiments. The structures of all POMs forming during the experiments are shown in **Figures S2 – S3**. The chemical shifts and percentages of parent (if still present) and formed POM species are given in **Table S20 – S22**.

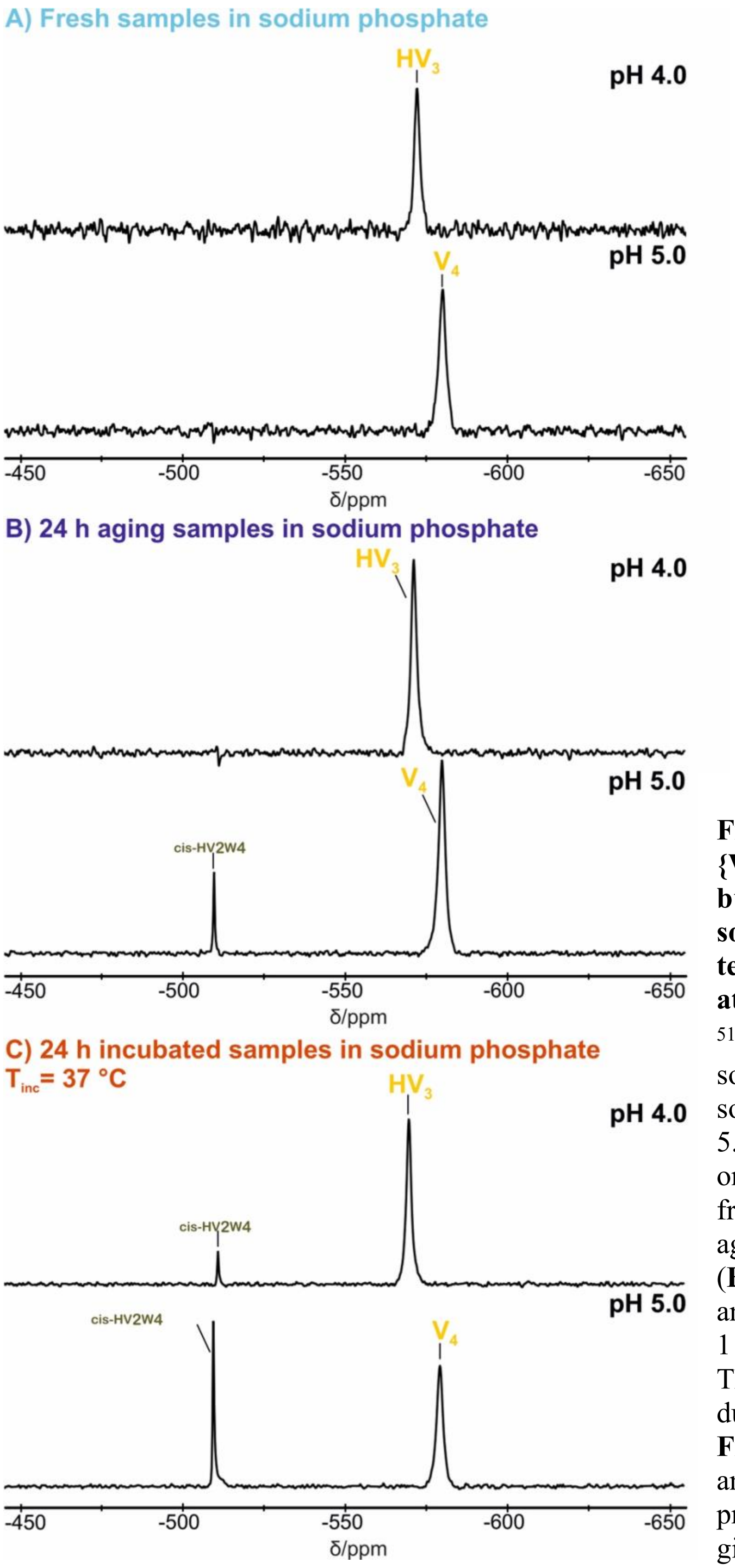

Fig. S49. ^{51}V NMR spectra of {$W_{72}V_{30}$} in 0.1 M sodium phosphate buffers (pH 4.0 and pH 5.0): A) fresh solutions; B) 24 h aging at room temperature, and C) 24 h incubation at 37 °C.

^{51}V NMR spectra for 0.15 mM fresh solutions (**A**) of {**$W_{72}V_{30}$**} in 0.1 M sodium phosphate buffers (pH 4.0 – 5.0) that were recorded approximately one hour after preparation. Aliquots of fresh solutions were taken for 24 h aging experiments at room temperature (**B**) and 24 h incubation at 37 °C (**C**) and were then recorded approximately 1 h after the end of 24 h experiments. The structures of all POMs forming during the experiments are shown in **Figures S2 – S3**. The chemical shifts and percentages of parent (if still present) and formed POM species are given in **Table S20 – S22**.

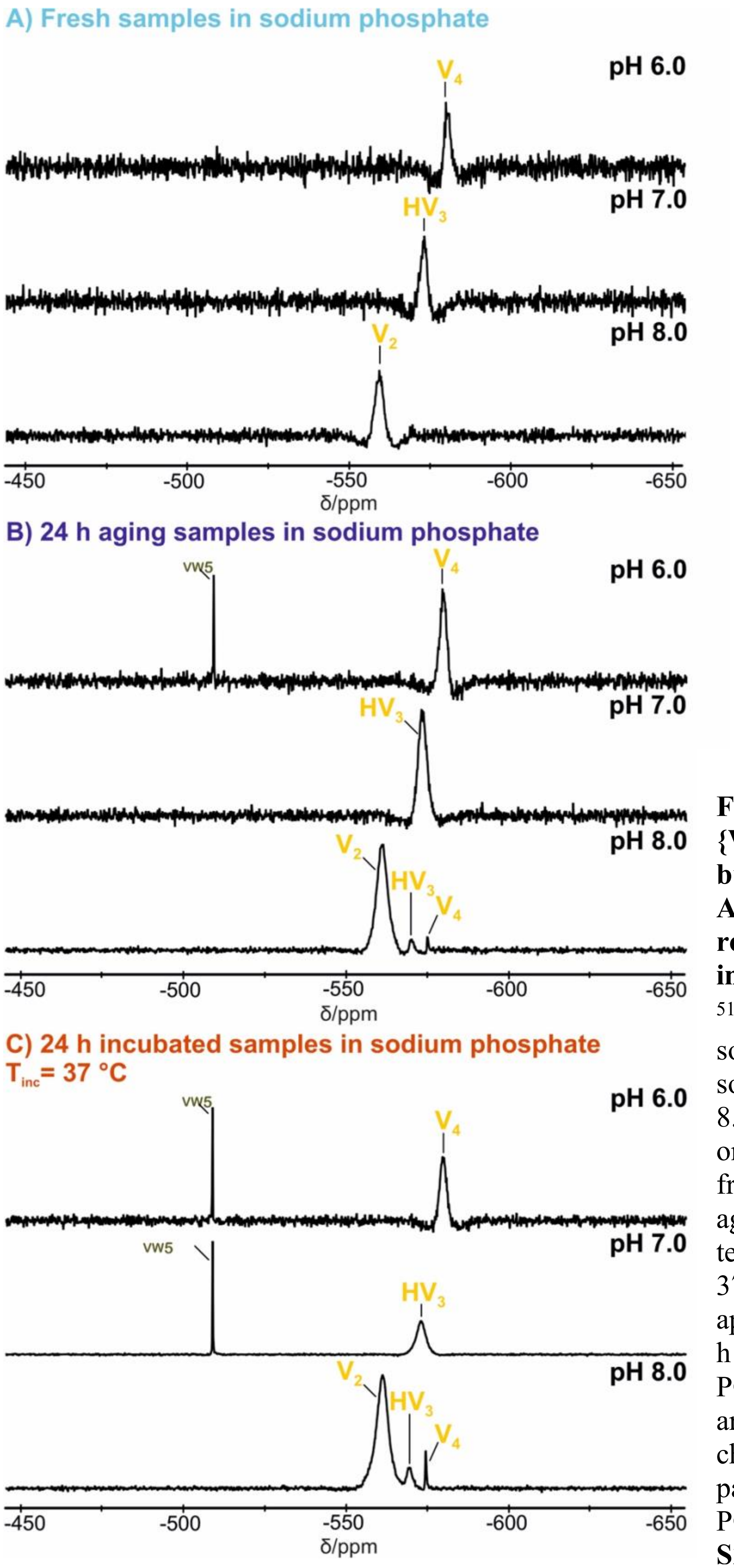

Fig. S50. ^{51}**V NMR spectra of** $\{W_{72}V_{30}\}$ **in 0.1 M sodium phosphate buffers (pH 6.0, pH 7.0 and pH 8.0): A) fresh solutions; B) 24 h aging at room temperature, and C) 24 h incubation at 37 °C.**

^{51}V NMR spectra for 0.15 mM fresh solutions (**A**) of $\{\mathbf{W_{72}V_{30}}\}$ in 0.1 M sodium phosphate buffers (pH 6.0 – 8.0) that were recorded approximately one hour after preparation. Aliquots of fresh solutions were taken for 24 h aging experiments at room temperature (**B**) and 24 h incubation at 37 °C (**C**) and were then recorded approximately 1 h after the end of 24 h experiments. The structures of all POMs forming during the experiments are shown in **Figures S2** – **S3**. The chemical shifts and percentages of parent (if still present) and formed POM species are given in **Table S20** – **S22**.

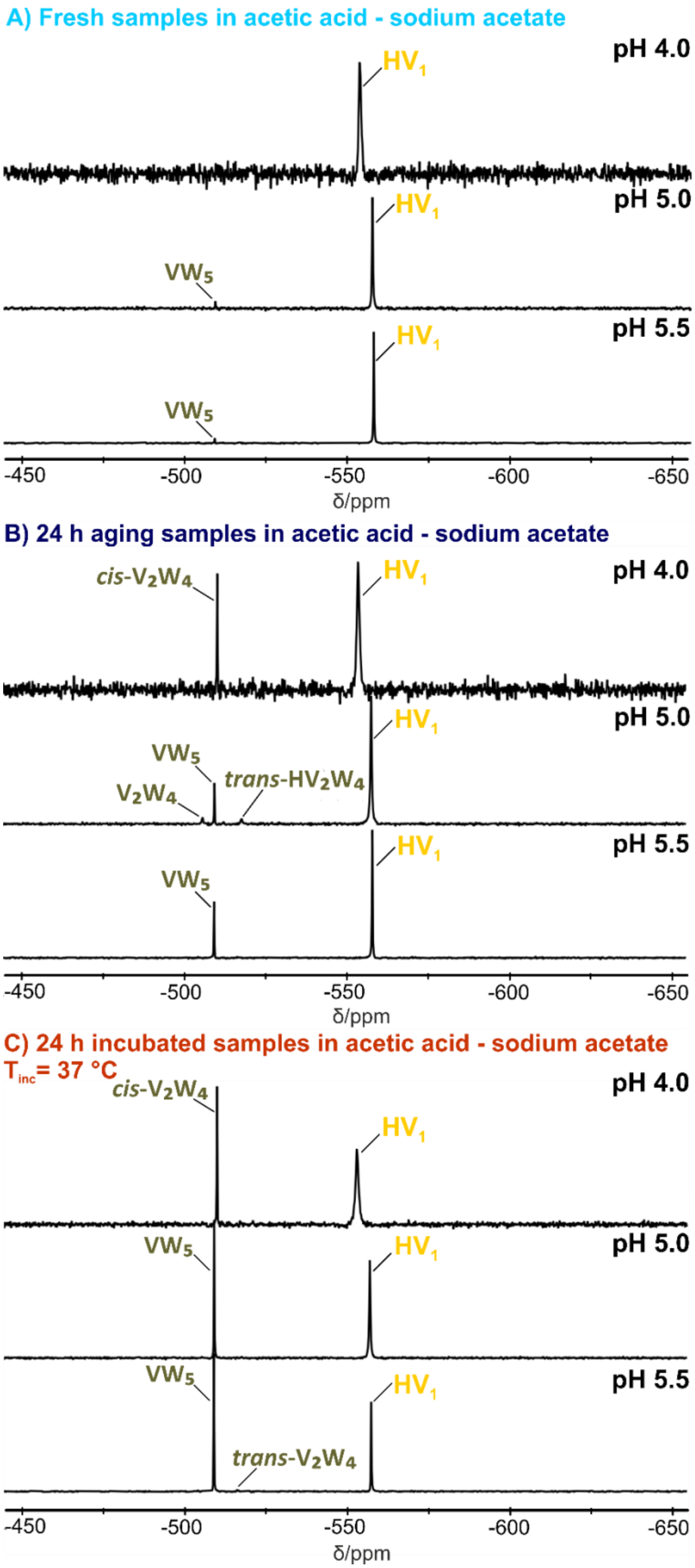

Fig. S51. ^{51}**V NMR spectra of {$W_{72}V_{30}$} in 0.1 M acetic acid – sodium acetate buffers (pH 4.0, pH 5.0 and pH 5.5): A) fresh solutions; B) 24 h aging at room temperature, and C) 24 h incubation at 37 °C.**

^{51}V NMR spectra for 0.15 mM fresh solutions (**A**) of {**$W_{72}V_{30}$**} in 0.1 M acetic acid – sodium acetate buffers (pH 4.0 – 5.5) that were recorded approximately one hour after preparation. Aliquots of fresh solutions were taken for 24 h aging experiments at room temperature (**B**) and 24 h incubation at 37 °C (**C**) and were then recorded approximately 1 h after the end of 24 h experiments. The structures of all POMs forming during the experiments are shown in **Figures S2 – S3**. The chemical shifts and percentages of parent (if still present) and formed POM species are given in **Table S20 – S22**.

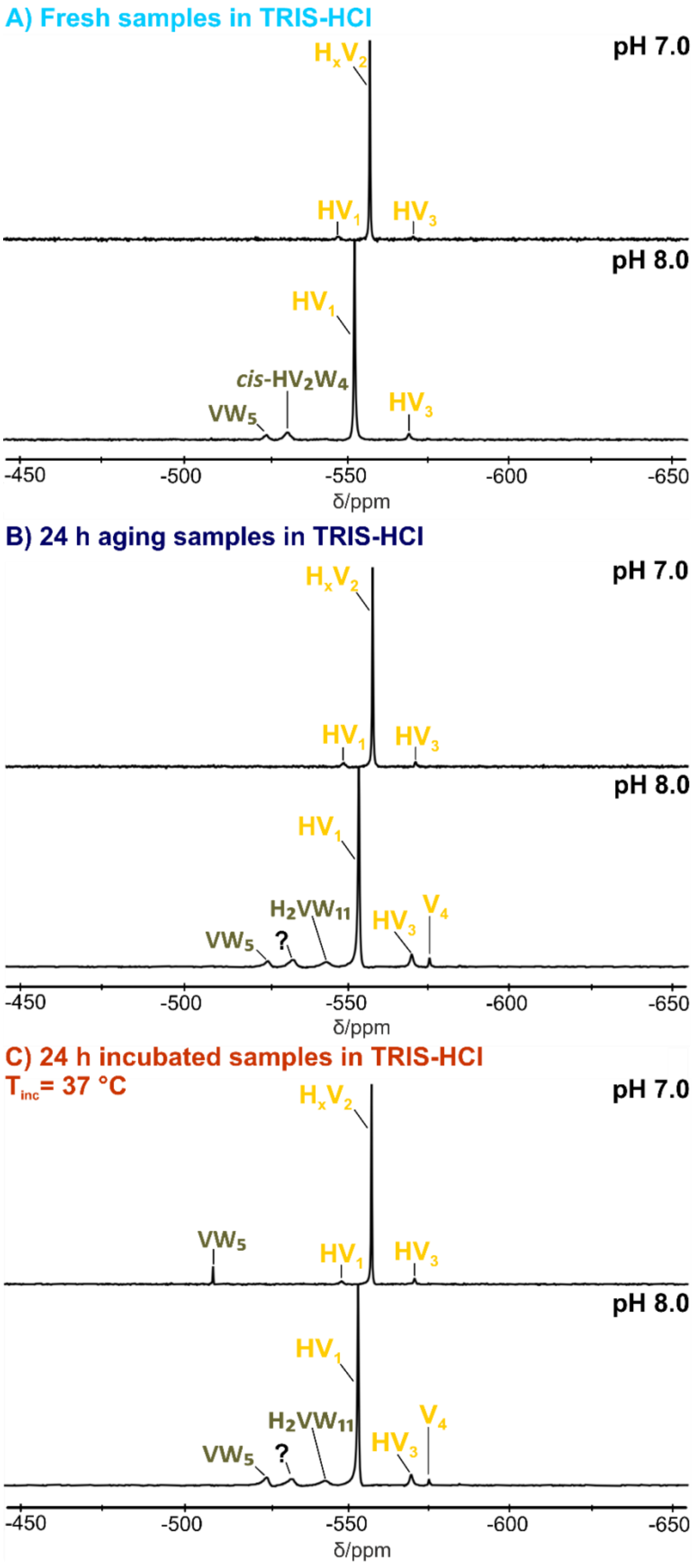

Fig. S52. ^{51}**V NMR spectra of** {$W_{72}V_{30}$} **in 0.1 M Tris-HCl buffers (pH 7.0 and pH 8.0): A) fresh solutions; B) 24 h aging at room temperature, and C) 24 h incubation at 37 °C.**

^{51}V NMR spectra for 0.15 mM fresh solutions (**A**) of {$W_{72}V_{30}$} in 0.1 M Tris-HCl buffers (pH 7.0 – 8.0) that were recorded approximately one hour after preparation. Aliquots of fresh solutions were taken for 24 h aging experiments at room temperature (**B**) and 24 h incubation at 37 °C (**C**) and were then recorded approximately 1 h after the end of 24 h experiments. The structures of all POMs forming during the experiments are shown in **Figures S2 – S3**. The chemical shifts and percentages of parent (if still present) and formed POM species are given in **Table S20 – S22**.

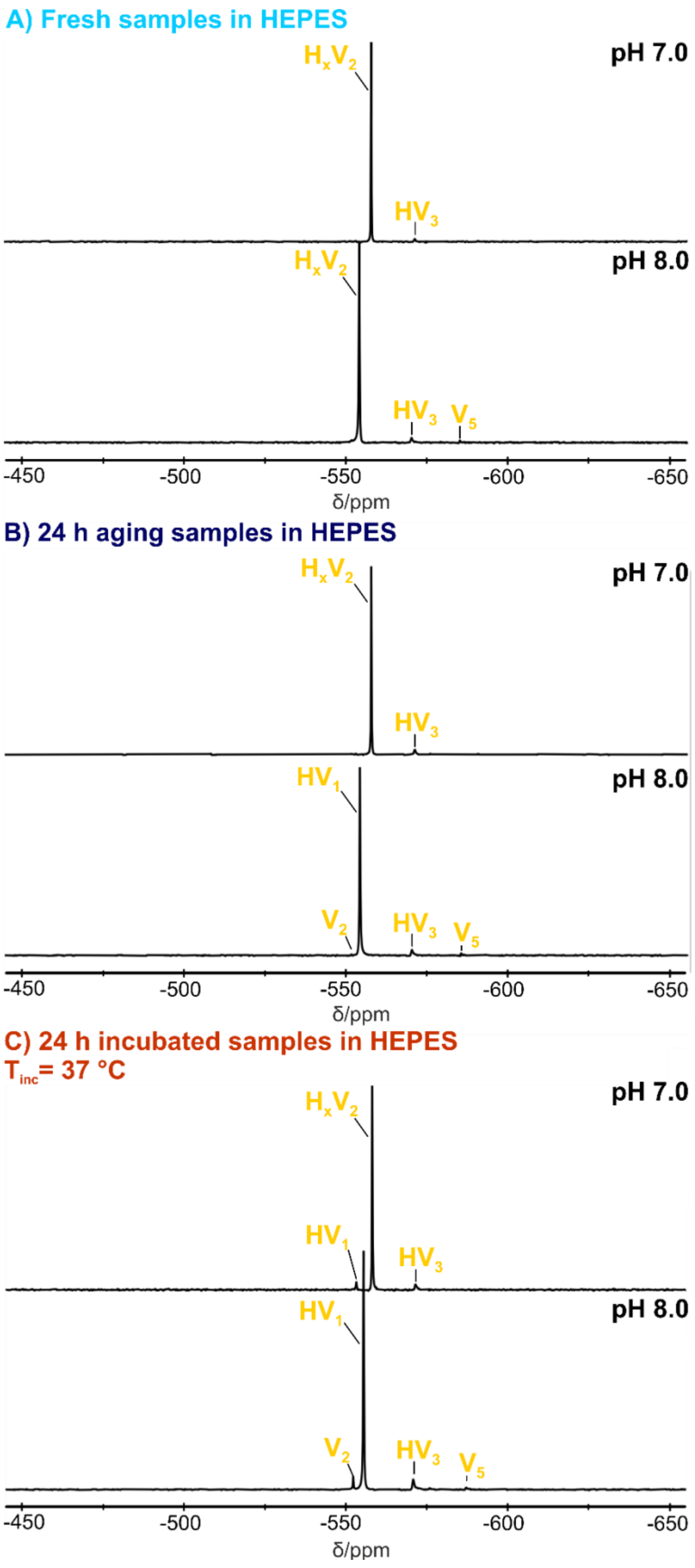

Fig. S53. ^{51}**V NMR spectra of {$W_{72}V_{30}$} in 0.1 M HEPES buffers (pH 7.0 and pH 8.0): A) fresh solutions; B) 24 h aging at room temperature, and C) 24 h incubation at 37 °C.**

^{51}V NMR spectra for 0.15 mM fresh solutions (**A**) of {$\mathbf{W_{72}V_{30}}$} in 0.1 M HEPES buffers (pH 7.0 – 8.0) that were recorded approximately one hour after preparation. Aliquots of fresh solutions were taken for 24 h aging experiments at room temperature (**B**) and 24 h incubation at 37 °C (**C**) and were then recorded approximately 1 h after the end of 24 h experiments. The structures of all POMs forming during the experiments are shown in **Figures S2** – **S3**. The chemical shifts and percentages of parent (if still present) and formed POM species are given in **Table S20** – **S22**.

Table S20. Analysis of NMR spectroscopic data recorded in {$W_{72}V_{30}$} solutions at three different conditions: fresh solutions.

Chemical shifts in ^{51}V NMR spectra measured in triplicate of **{$W_{72}V_{30}$}** (0.15 mM) dissolved in D_2O and 0.1 M buffers (acetic acid – sodium acetate pH 4 – 5.5; sodium phosphate pH 2 – 8 (while phosphate does not buffer at pH range from 3.5 – 5.5, experiments were conducted at this pH to provide comparisons to previously published studies (*239*)); Tris-HCl pH 7 – 8; HEPES pH 7 – 8). The content of species was calculated based on the integration of ^{51}V signals. Signals were assigned based on the literature data summarized in **Table S5**. Percentages are normalized within each pH row and sum to ~100 % (small deviations reflect rounding or trace unassigned signals).

pH	{$W_{72}V_{30}$} (0.15 mM) in Solvent / Buffer	% of all mixed V-W small POMs (Tables S23 – S26) in fresh solution				% of all iso(poly)vanadate (Tables S23 – S26) species in fresh solution			
		Sample			Mean of 1 to 3 ± SD[a]	Sample			Mean of 1 to 3 ± SD[a]
		#1	#2	#3		#1	#2	#3	
1	10% D_2O/H_2O pH 1	no signal detected							
		–	–	–		–	–	–	
1.5	10% D_2O/H_2O pH 1.5	no signal detected							
		–	–	–		–	–	–	
2	10% D_2O/H_2O pH 2	−δ (^{51}V) [ppm]: 524.4				−δ (^{51}V) [ppm]: –			
		100	100	100	100 ± 0.0	–	–	–	
	0.1 M sodium phosphate ($H_2PO_4^-$ /H_3PO_4) pH 2	no signal detected							
		–	–	–		–	–	–	
3	10% D_2O/H_2O pH 3	−δ (^{51}V) [ppm]: 519.6, 524.4, 544.8				−δ (^{51}V) [ppm]: –			
		100	100	100	100 ± 0.0	–	–	–	
	0.1 M sodium phosphate ($H_2PO_4^-$ /H_3PO_4) pH 3	−δ (^{51}V) [ppm]: –				−δ (^{51}V) [ppm]: 557.3			
		–	–	–		100	100	100	100 ± 0.0
4	10% D_2O/H_2O pH 4	−δ (^{51}V) [ppm]: 513.9, 516.0, 524.2				−δ (^{51}V) [ppm]: 554.6			
		36.2	36.1	34.4	35.6 ± 1.0	63.8	63.9	65.6	64.4 ± 1.0
	0.1 M sodium phosphate ($H_2PO_4^-$ /H_3PO_4) pH 4	−δ (^{51}V) [ppm]: –				−δ (^{51}V) [ppm]: 572.2			
		–	–	–		100	100	100	100 ± 0.0
	0.1 M acetic acid – sodium acetate (OAc^- /HOAc) pH 4	−δ (^{51}V) [ppm]: –				−δ (^{51}V) [ppm]: 553.8			
		–	–	–		100	100	100	100 ± 0.0
5	10% D_2O/H_2O pH 5	−δ (^{51}V) [ppm]: 510.2, 512.7				−δ (^{51}V) [ppm]: 521.7, 558.1			
		4.0	3.5	5.5	4.3 ± 1.0	96.0	96.0	94.6	95.5 ± 0.8
	0.1 M sodium phosphate ($H_2PO_4^-$ /H_3PO_4) pH 5	−δ (^{51}V) [ppm]: –				−δ (^{51}V) [ppm]: 580.2			
		–	–	–		100	100	100	100 ± 0.0

	0.1 M acetic acid – sodium acetate (OAc^-/HOAc) pH 5	−δ (^{51}V) [ppm]: 509.4				−δ (^{51}V) [ppm]: 557.8			
		3.3	2.9	3.3	3.1 ± 0.2	96.7	97.1	96.7	96.9 ± 0.2
5.5	0.1 M acetic acid – sodium acetate (OAc^-/HOAc) pH 5.5	−δ (^{51}V) [ppm]: 509.3				−δ (^{51}V) [ppm]: 558.2			
		2.0	2.6	2.5	2.4 ± 0.3	98.0	97.4	97.5	97.6 ± 0.3
6	10% D_2O/H_2O pH 6	−δ (^{51}V) [ppm]: 505.5, 509.3				−δ (^{51}V) [ppm]: 558.2, 570.5			
		2.5	2.5	2.0	2.3 ± 0.3	97.5	97.5	98.0	97.7 ± 0.3
	0.1 M sodium phosphate (HPO_4^{2-}/$H_2PO_4^-$) pH 6	−δ (^{51}V) [ppm]: –				−δ (^{51}V) [ppm]: 580.2			
		–	–	–		100	100	100	100 ± 0.0
7	10% D_2O/H_2O pH 7	−δ (^{51}V) [ppm]: 509.2				−δ (^{51}V) [ppm]: 558.2, 571.0			
		0.5	0.5	0.5	0.5 ± 0.0	99.5	99.5	99.5	99.5 ± 0.0
	0.1 M sodium phosphate (HPO_4^{2-}/$H_2PO_4^-$) pH 7	−δ (^{51}V) [ppm]: –				−δ (^{51}V) [ppm]: 573.6			
		–	–	–		100	100	100	100 ± 0.0
	0.1 M HEPES[c] pH 7	−δ (^{51}V) [ppm]: 571.2				−δ (^{51}V) [ppm]: 557.9			
		2.5	3.0	3.1	2.8 ± 0.3	97.5	97.0	96.9	97.3 ± 0.3
	0.1 M Tris[d]-HCl pH 7	−δ (^{51}V) [ppm]: –				−δ (^{51}V) [ppm]: 548.2, 557.8, 571.0			
		–	–	–		100	100	100	100 ± 0.0
8	10% D_2O/H_2O pH 8	−δ (^{51}V) [ppm]: 509.1				−δ (^{51}V) [ppm]: 558.1, 571.0			
		0	4.5	4.5	3.0 ± 2.6	100	95.50	95.50	97.0 ± 2.6
	0.1 M sodium phosphate (HPO_4^{2-}/$H_2PO_4^-$) pH 8	−δ (^{51}V) [ppm]: –				−δ (^{51}V) [ppm]: 559.5			
		–	–	–		100	100	100	100 ± 0.0
	0.1 M HEPES[c] pH 8	−δ (^{51}V) [ppm]: 570.4				−δ (^{51}V) [ppm]: 554.2, 585.3			
		4.3	4.9	5.2	4.8 ± 0.5	95.7	95.1	94.8	95.2 ± 0.5
	0.1 M Tris[d]-HCl pH 8	−δ (^{51}V) [ppm]: 523.3, 525.8				−δ (^{51}V) [ppm]: 553.1, 569.8			
		15.2	14.7	15.3	15.1 ± 0.3	84.8	85.3	85.0	85.0 ± 0.3

[a]SD – standard deviation; [b]RT- room temperature; [c]HEPES – 4-(2-hydroxyethyl)-1-piperazineethanesulfonic acid, $C_8H_{18}N_2O_4S$; [d]tris – tris(hydroxymethyl)aminomethane, $C_4H_{11}NO_3$.

Table S21. Analysis of NMR spectroscopic data recorded in $\{W_{72}V_{30}\}$ solutions at three different conditions: 24 h at room temperature.

Chemical shifts in ^{51}V NMR spectra measured in triplicate of **$\{W_{72}V_{30}\}$** (0.15 mM) dissolved in D_2O and 0.1 M buffers (acetic acid – sodium acetate pH 4 – 5.5; sodium phosphate pH 2 – 8 (while phosphate does not buffer at pH range from 3.5 – 5.5, experiments were conducted at this pH to provide comparisons to previously published studies (*239*)); Tris-HCl pH 7 – 8; HEPES pH 7 – 8) and measured after 24 h kept at room temperature. The content of species was calculated based on the integration of ^{51}V signals. Signals were assigned based on the literature data summarized in **Table S5**. Percentages are normalized within each pH row and sum to ~100 % (small deviations reflect rounding or trace unassigned signals).

pH	$\{W_{72}V_{30}\}$ (0.15 mM) in Solvent / Buffer	% of all mixed V-W small POMs (Tables S23 – S26) or/and *unassigned* species in solution after 24 h at RT				% of all iso(poly)vanadate (Tables S23 – S26) species after 24 h at RT			
		Sample #1	Sample #2	Sample #3	Mean of 1 to 3 ± SD[a]	Sample #1	Sample #2	Sample #3	Mean of 1 to 3 ± SD[a]
1	10% D_2O/H_2O pH 1	−δ (^{51}V) [ppm]: 524.4				−δ (^{51}V) [ppm]: –			
		100	100	100	100 ± 0.0	–	–	–	
1.5	10% D_2O/H_2O pH 1.5	−δ (^{51}V) [ppm]: 524.4				−δ (^{51}V) [ppm]: –			
		100	100	100	100 ± 0.0	–	–	–	
2	10% D_2O/H_2O pH 2	−δ (^{51}V) [ppm]: 524.4				−δ (^{51}V) [ppm]: –			
		100	100	100	100 ± 0.0	–	–	–	
	0.1 M sodium phosphate ($H_2PO_4^-$/H_3PO_4) pH 2	no signal detected							
		–	–	–		–	–	–	
3	10% D_2O/H_2O pH 3	−δ (^{51}V) [ppm]: 519.6, 524.4, 544.5				−δ (^{51}V) [ppm]: –			
		100	100	100	100 ± 0.0	–	–	–	
	0.1 M sodium phosphate ($H_2PO_4^-$/H_3PO_4) pH 3	−δ (^{51}V) [ppm]: –				−δ (^{51}V) [ppm]: 557.1			
		–	–	–		100	100	100	100 ± 0.0
4	10% D_2O/H_2O pH 4	−δ (^{51}V) [ppm]: 514.2, 516.2, 524.4				−δ (^{51}V) [ppm]: 554.1			
		48.8	47.9	47.6	48.1 ± 0.6	51.2	52.1	52.5	51.9 ± 0.7
	0.1 M sodium phosphate ($H_2PO_4^-$/H_3PO_4) pH 4	−δ (^{51}V) [ppm]:				−δ (^{51}V) [ppm]: 572.1			
		–	–	–		100	100	100	100 ± 0.0
	0.1 M acetic acid – sodium acetate (OAc^-/HOAc) pH 4	−δ (^{51}V) [ppm]: 510.4				−δ (^{51}V) [ppm]: 553.9			
		14.9	16.2	16.3	15.8 ± 0.8	85.1	83.8	83.7	84.2 ± 0.8
5	10% D_2O/H_2O pH 5	−δ (^{51}V) [ppm]: 511.4, 514.2				−δ (^{51}V) [ppm]: 505.5, 522.5, 557.5			
		12.5	10.4	8.5	10.5 ± 2.0	87.5	89.3	91.4	89.4 ± 2.0
		−δ (^{51}V) [ppm]: 509.5				−δ (^{51}V) [ppm]: 579.9			

	0.1 M sodium phosphate ($H_2PO_4^-$/H_3PO_4) pH 5	9.4	9.0	9.8	9.4 ± 0.4	90.6	91.0	90.2	90.6 ± 0.4
	0.1 M acetic acid – sodium acetate (OAc^-/HOAc) pH 5	−δ (^{51}V) [ppm]: 505.7, 509.4, 517.8				−δ (^{51}V) [ppm]: 557.8			
		16.5	17.5	15.7	16.8 ± 0.9	83.5	82.5	84.3	83.4 ± 0.9
5.5	0.1 M acetic acid – sodium acetate (OAc^-/HOAc) pH 5.5	−δ (^{51}V) [ppm]: 509.4				−δ (^{51}V) [ppm]: 558.2			
		23.3	24.8	25.8	24.6 ± 1.3	76.8	75.3	74.3	75.4 ± 1.3
6	10% D_2O/H_2O pH 6	−δ (^{51}V) [ppm]: 509.7, 512.4, 516.8, 519.9				−δ (^{51}V) [ppm]: 558.4			
		23.4	25.5	24.9	24.6 ± 1.1	76.4	74.4	75.0	75.4 ± 1.4
		unassigned −δ (^{51}V) [ppm]: 524.4							
		0.2	0.1	0.3	0.2 ± 0.1				
	0.1 M sodium phosphate (HPO_4^{2-}/$H_2PO_4^-$) pH 6	−δ (^{51}V) [ppm]: 509.5				−δ (^{51}V) [ppm]: 580.4			
		5.7	5.6	5.8	5.7 ± 0.1	94.3	94.4	94.2	94.3 ± 0.1
7	10% D_2O/H_2O pH 7	−δ (^{51}V) [ppm]: 509.2				−δ (^{51}V) [ppm]: 558.3, 571.0			
		4.5	0.5	1	2 ± 2.2	95.5	99.5	99.0	98.0 ± 2.2
	0.1 M sodium phosphate (HPO_4^{2-}/$H_2PO_4^-$) pH 7	−δ (^{51}V) [ppm]: –				−δ (^{51}V) [ppm]: 573.3			
		–	–	–		100	100	100	100 ± 0.0
	0.1 M HEPES[c] pH 7	−δ (^{51}V) [ppm]: –				−δ (^{51}V) [ppm]: 557.9, 571.3			
		–	–	–		100	100	100	100 ± 0.0
	0.1 M Tris[d]-HCl pH 7	−δ (^{51}V) [ppm]: –				−δ (^{51}V) [ppm]: 549.0, 557.9, 571.0			
		–	–	–		100	100	100	100 ± 0.0
8	10% D_2O/H_2O pH 8	−δ (^{51}V) [ppm]: 509.1				−δ (^{51}V) [ppm]: 558.1, 571.1, 591.1			
		1.0	3.1	3.3	2.5 ± 1.3	99.0	96.9	96.7	97.5 ± 1.3
	0.1 M sodium phosphate (HPO_4^{2-}/$H_2PO_4^-$) pH 8	−δ (^{51}V) [ppm]: –				−δ (^{51}V) [ppm]: 561.0, 570.1, 575.1			
		–	–	–		100	100	100	100 ± 0.0
	0.1 M HEPES[c] pH 8	−δ (^{51}V) [ppm]: –				−δ (^{51}V) [ppm]: 551.7, 554.2, 570.4, 585.4			
		–	–	–		100	100	100	100 ± 0.0
	0.1 M Tris[d]-HCl pH 8	−δ (^{51}V) [ppm]: 525.6				−δ (^{51}V) [ppm]: 543.6, 553.5, 570.0, 575.4			
		11.9	12.5	12.0	12.1 ± 0.3	87.3	87.0	86.9	87.1 ± 0.2
		unassigned −δ (^{51}V) [ppm]: 533.3							
		0.8	0.5	1.1	0.8 ± 0.2				

[a]SD – standard deviation; [b]RT- room temperature; [c]HEPES – 4-(2-hydroxyethyl)-1-piperazineethanesulfonic acid, $C_8H_{18}N_2O_4S$; [d]tris – tris(hydroxymethyl)aminomethane, $C_4H_{11}NO_3$. Iso(poly)vanadates include V_{10} and V_9Mo_1 decavanadate species.

Table S22. Analysis of NMR spectroscopic data recorded in $\{W_{72}V_{30}\}$ solutions at three different conditions: 24 h incubation at 37 °C.

Chemical shifts in ^{51}V NMR spectra measured in triplicate of **$\{W_{72}V_{30}\}$** (0.15 mM) dissolved in D_2O and 0.1 M buffers (acetic acid – sodium acetate pH 4 –5 .5; sodium phosphate pH 2 – 8 (while phosphate does not buffer at pH range from 3.5 – 5.5, experiments were conducted at this pH to provide comparisons to previously published studies (*239*)); Tris-HCl pH 7 – 8; HEPES pH 7 – 8) and measured after 24 h kept at 37 °C. The content of species was calculated based on the integration of ^{51}V signals. Signals were assigned based on the literature data summarized in **Table S5**. Percentages are normalized within each pH row and sum to ~100 % (small deviations reflect rounding or trace unassigned signals).

pH	$\{W_{72}V_{30}\}$ (0.15 mM) in Solvent / Buffer	% of all mixed V-W small POMs (Tables S23 – S26) or/and *unassigned* species in solution after 24 h incubation at 37 °C				% of all iso(poly)vanadate (Tables S23 – S26) species after 24 h incubation at 37 °C			
		Sample			Mean of 1 to 3 ± SD[a]	Sample			Mean of 1 to 3 ± SD[a]
		#1	#2	#3		#1	#2	#3	
1	10% D_2O/H_2O pH 1	−δ (^{51}V) [ppm]: 524.4				−δ (^{51}V) [ppm]: 555.7, 558.0, 561.5			
		90.8	92.6	88.6	90.7 ± 2.0	9.2	7.4	11.3	9.3 ± 2.0
1.5	10% D_2O/H_2O pH 1.5	−δ (^{51}V) [ppm]: 524.4				−δ (^{51}V) [ppm]: –			
		100	100	100	100 ± 0.0	–	–	–	
2	10% D_2O/H_2O pH 2	−δ (^{51}V) [ppm]: 524.4				−δ (^{51}V) [ppm]: –			
		100	100	100	100 ± 0.0	–	–	–	
	0.1 M sodium phosphate ($H_2PO_4^-$/H_3PO_4) pH 2	no signal detected							
		–	–	–		–	–	–	
3	10% D_2O/H_2O pH 3	−δ (^{51}V) [ppm]: 519.6, 524.4, 544.5				−δ (^{51}V) [ppm]: –			
		100	100	100	100 ± 0.0	–	–	–	
	0.1 M sodium phosphate ($H_2PO_4^-$/H_3PO_4) pH 3	−δ (^{51}V) [ppm]: –				−δ (^{51}V) [ppm]: 557.1			
		–	–	–		100	100	100	100 ± 0.0
4	10% D_2O/H_2O pH 4	−δ (^{51}V) [ppm]: 514.4, 524.4				−δ (^{51}V) [ppm]: 553.7			
		61.7	63.3	62.1	63.5 ± 1.9	38.3	36.7	37.9	37.6 ± 0.8
	0.1 M sodium phosphate ($H_2PO_4^-$/H_3PO_4) pH 4	−δ (^{51}V) [ppm]: 510.9				−δ (^{51}V) [ppm]: 570.3			
		5.0	5.4	5.3	5.2 ± 0.2	95.0	94.6	94.7	94.8 ± 0.2
	0.1 M acetic acid – sodium acetate (OAc^-/HOAc) pH 4	−δ (^{51}V) [ppm]: 510.4				−δ (^{51}V) [ppm]: 553.8			
		25.8	26	26.5	26.1 ± 0.4	74.3	74	73.5	73.9 ± 0.4
5	10% D_2O/H_2O pH 5	−δ (^{51}V) [ppm]: 511.9, 515.2				−δ (^{51}V) [ppm]: 505.6, 523.2, 557.3			
		32.0	31.1	30.0	31.0 ± 1.0	68.0	69.0	70.1	69.0 ± 1.1
		−δ (^{51}V) [ppm]: 509.6				−δ (^{51}V) [ppm]: 579.8			

	0.1 M sodium phosphate ($H_2PO_4^-$/H_3PO_4) pH 5	28.2	28.0	27.9	28.0 ± 0.2	71.8	72.0	72.1	72.0 ± 0.2
	0.1 M acetic acid – sodium acetate (OAc^-/HOAc) pH 5	$-\delta$ (^{51}V) [ppm]: 509.4				$-\delta$ (^{51}V) [ppm]: 557.8			
		39.7	38.5	39.0	39.1 ± 0.6	60.3	61.5	61.0	60.9 ± 0.6
5.5	0.1 M acetic acid – sodium acetate (OAc^-/HOAc) pH 5.5	$-\delta$ (^{51}V) [ppm]: 509.4, 516.5				$-\delta$ (^{51}V) [ppm]: 558.2			
		57.8	58.1	59.0	58.3 ± 0.6	42.2	41.9	41.0	41.7 ± 0.6
6	10% D_2O/H_2O pH 6	$-\delta$ (^{51}V) [ppm]: 509.7, 516.9, 519.9				$-\delta$ (^{51}V) [ppm]: 558.4			
		37.1	39.4	40.2	38.9 ± 1.6	62.7	60.4	59.6	60.9 ± 1.6
		unassigned $-\delta$ (^{51}V) [ppm]: 524.4							
		0.4	0.4	0.4	0.4 ± 0.0				
	0.1 M sodium phosphate (HPO_4^{2-}/$H_2PO_4^-$) pH 6	$-\delta$ (^{51}V) [ppm]: 509.5				$-\delta$ (^{51}V) [ppm]: 580.4			
		8.9	8.7	9.6	9.1 ± 0.5	91.1	91.3	90.4	90.9 ± 0.5
7	10% D_2O/H_2O pH 7	$-\delta$ (^{51}V) [ppm]: 509.1				$-\delta$ (^{51}V) [ppm]: 558.3, 571			
		15.2	0.5	0.4	5.4 ± 8.5	84.8	99.6	99.5	94.6 ± 8.5
	0.1 M sodium phosphate (HPO_4^{2-}/$H_2PO_4^-$) pH 7	$-\delta$ (^{51}V) [ppm]: 509.3				$-\delta$ (^{51}V) [ppm]: 573.6			
		18.0	19.0	19.6	18.9 ± 0.8	82.0	81.0	80.4	81.1 ± 0.8
	0.1 M HEPES[c] pH 7	$-\delta$ (^{51}V) [ppm]: –				$-\delta$ (^{51}V) [ppm]: 553.2, 558.1, 571.4			
		–	–	–		100	100	100	100 ± 0.0
	0.1 M Tris[d]-HCl pH 7	$-\delta$ (^{51}V) [ppm]: 509.1				$-\delta$ (^{51}V) [ppm]: 548.6, 557.8, 571.0			
		4.5	5	5.1	4.9 ± 0.3	95.6	94.9	94.9	95.1 ± 0.4
8	10% D_2O/H_2O pH 8	$-\delta$ (^{51}V) [ppm]: –				$-\delta$ (^{51}V) [ppm]: 557.7, 571.0, 590.5			
		–	–	–		100	100	100	100 ± 0.0
	0.1 M sodium phosphate (HPO_4^{2-}/$H_2PO_4^-$) pH 8	$-\delta$ (^{51}V) [ppm]:				$-\delta$ (^{51}V) [ppm]: 561.6, 569.8, 575.3			
		–	–	–		100	100	100	100 ± 0.0
	0.1 M HEPES[c] pH 8	$-\delta$ (^{51}V) [ppm]: –				$-\delta$ (^{51}V) [ppm]: 557.3, 555.5, 570.5, 586.5			
		–	–	–		100	100	100	100 ± 0.0
	0.1 M Tris[d]-HCl pH 8	$-\delta$ (^{51}V) [ppm]: 525.5, 543.7				$-\delta$ (^{51}V) [ppm]: 553.8, 570.1, 575.5			
		17.8	17.8	17.5	17.2 ± 0.4	82.9	82.4	81.7	82.3 ± 0.6
		unassigned $-\delta$ (^{51}V) [ppm]: 533.3							
		0	0.7	0.8	0.5 ± 0.4				

[a]SD – standard deviation; [b]RT- room temperature; [c]HEPES – 4-(2-hydroxyethyl)-1-piperazineethanesulfonic acid, $C_8H_{18}N_2O_4S$; [d]tris – tris(hydroxymethyl)aminomethane, $C_4H_{11}NO_3$. Iso(poly)vanadates include V_{10} and V_9Mo_1 decavanadate species

Table S23. Overview of W-V and V-based species detected in solution by ^{51}V NMR (based on NMR data in Tables S20 – S22) after dissolution of $\{W_{72}V_{30}\}$ Keplerate in H_2O with pH 1 – 8 at three different conditions: fresh solutions, 24 h at room temperature, and 24 h at 37 °C. Percentages are normalized within each pH row and sum to ~100 % (small deviations reflect rounding or trace unassigned signals).

POM anion structure	solutions	pH 1	pH 1.5	pH 2	pH 3	pH 4	pH 5	pH 6	pH 7	pH 8
$[V^{V}W^{VI}_5O_{19}]^{3-}$	fresh	-	-	100 ± 0.0 %	9.1 ± 1.0 %	13.6 ± 1.1 %	2.7 ± 0.8 %	1.7 ± 0.3 %	0.5 ± 0.1 %	3.0 ± 2.6 %
	RT	100 ± 0.0 %	100 ± 0.0 %	100 ± 0.0 %	9.9 ± 2.3 %	8.9 ± 0.1 %	-	20.8 ± 1.4 %	3.7 ± 0.7 %	2.8 ± 0.7 %
	inc.	90.7 ± 2.0 %	100 ± 0.0 %	100 ± 0.0 %	22.5 ± 0.4 %	3.6 ± 0.1 %	-	38.3 ± 1.6 %	10.4 ± 2.9 %	-
cis-$[V^{V}_2W^{VI}_4O_{19}]^{4-}$	fresh	-	-	-	-	7.3 ± 0.3 %	-	-	-	-
	RT	-	-	-	-	28.0 ± 1.3 %	3.3 ± 0.7 %	0.2 ± 0.1 %	-	-
	inc.	-	-	-	-	60.8 ± 1.9 %	26.2 ± 0.6 %	0.2 ± 0.0 %	-	-
trans-$[V^{V}_2W^{VI}_4O_{19}]^{4-}$	fresh	-	-	-	22.7 ± 4.4 %	14.6 ± 0.8 %	1.7 ± 0.3 %	-	-	-
	RT	-	-	-	53.4 ± 0.5 %	11.2 ± 0.8 %	7.2 ± 0.6 %	-	-	-
	inc.	-	-	-	59.8 ± 1.4 %	-	4.8 ± 1.0 %	-	-	-
$[V^{V}_2W^{VI}_4O_{19}]^{4-}$	fresh	-	-	-	-	-	-	0.7 ± 0.3 %	-	-
	RT	-	-	-	-	-	-	-	-	-
	inc.	-	-	-	-	-	-	-	-	-
mer-$[HV^{V}_3W^{VI}_3O_{19}]^{4-}$	fresh	-	-	-	-	-	-	-	-	-
	RT	-	-	-	-	-	-	3.6 ± 0.5 %	-	-
	inc.	-	-	-	-	-	-	0.4 ± 0.0 %	-	-
$[H_2V^{V}W^{VI}_{11}O_{40}]^{7-}$	fresh	-	-	-	68.2 ± 4.7 %	-	-	-	-	-
	RT	-	-	-	36.7 ± 2.1 %	-	-	-	-	-
	inc.	-	-	-	17.7 ± 1.1 %	-	-	-	-	-
$[V^{V}_3O_{10}]^{5-}$	fresh	-	-	-	-	-	5.2 ± 0.8 %	-	-	-
	RT	-	-	-	-	-	11.8 ± 0.6 %	-	-	91.9 ± 0.6 %
	inc.	-	-	-	-	-	6.2 ± 1.0 %	-	-	-

**$[HV^{V}_{3}O_{10}]^{4-}$**	fresh	-	-	-	-	-	-	0.7 ± 0.3 %	3.4 ± 0.9 %	3.8 ± 0.6 %
	RT	-	-	-	-	-	-	-	3.2 ± 0.3 %	5.3 ± 0.1 %
	inc.	-	-	-	-	-	-	-	5.8 ± 0.8 %	1.6 ± 0.5 %
**$[V^{V}_{2}O_{7}]^{4-}$**	fresh	-	-	-	-	-	-	-	-	-
	RT	-	-	-	-	-	-	-	-	-
	inc.	5.9 ± 1.5 %	-	-	-	-	-	-	-	-
**$[H_{x}V_{2}O_{7}]^{x-4}$**	fresh	-	-	-	-	-	90.4 ± 1.0 %	97.0 ± 0.5 %	96.1 ± 0.9 %	93.2 ± 2.0 %
	RT	-	-	-	-	-	77.6 ± 1.6 %	75.4 ± 1.4 %	93.1 ± 0.7 %	-
	inc.	3.4 ± 0.4 %	-	-	-	-	62.8 ± 2.0 %	60.9 ± 1.6 %	83.9 ± 2.3 %	97.8 ± 1.0 %
**$[H_{x}VO_{4}]^{x-3}$ x=0-2**	fresh	-	-	-	-	64.4 ± 1.0 %	-	-	-	-
	RT	-	-	-	-	51.9 ± 0.7 %	-	-	-	-
	inc.	-	-	-	-	35.6 ± 0.8 %	-	-	-	-

Table S24. Overview of W-V and V-based species detected in solution by ^{51}V NMR (based on NMR data in Tables S20 – S22) after dissolution of $\{W_{72}V_{30}\}$ Keplerate in 0.1 M sodium phosphate buffers with pH 2 – 8 at three different conditions: fresh solutions, 24 h at room temperature, and 24 h at 37 °C. Percentages are normalized within each pH row and sum to ~100 % (small deviations reflect rounding or trace unassigned signals).

POM anion structure	solutions	pH 2	pH 3	pH 4	pH 5	pH 6	pH 7	pH 8
**$[V^{V}W^{VI}_{5}O_{19}]^{3-}$**	fresh	-	-	-	-	-	-	-
	RT	-	-	-	9.4 ± 0.4 %	5.7 ± 0.1 %	-	-
	inc.	-	-	-	28.0 ± 0.2 %	9.1 ± 0.5 %	18.9 ± 0.8 %	-
***cis*-$[V^{V}_{2}W^{VI}_{4}O_{19}]^{4-}$**	fresh	-	-	-	-	-	-	-
	RT	-	-	-	-	-	-	-
	inc.	-	-	5.2 ± 0.2 %	-	-	-	-
**$[V^{V}_{4}O_{12}]^{4-}$**	fresh	-	-	-	100 ± 0.0 %	100 ± 0.0 %	-	-
	RT	-	-	-	90.6 ± 0.4 %	94.3 ± 0.1 %	-	1.3 ± 0.3 %
	inc.	-	-	-	72.0 ± 0.2 %	90.9 ± 0.5 %	-	2.4 ± 0.2 %

**$[H_xV^{V}{}_3O_{10}]^{5-x}$ x=1-2**	fresh	-	-	100 ± 0.0 %	-	-	100 ± 0.0 %	-
	RT	-	-	100 ± 0.0 %	-	-	100 ± 0.0 %	1.7 ± 0.8 %
	inc.	-	-	94.8 ± 0.2 %	-	-	81.1 ± 0.8 %	6.1 ± 1.1 %
**$[V^{V}{}_2O_7]^{4-}$**	fresh	-	-	-	-	-	-	100 ± 0.0 %
	RT	-	-	-	-	-	-	97.0 ± 1.0 %
	inc.	-	-	-	-	-	-	91.4 ± 1.3 %
**$[H_xV_2O_7]^{x-4}$**	fresh	-	-	-	-	-	-	-
	RT	-	-	-	-	-	-	-
	inc.	-	-	-	-	-	-	-
**$[H_xVO_4]^{x-3}$ x=0-2**	fresh	-	100 ± 0.0 %	-	-	-	-	-
	RT	-	100 ± 0.0 %	-	-	-	-	-
	inc.	-	100 ± 0.0 %	-	-	-	-	-

Table S25. Overview of W-V and V-based species detected in solution by ^{51}V NMR (based on NMR data in Tables S20 – S22) after dissolution of $\{W_{72}V_{30}\}$ Keplerate in 0.1 M acetic acid – sodium acetate buffers with pH 4 – 5.5 at three different conditions: fresh solutions, 24 h at room temperature, and 24 h at 37 °C. Percentages are normalized within each pH row and sum to ~100 % (small deviations reflect rounding or trace unassigned signals).

**POM anion structure**	**solutions**	**pH 4**	**pH 5**	**pH 5.5**
**$[V^{V}W^{VI}{}_5O_{19}]^{3-}$**	fresh	-	3.1 ± 0.2 %	2.4 ± 0.3 %
	RT	-	10.7 ± 0.9 %	24.6 ± 1.3 %
	inc.	-	39.1 ± 0.6 %	57.3 ± 0.8 %
***cis*-$[V^{V}{}_2W^{VI}{}_4O_{19}]^{4-}$**	fresh	-	-	-
	RT	15.8 ± 0.8 %	-	-
	inc.	26.1 ± 0.4 %	-	1.0 ± 0.2 %
***trans*-$[V^{V}{}_2W^{VI}{}_4O_{19}]^{4-}$**	fresh	-	-	-
	RT	-	3.0 ± 0.5 %	-
	inc.	-	-	-

$[V^{V}_{2}W^{VI}_{4}O_{19}]^{4-}$	fresh	-	-	-
	RT	-	2.9 ± 0.4 %	-
	inc.	-	-	-
$[H_xVO_4]^{x-3}$ x=0–2	fresh	100 ± 0.0 %	96.9 ± 0.2 %	97.6 ± 0.3 %
	RT	84.2 ± 0.8 %	83.4 ± 0.9 %	75.4 ± 1.3 %
	inc.	73.9 ± 0.4 %	60.9 ± 0.6 %	41.7 ± 0.6 %

Table S26. Overview of W-V and V-based species detected in solution by ^{51}V NMR (based on NMR data in Tables S20 – S22) after dissolution of $\{W_{72}V_{30}\}$ Keplerate in 0.1 M Tris-HCl and 0.1M HEPES buffers with pH 7 – 8 at three different conditions: fresh solutions, 24 h at room temperature, and 24 h at 37 °C. Percentages are normalized within each pH row and sum to ~100 % (small deviations reflect rounding or trace unassigned signals).

		Tris-HCl		HEPES	
**POM structure**	**solutions**	**pH 7**	**pH 8**	**pH 7**	**pH 8**
$[V^{V}W^{VI}_{5}O_{19}]^{3-}$	fresh	-	5.5 ± 0.8 %	-	-
	RT	-	6.0 ± 0.3 %	-	-
	inc.	5.5 ± 0.2 %	8.4 ± 0.5 %	-	-
cis-$[HV^{V}_{2}W^{VI}_{4}O_{19}]^{3-}$	fresh	-	9.9 ± 0.6 %	-	-
	RT	-	-	-	-
	inc.	-	-	-	-
$[H_2V^{V}W^{VI}_{11}O_{40}]^{3-}$	fresh	-	-	-	-
	RT	-	5.7 ± 0.8 %	-	-
	inc.	-	6.8 ± 0.4 %	-	-
$[V^{V}_{5}O_{15}]^{5-}$	fresh	-	-	-	1.5 ± 0.3 %
	RT	-	-	-	1.0 ± 0.0 %
	inc.	-	-	-	1.0 ± 0.0 %
$[V^{V}_{4}O_{12}]^{4-}$	fresh	-	-	-	-

	RT	-	1.8 ± 0.7 %	-	-
	inc.	-	1.6 ± 0.3 %	-	0.4 ± 0.3 %
**$[HV^{V}_{3}O_{10}]^{4-}$**	fresh	1.7 ± 0.3 %	4.0 ± 0.4 %	2.8 ± 0.3 %	4.6 ± 0.5 %
	RT	3.1 ± 0.7 %	7.0 ± 0.4 %	5.8 ± 0.1 %	5.4 ± 0.4 %
	inc.	4.5 ± 0.3 %	6.4 ± 0.2 %	5.2 ± 0.3 %	6.4 ± 0.6 %
**$[V^{V}_{2}O_{7}]^{4-}$**	fresh	-	-	-	-
	RT	-	-	-	1.0 ± 0.0 %
	inc.	-	-	-	2.0 ± 1.8 %
**$[H_xV_2O_7]^{x-4}$**	fresh	95.1 ± 0.1 %	-	97.3 ± 0.3 %	-
	RT	93.4 ± 0.7 %	-	94.2 ± 0.1 %	-
	inc.	85.9 ± 0.4 %	-	91.6 ± 0.8 %	-
**$[H_xVO_4]^{x-3}$ x=0-2**	fresh	3.2 ± 0.2 %	80.6 ± 0.2 %	-	94.0 ± 0.3 %
	RT	3.5 ± 0.0 %	71.3 ± 1.5 %	-	92.5 ± 0.5 %
	inc.	4.2 ± 0.1 %	68.4 ± 0.5 %	3.2 ± 0.8 %	90.2 ± 1.1 %

6.3. Resonance Raman spectroscopic studies of $\{W_{72}V_{30}\}$ solutions

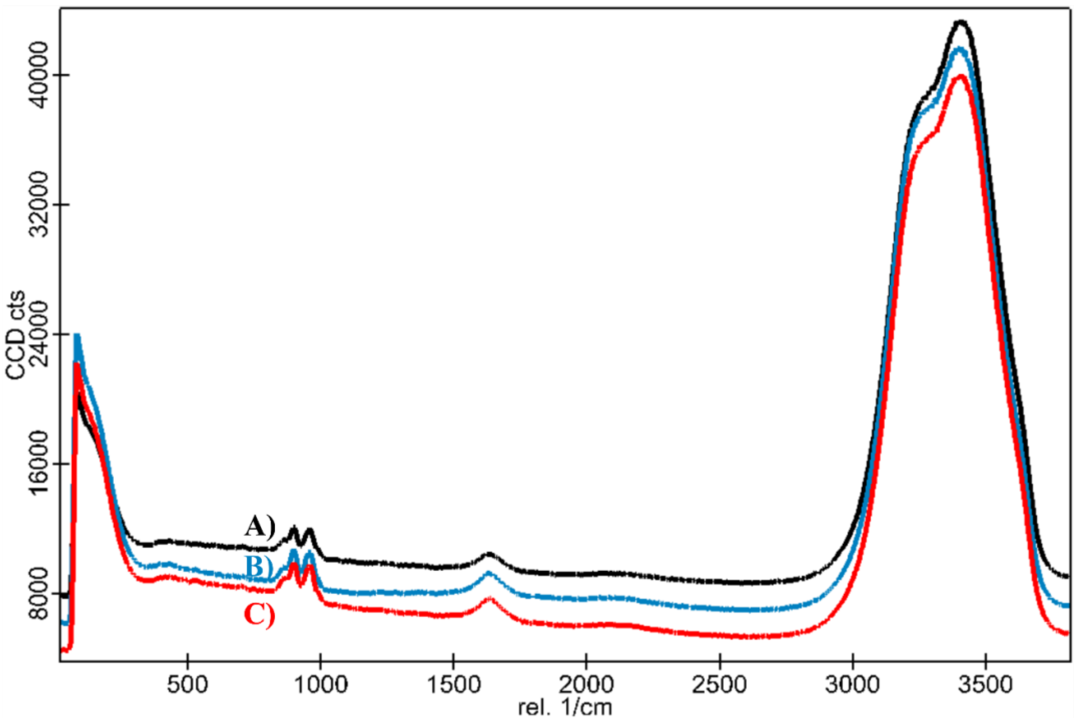

Fig. S54. Resonance Raman spectra of $\{W_{72}V_{30}\}$ in H_2O with pH 2: A) fresh solutions; B) 24 h aging at room temperature and C) 24 h incubation at 37 °C.

Resonance Raman spectra for 0.15 mM fresh solutions (**A**) of **$\{W_{72}V_{30}\}$** in H_2O that were recorded approximately 30 min after preparation. Aliquots of fresh solutions were taken for 24 h aging experiments at room temperature (**B**) and 24 h incubation at 37 °C (**C**) and were then recorded approximately 30 min after the end of 24 h experiments. The RR shifts of formed POM species are given in **Table S27.**

Table S27. Analysis of Resonance Raman spectroscopic data recorded of {$W_{72}V_{30}$} solutions at three different conditions: fresh solutions, 24 h at room temperature, and 24 h at 37 °C.

Resonance Raman (λ_e = 532 nm, E = 25 mW) shifts measured in duplicate of {$W_{72}V_{30}$} (0.15 mM) dissolved in H_2O (pH 1 – 8) or 0.1 M buffers (acetic acid – sodium acetate pH 4 – 5.5; sodium phosphate pH 2 – 8; Tris-HCl pH 7 – 8; HEPES pH 7 – 8). The resonance Raman shifts were assigned based on the literature data summarized in **Table S6**. RR shifts of the intact Keplerates are are shown in bold.

**Sample condition**		**Resonance Raman shifts/ cm^{-1}**	**Species detected**	**Assigment according to literature**
		**H_2O, c($W_{72}V_{30}$) = 0.15 mM**		
**pH 1**	Fresh	719 (w, b), 865 (sh), 899 (m, s), 959 (m, s), 990 (sh)	probably{$W_{72}V_{30}$}	v_{as+s}(O−V−O), v_s(V−O)/ v_s(W−O), v(V=O)/ v(W=O)
	24 h at RT	713 (w, b), 865 (sh), 901 (m, s), 960 (m, s), 990 (sh)	probably{$W_{72}V_{30}$}	v_{as+s}(O−V−O), v_s(V−O)/ v_s(W−O), v(V=O)/ v(W=O)
	24 h at 37 °C	707 (w, b), 865 (sh), 904 (m, s), 961 (m, s), 990 (sh)	{$W_{72}V_{30}$}	v_{as+s}(O−V−O), v_s(V−O)/ v_s(W−O), v(V=O)/ v(W=O)
**pH 1.5**	Fresh	**707 (w, b), 865 (sh), 899 (m, s), 959 (m, s), 990 (sh)**	{$W_{72}V_{30}$}	v_{as+s}(O−V−O), v_s(V−O)/ v_s(W−O), v(V=O)/ v(W=O)
	24 h at RT	716 (w, b), 862 (sh), 900 (m, s), 959 (m, s), 990 (sh)	probably{$W_{72}V_{30}$}	v_{as+s}(O−V−O), v_s(V−O)/ v_s(W−O), v(V=O)/ v(W=O)
	24 h at 37 °C	**703 (w, b), 864 (sh), 900 (m, s), 959 (m, s), 992 (sh)**	{$W_{72}V_{30}$}	v_{as+s}(O−V−O), v_s(V−O)/ v_s(W−O), v(V=O)/ v(W=O)
**pH 2**	Fresh	**705 (w, b), 865 (sh), 900 (m, s), 959 (m, s), 990 (sh)**	{$W_{72}V_{30}$}	v_{as+s}(O−V−O), v_s(V−O)/ v_s(W−O), v(V=O)/ v(W=O)
	24 h at RT	**705 (w, b), 865 (sh), 899 (m, s), 958 (m, s), 990 (sh)**	{$W_{72}V_{30}$}	v_{as+s}(O−V−O), v_s(V−O)/ v_s(W−O), v(V=O)/ v(W=O)
	24 h at 37 °C	**704 (w, b), 865 (sh), 899 (m, s), 959 (m, s), 990 (sh)**	{$W_{72}V_{30}$}	v_{as+s}(O−V−O), v_s(V−O)/ v_s(W−O), v(V=O)/ v(W=O)
**pH 3**	Fresh	**709 (w, b), 865 (sh), 899 (m, s), 957 (m, s), 990 (sh)**	{$W_{72}V_{30}$}	v_{as+s}(O−V−O), v_s(V−O)/ v_s(W−O), v(V=O)/ v(W=O)
	24 h at RT	**705 (w, b), 862 (sh), 899 (m, s), 958 (m, s), 990 (sh)**	{$W_{72}V_{30}$}	v_{as+s}(O−V−O), v_s(V−O)/ v_s(W−O), v(V=O)/ v(W=O)
	24 h at 37 °C	**706 (w, b), 865 (sh), 899 (m, s), 957 (m, s), 991 (sh)**	{$W_{72}V_{30}$}	v_{as+s}(O−V−O), v_s(V−O)/ v_s(W−O), v(V=O)/ v(W=O)
**pH 4**	Fresh	**705 (w, b), 865 (sh), 899 (m, s), 958 (m, s), 987 (sh)**	{$W_{72}V_{30}$}	v_{as+s}(O−V−O), v_s(V−O)/ v_s(W−O), v(V=O)/ v(W=O)
	24 h at RT	**708 (w, b), 862 (sh), 899 (m, s), 955 (m, s), 990 (sh)**	{$W_{72}V_{30}$}	v_{as+s}(O−V−O), v_s(V−O)/ v_s(W−O), v(V=O)/ v(W=O)

	24 h at 37 °C	**709 (w, b), 862 (sh), 898 (m, s), 956 (m, s), 990 (sh)**	$\{W_{72}V_{30}\}$	v_{as+s}(O−V−O), v_s(V−O)/ v_s(W−O), v(V=O)/ v(W=O)
**pH 5**	Fresh	**707 (w, b), 865 (sh), 900 (m, s), 957 (m, s), 990 (sh)**	$\{W_{72}V_{30}\}$	v_{as+s}(O−V−O), v_s(V−O)/ v_s(W−O), v(V=O)/ v(W=O)
	24 h at RT	**704 (w, b), 862 (sh), 899 (m, s), 955 (m, s), 990 (sh)**	$\{W_{72}V_{30}\}$	v_{as+s}(O−V−O), v_s(V−O)/ v_s(W−O), v(V=O)/ v(W=O)
	24 h at 37 °C	**709 (w, b), 862 (sh), 898 (m, s), 956 (m, s), 990 (sh)**	$\{W_{72}V_{30}\}$	v_{as+s}(O−V−O), v_s(V−O)/ v_s(W−O), v(V=O)/ v(W=O)
**pH 6**	Fresh	719 (w, b), **865 (sh)**, **899 (m, s)**, **955 (m, s)**, **990 (sh)**	probably $\{W_{72}V_{30}\}$	v_{as+s}(O−V−O), v_s(V−O)/ v_s(W−O), v(V=O)/ v(W=O)
			$[W_7O_{24}]^{6-}$	v_{as}(W−O), v_s(W−O)
			small vanadates $HV_1 – V_2$	v(V−O)
	24 h at RT	705 (w, b), 777 (w, b), 862 (sh), 899 (m, s), 954 (m, s), 990 (sh)	probably $\{W_{72}V_{30}\}$	v_{as+s}(O−V−O), v_s(V−O)/ v_s(W−O), v(V=O)/ v(W=O)
			$[W_7O_{24}]^{6-}$	v_{as}(W−O), v_s(W−O)
			small vanadates $HV_1 – V_2$	v(V−O)
			777 (w, b) – no matches in the literature	v(O−M−O)
	24 h at 37 °C	**705 (w, b)**, **859 (sh)**, **899 (m, s)**, **957 (m, s)**, **990 (sh)**	probably $\{W_{72}V_{30}\}$	v_{as+s}(O−V−O), v_s(V−O)/ v_s(W−O), v(V=O)/ v(W=O)
**pH 7**	Fresh	**702 (w, b), 862 (sh), 899 (m, s), 959 (m, s), 990 (sh)**	$\{W_{72}V_{30}\}$	v_{as+s}(O−V−O), v_s(V−O)/ v_s(W−O), v(V=O)/ v(W=O)
	24 h at RT	**707 (w, b), 865 (sh), 899 (m, s), 955 (m, s), 990 (sh)**	$\{W_{72}V_{30}\}$	v_{as+s}(O−V−O), v_s(V−O)/ v_s(W−O), v(V=O)/ v(W=O)
	24 h at 37 °C	**705 (w, b), 865 (sh), 899 (m, s), 957 (m, s), 990 (sh)**	$\{W_{72}V_{30}\}$	v_{as+s}(O−V−O), v_s(V−O)/ v_s(W−O), v(V=O)/ v(W=O)
**pH 8**	Fresh	**706 (w, b)**, **862 (sh)**, **899 (m, s)**, 951 (m, s), **987 (sh)**	probably $\{W_{72}V_{30}\}$	v_{as+s}(O−V−O), v_s(V−O)/ v_s(W−O), v(V=O)/ v(W=O)
			$[W_7O_{24}]^{6-}$	v_{as}(W−O), v_s(W−O)
			small vanadates $HV_1 – V_2$	v(V−O)
	24 h at RT	533 (w, b), **706 (w, b)**, **862 (sh)**, **899 (m, s)**, **954 (m, s)**, **990 (sh)**	probably $\{W_{72}V_{30}\}$	v_{as+s}(O−V−O), v_s(V−O)/ v_s(W−O), v(V=O)/ v(W=O)
			$[W_7O_{24}]^{6-}$	v_{as}(W−O), v_s(W−O)
			small vanadates $HV_1 – V_2$	v(V−O)
			$WO_3 \cdot nH_2O$	H_2O libration

	24 h at 37 °C	532 (w, b), 708 (w, b), 865 (sh), 899 (m, s), 956 (m, s), 990 (sh)	probably{$W_{72}V_{30}$}	v_{as+s}(O–V–O), v_s(V–O)/ v_s(W–O), v(V=O)/ v(W=O)
			$[W_7O_{24}]^{6-}$	v_{as}(W–O), v_s(W–O)
			small vanadates HV_1 – V_2	v(V–O)
			WO_3 – nH_2O	H_2O libration
*H_2O shifts / cm^{-1}: 1650 – 3420 (st, b)				
**0.1 M sodium phosphate buffers, c($W_{72}V_{30}$)= 0.15 mM**				
**pH 2**	Fresh	515 (w, b), **707 (w, mb), 862 (sh), 895 (m, mb), 959 (m, s), 993 (sh)**	probably{$W_{72}V_{30}$}	v_{as+s}(O–V–O), v_s(V–O)/ v_s(W–O), v(V=O)/ v(W=O)
			WO_3 – nH_2O	H_2O libration
	24 h at RT	497 (w, b), 713 (w, mb), 865 (sh), 895 (m, s), 960 (m, s), 990 (sh)	$[HW_6O_{21}]^{5-}$ or $[W_7O_{24}]^{6-}$	v_{as}(W–O), v_s(W–O)
			small vanadates HV_1 – V_5	v(V–O)
			$WO_3 \cdot nH_2O$	H_2O libration
	24 h at 37 °C	530 (w, b), 719 (w, b), 871 (sh), 896 (m, s), 960 (m, s), 990 (sh)	$[HW_6O_{21}]^{5-}$ or $[W_7O_{24}]^{6-}$	v_{as}(W–O), v_s(W–O)
			small vanadates HV_1 – V_5	v(V–O)
			$WO_3 \cdot nH_2O$	H_2O libration
**pH 3**	Fresh	524 (w, b), 708 (w, b), 878 (sh), 898 (m, s), 959 (m, s), 990 (sh)	$[HW_6O_{21}]^{5-}$ or $[W_7O_{24}]^{6-}$	v_{as}(W–O), v_s(W–O)
			small vanadates HV_1 – V_5	v(V–O)
			$WO_3 \cdot nH_2O$	H_2O libration
	24 h at RT	508 (w, b), 706 (w, b), 878 (sh), 899 (m, s), 960 (m, s), 990 (sh)	$[HW_6O_{21}]^{5-}$ or $[W_7O_{24}]^{6-}$	v_{as}(W–O), v_s(W–O)
			small vanadates HV_1 – V_5	v(V–O)
			$WO_3 \cdot nH_2O$	H_2O libration
	24 h at 37 °C	502 (w, b), 707 (w, b), 872 (sh), 899 (m, s), 960 (m, s)	$[HW_6O_{21}]^{5-}$ or $[W_7O_{24}]^{6-}$	v_{as}(W–O), v_s(W–O)
			small vanadates HV_1 – V_5	v(V–O)
			$WO_3 \cdot nH_2O$	H_2O libration
**pH 4**	Fresh	521 (w, b), 704 (w, b), 881 (sh), 899 (m, s), 957 (m, s), 990 (sh)	$[HW_6O_{21}]^{5-}$	v_{as}(W–O), v_s(W–O)
			small vanadates HV_1 – V_5	v(V–O)
			$WO_3 \cdot nH_2O$	H_2O libration
	24 h at RT	516 (w, b), 706 (w, b), 875 (sh), 898 (m, s), 958 (m, s)	$[HW_6O_{21}]^{5-}$	v_{as}(W–O), v_s(W–O)
			small vanadates HV_1 – V_5	v(V–O)

			$WO_3 \cdot nH_2O$	H_2O libration
	24 h at 37 °C	499 (w, b), 705 (w, b), 871 (sh), 899 (m, s), 959 (m, s), 990 (sh)	$[HW_6O_{21}]^{5-}$	v_{as}(W−O), v_s(W−O)
			small vanadates $HV_1 - V_5$	v(V−O)
			$WO_3 \cdot nH_2O$	H_2O libration
**pH 5**	Fresh	514 (w, b), 708 (w, b), 875 (sh), 899 (m, s), 957 (m, s), 990 (sh)	$[HW_6O_{21}]^{5-}$	v_{as}(W−O), v_s(W−O)
			small vanadates $HV_1 - V_5$	v(V−O)
			$WO_3 \cdot nH_2O$	H_2O libration
	24 h at RT	532 (w, b), 711 (w, b), 871 (sh), 899 (m, s), 959 (m, s), 990 (sh)	$[HW_6O_{21}]^{5-}$	v_{as}(W−O), v_s(W−O)
			small vanadates $HV_1 - V_5$	v(V−O)
			$WO_3 \cdot nH_2O$	H_2O libration
	24 h at 37 °C	605 (w, b), 711 (w, b), 875 (sh), 899 (m, s), 959 (m, s)	$[HW_6O_{21}]^{5-}$	v_{as}(W−O), v_s(W−O)
			small vanadates $HV_1 - V_5$	v(V−O)
			$WO_3 \cdot nH_2O$	H_2O libration
**pH 6**	Fresh	517 (w, b), 710 (w, b), 875 (sh), 900 (m, s), 956 (m, s), 990 (sh)	$[HW_6O_{21}]^{5-}$	v_{as}(W−O), v_s(W−O)
			small vanadates $HV_1 - V_5$	v(V−O)
			$WO_3 \cdot nH_2O$	H_2O libration
	24 h at RT	507 (w, b), 711 (w, b), 881 (sh), 898 (m, s), 956 (m, s)	$[HW_6O_{21}]^{5-}$	v_{as}(W−O), v_s(W−O)
			small vanadates $HV_1 - V_5$	v(V−O)
			$WO_3 \cdot nH_2O$	H_2O libration
	24 h at 37 °C	520 (w, b), 710 (w, b), 875 (sh), 899 (m, s), 956 (m, s), 990 (sh)	$[HW_6O_{21}]^{5-}$	v_{as}(W−O), v_s(W−O)
			small vanadates $HV_1 - V_5$	v(V−O)
			$WO_3 \cdot nH_2O$	H_2O libration
**pH 7**	Fresh	520 (w, b), **705 (w, b), 868 (sh), 899 (m, s), 959 (m, s), 987 (sh)**	probably $\{W_{72}V_{30}\}$	v_{as+s}(O−V−O), v_s(V−O)/ v_s(W−O), v(V=O)/ v(W=O)
			$WO_3 \cdot nH_2O$	H_2O libration
	24 h at RT	521 (w, b), **705 (w, b), 865 (sh), 899 (m, s), 958 (m, s), 987 (sh)**	probably $\{W_{72}V_{30}\}$	v_{as+s}(O−V−O), v_s(V−O)/ v_s(W−O), v(V=O)/ v(W=O)
			$WO_3 \cdot nH_2O$	H_2O libration
	24 h at 37 °C	531 (w, b), 704 (w, b), 865 (sh), 899 (m, s), 963 (m, s), 974 (sh)	$[HW_6O_{21}]^{5-}$	v_{as}(W−O), v_s(W−O)
			small vanadates $HV_1 - V_5$	v(V−O)
			$WO_3 \cdot nH_2O$	H_2O libration

**pH 8**	Fresh	531 (w, b), 704 (w, b), 868 (sh), 900 (m, s), 959 (m, mb), 984 (m, mb)	$WO_3 \cdot nH_2O$	H_2O libration
			$[W_7O_{24}]^{6-}$	v_{as}(W−O), v_s(W−O)
			small vanadates $HV_1 - V_5$	v(V−O)
	24 h at RT	526 (w, b), 702 (w, b), 792 (sh), 859 (sh), 895 (m, mb), 953 (m, mb), 963 (sh), 987 (m, s)	$WO_3 \cdot nH_2O$	H_2O libration
			$[W_7O_{24}]^{6-}$	v_{as}(W−O), v_s(W−O)
			$[W_6O_{21}]^{5-}$	v_{as}(W−O), v_s(W−O)
			small vanadates $HV_1 - V_5$	v(V−O)
	24 h at 37 °C	529 (w, b), 707 (w, b), 801 (sh), 892 (m, mb), 933 (sh), 947 (m, mb), 983 (m, s)	$WO_3 \cdot nH_2O$	H_2O libration
			$[W_{12}O_{40}]^{8-}$	v_{as}(W−O), v_s(W−O)
			small vanadates $HV_1 - V_5$	v(V−O)
*sodium phosphate buffers shift / cm^{-1}: 1075 (w, mb), 1651 (m, b), 2084 (w, b), 2616 (w, b), 3426 (st, b)				
**0.1 M acetic acid – sodium acetate buffers, $c(W_{72}V_{30})$ = 0.20 mM**				
**pH 4**	Fresh	**708 (w, b)**, **865 (sh)**, **898 (m, s)**, **958 (m, s)**, **990 (sh)**	probably $\{W_{72}V_{30}\}$	v_{as+s}(O−V−O), v_s(V−O)/ v_s(W−O), v(V=O)/ v(W=O)
	24 h at RT	**706 (w, b)**, **864 (sh)**, **897 (m, s)**, **958 (m, s)**, **992 (sh)**	probably $\{W_{72}V_{30}\}$	v_{as+s}(O−V−O), v_s(V−O)/ v_s(W−O), v(V=O)/ v(W=O)
	24 h at 37 °C	712 (w, b), 868 (sh), 897 (m, s), 959 (m, s), 992 (sh)	$[HW_6O_{21}]^{5-}$ or $[W_7O_{24}]^{6-}$	v_{as}(W−O), v_s(W−O)
			small vanadates $HV_1 - V_5$	v(V−O)
**pH 5**	Fresh	527 (w, b), 708 (w, b), 864 (sh), 899 (m, s), 930 (w, b), 956 (m, s), 992 (sh)	$WO_3 \cdot nH_2O$	H_2O libration
			$[W_{12}O_{40}]^{8-}$	v_{as}(W−O), v_s(W−O)
			small vanadates $HV_1 - V_5$	v(V−O)
	24 h at RT	529 (w, b), 707 (w, b), 864 (sh), 898 (m, s), 929 (w, b), 957 (m, s), 992 (sh)	$WO_3 \cdot nH_2O$	H_2O libration
			$[W_{12}O_{40}]^{8-}$	v_{as}(W−O), v_s(W−O)
			small vanadates $HV_1 - V_5$	v(V−O)
	24 h at 37 °C	542 (w, b), 709 (w, b), 865 (sh), 899 (m, s), 929 (w, b), 959 (m, s), 993 (sh)	$WO_3 \cdot nH_2O$	H_2O libration
			$[W_{12}O_{40}]^{8-}$	v_{as}(W−O), v_s(W−O)
			small vanadates $HV_1 - V_5$	v(V−O)
**pH 5.5**	Fresh	542 (w, b), 703 (w, b), 862 (sh), 899 (m, s), 929 (w, b), 954 (m, s), 990 (sh)	$WO_3 \cdot nH_2O$	H_2O libration
			$[W_{12}O_{40}]^{8-}$	v_{as}(W−O), v_s(W−O)
			small vanadates $HV_1 - V_5$	v(V−O)
			$WO_3 \cdot nH_2O$	H_2O libration

	24 h at RT	536 (w, b), 708 (w, b), 859 (sh), 899 (m, s), 929 (w, b), 957 (m, s), 990 (sh)	$[W_{12}O_{40}]^{8-}$	$v_{as}(W-O)$, $v_s(W-O)$
			small vanadates $HV_1 - V_5$	$v(V-O)$
	24 h at 37 °C	546 (w, b), 707 (w, b), 865 (sh), 900 (m, s), 932 (w, b), 957 (m, s), 993 (sh)	$WO_3{\cdot}nH_2O$	H_2O libration
			$[W_{12}O_{40}]^{8-}$	$v_{as}(W-O)$, $v_s(W-O)$
			small vanadates $HV_1 - V_5$	$v(V-O)$
*acetic acid – sodium acetate buffers shifts / cm^{-1}: 1352 (w, mb), 1411 (w, mb), 1696 (m, b), 2936 (w, s), 3441 (st, b)				
**0.1 M Tris – HCl buffers, $c(W_{72}V_{30})$ = 0.15 mM**				
**pH 7**	Fresh	525 (w, b), 707 (w, b), 861 (sh), 896 (m, s), 956 (m, s), 992 (sh)	$WO_3{\cdot}nH_2O$	H_2O libration
			$[HW_6O_{21}]^{5-}$ or $[W_7O_{24}]^{6-}$	$v_{as}(W-O)$, $v_s(W-O)$
			small vanadates $HV_1 - V_5$	$v(V-O)$
	24 h at RT	710 (w, b), 861 (sh), 897 (m, s), 967 (m, s), 995 (sh)	$WO_3{\cdot}nH_2O$	H_2O libration
			$[HW_6O_{21}]^{5-}$ or $[W_7O_{24}]^{6-}$	$v_{as}(W-O)$, $v_s(W-O)$
			$[W_{10}O_{32}]^{4-}$	$v(W-O)$
			small vanadates $HV_1 - V_5$	$v(V-O)$
	24 h at 37 °C	520 (w, b), 699 (w, b), 714 (w, b), 864 (m, s), 957 (m, s)	$WO_3{\cdot}nH_2O$	H_2O libration
			699 (w, b) – no matches in the literature	$v(O-V-O)$
			$[HW_6O_{21}]^{5-}$ or $[W_7O_{24}]^{6-}$	$v_{as}(W-O)$, $v_s(W-O)$
			small vanadates $HV_1 - V_5$	$v(V-O)$
**pH 8**	Fresh	527 (w, b), 598 (w, b), 707 (w, b), 864 (sh), 895 (m, s), 956 (m, s), 995 (sh), 1225 (w, b), 1360 (w, b)	$WO_3{\cdot}nH_2O$	H_2O libration
			598 (w, b) – no matches in the literature	$v(O-V-O)$
			$[W_{10}O_{32}]^{4-}$	$v(W-O)$
			small vanadates $HV_1 - V_5$	$v(V-O)$
	24 h at RT	529 (w, b), 607 (w, b), 857 (w, s), 893 (w, s), 933 (w, s), 957 (w, s)	$WO_3{\cdot}nH_2O$	H_2O libration
			$[WO_4]^{2-}$ or $[W_2O_7]^{2-}$	$v(W-O)$
			$[W_{12}O_{40}]^{8-}$	$v_{as}(W-O)$, $v_s(W-O)$
			small vanadates $HV_1 - V_5$	$v(V-O)$
	24 h at 37 °C	868 (w, s), 893 (w, s), 933 (w, s), 957 (w, s), 992 (sh)	$[W_{12}O_{40}]^{8-}$	$v_{as}(W-O)$, $v_s(W-O)$
			small vanadates $HV_1 - V_5$	$v(V-O)$

*Tris – HCl buffers shifts / cm^{-1}: 1064 (w, mb), 1468 (w, s), 1732 (m, b), 2604 (w, b), 2898 (m, s), 2961 (m, s), 3435 (st, b)				
**0.1 M HEPES buffers, c($W_{72}V_{30}$) = 0.15 mM**				
**pH 7**	Fresh	864 (sh), 899 (m, s), 961 (m, s), 988 (sh)	$[W_7O_{24}]^{6-}$	v_{as}(W−O), v_s(W−O)
			small vanadates HV_1 – V_5	v(V−O)
	24 h at RT	701 (w, b), 795 (w, s), 864 (sh), 899 (m, s), 959 (m, s)	701 (w, b) – no matches in the literature	v(V−O)
			$[W_7O_{24}]^{6-}$	v_{as}(W−O), v_s(W−O)
			small vanadates HV_1 – V_6	v(V−O)
	24 h at 37 °C	868 (sh), 899 (m, s), 960 (m, s)	$[W_7O_{24}]^{6-}$	v_{as}(W−O), v_s(W−O)
			small vanadates HV_1 – V_5	v(V−O)
**pH 8**	Fresh	502 (w, mb), 602 (w, b), 859 (sh), 899 (m, s), 958 (m, s)	$WO_3{\cdot}nH_2O$	H_2O libration
			602 (w, b) – no matches in the literature	v(O−V−O)
			$[W_7O_{24}]^{6-}$	v_{as}(W−O), v_s(W−O)
			small vanadates HV_1 – V_6	v(V−O)
	24 h at RT	850 (sh), 897 (w, s), 955 (w, s)	$[W_7O_{24}]^{6-}$	v_{as}(W−O), v_s(W−O)
			small vanadates HV_1 – V_6	v(V−O)
	24 h at 37 °C	856 (sh), 898 (w, s), 935 (sh), 956 (w, mb)	$[W_{12}O_{40}]^{8-}$	v_{as}(W−O), v_s(W−O)
			small vanadates HV_1 – V_6	v(V−O)
*HEPES buffers shifts / cm^{-1}: 765 (w, mb), 840 (sh), 1035 (m, s), 1195 (w, b), 1305 (w, mb), 1429 (sh), 1451 (w, mb), 1464 (w, mb), 1749 (m, b), 2841 (m, mb), 2944 (sh), 2970 (st, s), 3480 (st, b)				

*Intensity: st – strong, m – medium, w – weak. Shape: b – broad, mb – medium broad, s – sharp, sh – shoulder.

6.4. {$W_{72}V_{30}$} rearrangement schemes

6.4.1. {$W_{72}V_{30}$} rearrangement schemes in H_2O (pH 1 – 8)

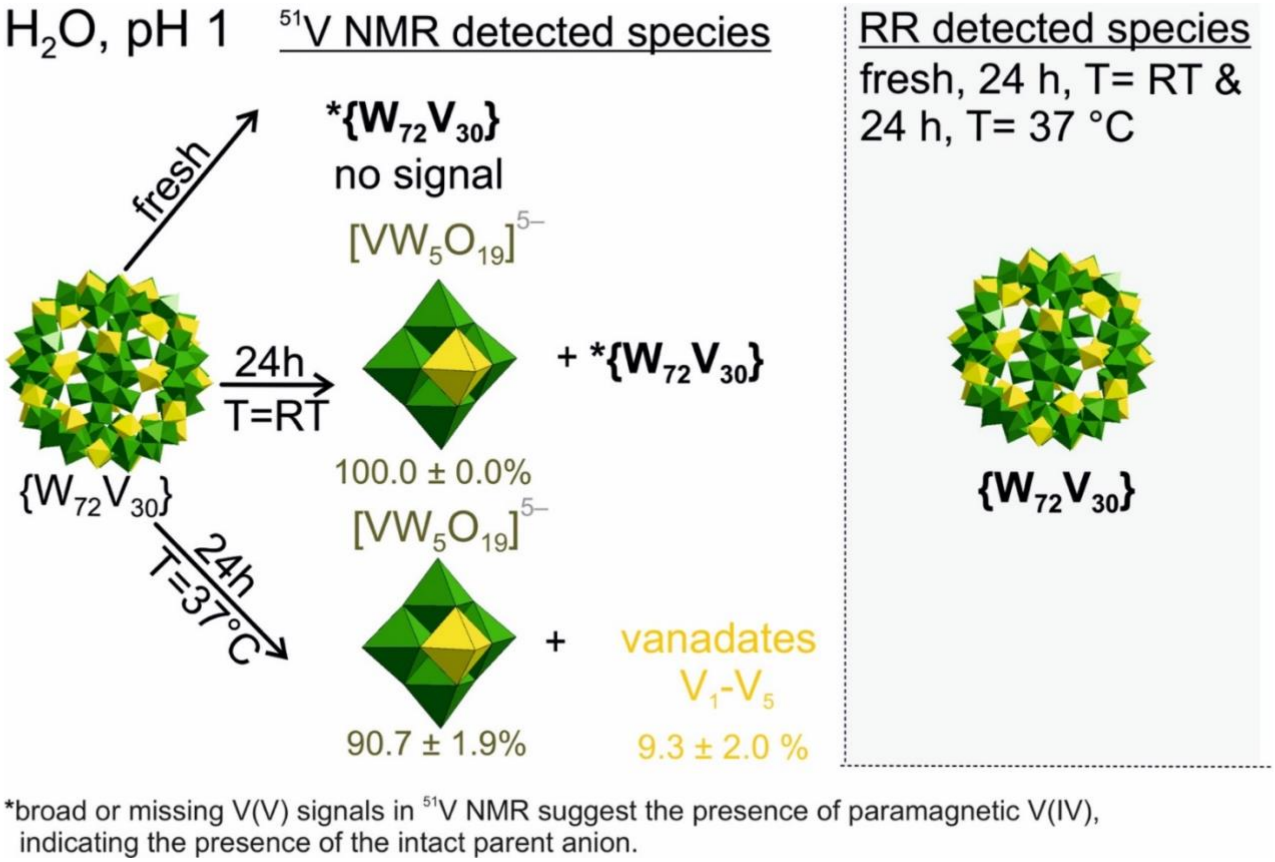

Fig. S55. Rearrangement scheme of {$W_{72}V_{30}$} in H_2O (pH 1).

The scheme is based on the collected and analyzed ^{51}V-NMR and resonance Raman data (gray field). The structures of all POMs are shown in **Figures S2** and **S3**. **Tables S20 – S22** give all ^{51}V NMR chemical shifts and percentages of species (**Table S23**) formed based on ^{51}V NMR and **Table S27** gives resonance Raman shifts, and their assignation based on literature data (**Tables S5 – S6**). Only species with a percentage > 10 % are shown separately; other species are separated by the type of addenda metals in the structure. Color code: {WO_6}, green; {VO_6}, yellow.

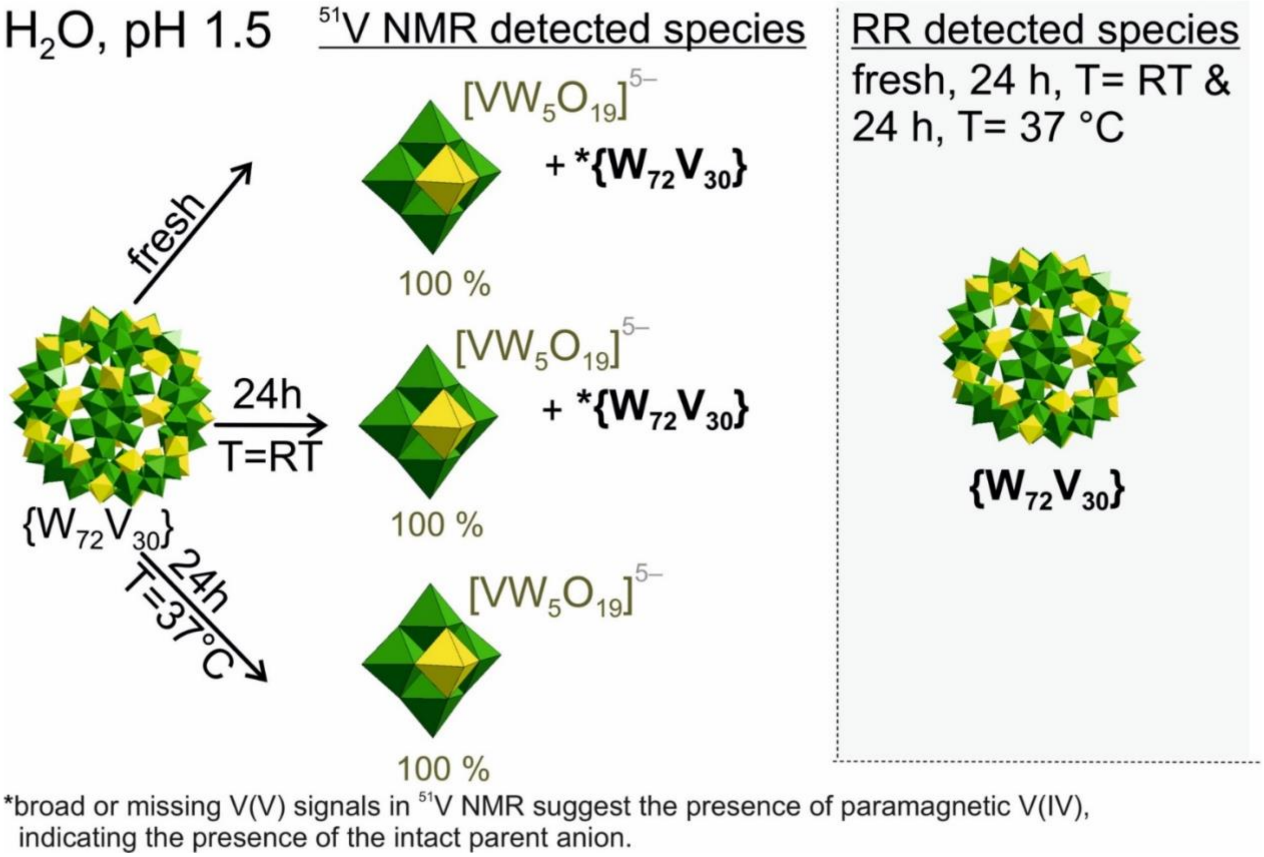

Fig. S56. Rearrangement scheme of $\{W_{72}V_{30}\}$ H_2O (pH 1.5).

The scheme is based on the collected and analyzed ^{51}V-NMR and resonance Raman data (gray field). The structures of all POMs are shown in **Figures S2** and **S3**. **Tables S20 – S22** give all ^{51}V NMR chemical shifts and percentages of species (**Table S23**) formed based on ^{51}V NMR and **Table S27** gives resonance Raman shifts, and their assignation based on literature data (**Tables S5 – S6**). Only species with a percentage > 10 % are shown separately; other species are separated by the type of addenda metals in the structure. Color code: $\{WO_6\}$, green; $\{VO_6\}$, yellow.

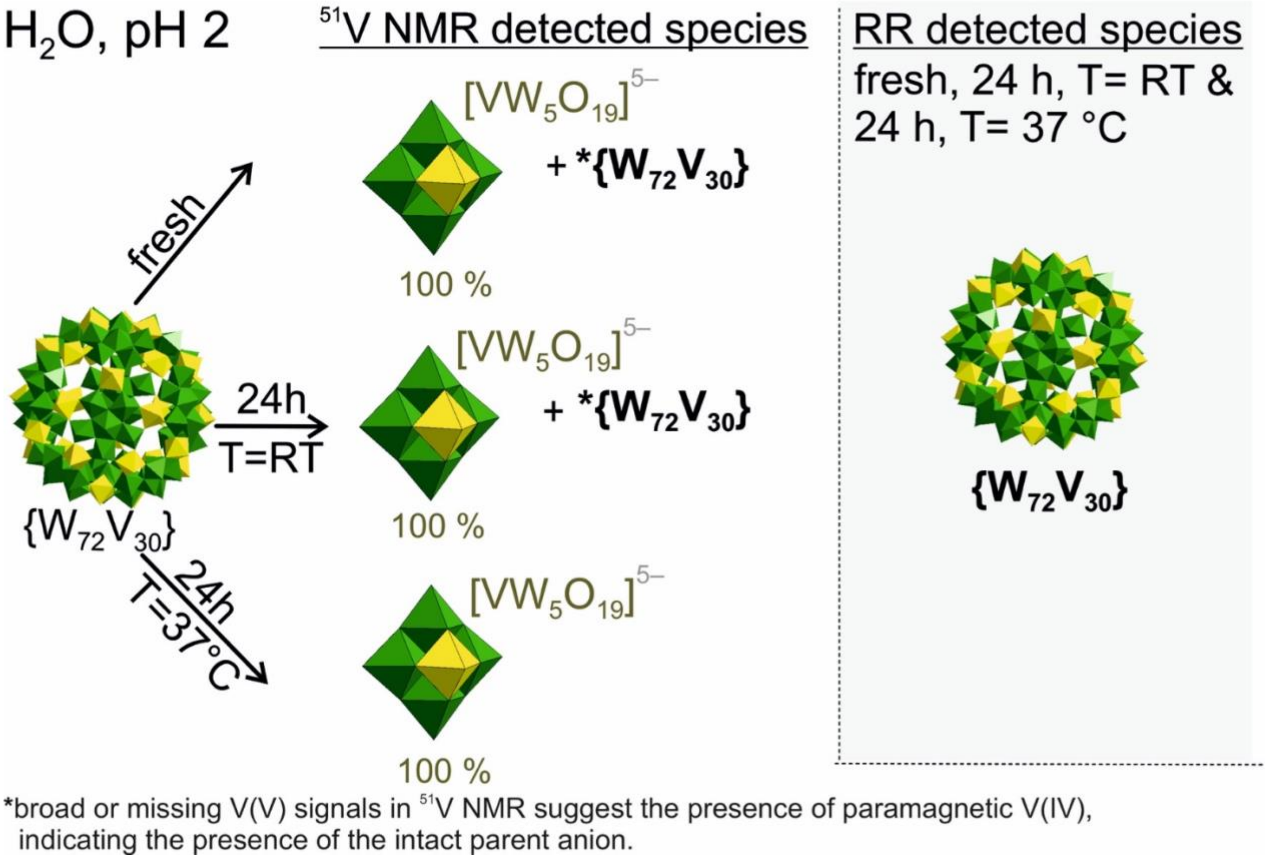

Fig. S57. Rearrangement scheme of $\{W_{72}V_{30}\}$ in H_2O (pH 2).

The scheme is based on the collected and analyzed ^{51}V-NMR and resonance Raman data (gray field). The structures of all POMs are shown in **Figures S2** and **S3**. **Tables S20 – S22** give all ^{51}V NMR chemical shifts and percentages of species (**Table S23**) formed based on ^{51}V NMR and **Table S27** gives resonance Raman shifts, and their assignation based on literature data (**Tables S5 – S6**). Only species with a percentage > 10 % are shown separately; other species are separated by the type of addenda metals in the structure. Color code: $\{WO_6\}$, green; $\{VO_6\}$, yellow.

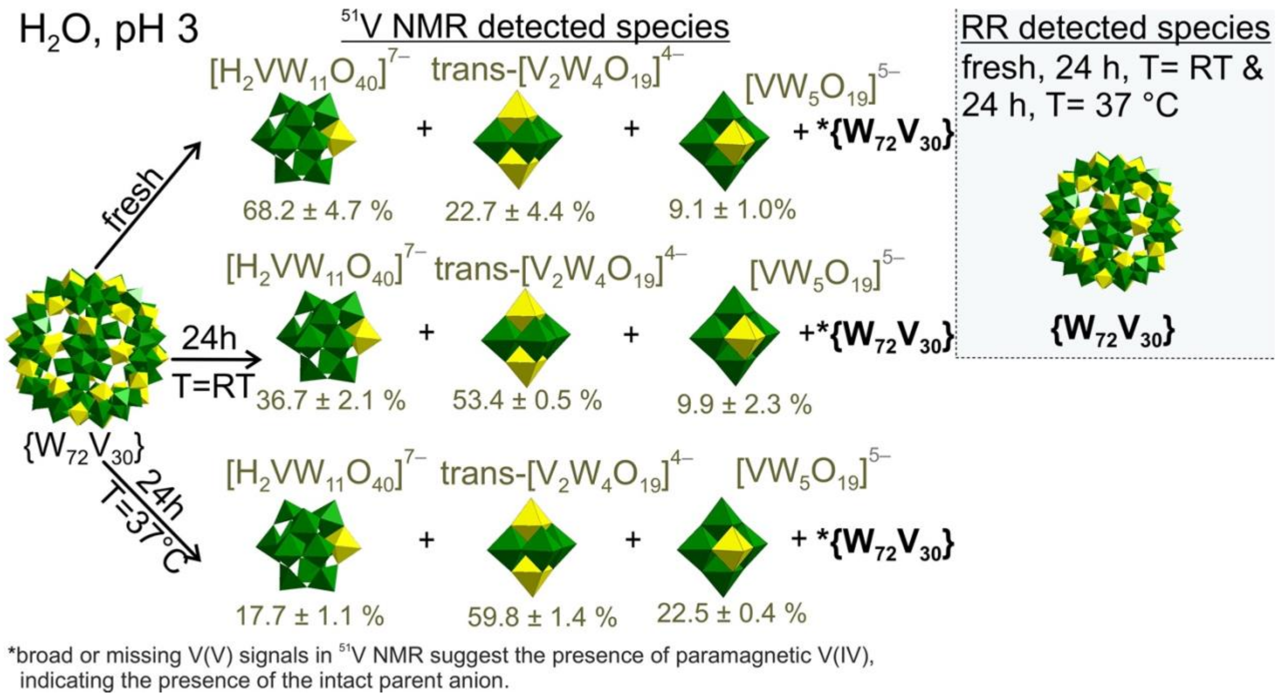

Fig. S58. Rearrangement scheme of {$W_{72}V_{30}$} in H_2O (pH 3).

The scheme is based on the collected and analyzed ^{51}V-NMR and resonance Raman data (gray field). The structures of all POMs are shown in **Figures S2** and **S3**. **Tables S20 – S22** give all ^{51}V NMR chemical shifts and percentages of species (**Table S23**) formed based on ^{51}V NMR and **Table S27** gives resonance Raman shifts, and their assignation based on literature data (**Tables S5 – S6**). Only species with a percentage > 10 % are shown separately; other species are separated by the type of addenda metals in the structure. Color code: {WO_6}, green; {VO_6}, yellow.

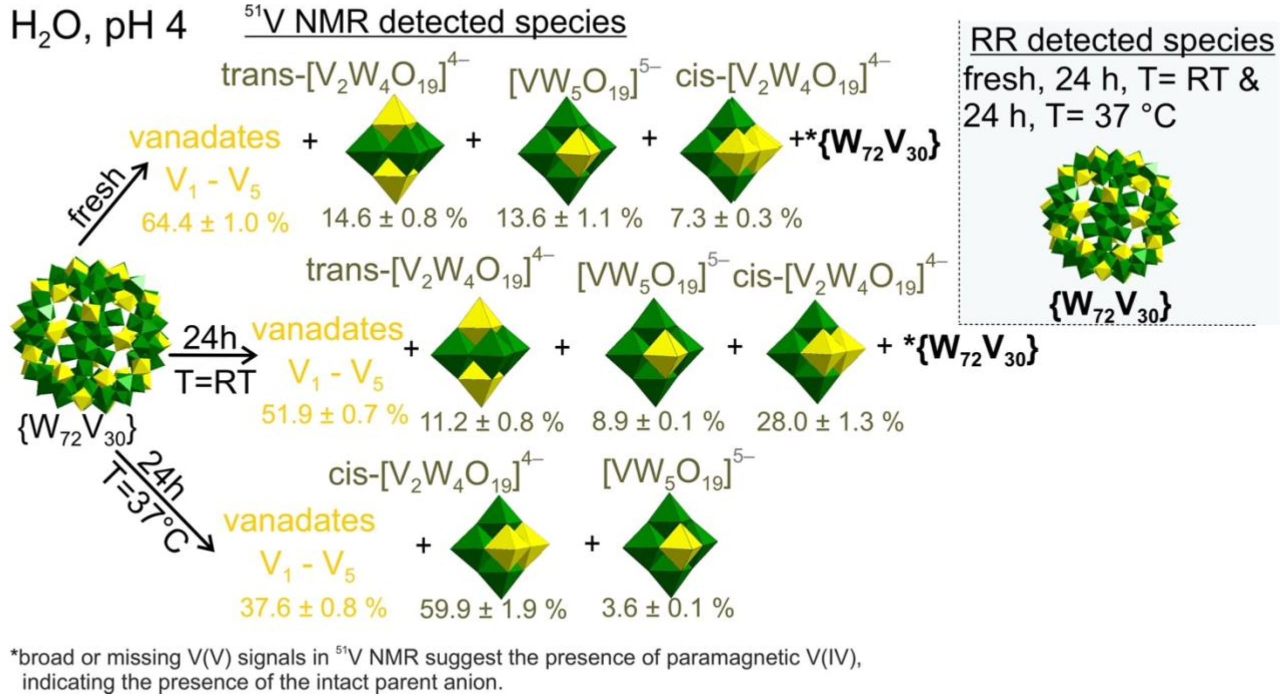

Fig. S59. Rearrangement scheme of {$W_{72}V_{30}$} in H_2O (pH 4).

The scheme is based on the collected and analyzed ^{51}V-NMR and resonance Raman data (gray field). The structures of all POMs are shown in **Figures S2** and **S3**. **Tables S20 – S22** give all ^{51}V NMR chemical shifts and percentages of species (**Table S23**) formed based on ^{51}V NMR and **Table S27** gives resonance Raman shifts, and their assignation based on literature data (**Tables S5 – S6**). Only species with a percentage > 10 % are shown separately; other species are separated by the type of addenda metals in the structure. Color code: {WO_6}, green; {VO_6}, yellow.

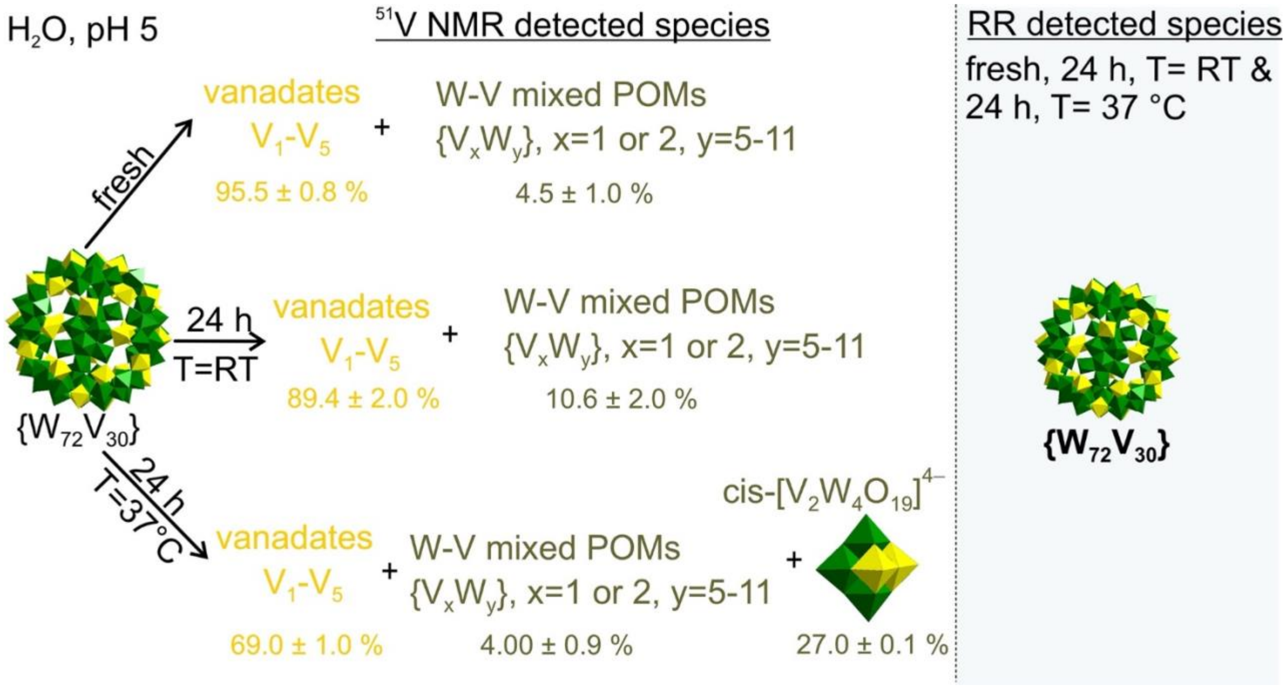

Fig. S60. Rearrangement scheme of $\{W_{72}V_{30}\}$ in H_2O (pH 5).

The scheme is based on the collected and analyzed ^{51}V-NMR and resonance Raman data (gray field). The structures of all POMs are shown in **Figures S2** and **S3**. **Tables S20 – S23** give all ^{51}V NMR chemical shifts and percentages of species (**Table S23**) formed based on ^{51}V NMR and **Table S27** gives resonance Raman shifts, and their assignation based on literature data (**Tables S5 – S6**). Only species with a percentage > 10 % are shown separately; other species are separated by the type of addenda metals in the structure. Color code: $\{WO_6\}$, green; $\{VO_6\}$, yellow.

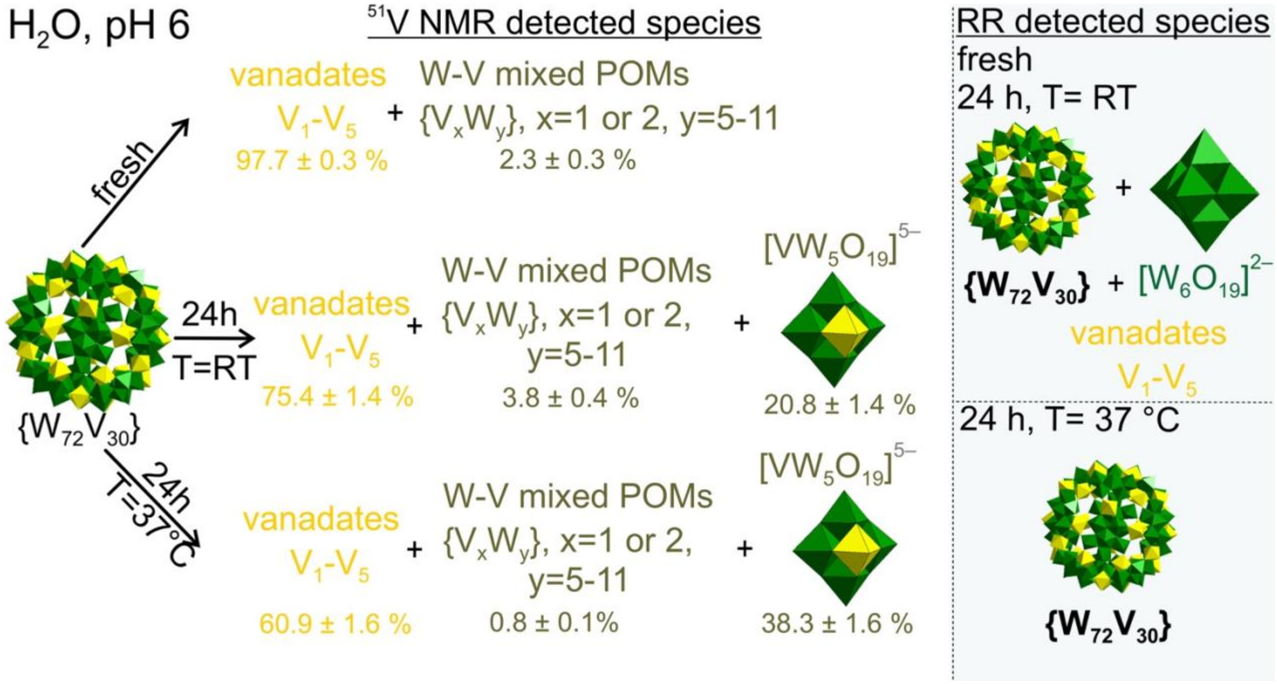

Fig. S61. Rearrangement scheme of $\{W_{72}V_{30}\}$ in H_2O (pH 6).

The scheme is based on the collected and analyzed ^{51}V-NMR and resonance Raman data (gray field). The structures of all POMs are shown in **Figures S2** and **S3**. **Tables S20 – S23** give all ^{51}V NMR chemical shifts and percentages of species (**Table S23**) formed based on ^{51}V NMR and **Table S27** gives resonance Raman shifts, and their assignation based on literature data (**Tables S5 – S6**). Only species with a percentage > 10 % are shown separately; other species are separated by the type of addenda metals in the structure. Color code: $\{WO_6\}$, green; $\{VO_6\}$, yellow.

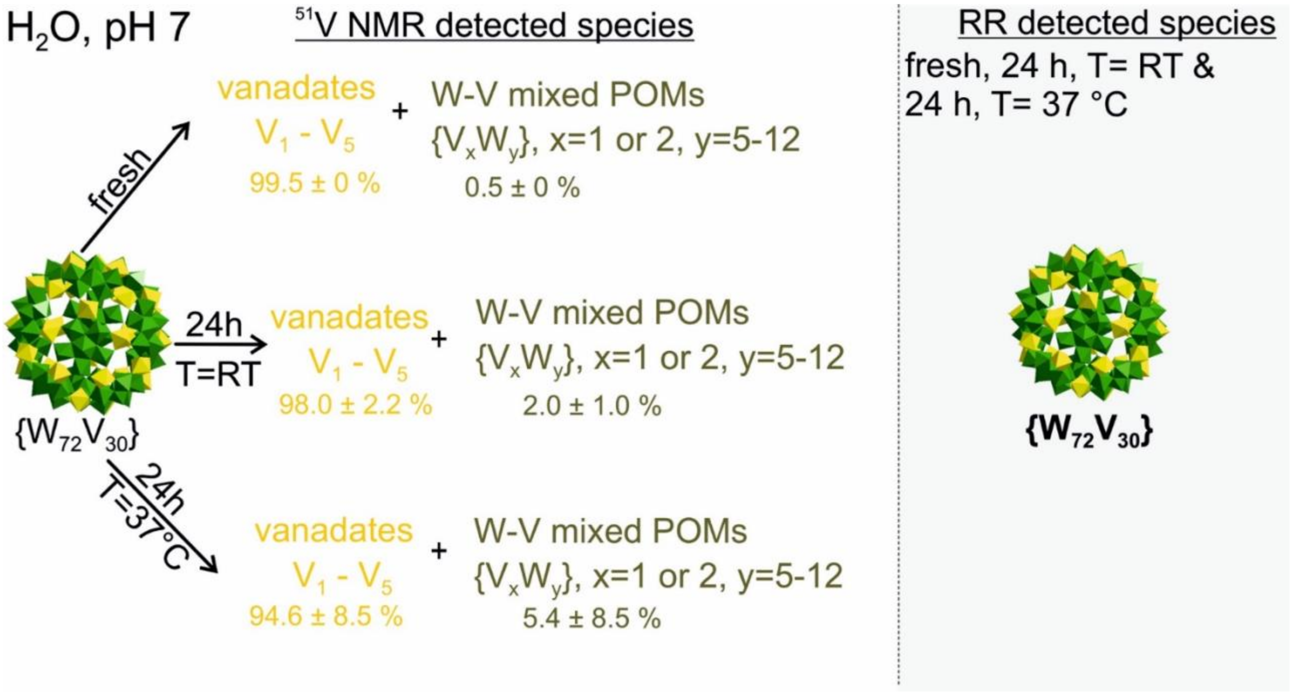

Fig. S62. Rearrangement scheme of $\{W_{72}V_{30}\}$ in H_2O (pH 7).

The scheme is based on the collected and analyzed ^{51}V-NMR and resonance Raman data (gray field). The structures of all POMs are shown in **Figures S2** and **S3**. **Tables S20 – S22** give all ^{51}V NMR chemical shifts and percentages of species (**Table S23**) formed based on ^{51}V NMR and **Table S27** gives resonance Raman shifts, and their assignation based on literature data (**Tables S5 – S6**). Only species with a percentage > 10 % are shown separately; other species are separated by the type of addenda metals in the structure. Color code: $\{WO_6\}$, green; $\{VO_6\}$, yellow.

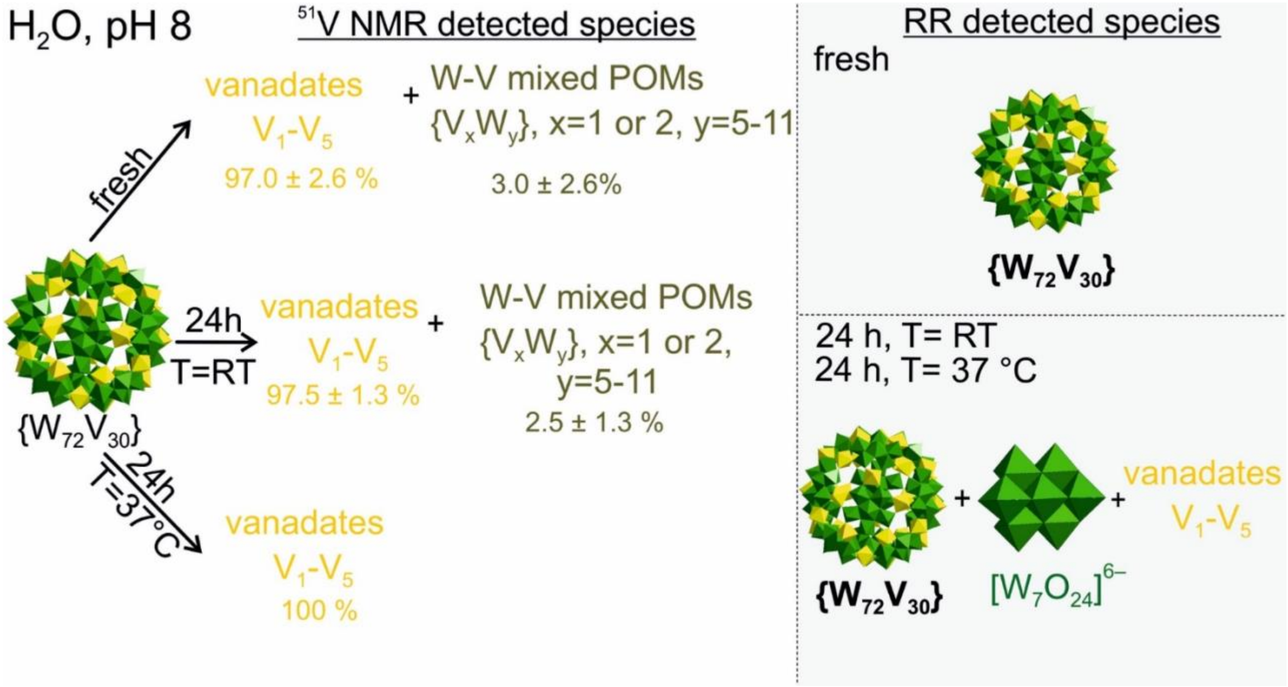

Fig. S63. Rearrangement scheme of $\{W_{72}V_{30}\}$ in H_2O (pH 8).

The scheme is based on the collected and analyzed ^{51}V-NMR and resonance Raman data (gray field). The structures of all POMs are shown in **Figures S2** and **S3**. **Tables S20 – S22** give all ^{51}V NMR chemical shifts and percentages of species (**Table S23**) formed based on ^{51}V NMR and **Table S27** gives resonance Raman shifts, and their assignation based on literature data (**Tables S5 – S6**). Only species with a percentage > 10 % are shown separately; other species are separated by the type of addenda metals in the structure. Color code: $\{WO_6\}$, green; $\{VO_6\}$, yellow.

6.4.2. {$W_{72}V_{30}$} rearrangement schemes in 0.1 M Sodium phosphate buffers.

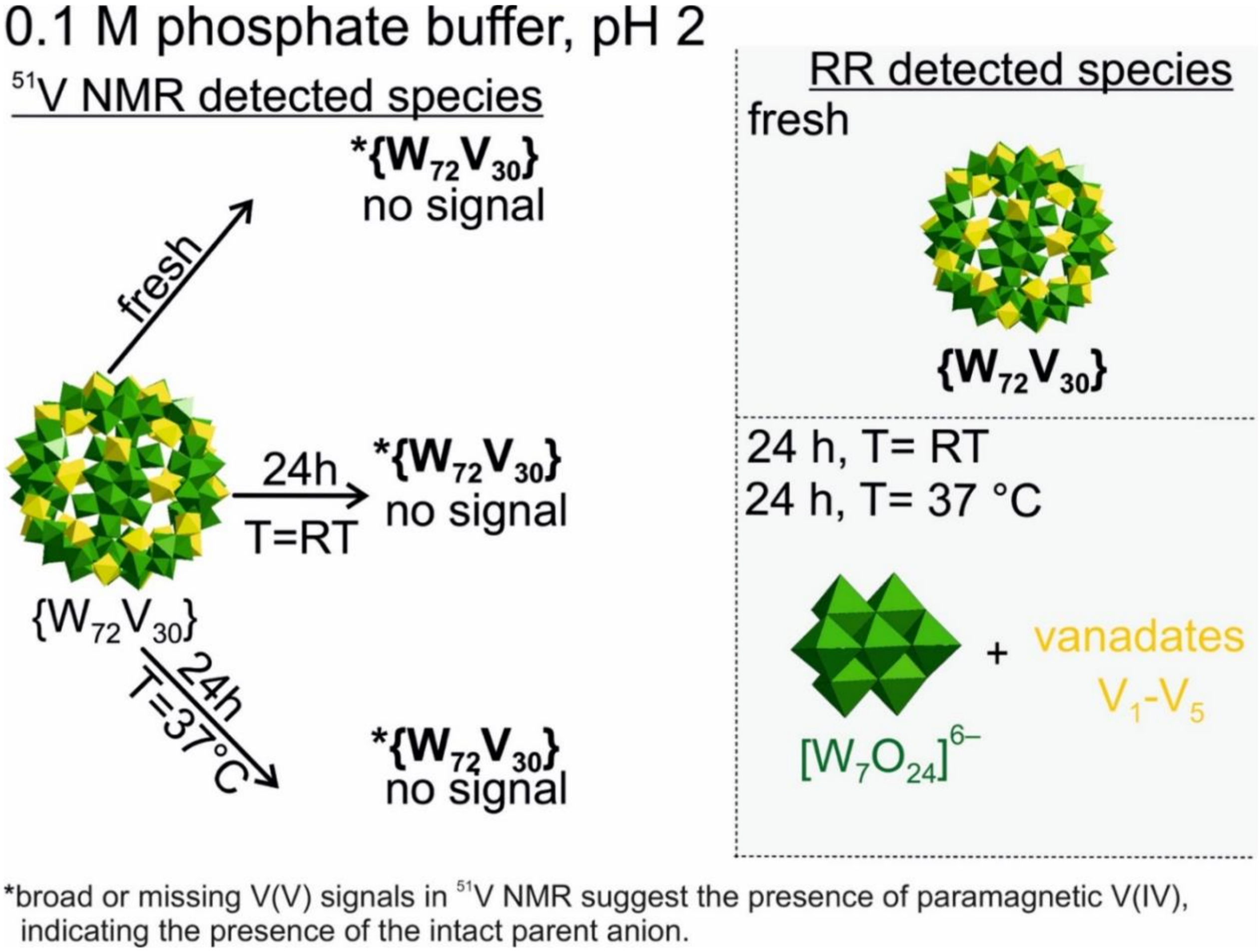

Fig. S64. Rearrangement scheme of {$W_{72}V_{30}$} in 0.1 M sodium phosphate buffer (pH 2).

The scheme is based on the collected and analyzed ^{51}V-NMR and resonance Raman data (gray field). The structures of all POMs are shown in **Figures S2** and **S3**. **Tables S20 – S22** give all ^{51}V NMR chemical shifts and percentages of species (**Table S24**) formed based on ^{51}V NMR and **Table S27** gives resonance Raman shifts, and their assignation based on literature data (**Tables S5 – S6**). Only species with a percentage > 10 % are shown separately; other species are separated by the type of addenda metals in the structure. Color code: {WO_6}, green; {VO_6}, yellow.

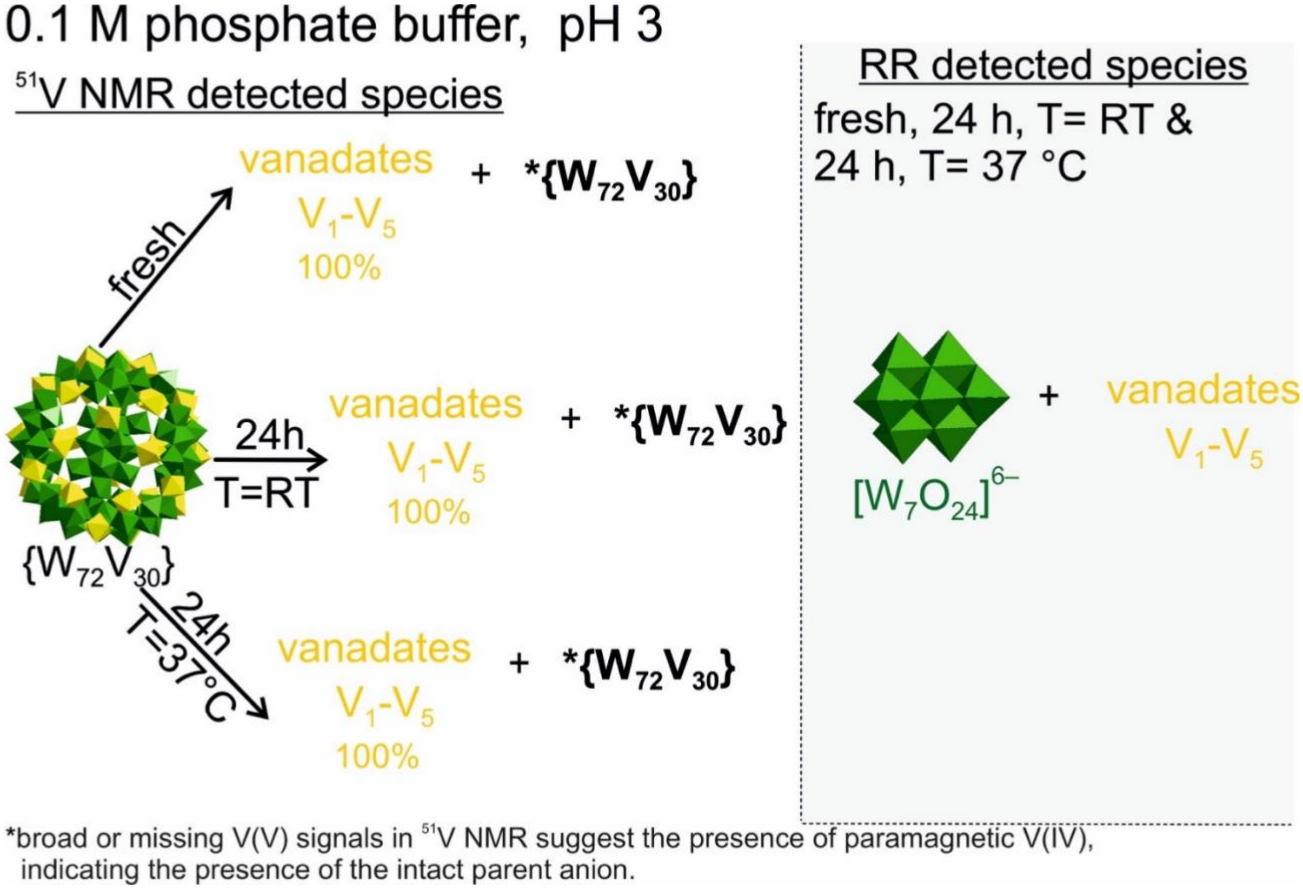

Fig. S65. Rearrangement scheme of $\{W_{72}V_{30}\}$ in 0.1 M sodium phosphate buffer (pH 3). The scheme is based on the collected and analyzed ^{51}V-NMR and resonance Raman data (gray field). The structures of all POMs are shown in **Figures S2** and **S3**. **Tables S20 – S22** give all ^{51}V NMR chemical shifts and percentages of species (**Table S24**) formed based on ^{51}V NMR and **Table S27** gives resonance Raman shifts, and their assignation based on literature data (**Tables S5 – S6**). Only species with a percentage > 10 % are shown separately; other species are separated by the type of addenda metals in the structure. Color code: $\{WO_6\}$, green; $\{VO_6\}$, yellow.

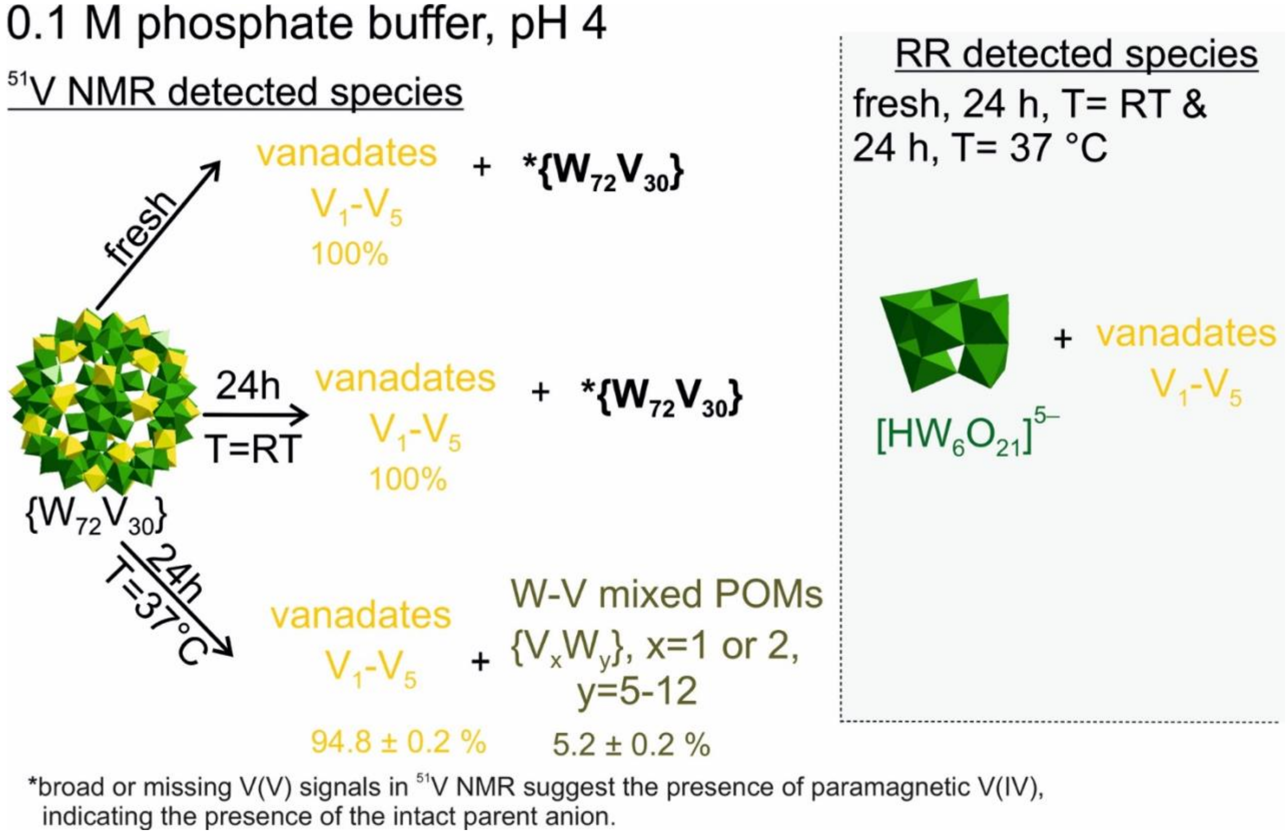

Fig. S66. Rearrangement scheme of $\{W_{72}V_{30}\}$ in 0.1 M sodium phosphate buffers (pH 4).
The scheme is based on the collected and analyzed ^{51}V-NMR and resonance Raman data (gray field). The structures of all POMs are shown in **Figures S2** and **S3**. **Tables S20 – S22** give all ^{51}V NMR chemical shifts and percentages of species (**Table S24**) formed based on ^{51}V NMR and **Table S27** gives resonance Raman shifts, and their assignation based on literature data (**Tables S5 – S6**). Only species with a percentage > 10 % are shown separately; other species are separated by the type of addenda metals in the structure. Color code: $\{WO_6\}$, green; $\{VO_6\}$, yellow.

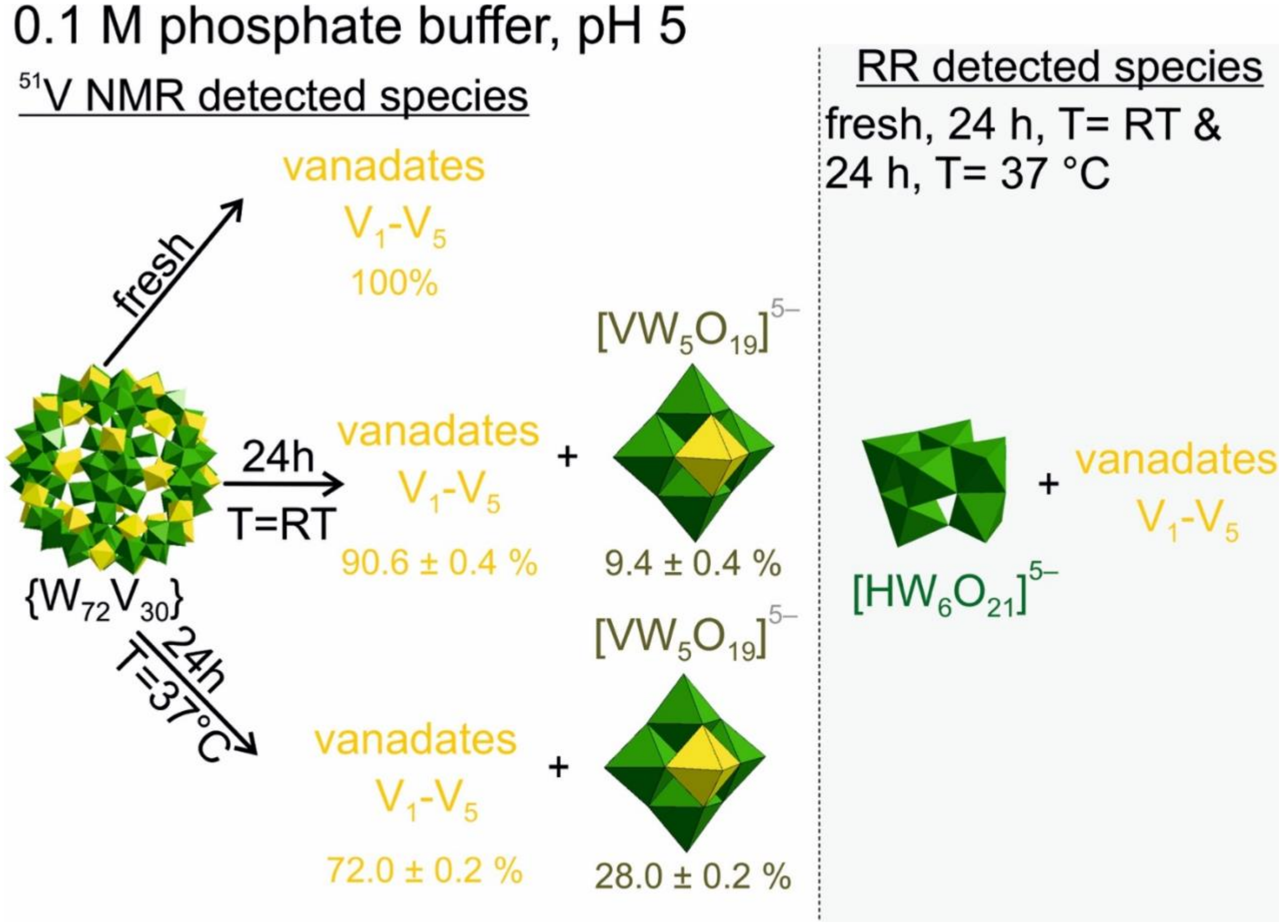

Fig. S67. Rearrangement scheme of {$W_{72}V_{30}$} in 0.1 M sodium phosphate buffers (pH 5). The scheme is based on the collected and analyzed ^{51}V-NMR and resonance Raman data (gray field). The structures of all POMs are shown in **Figures S2** and **S3**. **Tables S20 – S22** give all ^{51}V NMR chemical shifts and percentages of species (**Table S24**) formed based on ^{51}V NMR and **Table S27** gives resonance Raman shifts, and their assignation based on literature data (**Tables S5 – S6**). Only species with a percentage > 10 % are shown separately; other species are separated by the type of addenda metals in the structure. Color code: {WO_6}, green; {VO_6}, yellow.

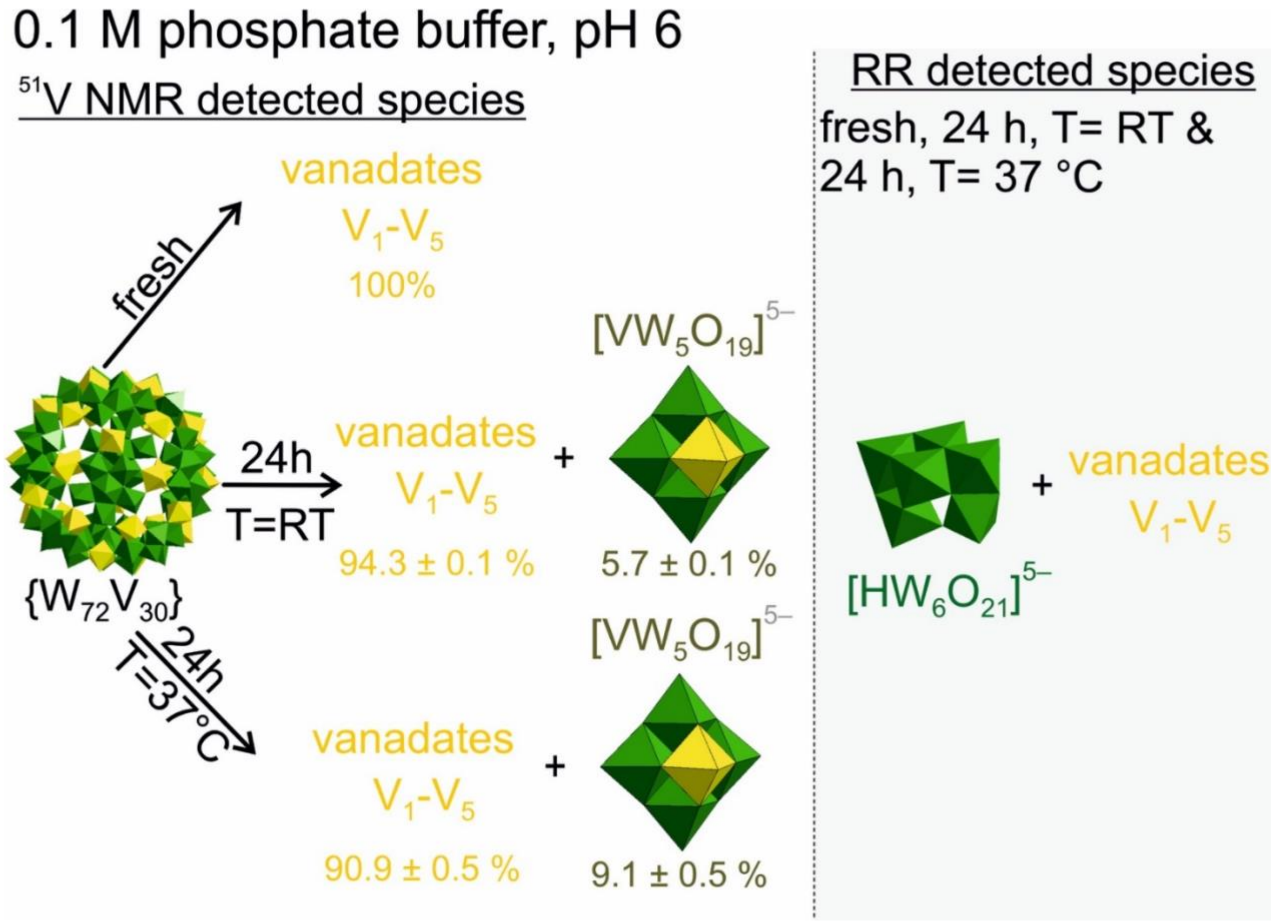

Fig. S68. Rearrangement scheme of $\{W_{72}V_{30}\}$ in 0.1 M sodium phosphate buffer (pH 6). The scheme is based on the collected and analyzed ^{51}V-NMR and resonance Raman data (gray field). The structures of all POMs are shown in **Figures S2** and **S3**. **Tables S20 – S22** give all ^{51}V NMR chemical shifts and percentages of species (**Table S24**) formed based on ^{51}V NMR and **Table S27** gives resonance Raman shifts, and their assignation based on literature data (**Tables S5 – S6**). Only species with a percentage > 10 % are shown separately; other species are separated by the type of addenda metals in the structure. Color code: $\{WO_6\}$, green; $\{VO_6\}$, yellow.

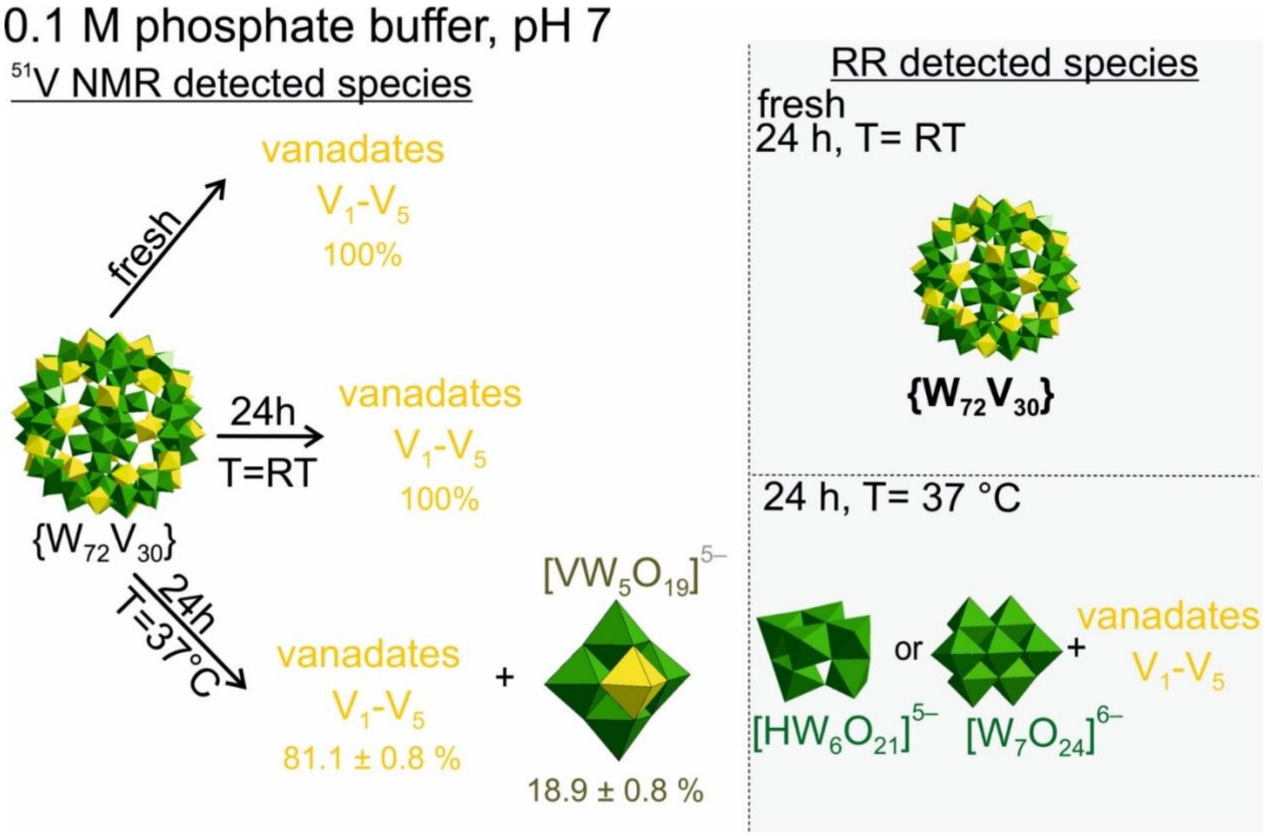

Fig. S69. Rearrangement scheme of {$W_{72}V_{30}$} in 0.1 M sodium phosphate buffer (pH 7). The scheme is based on the collected and analyzed ^{51}V-NMR and resonance Raman data (gray field). The structures of all POMs are shown in **Figures S2** and **S3**. **Tables S20 – S22** give all ^{51}V NMR chemical shifts and percentages of species (**Table S24**) formed based on ^{51}V NMR and **Table S27** gives resonance Raman shifts, and their assignation based on literature data (**Tables S5 – S6**). Only species with a percentage > 10 % are shown separately; other species are separated by the type of addenda metals in the structure. Color code: {WO_6}, green; {VO_6}, yellow.

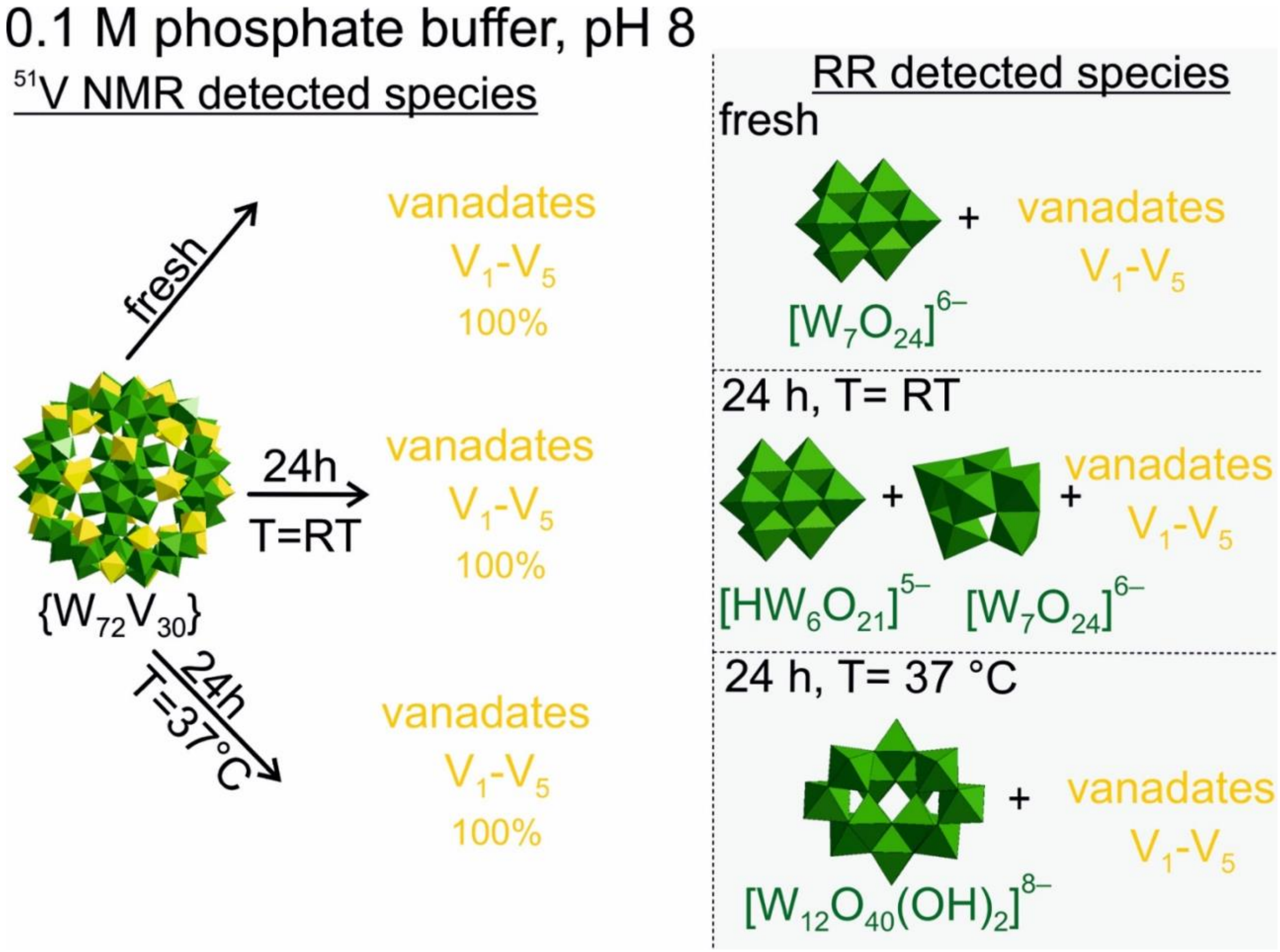

Fig. S70. Rearrangement scheme of $\{W_{72}V_{30}\}$ in 0.1 M sodium phosphate buffer (pH 8).
The scheme is based on the collected and analyzed ^{51}V-NMR and resonance Raman data (gray field). The structures of all POMs are shown in **Figures S2** and **S3**. **Tables S20 – S22** give all ^{51}V NMR chemical shifts and percentages of species (**Table S24**) formed based on ^{51}V NMR and **Table S27** gives resonance Raman shifts, and their assignation based on literature data (**Tables S5 – S6**). Only species with a percentage > 10 % are shown separately; other species are separated by the type of addenda metals in the structure. Color code: $\{WO_6\}$, green; $\{VO_6\}$, yellow.

6.4.3. $\{W_{72}V_{30}\}$ rearrangement schemes in 0.1 M acetic acid – sodium acetate buffers

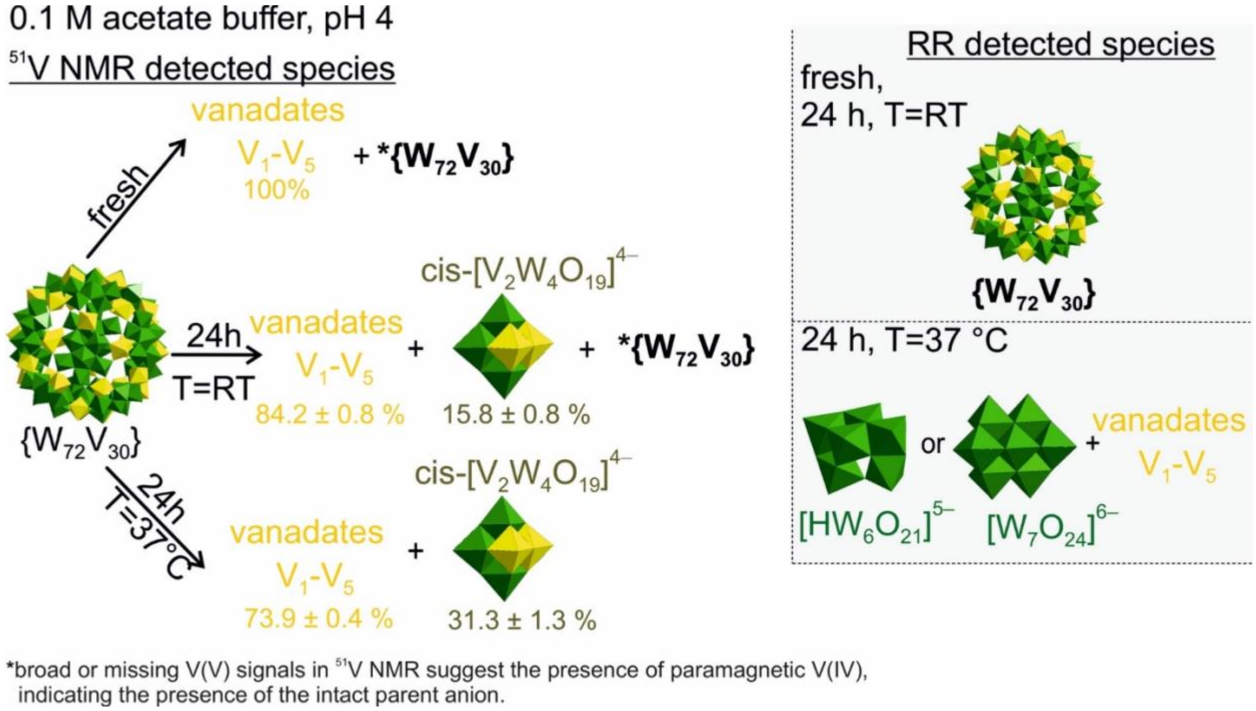

Fig. S71. Rearrangement scheme of $\{W_{72}V_{30}\}$ in 0.1 M acetic acid – sodium acetate buffer (pH 4).

The scheme is based on the collected and analyzed ^{51}V-NMR and resonance Raman data (gray field). The structures of all POMs are shown in **Figures S2** and **S3**. **Tables S20 – S22** give all ^{51}V NMR chemical shifts and percentages of species (**Table S25**) formed based on ^{51}V NMR and **Table S27** gives resonance Raman shifts, and their assignation based on literature data (**Tables S5 – S6**). Only species with a percentage > 10 % are shown separately; other species are separated by the type of addenda metals in the structure. Color code: $\{WO_6\}$, green; $\{VO_6\}$, yellow.

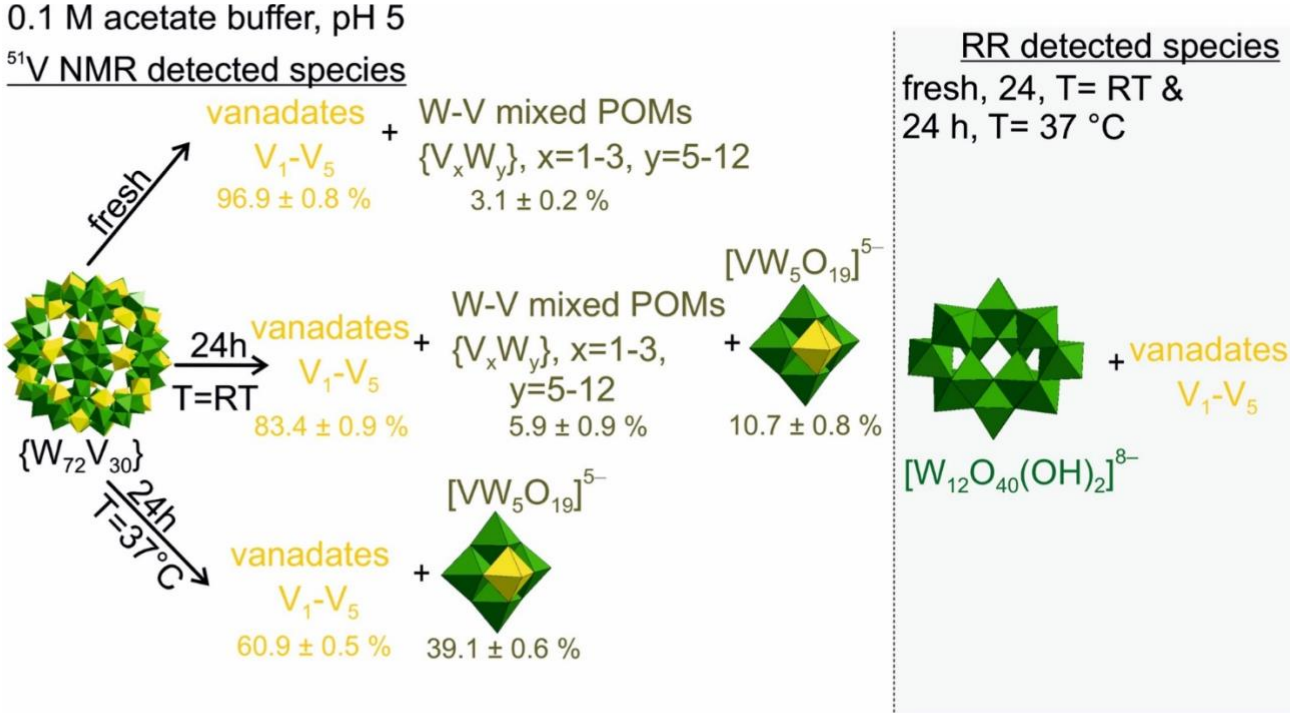

Fig. S72. Rearrangement scheme of $\{W_{72}V_{30}\}$ in 0.1 M acetic acid – sodium acetate buffer (pH 5).

The scheme is based on the collected and analyzed ^{51}V-NMR and resonance Raman data (gray field). The structures of all POMs are shown in **Figures S2** and **S3**. **Tables S20 – S22** give all ^{51}V NMR chemical shifts and percentages of species (**Table S25**) formed based on ^{51}V NMR and **Table S27** gives resonance Raman shifts, and their assignation based on literature data (**Tables S5 – S6**). Only species with a percentage > 10 % are shown separately; other species are separated by the type of addenda metals in the structure. Color code: $\{WO_6\}$, green; $\{VO_6\}$, yellow.

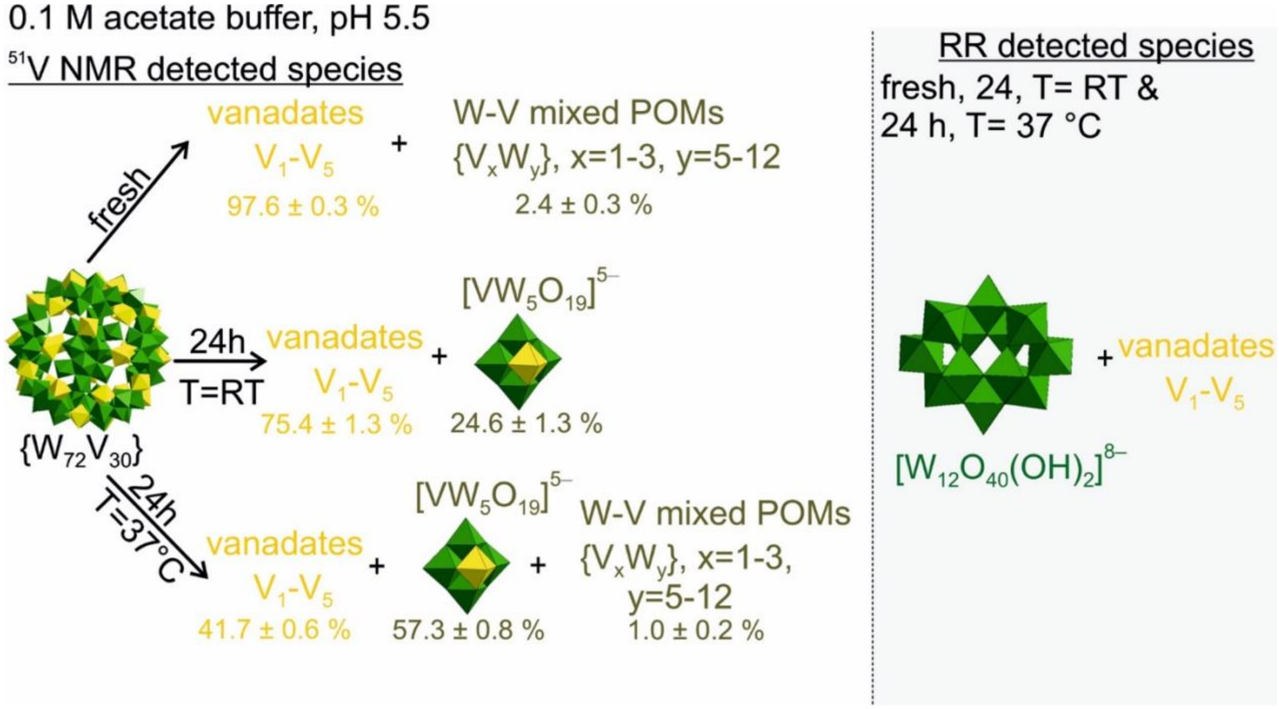

Fig. S73. Rearrangement scheme of $\{W_{72}V_{30}\}$ in 0.1 M acetic acid – sodium acetate buffer (pH 5.5).

The scheme is based on the collected and analyzed ^{51}V-NMR and resonance Raman data (gray field). The structures of all POMs are shown in **Figures S2** and **S3**. **Tables S20 – S22** give all ^{51}V NMR chemical shifts and percentages of species (**Table S25**) formed based on ^{51}V NMR and **Table S27** gives resonance Raman shifts, and their assignation based on literature data (**Tables S5 – S6**). Only species with a percentage > 10 % are shown separately; other species are separated by the type of addenda metals in the structure. Color code: $\{WO_6\}$, green; $\{VO_6\}$, yellow.

6.4.4. {W72V30} rearrangement schemes in 0.1 M Tris – HCl buffers

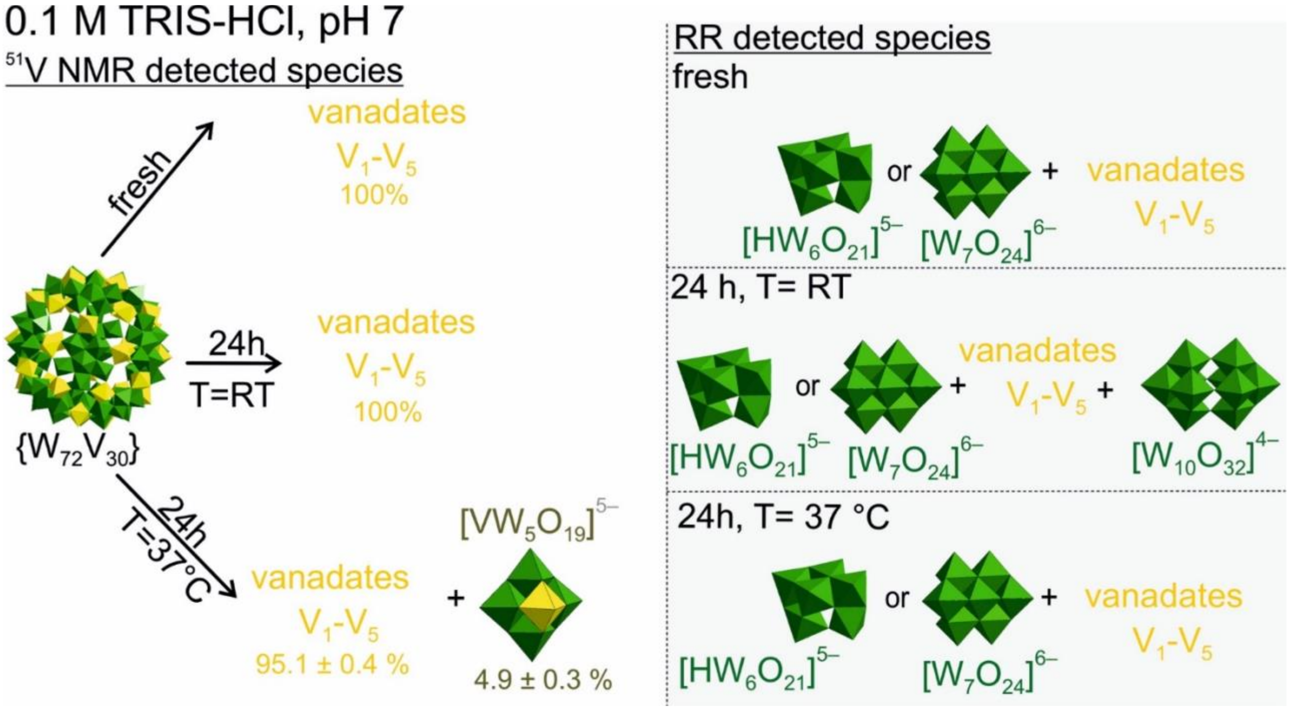

Fig. S74. Rearrangement scheme of {Mo72V30} in 0.1 M Tris–HCl buffer (pH 7).

The scheme is based on the collected and analyzed [51]V-NMR and resonance Raman data (gray field). The structures of all POMs are shown in **Figures S2** and **S3**. **Tables S20 – S22** give all [51]V NMR chemical shifts and percentages of species (**Table S26**) formed based on [51]V NMR and **Table S27** gives resonance Raman shifts, and their assignation based on literature data (**Tables S5 – S6**). Only species with a percentage > 10 % are shown separately; other species are separated by the type of addenda metals in the structure. Color code: {WO_6}, green; {VO_6}, yellow.

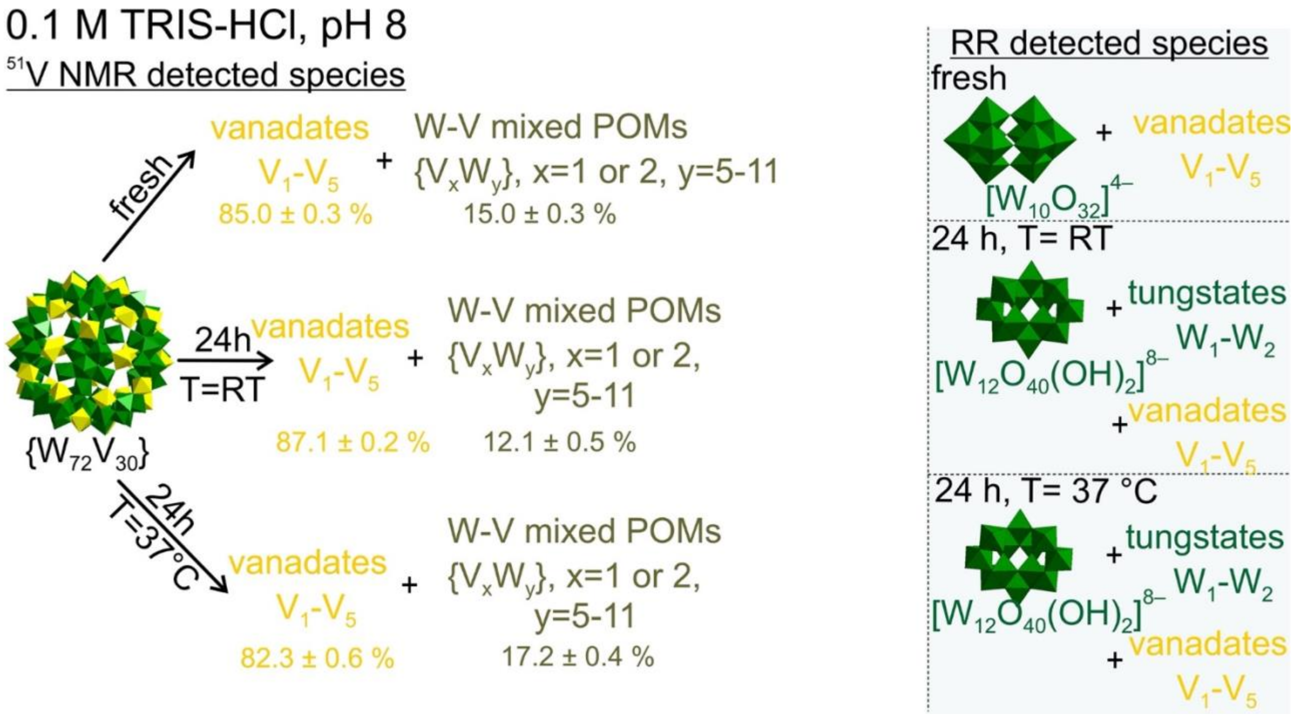

Fig. S75. Rearrangement scheme of $\{W_{72}V_{30}\}$ in 0.1 M Tris–HCl buffer (pH 8).

The scheme is based on the collected and analyzed ^{51}V-NMR and resonance Raman data (gray field). The structures of all POMs are shown in **Figures S2** and **S3**. **Tables S20 – S22** give all ^{51}V NMR chemical shifts and percentages of species (**Table S26**) formed based on ^{51}V NMR and **Table S27** gives resonance Raman shifts, and their assignation based on literature data (**Tables S5 – S6**). Only species with a percentage > 10 % are shown separately; other species are separated by the type of addenda metals in the structure. Color code: $\{WO_6\}$, green; $\{VO_6\}$, yellow.

6.4.5. $\{W_{72}V_{30}\}$ rearrangement schemes in 0.1 M HEPES buffers

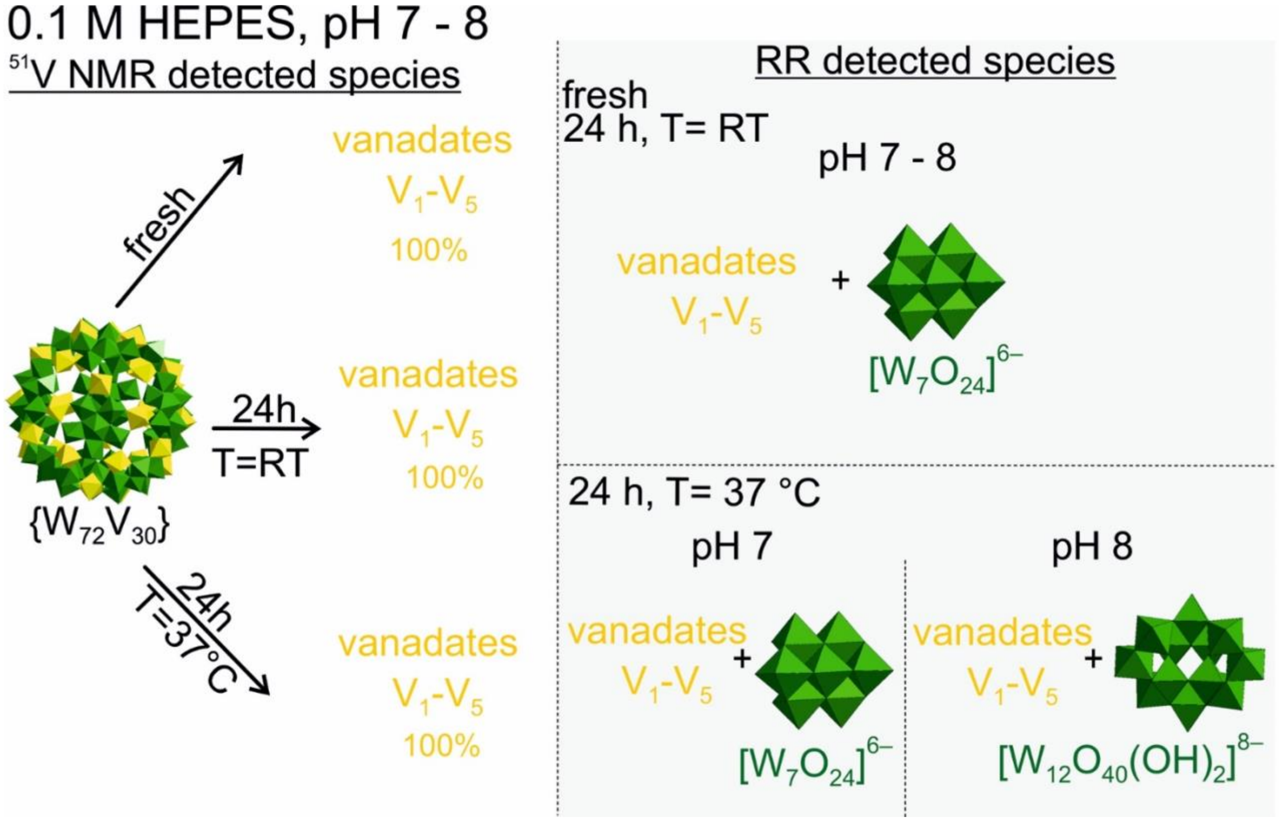

Fig. S76. Rearrangement scheme of $\{W_{72}V_{30}\}$ in 0.1 M HEPES buffers (pH 7 and 8).

The scheme is based on the collected and analyzed ^{51}V-NMR and resonance Raman data (gray field). The structures of all POMs are shown in **Figures S2** and **S3**. **Tables S20 – S22** give all ^{51}V NMR chemical shifts and percentages of species (**Table S26**) formed based on ^{51}V NMR and **Table S27** gives resonance Raman shifts, and their assignation based on literature data (**Tables S5 – S6**). Only species with a percentage > 10 % are shown separately; other species are separated by the type of addenda metals in the structure. Color code: $\{WO_6\}$, green; $\{VO_6\}$, yellow.

6.5. UV-vis spectroscopy of $\{W_{72}V_{30}\}$ in aqueous solutions

UV-vis data was collected every 30 min for 24 h. The “fresh solutions” were measured after 3 min of stirring until the $\{W_{72}V_{30}\}$ was dissolved entirely and 1 min of incubation in the UV-vis spectrometer in total 4 min).

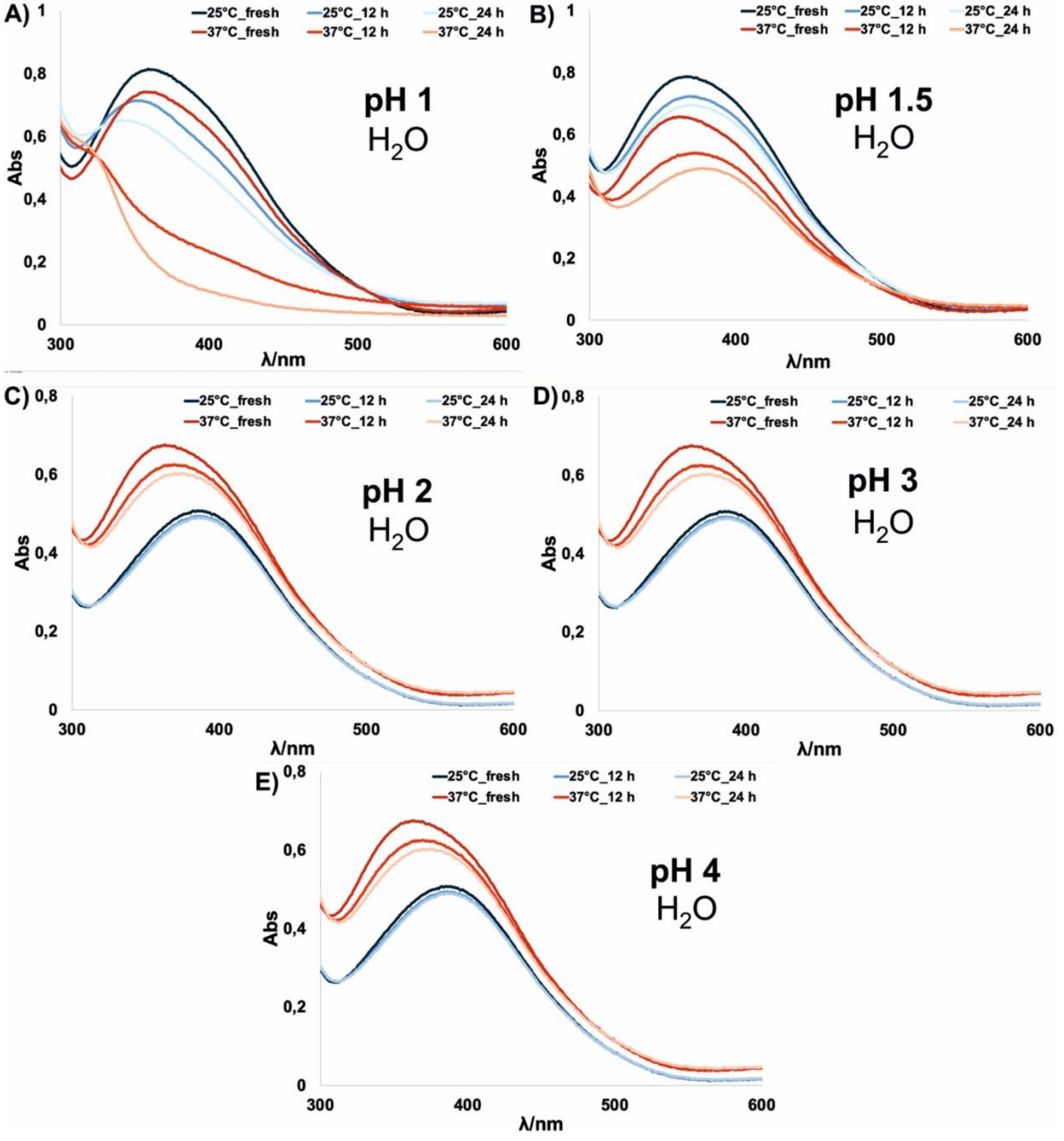

Fig. S77. UV-vis spectra of $\{W_{72}V_{30}\}$ in H_2O.

UV-vis spectra of 10 μM $\{W_{72}V_{30}\}$ in H_2O at T = 25 °C and T = 37 °C either until complete oxidation (loss of absorbance band) or 0 – 12 – 24 h period: A) H_2O pH 1, B) H_2O pH 1.5, C) H_2O pH 2, D) H_2O pH 3, E) H_2O pH 4.

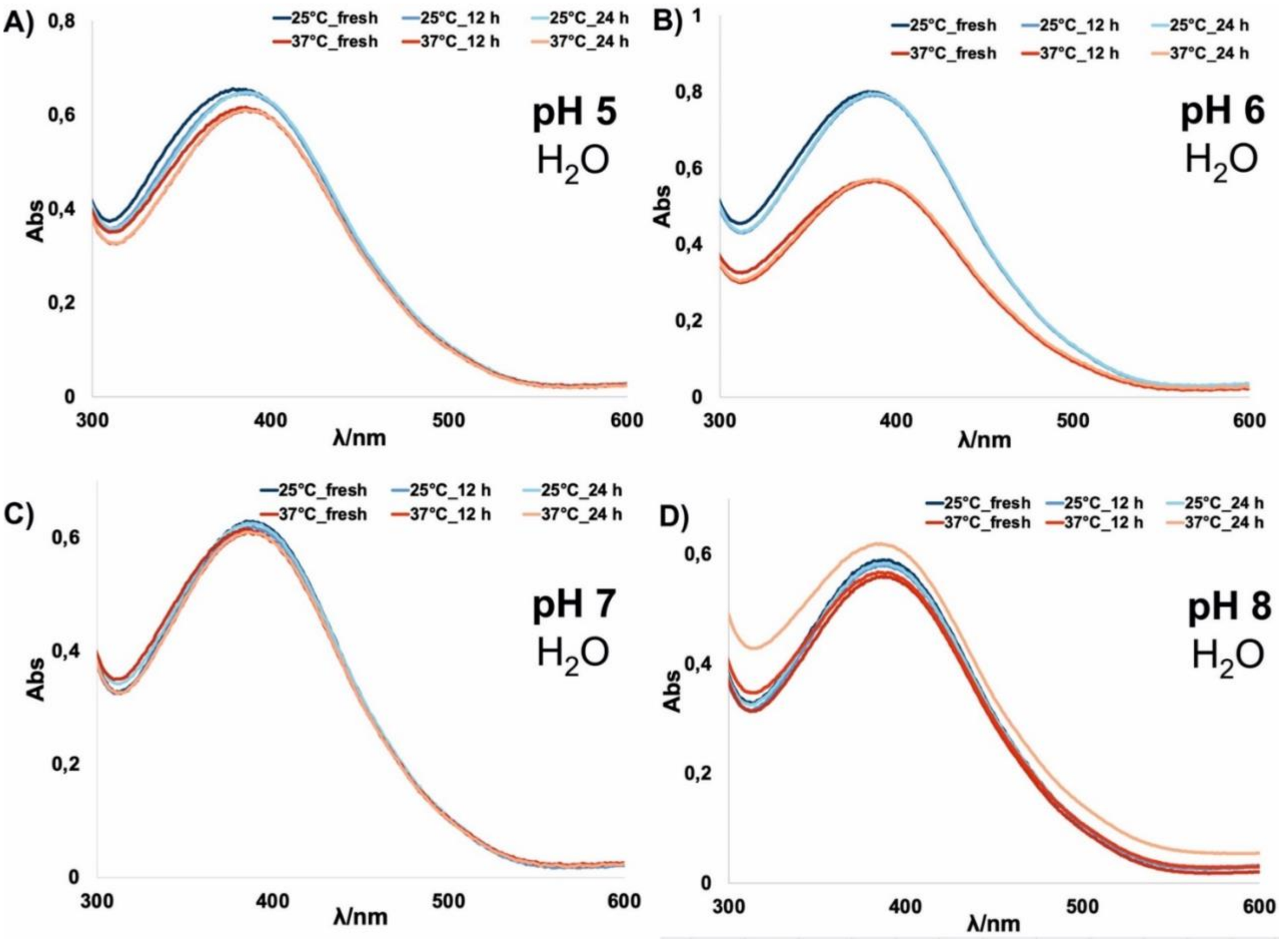

Fig. S78. UV-vis spectra of $\{W_{72}V_{30}\}$ in H_2O.

UV-vis spectra of 10 μM $\{W_{72}V_{30}\}$ in H_2O at T = 25 °C and T = 37 °C either until complete oxidation (loss of absorbance band) or 0 – 12 – 24 h period: A) H_2O pH 5, B) H_2O pH 6, C) H_2O pH 7 and D) H_2O pH 8.

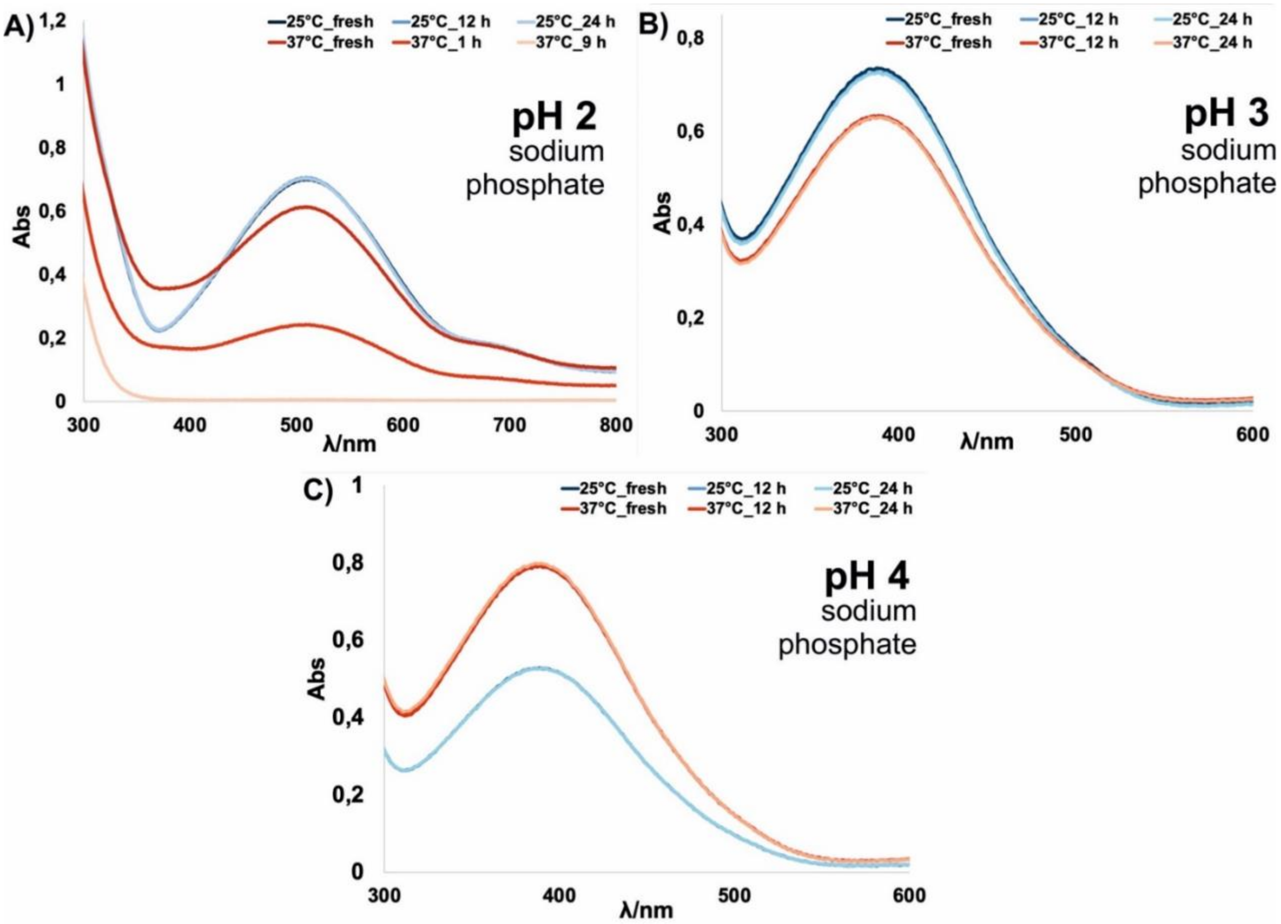

Fig. S79. UV-vis spectra of {$W_{72}V_{30}$} in sodium phosphate buffer

UV-vis spectra of 10 μM {$W_{72}V_{30}$} in H_2O and 0.1 sodium phosphate buffers at T = 25 °C and T = 37 °C either until complete oxidation (loss of absorbance band) or 0 – 12 – 24 h period: A) 0.1 M Sodium phosphate buffer pH 2, B) 0.1 M Sodium phosphate buffer pH 3 and C) 0.1 M Sodium phosphate buffer pH 4.

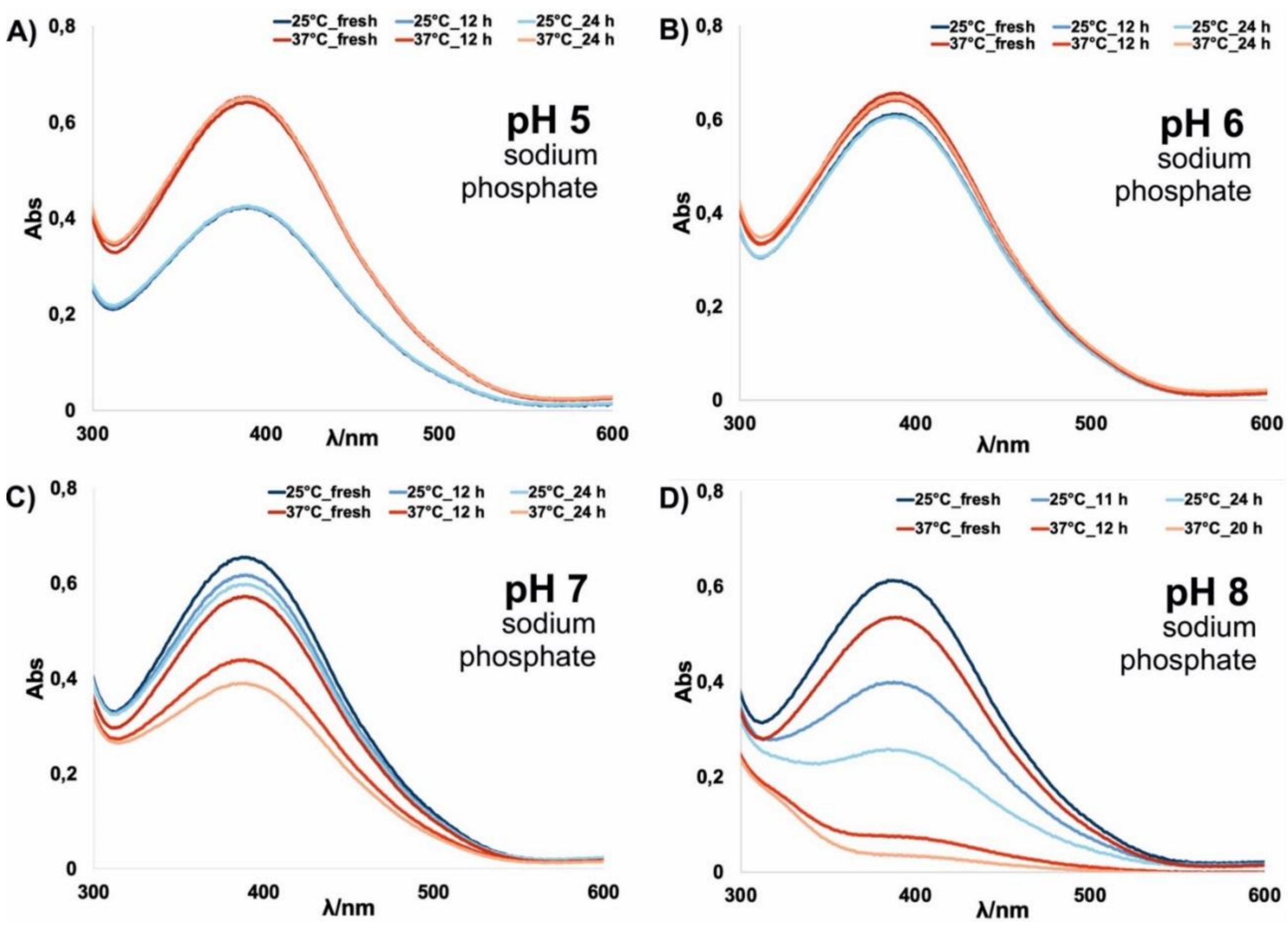

Fig. S80. UV-vis spectra of {$W_{72}V_{30}$} in sodium phosphate buffer.

UV-vis spectra of 10 μM {$W_{72}V_{30}$} in 0.1 sodium phosphate buffers at T = 25 °C and T = 37 °C either until complete oxidation (loss of absorbance band) or 0 – 12 – 24 h period: A) 0.1 M sodium phosphate buffer pH 5, B) 0.1 M sodium phosphate buffer pH 6, C) 0.1 M sodium phosphate buffer pH 7 and D) 0.1 M sodium phosphate buffer pH 8.

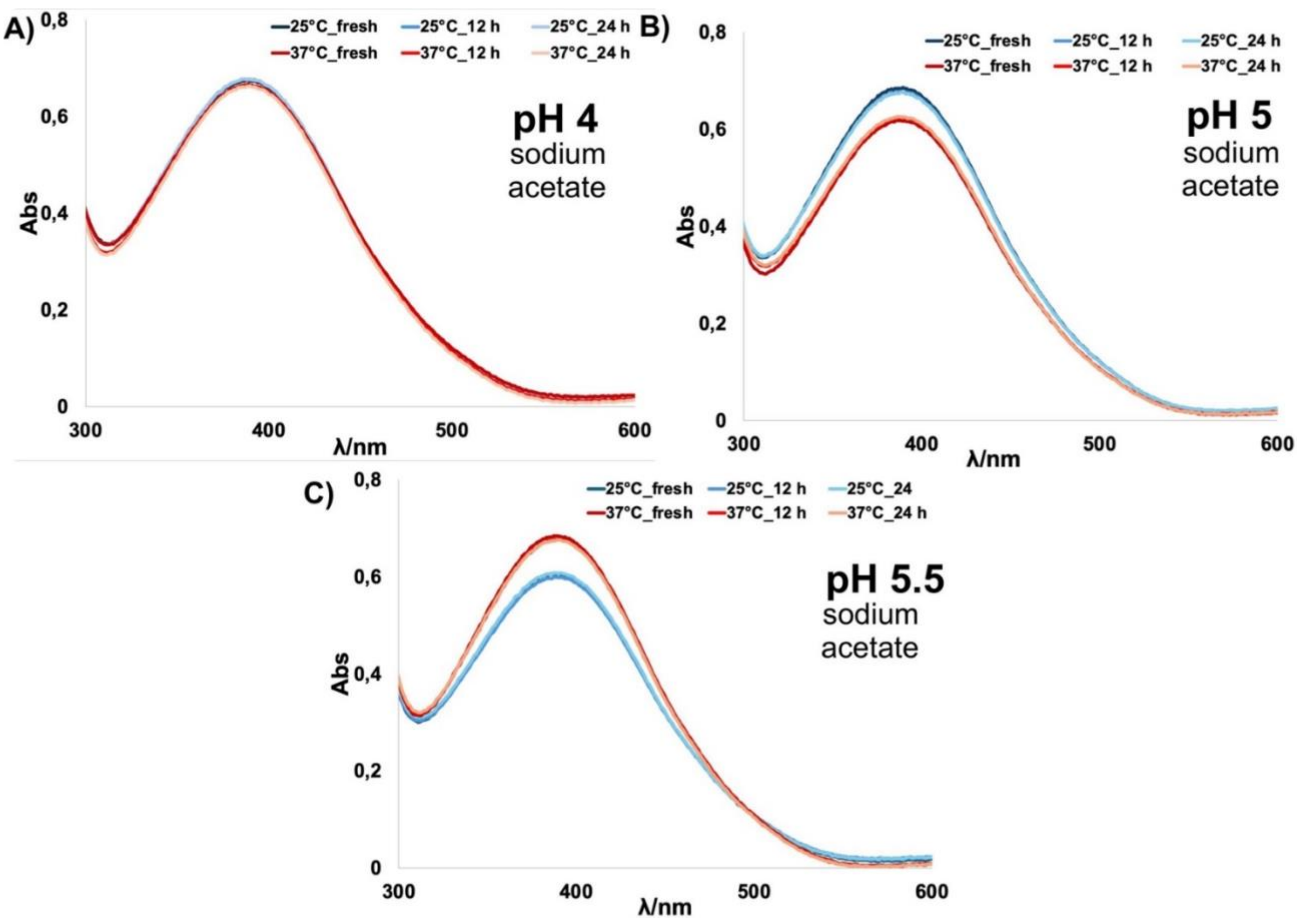

Fig. S81. UV-vis spectra of {$W_{72}V_{30}$} in acetic acid – sodium acetate buffer.

UV-vis spectra of 10 μM {$W_{72}V_{30}$} in 0.1 acetic acid – sodium acetate buffers at T = 25 °C and T = 37 °C either until complete oxidation (loss of absorbance band) or 0 – 12 – 24 h period: A) 0.1 M acetic acid – sodium acetate buffer pH 4, B) 0.1 M acetic acid – sodium acetate buffer pH 5, and C) 0.1 M acetic acid – sodium acetate buffer pH 5.5.

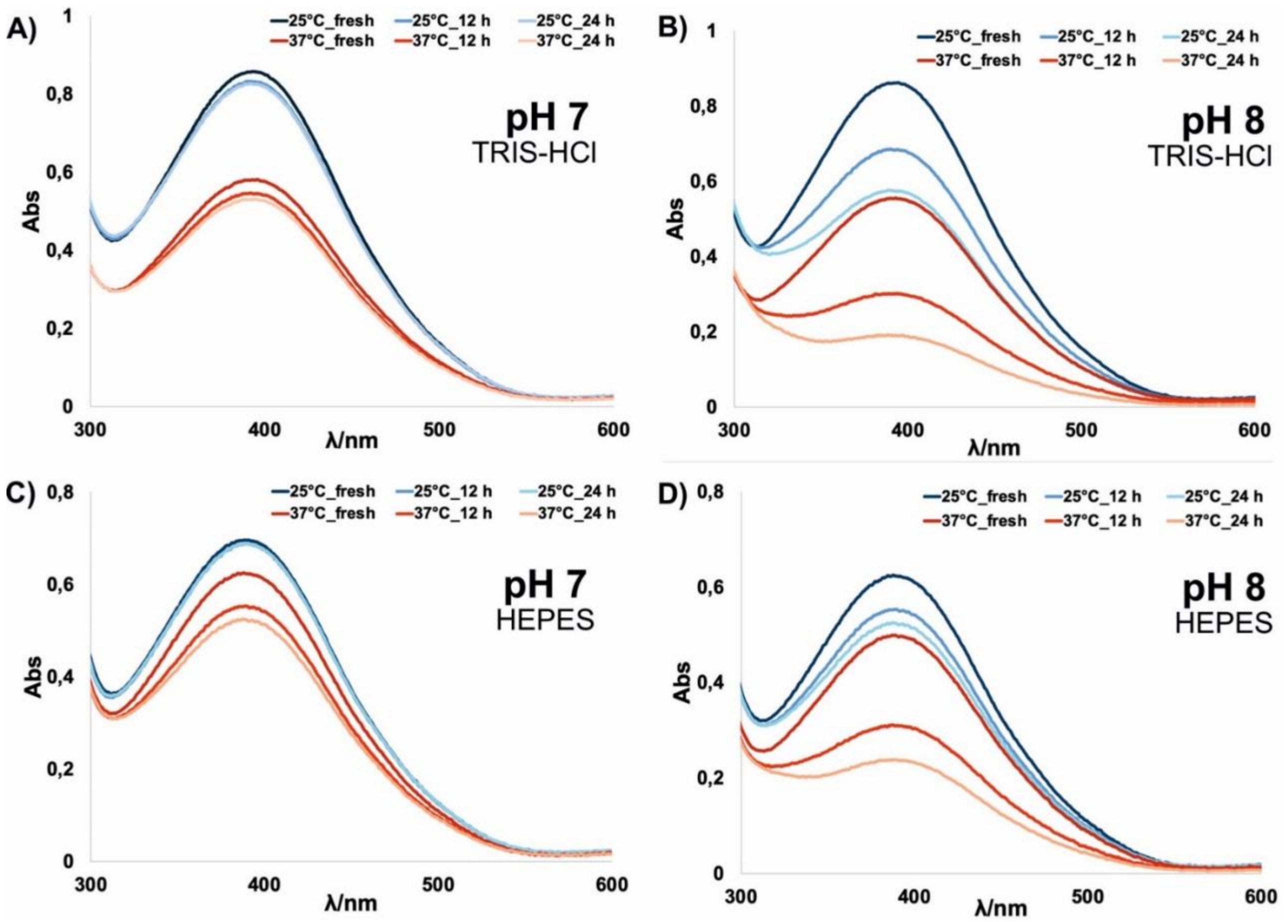

Fig. S82. UV-vis spectra of $\{W_{72}V_{30}\}$ in Tris-HCl and HEPES buffers.

UV-vis spectra of 10 μM $\{W_{72}V_{30}\}$ in 0.1 M Tris – HCl and 0.1 M HEPES buffers at T = 25 °C and T = 37 °C either until complete oxidation (loss of absorbance band) or 0 – 12 – 24 h period: A) 0.1 M Tris – HCl buffer pH 7, B) 0.1 M Tris – HCl buffer pH 8, C) 0.1 M HEPES buffer pH 7 and D) 0.1 M HEPES buffer pH 8.

6.6. Summary of ^{51}V-NMR, resonance Raman and UV-VIS spectroscopy experiments

Table S28. Summarized comparison of results of three analytical methods: ^{51}V-NMR, Resonance Raman, UV – nir – VIS and ESI-MS (fresh {$W_{72}V_{30}$} non-buffred solutions with pH 1 – 8).

H₂O								
		NMR (0.15 mM {$W_{72}V_{30}$})		Resonance Raman (0.15 mM {$W_{72}V_{30}$})		UV – VIS (10 µM {$W_{72}V_{30}$})	ESI-MS (1mg/mL {$W_{72}V_{30}$})
Sample condition		Intact {$W_{72}V_{30}$}	Post-decomposition POM species	Intact {$W_{72}V_{30}$}	Post-decomposition POM species	Reduced/ Not reduced	Detected {$W_{72}V_{30}$}
pH 1	Fresh	Yes[a]	Yes	Yes	No	Yes	Yes[b]
	24 h at RT	Yes[a]	Yes	Yes	No	Yes	-
	24 h at 37 °C	No	Yes	Yes	No	No	-
pH 1.5	Fresh	Yes[a]	Yes	Yes	No	Yes	Yes[b]
	24 h at RT	Yes[a]	Yes	Yes	No	Yes	-
	24 h at 37 °C	No	Yes	Yes	No	Yes	-
pH 2	Fresh	Yes[a]	Yes	Yes	No	Yes	Yes[b]
	24 h at RT	Yes[a]	Yes	Yes	No	Yes	-
	24 h at 37 °C	No	Yes	Yes	No	Yes	-
pH 3	Fresh	Yes[a]	Yes	Yes	No	Yes	Yes
	24 h at RT	Yes[a]	Yes	Yes	No	Yes	-
	24 h at 37 °C	Yes[a]	Yes	Yes	No	Yes	-
pH 4	Fresh	Yes[a]	Yes	Yes	No	Yes	Yes
	24 h at RT	Yes[a]	Yes	Yes	No	Yes	-
	24 h at 37 °C	No	Yes	Yes	No	Yes	-
pH 5	Fresh	No	Yes	Yes	No	Yes	Yes
	24 h at RT	No	Yes	Yes	No	Yes	-
	24 h at 37 °C	No	Yes	Yes	No	Yes	-
pH 6	Fresh	No	Yes	Yes	Yes	Yes	Yes
	24 h at RT	No	Yes	Yes	Yes	Yes	-
	24 h at 37 °C	No	Yes	Yes	Yes	Yes	-
pH 7	Fresh	No	Yes	Yes	No	Yes	Yes
	24 h at RT	No	Yes	Yes	No	Yes	-
	24 h at 37 °C	No	Yes	Yes	No	Yes	-
pH 8	Fresh	No	Yes	Yes	Yes	Yes	Yes
	24 h at RT	No	Yes	Yes	Yes	Yes	-
	24 h at 37 °C	No	Yes	Yes	Yes	Yes	-
0.1 M sodium phosphate buffers							

		NMR (0.15 mM $\{W_{72}V_{30}\}$)		Resonance Raman (0.15 mM $\{W_{72}V_{30}\}$)		UV – VIS (10 µM $\{W_{72}V_{30}\}$)	ESI-MS (1mg/mL $\{W_{72}V_{30}\}$)
Sample condition		Intact $\{W_{72}V_{30}\}$	Post-decomposition POM species	Intact $\{W_{72}V_{30}\}$	Post-decomposition POM species	Reduced/ Not reduced	Detected $\{W_{72}V_{30}\}$
pH 2	Fresh	Yes[a]	No	Yes	Yes	Yes	-
	24 h at RT	Yes[a]	No	No	Yes	Yes	-
	24 h at 37 °C	Yes[a]	No	No	Yes	Yes	-
pH 3	Fresh	Yes[a]	No	No	Yes	Yes	-
	24 h at RT	Yes[a]	No	No	Yes	Yes	-
	24 h at 37 °C	Yes[a]	Yes	No	Yes	Yes	-
pH 4	Fresh	Yes[a]	Yes	No	Yes	Yes	-
	24 h at RT	Yes[a]	Yes	No	Yes	Yes	-
	24 h at 37 °C	No	Yes	No	Yes	Yes	-
pH 5	Fresh	No	Yes	No	Yes	Yes	-
	24 h at RT	No	Yes	No	Yes	Yes	-
	24 h at 37 °C	No	Yes	No	Yes	Yes	-
pH 6	Fresh	No	Yes	No	Yes	Yes	-
	24 h at RT	No	Yes	No	Yes	Yes	-
	24 h at 37 °C	No	Yes	No	Yes	Yes	-
pH 7	Fresh	No	Yes	Yes	Yes	Yes	-
	24 h at RT	No	Yes	Yes	Yes	Yes	-
	24 h at 37 °C	No	Yes	No	Yes	Yes	-
pH 8	Fresh	No	Yes	No	Yes	Yes	-
	24 h at RT	No	Yes	No	Yes	Yes	-
	24 h at 37 °C	No	Yes	No	Yes	No	-

0.1 M acetic acid – sodium acetate buffers

		NMR (0.20 mM $\{W_{72}V_{30}\}$)		Resonance Raman (0.20 mM $\{W_{72}V_{30}\}$)		UV – VIS (10 µM $\{W_{72}V_{30}\}$)	ESI-MS (1mg/mL $\{W_{72}V_{30}\}$)
Sample condition		Intact $\{W_{72}V_{30}\}$	Post-decomposition POM species	Intact $\{W_{72}V_{30}\}$	Post-decomposition POM species	Reduced/ Not reduced	Detected $\{W_{72}V_{30}\}$
pH 4	Fresh	Yes[a]	Yes	Yes	No	Yes	-
	24 h at RT	Yes[a]	Yes	Yes	No	Yes	-
	24 h at 37 °C	No	Yes	No	Yes	Yes	-
pH 5	Fresh	No	Yes	No	Yes	Yes	-
	24 h at RT	No	Yes	No	Yes	Yes	-
	24 h at 37 °C	No	Yes	No	Yes	Yes	-
pH 5.5	Fresh	No	Yes	No	Yes	Yes	-
	24 h at RT	No	Yes	No	Yes	Yes	-
	24 h at 37 °C	No	Yes	No	Yes	Yes	-

0.1 M Tris – HCl buffers

		NMR (0.15 mM $\{W_{72}V_{30}\}$)		Resonance Raman (0.15 mM $\{W_{72}V_{30}\}$)		UV – VIS (10 μM $\{W_{72}V_{30}\}$)	ESI-MS (1mg/mL $\{W_{72}V_{30}\}$)
Sample condition		Intact $\{W_{72}V_{30}\}$	Post-decomposition POM species	Intact $\{W_{72}V_{30}\}$	Post-decomposition POM species	Reduced/ Not reduced	Detected $\{W_{72}V_{30}\}$
pH 7	Fresh	No	Yes	No	Yes	Yes	-
	24 h at RT	No	Yes	No	Yes	Yes	-
	24 h at 37 °C	No	Yes	No	Yes	Yes	-
pH 8	Fresh	No	Yes	No	Yes	Yes	-
	24 h at RT	No	Yes	No	Yes	Yes	-
	24 h at 37 °C	No	Yes	No	Yes	Yes	-

0.1 M HEPES buffers

		NMR (0.15 mM $\{W_{72}V_{30}\}$)		Resonance Raman (0.15 mM $\{W_{72}V_{30}\}$)		UV – VIS (10 μM $\{W_{72}V_{30}\}$)	ESI-MS (1mg/mL $\{W_{72}V_{30}\}$)
Sample condition		Intact $\{W_{72}V_{30}\}$	Post-decomposition POM species	Intact $\{W_{72}V_{30}\}$	Post-decomposition POM species	Reduced/ Not reduced	Detected $\{W_{72}V_{30}\}$
pH 7	Fresh	No	Yes	No	Yes	Yes	-
	24 h at RT	No	Yes	No	Yes	Yes	-
	24 h at 37 °C	No	Yes	No	Yes	Yes	-
pH 8	Fresh	No	Yes	No	Yes	Yes	-
	24 h at RT	No	Yes	No	Yes	Yes	-
	24 h at 37 °C	No	Yes	No	Yes	Yes	-

a − presence of Keplerate anion due to either shape of ^{51}V NMR spectra or no ^{51}V NMR signal in 0.15 mM aqueous solution at pH ≤ 2 because of paramagnetic V(IV) in solution.
b – Keplerate anions visible only when we use LC-MS. Not visible during ESI-MS.

6.7. Electrospray ionization-mass spectrometry experiments

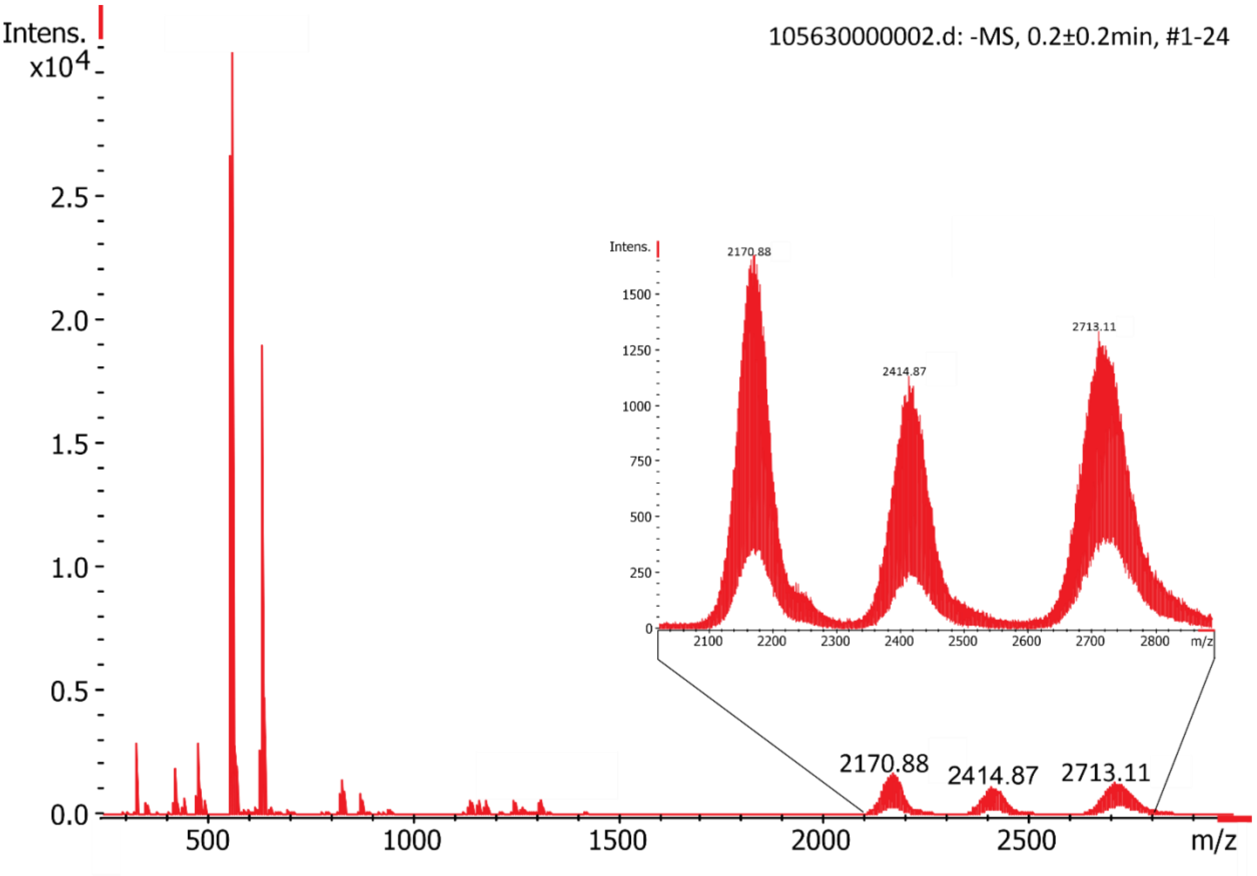

Fig. S83. ESI-MS spectra of 1 mg/mL $\{W_{72}V_{30}\}$ fresh solution in H_2O at pH 3.

The pH of solutions was adjusted using acid (formic acid for pH 3). The spectra were measured ~30 min after sample preparation.

Table S29. ESI-MS data of 1 mg/ mL $\{W_{72}V_{30}\}$ fresh solution in H_2O with pH 3.

ESI-MS data of $\{W_{72}V_{30}\}$ in H_2O with pH 3. The pH of solutions was adjusted using acid (HCl for pH ≤ 2 and formic acid for pH 3 – 6) or NH_3(aq) (pH > 7). The spectra were measured ~30 min after sample preparation. The ESI-MS spectra of $\{W_{72}V_{30}\}$ solutions with pH ≤ 2 showed no presence of Keplerate anions and it could be that the higher concentration of Cl^- ions hinder the big anions (m/z) from detector. The Keplerate anions only become visible during LC-ESI-MS measurement, matching anions visible in ESI-MS spectra in H_2O with pH 3.

**pH 3**				
**Anion charge**	**POM anion formula**	**m/z(exp)**	**m/z(calc)**	**m/z (average)**
**-10**	**$\{Na_xK_yH_{108-x-y}W_{72}V_{30}S_{10}O_{365}\}^{10-}$, x=10 – 27, y=7 – 8**	2155.9 – 2189.4	2155.9 – 2189.4	**2162.8**

	**$\{Na_xK_yH_{104-x-y}W_{72}V_{30}S_{10}O_{363}\}^{10-}$, x=2 − 21, y=4 − 8**	2134.7 – 2161.2	2134.7 – 2161.2	
	**$\{Na_xK_yH_{98-x-y}W_{72}V_{30}S_{11}O_{363}\}^{10-}$, x=3 − 15, y=3 − 7**	2128.1 – 2153.3	2128.1 – 2153.3	
	**$\{Na_xK_yH_{96-x-y}W_{72}V_{30}S_{12}O_{365}\}^{10-}$, x= 0 – 22, y=1 – 7**	2131.5 – 2168.4	1470.5 – 1488.0	
	**$\{Na_xK_yH_{109-x-y}W_{72}V_{30}S_{10}O_{365}\}^{9-}$, x=10 − 24, y=7 − 8**	2395.5 – 2425.5	2395.5 – 2425.5	
	**$\{Na_xK_yH_{105-x-y}W_{72}V_{30}S_{10}O_{363}\}^{9-}$, x=6 − 21, y=4 − 8**	2381.7 – 2409.0	2381.7 – 2409.0	
**-9**	**$\{Na_xK_yH_{103-x-y}W_{72}V_{30}S_{11}O_{365}\}^{9-}$, x=2 − 28, y=2 − 8**	2378.9 – 2421.2	2378.9 – 2421.2	**2414.9**
	**$\{Na_xK_yH_{99-x-y}W_{72}V_{30}S_{11}O_{363}\}^{9-}$, x=0 – 6, y=5 – 8**	2372.6 – 2376.2	2372.6 – 2376.2	
	**$\{Na_xK_yH_{97-x-y}W_{72}V_{30}S_{12}O_{365}\}^{9-}$, x=0 – 8, y=4 – 8**	2372.6 – 2379.5	2372.6 – 2379.5	
	**$\{Na_xK_yH_{110-x-y}W_{72}V_{30}S_{10}O_{365}\}^{8-}$, x=10 − 17, y= 7 − 8**	2699.1 – 2712.8	2699.1 – 2712.8	
	**$\{Na_xK_yH_{106-x-y}W_{72}V_{30}S_{10}O_{363}\}^{8-}$, x=6 − 28, y=3 − 7**	2674.8 – 2716.2	2674.8 – 2716.2	**2712.1**
**-8**	**$\{Na_xK_yH_{104-x-y}W_{72}V_{30}S_{11}O_{365}\}^{8-}$, x=2 − 28, y= 2 − 8**	2676.9 – 2719.4	2676.9 – 2719.4	
	**$\{Na_xK_yH_{100-x-y}W_{72}V_{30}S_{12}O_{363}\}^{8-}$, x=5 − 24, y=2 – 5**	2662.5 – 2703.7	2662.5 – 2703.7	
	**$\{Na_xK_yH_{98-x-y}W_{72}V_{30}S_{12}O_{365}\}^{8-}$, x=4 − 27, y=0 − 6**	2668.3 – 2710.2	2668.3 – 2710.2	

Table S30. ESI-MS data overview of 1 mg/ mL $\{W_{72}V_{30}\}$ fresh solution in H_2O with pH 1 – 8. Overview of all Keplerate anions detected by ESI-MS in fresh solutions of 1 mg/ mL $\{W_{72}V_{30}\}$ in H_2O with pH 1 – 8. The m/z of all POM anions formula (for pH 3) are listed in **Table S29**. Legend: − anion not detected; ✓ anion detected.

Anion charge	POM anion formula	*pH 1	*pH 1.5	*pH 2	pH 3	pH 4	pH 5	pH 6	pH 7	pH 8
	**$\{Na_xK_yH_{108-x-y}W_{72}V_{30}S_{10}O_{365}\}^{10-}$, x=10 − 28, y=7 − 8**	–	–	–	✓	✓	✓	✓	✓	✓
	**$\{Na_xK_yH_{104-x-y}W_{72}V_{30}S_{10}O_{363}\}^{10-}$, x=2 − 21, y=4 − 8**	–	–	–	✓	–	–	–	–	–
**-10**	**$\{Na_xK_yH_{98-x-y}W_{72}V_{30}S_{11}O_{363}\}^{10-}$, x=3 − 28, y=2 − 8**	–	–	–	✓	✓	✓	✓	✓	✓
	**$\{Na_xK_yH_{96-x}W_{72}V_{30}S_{12}O_{365}\}^{10-}$, x=0 – 28, y=1 – 8**	–	–	–	✓	✓	✓	✓	✓	✓
**-9**	**$\{Na_xK_yH_{109-x-y}W_{72}V_{30}S_{10}O_{365}\}^{9-}$, x=10 − 24, y=7 − 8**	–	–	–	✓	–	–	–	–	–

	**$\{Na_xK_yH_{105-x-y}W_{72}V_{30}S_{10}O_{363}\}^{9-}$, x=6 − 28, y=4 − 8**	–	–	–	✓	✓	✓	✓	✓	–
	**$\{Na_xK_yH_{103-x-y}W_{72}V_{30}S_{11}O_{365}\}^{9-}$, x=2 − 28, y=2 − 8**	–	–	–	✓	✓	✓	✓	✓	–
	**$\{Na_xK_yH_{99-x-y}W_{72}V_{30}S_{11}O_{363}\}^{9-}$, x=0 − 6, y=5 − 8**	–	–	–	✓	–	–	–	–	–
	**$\{Na_xK_yH_{97-x-y}W_{72}V_{30}S_{12}O_{365}\}^{9-}$, x=0 − 28, y=2 − 8**	–	–	–	✓	✓	✓	✓	✓	–
**-8**	**$\{Na_xK_yH_{110-x-y}W_{72}V_{30}S_{10}O_{365}\}^{8-}$, x=10 − 17, y= 7 − 8**	–	–	–	✓	–	–	–	–	–
	**$\{Na_xK_yH_{106-x-y}W_{72}V_{30}S_{10}O_{363}\}^{8-}$, x=6 − 28, y=3 − 7**	–	–	–	✓	✓	✓	–	–	–
	**$\{Na_xK_yH_{104-x-y}W_{72}V_{30}S_{11}O_{365}\}^{8-}$, x=2 − 28, y= 2 − 8**	–	–	–	✓	✓	✓	–	–	–
	**$\{Na_xK_yH_{100-x-y}W_{72}V_{30}S_{12}O_{363}\}^{8-}$, x=5 − 28, y=2 – 8**	–	–	–	✓	✓	✓	–	–	–
	**$\{Na_xK_yH_{98-x-y}W_{72}V_{30}S_{12}O_{365}\}^{8-}$, x=4 − 28, y=0 − 8**	–	–	–	✓	✓	✓	–	–	–

*visible in LC-ESI-MS spectra in acidified H_2O with pH 1 – 8; similar to ESI-MS spectra in acidified H_2O with pH 3.

Table S31. Buffer fingerprints: ligand strength, speciation bias, and characteristic decomposition products.

				Buffer specific mixed Mo/W-V products
(a) sodium phosphate	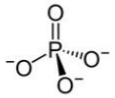	pK_a = 2.15, 6.86, 12.32	multidentate, oxophilic; heteroion template for POMs (e.g. $[P_2Mo_5O_{23}]^{6-}$)	**Mo:** $[P_2Mo_5O_{23}]^{6-}$, $[V^{V}Mo^{VI}O_{17}]^{5-}$, *β*-$[V^{V}Mo^{VI}_7O_{26}]^{5-}$, *α*-$[V^{V}_2Mo^{VI}_{18}O_{62}]^{6-}$<br>**W:** Lindqvist-type $[V^{V}W^{VI}_5O_{19}]^{3-}$ and *cis*-$[V^{V}_2W^{VI}_4O_{19}]^{4-}$
(b) acetic acid – sodium acetate	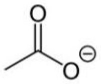	pK_a = 4.76	weak, bidentate O-donor	**Mo:** decavanadates $[V^{V}_{10}O_{28}]^{6-}$ and $[V^{V}_9Mo^{VI}O_{28}]^{5-}$, Lindqvist-type $[V^{V}Mo^{VI}_5O_{19}]^{3-}$ and $[V^{V}_2Mo^{VI}_4O_{19}]^{4-}$, *α*-$[V^{V}Mo^{VI}_7O_{26}]^{5-}$, *α*-$[V^{V}_2Mo^{VI}_{18}O_{62}]^{6-}$<br>**W:** Lindqvist-type $[V^{V}W^{VI}_5O_{19}]^{3-}$ and *cis*-, *trans*-$[V^{V}_2W^{VI}_4O_{19}]^{4-}$
(c) TRIS-HCl	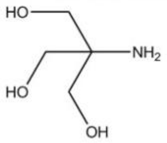	pK_a = 8.06	N/O-donor, chelator	**Mo:** Wough-type $[HV^{V}Mo^{VI}_9O_{32}]^{4-}$ and $[HV^{V}_2Mo^{VI}_{10}O_{38}]^{5-}$, *β*-$[V^{V}Mo^{VI}_7O_{26}]^{5-}$, $[V^{V}_9Mo^{VI}O_{28}]^{5-}$<br>**W:** Keggin-type $[H_2V^{V}W^{VI}_{11}O_{40}]^{3-}$, Lindqvist-type $[V^{V}W^{VI}_5O_{19}]^{3-}$ and *cis*-, $[V^{V}_2W^{VI}_4O_{19}]^{4-}$
(d) HEPES	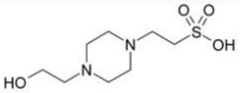	pK_a = 7.48	zwitterionic, bulky, poor chelator	only small vanadates V_1-V_5 detected by ^{51}V NMR

Abbreviations:

APC − all-phenyl complex
APTMS − 3-aminopropyltrimethoxysilane
ATR – Attenuated Total Reflection
aq – aqueous solution
BG − bioactive glass
BMSCs − rat primary bone marrow mesenchymal stem cells
bpy − 2,2′-bipyridine
BT – benzothiophene
BVS – Bond Valence Sum
cat. − catalyst
CC − carbon cloth
CD – circular dichroism
CDC − β-cyclodextrin (CD) grafting a pyridinium cation
CD − β-cyclodextrin
CEES – 2-chloroethyl ethyl sulfide
CFL − compact fluorescent lamp
CNTs − carbon nanotubes
cTnI − cardiac troponin I
CV – cyclic voltammetry
CVD − chemical vapor deposition
Cys − cysteine
DBT − dibenzothiophene
DEC − diethyl carbonate
DFNT − dendritic fibrous nanotitanium
DFNS − dendritic fibrous nanosilica
DI H_2O − deionized water
DLS − dynamic light scattering
DMC − dimethyl carbonate
DMSO− dimethyl sulfoxide
DMF – dimethylformamide
DODA− dimethyl-dioctadecylammonium
DODMA Br − dimethyldistearylammonium bromide
DOX − doxorubicin
DSC − differential scanning calorimetry
DTAB − dodecyltrimethylammonium bromide
Dye N719 − di-tetrabutylammonium cis-bis(isothiocyanato)bis(2,2'-bipyridyl-4,4'-dicarboxylato)ruthenium(II)
EC − ethylene carbonate
EEG − electrochemically exfoliated graphene nanosheets
EMI − 1-ethyl-3-methylimidazolium
EtOH − ethanol
ErChB − Eriochrome black T
ESI–MS – Electrospray Ionization Mass Spectrometry
FAAs − free fatty acids
FEC − fluoroethylene carbonate
FL − fluorescin

FTIR − Fourier transform infrared spectroscopy
HBSS-HEPES− Hank's Balanced Salt Solution with 4-(2-hydroxyethyl)-1-piperazineethanesulfonic acid
HBSS-MES − Hank's Balanced Salt Solution with 2-(N-morpholino)ethanesulfonic acid
HEPES – 4-(2-hydroxyethyl)-1-piperazineethanesulfonic acid
HMF − hydroxyfurfural
IL − ionic liquid
ITO − Indium tin oxide
IR – infrared spectroscopy
IVCT – Intervalence Charge Transfer
LB − Langmuir Blodgett Films
LB medium − Lysogeny broth medium
LIBs − lithium-ion batteries
MBs − molybdenum blues
MeCN − acetonitrile
Melem − 2,5,8-triamino-tri-s-triazine
MimAm – 3-aminoethyl-1-methylimidazolium
MIL-100 (Fe) − mesoporous iron carboxylate (III) MIL-100 metal-organic framework (iron(III) benzene-1,3,5-tricarboxylate)
MRBs − Mg-rechargeable batteries
MSNP − magnetic silica nanoparticles (Fe_3O_4@SiO_2 nanoparticles)
MSNP/APTES − amine-functionalized MSNP
MTN – metronidazole
NCM11-A – Ni-Co-Mn polycrystalline compound
NHPI – *N*-hydroxyphthalimide
NIR – near infra-red light
NMP – 1-methyl-2-pyrrolidinone
NMR – Nuclear Magnetic Resonance
PAAm – polyacrylamide
PAH – poly(allylamine hydrochloride)
PBS – Phosphate-buffered saline
PCL – ε-caprolactone
PDMS–NH_2 – amine-terminated polydimethylsiloxane
PEG – poly(ethylene glycol)
PEI – poly(ethylene-imine)
PEO – polyethylene oxide
PEO-b-PCL − poly(ethylene oxide)-block-poly(ε-caprolactone)
PhMgCl – phenylmagnesium chloride
PMo_{12} – $H_xPMo_{12}O_{40}$
POM – Polyoxometalate
PSS − polystyrene sulfonate
PVDF – poly(vinylidene fluoride)
py – pyridine
rGO – reduced graphene oxide
RhB – rhodamine B
RT – room temperature
RR – resonance Raman
SLS – static light scattering

SOMs – soft-oxometalates
SXRD – Single-Crystal X-ray Diffraction
TBHP – *tert*-butyl hydroperoxide
TENG – triboelectric nanogenerator
TEM – transmission electron microscopy
THF – tetrahydrofuran
TMOS – tetramethylorthosilicate
Tris – tris(hydroxymethyl)aminomethane
UV-vis – Ultraviolet–Visible Spectroscopy
VLP – virus-like particles
ZIF-8 – zeolitic imidazolate framework

REFERENCES AND NOTES

1. M. T. Pope, *Heteropoly and Isopoly Oxometalates* (Springer-Verlag, 1983) (Softcover ISBN: 978-3-662-12006-4).

2. M. T. Pope, A. Müller, Polyoxometalate chemistry: An old field with new dimensions in several disciplines. *Angew. Chem. Int. Ed. Engl.* **30**, 34–48 (1991).

3. A. Müller, C. Serain, Soluble molybdenum blues-"des Pudels Kern". *Acc. Chem. Res.* **33**, 2–10 (2000).

4. A. Müller, B. Botar, S. K. Das, H. Bögge, M. Schmidtmann, A. Merca, On the complex hedgehog-shaped cluster species containing 368 Mo atoms: Simple preparation method, new spectral details and information about the unique formation. *Polyhedron* **23**, 2381–2385 (2004).

5. B. Krebs, Comment on molybdenum polyoxo clusters: From the `Blues' to the `Reds'. *Acta Crystallogr. C Struct. Chem.* **78**, 322–323 (2022).

6. A. Müller, S. Sarkar, S. Q. N. Shah, H. Bögge, M. Schmidtmann, S. Sarkar, P. Kögerler, B. Hauptfleisch, A. X. Trautwein, V. Schünemann, Archimedean synthesis and magic numbers: "Sizing" giant molybdenum-oxide-based molecular spheres of the Keplerate type. *Angew. Chem. Int. Ed. Engl.* **38**, 3238–3241 (1999).

7. B. Botar, P. Kögerler, C. L. Hill, $[\{(Mo)Mo_5O_{21}(H_2O)_3(SO_4)\}_{12}(VO)_{30}(H_2O)_{20}]^{36-}$: A molecular quantum spin icosidodecahedron. *Chem. Commun.* **25**, 3138–3140 (2005).

8. A. M. Todea, A. Merca, H. Bögge, J. van Slageren, M. Dressel, L. Engelhardt, M. Luban, T. Glaser, M. Henry, A. Müller, Extending the $\{(Mo)Mo_5\}_{12}M_{30}$ capsule Keplerate sequence: A $\{Cr_{30}\}$ cluster of S=3/2 metal centers with a $\{Na(H_2O)_{12}\}$ encapsulate. *Angew. Chem. Int. Ed. Engl.* **46**, 6106–6110 (2007).

9. A. Müller, E. Krickemeyer, H. Bögge, M. Schmidtmann, F. Peters, Organizational forms of matter: An inorganic super fullerene and keplerate based on molybdenum oxide. *Angew. Chem. Int. Ed. Engl.* **37**, 3359–3363 (1998).

10. S.-I. Noro, R. Tsunashima, Y. Kamiya, K. Uemura, H. Kita, L. Cronin, T. Akutagawa, T. Nakamura, Adsorption and catalytic properties of the inner nanospace of a gigantic ring-shaped polyoxometalate cluster. *Angew. Chem. Int. Ed. Engl.* **48**, 8703–8706 (2009).

11. S. Das, T. Balaraju, S. Barman, S. S. Sreejith, R. Pochamoni, S. Roy, A molecular CO_2 reduction catalyst based on giant polyoxometalate $\{Mo_{368}\}$. *Front. Chem.* **6**, 514 (2018).

12. A. A. Abdelrahman, M. A. Betiha, A. M. Rabie, H. S. Ahmed, M. F. Elshahat, Removal of refractory organo-sulfur compounds using an efficient and recyclable $\{Mo_{132}\}$ nanoball supported graphene oxide. *J. Mol. Liq.* **252**, 121–132 (2018).

13. S. Xu, Y. Wang, Y. Zhao, W. Chen, J. Wang, L. He, Z. Su, E. Wang, Z. Kang, Keplerate-type polyoxometalate/semiconductor composite electrodes with light-enhanced conductivity towards highly efficient photoelectronic devices. *J. Mater. Chem. A* **4**, 14025–14032 (2016).

14. S. Das, A. Misra, S. Roy, Light driven decarboxylative cross coupling of acrylic acid and iodobenzene using $[Mo_{132}]$ type Keplerate as a catalyst. *Inorg. Chim. Acta* **460**, 77–82 (2017).

15. J. Yin, C. Huang, Y. Zhou, L. Zhang, N. Li, R. Sun, Selective oxidation of 2-chloroethyl ethyl sulfide in aqueous media catalyzed by $\{Mo_{72}M_{30}\}$ nano-polyoxometalate clusters differentiating the catalytic activity of nodal metals. *Ind. Eng. Chem. Res.* **61**, 7699–7708 (2022).

16. M. Gong, X.-Y. Wang, M.-Q. Li, W.-X. Mu, Y.-D. Cao, H. Liu, H. Y.-G. Lv, X.-H. Qi, G.-G. Gao, High-efficient and recoverable $Mo_{72}V_{30}$@Fe_3O_4/C catalyst for oxidation of hydroxyfurfural. *Fuel* **332**, 126050 (2023).

17. G. Markiewicz, D. Pakulski, A. Galanti, V. Patroniak, A. Ciesielski, A. R. Stefankiewicz, P. Samorì, Photoisomerisation and light-induced morphological switching of a polyoxometalate–azobenzene hybrid. *Chem. Commun.* **53**, 7278–7281 (2017).

18. A. Khoshyan, M. Pourtahmasb, F. Feizpour, M. Jafarpour, A. Rezaeifard, Aerobic $\{Mo_{72}V_{30}\}$ nanocluster-catalysed heterogeneous one-pot tandem synthesis of benzimidazoles. *Appl. Organometal. Chem.* **33**, e4638 (2019).

19. Z. Garazhian, A. Rezaeifard, M. Jafarpour, A nanoscopic icosahedral {$Mo_{72}Fe_{30}$} cluster catalyzes the aerobic synthesis of benzimidazoles. *RSC Adv.* **9**, 34854–34861 (2019).

20. S. Das, S. Biswas, T. Balaraju, S. Barman, R. Pochamoni, S. Roy, Photochemical reduction of carbon dioxide coupled with water oxidation using various soft-oxometalate (SOM) based catalytic systems. *J. Mater. Chem. A* **4**, 8875–8887 (2016).

21. Y. Wang, X. Kong, F. Li, B. Li, L. Wu, K. Chen, Y. Wu, Mo_{154} synergistically enhanced antibiofilm and antibacterial effects of spermine via coassembly. *ACS Appl. Bio Mater.* **5**, 5281–5288 (2022).

22. S. Zhang, H. Chen, G. Zhang, X. Kong, S. Yin, B. Li, L. Wu, An ultra-small thermosensitive nanocomposite with a Mo_{154}-core as a comprehensive platform for NIR-triggered photothermal-chemotherapy. *J. Mater. Chem. B* **6**, 241–248 (2018).

23. Y.-R. Xue, Y. Wang, G. Chen, B. Sun, B. Li, L. Wu, Y. Wu, A hybrid HPV capsid protein L_1 with giant Mo-containing polyoxometalate improves the stability of virus-like particles and the anti-tumor effect of [Mo_{154}]. *Biomater. Sci.* **9**, 3875–3883 (2021).

24. Y. Wang, G. Chen, R. Liu, X. Fang, F. Li, L. Wu, Y. Wu, Synergistically enhanced photothermal transition of a polyoxometalate/peptide assembly improved the antibiofilm and antibacterial activities. *Soft Matter* **18**, 2951–2958 (2022).

25. C. Falaise, S. Khlifi, P. Bauduin, P. Schmid, W. Shepard, A. A. Ivanov, M. N. Sokolov, M. A. Shestopalov, P. A. Abramov, S. Cordier, J. Marrot, M. Haouas, E. Cadot, “Host in Host” supramolecular core-shell type systems based on giant ring-shaped polyoxometalates. *Angew. Chem. Int. Ed. Engl.* **60**, 14146–14153 (2021).

26. B. Jing, M. Hutin, E. Connor, L. Cronin, Y. Zhu, Polyoxometalate macroion induced phase and morphology instability of lipid membrane. *Chem. Sci.* **4**, 3818–3826 (2013).

27. A. Tsuda, E. Hirahara, Y.-S. Kim, H. Tanaka, T. Kawai, T. Aida, A molybdenum crown cluster forms discrete inorganic–organic nanocomposites with metalloporphyrins. *Angew. Chem. Int. Ed. Engl.* **43**, 6327–6331 (2004).

28. I. M. Mbomekallé, F. Bian, H. Tebba, I. A. Weinstock, Electrocatalytic reduction of O_2 by a Cu(II)-substituted electron-rich wheel-type oxomolybdate nanocluster. *J. Clust. Sci.* **17**, 333–348 (2006).

29. X. Fan, S. Garai, R. R. Gaddam, P. V. Menezes, D. P. Dubal, Y. Yamauchi, P. W. Menezes, A. K. Nanjundan, X. S. Zhao, Uncovering giant nanowheels for magnesium ion–based batteries. *Mater. Today Chem.* **16**, 100221 (2020).

30. S. Polarz, B. Smarsly, C. Göltner, M. Antonietti, The interplay of colloidal organization and oxo-cluster chemistry: Polyoxometalate–silica hybrids—Materials with a nanochemical function. *Adv. Mater.* **12**, 1503–1507 (2000).

31. M. Clemente-León, T. Ito, H. Yashiro, T. Yamase, E. Coronado, Langmuir−Blodgett films of a Mo-Blue nanoring $[Mo_{142}O_{429}H_{10}(H_2O)_{49}(CH_3CO_2)_5(CH_3CH_2CO_2)]^{30-}$ (Mo_{142}) by the semiamphiphilic method. *Langmuir* **23**, 4042–4047 (2007).

32. P. Wang, T. Wang, M. Xu, Z. Gao, H. Li, B. Li, Y. Wang, C. Qu, M. Feng, Keplerate polyoxomolybdate nanoball mediated controllable preparation of metal-doped molybdenum disulfide for electrocatalytic hydrogen evolution in acidic and alkaline media. *Chin. Chem. Lett.* **35**, 108930 (2024).

33. N. I. Gumerova, A. Rompel, Polyoxometalates in solution: Speciation under spotlight. *Chem. Soc. Rev.* **49**, 7568–7601 (2020).

34. N. I. Gumerova, A. Rompel, Synthesis, structures and applications of electron-rich polyoxometalates. *Nat. Rev. Chem.* **2**, 0112 (2018).

35. T. Kiss, É. A. Enyedy, T. Jakusch, Development of the application of speciation in chemistry. *Coord. Chem. Rev.* **352**, 401–423 (2017).

36. A. Kot, J. Namieśnik, The role of speciation in analytical chemistry. *TrAC Trends Anal. Chem.* **19**, 69–79 (2000).

37. S. Shishido, T. Ozeki, The pH dependent nuclearity variation of {Mo_{154-x}}-type polyoxomolybdates and tectonic effect on their aggregations. *J. Am. Chem. Soc.* **130**, 10588–10595 (2008).

38. M. L. Kistler, T. Liu, P. Gouzerh, A. M. Todea, A. Müller, Molybdenum-oxide based unique polyprotic nanoacids showing different deprotonations and related assembly processes in solution. *Dalton Trans.* **26**, 5094–5100 (2009).

39. N. I. Gumerova, A. Rompel, Speciation atlas of polyoxometalates in aqueous solutions. *Sci. Adv.* **9**, eadi0814 (2023).

40. M. Ždrnja, N. I. Gumerova, A. Rompel, Exploring polyoxometalate speciation: The interplay of concentration, ionic strength, and buffer composition. *Front. Chem. Biol.* **3**, 1444359 (2024).

41. J. F. Keggin, Structure of the molecule of 12-phosphotungstic acid. *Nature* **131**, 908–909 (1933).

42. C. R. Graham, R. G. Finke, The classic Wells−Dawson polyoxometalate, $K_6[\alpha\text{-}P_2W_{18}O_{62}]\cdot 14H_2O$. Answering an 88 year-old question: What is its preferred, optimum synthesis? *Inorg. Chem.* **47**, 3679–3686 (2008).

43. A. Blazevic, A. Rompel, The Anderson-Evans polyoxometalate: From inorganic building blocks via hybrid organic-inorganic structures to tomorrows “Bio-POM”. *Coord. Chem. Rev.* **307**, 42–64 (2016).

44. L. Chen, K. A. San, M. J. Turo, M. Gembicky, S. Fereidouni, M. Kalaj, A. M. Schimpf, Tunable metal oxide frameworks via coordination assembly of Preyssler-type molecular clusters. *J. Am. Chem. Soc.* **141**, 20261–20268 (2019).

45. M. Aureliano, D. C. Crans, Decavanadate ($V_{10}O_{28}^{6-}$) and oxovanadates: Oxometalates with many biological activities. *J. Inorg. Biochem.* **103**, 536–546 (2009).

46. M. Haouas, J. Trébosc, C. Roch-Marchal, E. Cadot, F. Taulelle, C. Martineau-Corcos, High-field ^{95}Mo and ^{183}W static and MAS NMR study of polyoxometalates. *Magn. Reson. Chem.* **55**, 902–908 (2017).

47. L. P. Kazansky, T. Yamase, Electronic population on tungsten, molybdenum, and vanadium atoms and ^{183}W, ^{95}Mo, and ^{51}V NMR in polyoxometalates. *Phys. Chem. A* **108**, 6437–6448 (2004).

48. B. Botar, A. Ellern, P. Kögerler, Mapping the formation areas of giant molybdenum blue clusters: A spectroscopic study. *Dalton Trans.* **41**, 8951–8959 (2012).

49. E. Al-Sayed, A. Rompel, Lanthanides singing the blues: Their fascinating role in the assembly of gigantic molybdenum blue wheels. *ACS Nanosci. Au* **2**, 179–197 (2022).

50. D.-L. Long, C. Streb, Y.-F. Song, S. Mitchell, L. Cronin, Unravelling the complexities of polyoxometalates in solution using mass spectrometry: Protonation versus heteroatom inclusion. *J. Am. Chem. Soc.* **130**, 1830–1832 (2008).

51. I. Nakamura, H. N. Miras, A. Fujiwara, M. Fujibayashi, Y.-F. Song, L. Cronin, R. Tsunashima, Investigating the formation of “molybdenum blues” with gel electrophoresis and mass spectrometry. *J. Am. Chem. Soc.* **137**, 6524–6530 (2015).

52. A. M. Todea, A. Merca, H. Bögge, T. Glaser, L. Engelhardt, R. Prozorov, M. Luban, A. Müller, Polyoxotungstates now also with pentagonal units: Supramolecular chemistry and tuning of magnetic exchange in $\{(M)M_5\}_{12}V_{30}$ keplerates (M = Mo, W). *Chem. Commun.* **23**, 3351–3353 (2009).

53. Y. Li, Y.-G. Li, Z.-M. Zhang, Q. Wu, E.-B. Wang, A new polyoxotungstate-based $\{W_{72}V_{30}\}$ spherical cage. *Inorg. Chem. Commun.* **12**, 864–867 (2009).

54. D. Render, A survey of ^{51}V NMR spectroscopy. *Bull. Magn. Reson.* **4**, 33–83 (1982).

55. A. A. Shubin, D. F. Khabibulin, O. B. Lapina, Solid-state ^{51}V NMR and its potentiality in investigation of vanadia systems with paramagnetic centres. *Catal. Today* **142**, 220–226 (2009).

56. A. T. Iavarone, O. A. Udekwu, E. R. Williams, Buffer loading for counteracting metal salt-induced signal suppression in electrospray ionization. *Anal. Chem.* **76**, 3944–3950 (2004).

57. A. A. Ostroushko, M. O. Tonkushina, V. Y. Korotaev, A. V. Prokof'eva, I. B. Kutyashev, V. A. Vazhenin, I. G. Danilova, S. Y. Men'shikov, Stability of the $Mo_{72}Fe_{30}$ polyoxometalate buckyball in solution. *Russ. J. Inorg. Chem.* **57**, 1210–1213 (2012).

58. T. Liu, B. Imber, E. Diemann, G. Liu, K. Cokleski, H. Li, Z. Chen, A. Müller, Deprotonations and charges of well-defined $\{Mo_{72}Fe_{30}\}$ nanoacids simply stepwise tuned by ph allow control/variation of related self-assembly processes. *J. Am. Chem. Soc.* **128**, 15914–15920 (2006).

59. T. Matsumoto, I. Nakamura, K. Ishiguro, R. Tsunashima, Concentration dependent stability of fullerene-shaped metal-oxide nanocluster $\{Mo_{132}\}$ in aqueous solution. *Sci. Adv. Mater.* **6**, 1389–1393 (2014).

60. T. Liu, Supramolecular structures of polyoxomolybdate-based giant molecules in aqueous solution. *J. Am. Chem. Soc.* **124**, 10942–10943 (2002).

61. A. A. Ostroushko, M. O. Tonkushina, A. P. Safronov, New data for molybdenum polyoxometallate with the buckyball structure containing acetate groups and compositions based thereon. *Russ. J. Inorg. Chem.* **55**, 808–813 (2010).

62. M. L. Kistler, A. Bhatt, G. Liu, D. Casa, T. Liu, A complete macroion−"Blackberry" assembly−Macroion transition with continuously adjustable assembly sizes in $\{Mo_{132}\}$ water/acetone systems. *J. Am. Chem. Soc.* **129**, 6453–6460 (2007).

63. R. Mekala, S. Supriya, S. K. Das, Fate of a giant $\{Mo_{72}Fe_{30}\}$-type polyoxometalate cluster in an aqueous solution at higher temperature: Understanding related Keplerate chemistry, from molecule to material. *Inorg. Chem.* **52**, 9708–9710 (2013).

64. V. Lakhanpal, M. Guillén-Soler, L. Vilà-Nadal, D.-L. Long, L. Cronin, Compression of molybdenum blue polyoxometalate cluster rings. *J. Am. Chem. Soc.* **147**, 10579–10586 (2025).

65. A. Müller, S. Q. N. Shah, H. Bögge, M. Schmidtmann, P. Kögerler, B. Hauptfleisch, S. Leiding, K. Wittler, Thirty electrons "Trapped" in a spherical matrix: A molybdenum oxide-based nanostructured Keplerate reduced by 36 electrons. *Angew. Chem. Int. Ed. Engl.* **39**, 1614–1616 (2000).

66. P. Yin, D. Li, T. Liu, Solution behaviors and self-assembly of polyoxometalates as models of macroions and amphiphilic polyoxometalate–organic hybrids as novel surfactants. *Chem. Soc. Rev.* **41**, 7368–7383 (2012).

67. W. Yang, C. Lu, X. Lin, S. Wang, H. Zhuang, A new defective derivative of a ring-shaped nanosize polyoxomolybdate: Synthesis and structure of $Na_{28}[Mo^{VI}_{112}Mo^{V}_{28}O_{427}H_{14}(H_2O)_{56}]\cdot$ca. $300H_2O$. *Inorg. Chem. Commun.* **4**, 245–247 (2001).

68. A. Müller, C. Beugholt, M. Koop, S. K. Das, M. Schmidtmann, H. Bögge, Facile and optimized syntheses and structures of crystalline molybdenum blue compounds including one with an interesting high degree of defects: $Na_{26}[Mo_{142}O_{432}(H_2O)_{58}H_{14}]\cdot$ca. $300\,H_2O$ and $Na_{16}[(MoO_3)_{176}(H_2O)_{63}(CH_3OH)_{17}H_{16}]\cdot$ca. $600\,H_2O\cdot$ca. $6\,CH_3OH$. *Z. anorg. allg. Chem.* **625**, 1960–1962 (1999).

69. A. Müller, E. Krickemeyer, H. Bögge, M. Schmidtmann, F. Peters, C. Menke, J. Meyer, An unusual polyoxomolybdate: Giant wheels linked to chains. *Angew. Chem. Int. Ed. Engl.* **36**, 484–486 (1997).

70. A. Müller, S. K. Das, V. P. Fedin, E. Krickemeyer, C. Beugholt, H. Bögge, M. Schmidtmann, B. Hauptfleisch, Rapid and simple isolation of the crystalline molybdenum-blue compounds with discrete and linked nanosized ring-shaped anions: $Na_{15}[Mo^{VI}_{126}Mo^{V}_{28}O_{462}H_{14}(H_2O)_{70}]_{0.5}$ $[Mo^{VI}_{124}Mo^{V}_{28}O_{457}H_{14}(H_2O)_{68}]_{0.5}\cdot$ca. $400\,H_2O$ and $Na_{22}[Mo^{VI}_{118}Mo^{V}_{28}O_{442}H_{14}(H_2O)_{58}]\cdot$ca. $250\,H_2O$. *Z. anorg. allg. Chem.* **625**, 1187–1192 (1999).

71. A. Müller, E. Krickemeyer, H. Bögge, M. Schmidtmann, C. Beugholt, S. K. Das, F. Peters, Giant ring-shaped building blocks linked to form a layered cluster network with nanosized channels: $[Mo_{124}{}^{VI}Mo_{28}{}^{V}O_{429}(\mu_3\text{-}O)_{28}H_{14}(H_2O)_{66.5}]^{16-}$. *Chem. A Eur. J.* **5**, 1496–1502 (1999).

72. A. Müller, E. Krickemeyer, J. Meyer, H. Bögge, F. Peters, W. Plass, E. Diemann, S. Dillinger, F. Nonnenbruch, M. Randerath, C. Menke, $[Mo_{154}(NO)_{14}O_{420}(OH)_{28}(H_2O)_{70}]^{(25 \pm 5)-}$: A water-soluble big wheel with more than 700 atoms and a relative molecular mass of about 24000. *Angew. Chem. Int. Ed. Engl.* **34**, 2122–2124 (1995).

73. W. Xuan, R. Pow, Q. Zheng, N. Watfa, D.-L. Long, L. Cronin, Ligand-directed template assembly for the construction of gigantic molybdenum blue wheels. *Angew. Chem. Int. Ed. Engl.* **58**, 10867–10872 (2019).

74. W. Xuan, R. Pow, N. Watfa, Q. Zheng, A. J. Surman, D.-L. Long, L. Cronin, Stereoselective assembly of gigantic chiral molybdenum blue wheels using lanthanide ions and amino acids. *J. Am. Chem. Soc.* **141**, 1242–1250 (2019).

75. A. Müller, S. K. Das, C. Kuhlmann, H. Bögge, M. Schmidtmann, E. Diemann, E. Krickemeyer, J. Hormes, H. Modrow, M. Schindler, On the option of generating novel type surfaces with multiphilic ligands within the cavity of a giant metal–oxide based wheel type cluster: Chemical reactions with well-defined nanoobjects. *Chem. Commun.* **7**, 655–656 (2001).

76. L. Zhang, Y. Li, Y. Zhou, Surface modification with multiphilic ligands at detectable well defined active positions of nano-object of giant wheel shaped molybdenum blue showing third-order nonlinear optical properties. *J. Mol. Struct.* **969**, 69–74 (2010).

77. C.-C. Jiang, Y.-G. Wei, Q. Liu, S.-W. Zhang, M.-C. Shao, Y.-Q. Tang, Self-assembly of a novel nanoscale giant cluster: $[Mo_{176}O_{496}(OH)_{32}(H_2O)_{80}]$. *Chem. Commun.* **18**, 1937–1938 (1998). DOI: 10.25365/phaidra.694.

78. W. Xuan, R. Pow, D.-L. Long, L. Cronin, Exploring the molecular growth of two gigantic half-closed polyoxometalate clusters $\{Mo_{180}\}$ and $\{Mo_{130}Ce_6\}$. *Angew. Chem. Int. Ed.* **56**, 9727 (2017).

79. H. M. Miras, C. J. Richmond, D.-L. Long, L. Cronin, Solution-phase monitoring of the structural evolution of a molybdenum blue nanoring. *J. Am. Chem. Soc.* **134**, 3816–3824 (2012).

80. J. Lin, N. Li, S. Yang, M. Jia, J. Liu, X.-M. Li, L. An, Q. Tian, L.-Z. Dong, Y.-Q. Lan, Self-assembly of giant Mo_{240} hollow opening dodecahedra. *J. Am. Chem. Soc.* **142**, 13982–13988 (2020).

81. A. Müller, S. Q. N. Shah, H. Bögge, M. Schmidtmann, Molecular growth from a Mo_{176} to a Mo_{248} cluster. *Nature* **397**, 48–50 (1999).

82. A. Müller, E. Beckmann, H. Bögge, M. Schmidtmann, A. Dress, Inorganic chemistry goes protein size: A Mo_{368} nano-hedgehog initiating nanochemistry by symmetry breaking. *Angew. Chem. Int. Ed. Engl.* **41**, 1162–1167 (2002).

83. A. Müller, E. Krickemeyer, S. Dillinger, H. Bögge, W. Plass, A. Proust, L. Dloczik, C. Menke, J. Meyer, R. Rohlfing, New perspectives in polyoxometalate chemistry by isolation of compounds containing very large moieties as transferable building blocks: $(NMe_4)_5[As_2Mo_8V_4AsO_{40}]\cdot 3H_2O$, $(NH_4)_{21}[H_3Mo_{57}V_6(NO)_6O_{183}(H_2O)_{18}]\cdot 65\ H_2O$, $(NH_2Me_2)_{18}(NH_4)_6[Mo_{57}V_6(NO)_6O_{183}(H_2O)_{18}]\cdot 14\ H_2O$, and $(NH_4)_{12}[Mo_{36}(NO)_4O_{108}(H_2O)_{16}]\cdot 33\ H_2O$. *Z. anorg. allg. Chem.* **620**, 599–619 (1994).

84. A. Müller, J. Meyer, E. Krickemeyer, C. Beugholt, H. Bögge, F. Peters, M. Schmidtmann, P. Kögerler, M. J. Koop, Unusual stepwise assembly and molecular growth: $[H_{14}Mo_{37}O_{112}]^{14-}$ and $[H_3Mo_{57}V_6(NO)_6O_{189}(H_2O)_{12}(MoO)_6]^{21-}$. *Chem. Eur. J.* **4**, 1000–1006 (1998).

85. A. Nicoara, A. Patrut, D. Margineanu, A. Müller, Electrochemical investigation of molecular growth of the $\{Mo_{57}V_6\}$ polyoxometalate cluster. *Electrochem. Commun.* **5**, 511–518 (2003).

86. W. Yang, X. Lin, C. Lu, H. Zhuang, J. Huang, $(NH_4)_{15}[H_3Mo_{57}V_6(NO)_6O_{189}(H_2O)_{12}(VO)_6]\cdot$□6 0$H_2O$: new nanocompound obtained by chemical embellishment of $\{M_{57}V_6\}$. *Inorg. Chem.* **39**, 2706–2707 (2000).

87. A. Müller, M. Koop, H. Bögge, M. Schmidtmann, F. Peters, P. Kögerler, Building blocks as a disposition in solution: $[\{Mo^{VI}O_3(H_2O)\}_{10}\{V^{IV}O(H_2O)\}_{20}\{(Mo^{VI}/Mo^{VI}{}_5O_{21})(H_2O)_3\}_{10}(\{Mo^{VI}O_2(H_2O)_2\}_{5/2})_2(\{NaSO_4\}_5)_2]^{20-}$, a giant spherical cluster with unusual structural features of interest for supramolecular. *Chem. Commun.* **1999**, 1885–1886 (1999).

88. A. Müller, B. Botar, H. Bögge, P. Kögerler, A. Berkle, A potassium selective 'nanosponge' with well defined pores. *Chem. Commun.* **24**, 2944–2945 (2002).

89. B. Botar, P. Kögerler, C. L. Hill, A nanoring–nanosphere molecule, $\{Mo_{214}V_{30}\}$: Pushing the boundaries of controllable inorganic structural organization at the molecular level. *J. Am. Chem. Soc.* **128**, 5336–5337 (2006).

90. A. Müller, W. Plass, E. Krickemeyer, S. Dillinger, H. Bögge, A. Armatage, A. Proust, C. Beugholt, U. Bergmann, $[Mo_{57}Fe_6(NO)_6O_{174}(OH)_3(H_2O)_{24}]^{15-}$: A highly symmetrical giant cluster with an unusual cavity and the possibility of positioning paramagnetic centers on extremely large cluster surfaces. *Angew. Chem. Int. Ed. Engl.* **33**, 849–851 (1994).

91. J. Fielden, Y. L. Malaestean, A. Ellern, R. Garcia-Serres, L. Cronin, P. Kögerler, Inducing molecular growth in an $\{Mo_{57}Fe_6\}$-type nanocluster: Synthesis, structure, and properties of $\{Mo_{57}(Mo)_2Fe^{III}{}_6\}$. *J. Clust. Sci.* **17**, 291–302 (2006).

92. A. Müller, S. K. Das, M. O. Talismanova, H. Bögge, P. Kögerler, M. Schmidtmann, S. S. Talismanov, M. Luban, E. Krickemeyer, Paramagnetic keplerate "necklaces" synthesized by a novel room-temperature solid-state reaction: Controlled linking of metal-oxide-based nanoparticles. *Angew. Chem. Int. Ed. Engl.* **41**, 579–582 (2002).

93. Z.-H. Xu, Y.-G. Li, E.-B. Wang, C. Qin, X.-L. Wang, Synthesis, characterization, and crystal structures of two new polyoxomolybdate wheel clusters. *Inorg. Chem. Commun.* **9**, 1315–1318 (2006).

94. A. Müller, E. Krickemeyer, H. Bögge, M. Schmidtmann, P. Kögerler, C. Rosu, E. Beckmann, "Nanoobjects" by self-assembly concomitant with modifications under alterable boundary conditions: Incorporation of paramagnetic metal centers (Cu^{2+}) in ring-shaped molybdenum-oxide based clusters. *Angew. Chem. Int. Ed. Engl.* **113**, 4158–4161 (2001).

95. T. Yamase, E. Ishikawa, Y. Abe, Y. Yano, Photoinduced self-assembly to lanthanide-containing molybdenum-blue superclusters and molecular design. *J. Alloys Compd.* **408-412**, 693–700 (2006).

96. R. Garrido Ribó, N. L. Bell, W. Xuan, J. Luo, D.-L. Long, T. Liu, L. Cronin, Synthesis, assembly, and sizing of neutral, lanthanide substituted molybdenum blue wheels $\{Mo_{90}Ln_{10}\}$. *J. Am. Chem. Soc.* **142**, 17508–17514 (2020).

97. T. Yamase, S. Kumagai, P. V. Prokop, E. Ishikawa, A.-R. Tomsa, $\{Mo_{96}La_8\}$ eggshell ring and self-assembly to $\{Mo_{132}\}$ keplerate through Mo-blue intermediate, involved in UV-photolysis of $[Mo_7O_{24}]^{6-}$/carboxylic acid system at pH 4. *Inorg. Chem.* **49**, 9426–9437 (2010).

98. E. Ishikawa, Y. Yano, T. Yamase, Coordination of $\{Mo_{142}\}$ ring to La^{3+} provides elliptical $\{Mo_{134}La_{10}\}$ ring with a variety of coordination modes. *Materials (Basel)* **3**, 64–75 (2010).

99. X.-X. Li, C.-H. Li, M.-J. Hou, B. Zhu, W.-C. Chen, C.-Y. Sun, Y. Yuan, W. Guan, C. Qin, K.-Z. Shao, X.-L. Wang, Z.-M. Su, Ce-mediated molecular tailoring on gigantic polyoxometalate $\{Mo_{132}\}$ into half-closed $\{Ce_{11}Mo_{96}\}$ for high proton conduction. *Nat. Commun.* **14**, 5025 (2023).

100. W. Xuan, A. J. Surman, H. N. Miras, D.-L. Long, L. Cronin, Controlling the ring curvature, solution assembly, and reactivity of gigantic molybdenum blue wheels. *J. Am. Chem. Soc.* **136**, 14114–14120 (2014).

101. V. Duros, J. Grizou, W. Xuan, Z. Hosni, D.-L. Long, H. N. Miras, L. Cronin, Human versus robots in the discovery and crystallization of gigantic polyoxometalates. *Angew. Chem. Int. Ed. Engl.* **56**, 10815–10820 (2017).

102. E. Al-Sayed, E. Tanuhadi, G. Giester, A. Rompel, Synthesis and characterization of the `Japanese rice-ball'-shaped molybdenum blue $Na_4[Mo_2O_2(OH)_4(C_6H_4NO_2)_2]_2[Mo_{120}Ce_6O_{366}H_{12}(OH)_2(H_2O)_{76}]$□$200H_2O$. *Acta Crystallogr. Sect. C Struct. Chem.* **78**, 299–304 (2022).

103. S. She, W. Xuan, N. L. Bell, R. Pow, E. G. Ribo, Z. Sinclair, D.-L. Long, L. Cronin, Peptide sequence mediated self-assembly of molybdenum blue nanowheel superstructures. *Chem. Sci.* **12**, 2427–2432 (2021).

104. A. Müller, C. Beugholt, H. Bögge, M. Schmidtmann, Influencing the size of giant rings by manipulating their curvatures: $Na_6[Mo_{120}O_{366}(H_2O)_{48}H_{12}\{Pr(H_2O)_5\}_6]\cdot$(□200$H_2O$) with open shell metal centers at the cluster surface. *Inorg. Chem.* **39**, 3112–3113 (2000).

105. L. Cronin, C. Beugholt, E. Krickemeyer, M. Schmidtmann, H. Bögge, P. Kögerler, T. K. K. Luong, A. Müller, "Molecular symmetry breakers" generating metal-oxide-based nanoobject fragments as synthons for complex structures: $[\{Mo_{128}Eu_4O_{388}H_{10}(H_2O)_{81}\}_2]^{20-}$, a giant-cluster dimer. *Angew. Chem. Int. Ed. Engl.* **41**, 2805–2808 (2002).

106. Y.-L. Wu, J. Du, H.-Y. Zhang, M.-J. Hou, Q.-Y. Li, W.-C. Chen, K.-Z. Shao, B. Zhu, C. Qin, X.-L. Wang, Z.-M. Su, Dimensional regulation in gigantic molybdenum blue wheels featuring $\{(W)Mo_5\}$ motifs for enhanced proton conductivity. *Nano Res.* **17**, 8261–8268 (2024).

107. C. Schäffer, A. Merca, H. Bögge, A. M. Todea, M. L. Kistler, T. Liu, R. Thouvenot, P. Gouzerh, A. Müller, Unprecedented and differently applicable pentagonal units in a dynamic library: A Keplerate of the type $\{(W)W_5\}_{12}\{Mo_2\}_{30}$. *Angew. Chem. Int. Ed. Engl.* **48**, 149–153 (2009).

108. A. Todea, A. Merca, H. Bögge, T. Glaser, J. Pigga, M. L. K. Langston, T. Liu, R. Prozorov, M. Luban, C. Schröder, W. Casey, A. Müller, Porous capsules $\{(M)M_5\}_{12}Fe^{III}{}_{30}$ (M=Mo^{VI}, W^{VI}): Sphere surface supramolecular chemistry with 20 ammonium ions, related solution properties, and tuning of magnetic exchange interactions. *Angew. Chem. Int. Ed. Engl.* **49**, 514–519 (2010).

109. S. Das, D. Lai, A. Mallick, S. Roy, Photo redox mediated inexpensive one-pot synthesis of 1,4-diphenyl substituted butane-1,4-dione from styrene using polyoxometalate as a catalyst. *ChemistrySelect* **1**, 691–695 (2016).

110. A. M. Kermani, V. Mahmoodi, M. Ghahramaninezhad, A. Ahmadpour, Highly efficient and green catalyst of $\{Mo_{132}\}$ nanoballs supported on ionic liquid-functionalized magnetic silica nanoparticles for oxidative desulfurization of dibenzothiophene. *Sep. Purif. Technol.* **258**, 117960 (2021).

111. V. Mahmoudi, A. M. Kermani, M. Ghahramaninezhad, A. Ahmadpour, Oxidative desulfurization of dibenzothiophene by magnetically recoverable polyoxometalate-based nanocatalyst: Optimization by response surface methodology. *Mol. Catal.* **509**, 111611 (2021).

112. M. Ghahramaninezhad, F. Pakdel, M. N. Shahrak, Boosting oxidative desulfurization of model fuel by POM-grafting ZIF-8 as a novel and efficient catalyst. *Polyhedron* **170**, 364–372 (2019).

113. M. Gong, W.-X. Mu, Y.-D. Cao, Y.-W. Shao, X. Hu, D. Yin, H. Liu, C.-H. Zhang, G.-G. Gao, A giant polyoxomolybdate molecular catalyst with unusual Mo^{6+}/Mo^{5+} synergistic mechanism for oxidation of hydroxyfurfural under atmospheric pressure. *Fuel Process. Technol.* **242**, 107635 (2023).

114. C. Yang, W. Zhao, Z. Cheng, B. Luoa, D. Bia, Catalytic system for pyridine oxidation to N-oxides under mild conditions based on polyoxomolybdate. *RSC Adv.* **5**, 36809–36812 (2015).

115. A. Rezaeifard, M. Jafarpour, R. Haddad, H. Tavallaei, M. Hakimi, Clean and heterogeneous condensation of 1,2-diamines with 1,2-dicarbonyls catalyzed by $\{Mo_{132}\}$ giant ball nanocluster. *J. Clust. Sci.* **26**, 1439–1450 (2015).

116. A. Rezaeifard, R. Haddad, M. Jafarpour, M. Hakimi, $\{Mo_{132}\}$ nanoball as an efficient and cost-effective catalyst for sustainable oxidation of sulfides and olefins with hydrogen peroxide. *ACS Sustainable Chem. Eng.* **2**, 942–950 (2014).

117. M. Rohaniyan, A. Davoodnia, A. Nakhaei, Another application of $(NH_4)_{42}[Mo^{VI}_{72}Mo^{V}_{60}O_{372}(CH_3COO)_{30}(H_2O)_{72}]$ as a highly efficient recyclable catalyst for the synthesis of dihydropyrano[3,2-c]chromenes. *Appl. Organometal. Chem.* **30**, 626–629 (2016).

118. A. Davoodnia, A. Nakhaei, N. Tavakoli-Hoseini, Catalytic performance of a Keplerate-type, giant-ball nanoporous isopolyoxomolybdate as a highly efficient recyclable catalyst for the synthesis of biscoumarins. *Z. Naturforsch. B* **71**, 219–225 (2016).

119. Y. Zhou, L. Qin, C. Yu, T. Xiong, L. Zhang, W. Ahmad, H. Han, Towards applications in catalysis: Investigation on photocatalytic activities of a derivative family of the Keplerate type molybdenum–oxide based polyoxometalates. *RSC Adv.* **4**, 54928–54935 (2014).

120. W. Xie, X. Wang, L. Guo, Utilization of Keplerate-type polyoxomolybdates $\{Mo_{132}\}$ supported on hierarchical porous SOM-ZIF-8 as reusable catalyst boosts biodiesel production from acidic soybean oils by simultaneous transesterification-esterifications. *Renew. Energy* **225**, 120312 (2024).

121. Y. Chen, S. Mohsen Sadeghzadeh, Dendritic fibrous nano-titanium (DFNT) with highly dispersed poly(ionic liquids) as a nanocatalyst for synthesis of dimethyl carbonate from methanol and carbon dioxide. *J. Mol. Liq.* **384**, 122201 (2023).

122. H.-J. Lee, E. S. Cho, Selective photocatalytic C–C bond cleavage of lignin models and conversion to high-value chemical by polyoxometalate under a mild water-based environment. *ACS Sustainable Chem. Eng.* **11**, 7624–7632 (2023).

123. S. Chen, J. Li, R. Haddad, S. M. Sadeghzadeh, Cycloaddition of allylic chlorides, aryl alkynes, and carbon dioxide using nanoclusters of polyoxomolybdate buckyball supported by ionic liquid on dendritic fibrous nanosilica. *J. CO_2 Util.* **61**, 102035 (2022).

124. K.-R. Oh, Y. Han, G.-Y. Cha, A. H. Valekar, M. Lee, S. E. Sivan, Y.-U. Kwon, Y. K. Hwang, Carbonic anhydrase-mimicking Keplerate cluster encapsulated iron trimesate for base-free CO_2 hydrogenation. *ACS Sustainable Chem. Eng.* **9**, 14051–14060 (2021).

125. J. Liu, N. Jiang, J.-M. Lin, Z.-B. Mei, L.-Z. Dong, Y. Kuang, J.-J. Liu, S.-J. Yao, S.-L. Li, Y.-Q. Lan, Structural evolution of giant polyoxometalate: From “Keplerate” to “Kantern” type Mo_{132} for improved oxidation catalysis. *Angew. Chem. Int. Ed. Engl.* **62**, e202304728 (2023).

126. M. R. Farsani, B. Yadollahi, H. Taghiyar, A. J. Moghadam, Mo_{132}-ionic liquid as an effective hybrid catalyst for selective epoxidation of different alkenes with H_2O_2. *Catal. Lett.* **155**, 70 (2025).

127. S. Zafari, M. N. Shahrak, M. Ghahramaninezhad, New MOF-based corrosion inhibitor for carbon steel in acidic media. *Met. Mater. Int.* **26**, 25–38 (2020).

128. D. Pakulski, A. Gorczyński, W. Czepa, Z. Liu, L. Ortolani, V. Morandi, V. Patroniak, A. Ciesielski, P. Samorì, Novel Keplerate type polyoxometalate-surfactant-graphene hybrids as advanced electrode materials for supercapacitors. *Energy Storage Mater.* **17**, 186–193 (2019).

129. A. Denikaev, Y. Kuznetsova, A. Bykov, A. Zhilyakov, K. Belova, P. Abramov, N. Moskalenko, E. Skorb, K. Grzhegorzhevskii, Keplerate $\{Mo_{132}\}$–Stearic acid conjugates: Supramolecular synthons for the design of dye-loaded nanovesicles, Langmuir–Schaefer films, and infochemical applications. *ACS Appl. Mater. Interfaces* **16**, 7430–7443 (2024).

130. W. Du, Y. Hao, Y. He, Y. Chen, Y. Peng, W. Chen, Keplerate-type polyoxometalates-based triboelectric nanogenerator for higher performance via themorphology modulation of blackberry structure. *Chem. A Eur. J.* **30**, e202400882 (2024).

131. F. Ji, F. Jiang, H. Luo, W.-W. He, X. Han, W. Shen, M. Liu, T. Zhou, J. Xu, Z. Wang, Y.-Q. Lan, Hybrid membrane of sulfonated Poly(aryl ether ketone sulfone) modified by molybdenum clusters with enhanced proton conductivity. *Small* **20**, e2312209 (2024).

132. X. Xu, L. Zhang, T. Wang, Y. Li, T. Ji, W. Chen, C. Wang, C. Lind, The dual effect of "inorganic fullerene" $\{Mo_{132}\}$ doped with SnO_2 for efficient perovskite-based photodetectors. *Mater. Chem. Front.* **5**, 6931–6940 (2021). DOI: 10.1039/D1QM00618E.

133. Z. Shi, Y. Zhou, L. Zhang, D. Yang, C. Mu, H. Ren, F. K. Shehzada, J. Lia, Fabrication and optical nonlinearities of composite films derived from the water-soluble Keplerate-type polyoxometalate and chloroform-soluble porphyrin. *Dalton Trans.* **44**, 4102–4107 (2015).

134. J. Zhang, C. Ma, Y. Hao, W. Chen, A flexible triboelectric nanogenerator based on TiO_2 nanoarrays and polyoxometalate for harvesting biomechanical energy. *ACS Appl. Nano Mater.* **7**, 16922–16931 (2014).

135. Y. Xue, M. Wei, D. Fu, Y. Wu, B. Sun, X. Yu, L. Wu, A visual discrimination of existing states of virus capsid protein by a giant molybdate cluster. *Nanomaterials (Basel)* **12**, 736 (2022).

136. V. Fazylova, N. Shevtsev, S. Mikhailov, G. Kim, A. Ostroushko, K. Grzhegorzhevskii, Fundamental aspects of xanthene dye aggregation on the surfaces of nanocluster polyoxometalates: H- to J-aggregate switching. *Chem. A Eur. J.* **26**, 5685–5693 (2020).

137. H. Taghiyar, B. Yadollahi, A. A. Kajani, Controlled drug delivery and cell adhesion for bone tissue regeneration by Keplerate polyoxometalate (Mo_{132})/metronidazole/PMMA scaffolds. *Sci. Rep.* **12**, 14443 (2022).

138. M. Conte, X. Liu, D. M. Murphy, S. H. Taylor, K. Whiston, G. J. Hutchings, Insights into the reaction mechanism of cyclohexane oxidation catalysed by molybdenum blue nanorings. *Catal. Lett.* **146**, 126–135 (2016).

139. X. Liu, M. Conte, W. Weng, Q. He, R. L. Jenkins, M. Watanabe, D. J. Morgan, D. W. Knight, D. M. Murphy, K. Whiston, C. J. Kiely, G. J. Hutchings, Molybdenum blue nano-rings: An effective catalyst for the partial oxidation of cyclohexane. *Cat. Sci. Technol.* **5**, 217–227 (2015).

140. X. Chen, G. Zhang, B. Li, L. Wu, An integrated giant polyoxometalate complex for photothermally enhanced catalytic oxidation. *Sci. Adv.* **7**, eabf8413 (2021).

141. R. Haddad, Highly reactive heterogeneous nanofibers catalyst based on [Mo_{154}] clusters for green aerobic oxidation of sulfur mustard analogues under ambient conditions. *Cur. Org. Synth.* **19**, 808–818 (2022).

142. G. Guedes, S. Wang, F. Fontana, P. Figueiredo, J. Lindén, A. Correia, R. J. B. Pinto, S. Hietala, F. L. Sousa, H. A. Santos, Dual-crosslinked dynamic hydrogel incorporating {Mo_{154}} with pH and NIR responsiveness for chemo-photothermal therapy. *Adv. Mater.* **33**, e2007761 (2021).

143. G. Chen, Y. Wang, X. Kong, H. Li, B. Li, X. Yu, L. Wu, Y. Wu, Synergistic TME-manipulation effects of a molybdenum-based polyoxometalate enhance the PTT effects on cancer cells. *New J. Chem.* **46**, 6932–6939 (2022).

144. L. Zhang, T. Xiong, Y. Zhou, L. Zhang, En route to nanodevices of polyoxometalate: Incorporating the giant nanoporous molybdenum-oxide based wheels and balls into nanotubular arrays. *Chem. Asian J.* **5**, 1984–1987 (2010).

145. H.-Y. Wang, S.-R. Li, X. Wang, L.-S. Long, X.-J. Kong, L.-S. Zheng, Enhanced proton conductivity of Mo_{154}-based porous inorganic framework. *Sci. China Chem.* **64**, 959–963 (2021).

146. B. Jing, X. Wang, H. Wang, J. Qiu, Y. Shi, H. Gao, Y. Zhu, Shape and mechanical control of poly(ethylene oxide) based polymersome with polyoxometalates via hydrogen bond. *J. Phys. Chem. B* **121**, 1723–1730 (2017).

147. O. Koyun, S. Gorduk, M. B. Arvas, Y. Sahin, Direct, one-step synthesis of molybdenum blue using an electrochemical method, and characterization studies. *Synth. Met.* **233**, 111–118 (2017).

148. R. Sun, T. Wang, X. Ren, L. Zhang, Y. Zhou, C. Huang, Third-order optical nonlinearities of zinc porphyrins accommodated in the cavity of a doughnut-like molybdenum crown cluster. *Dalton Trans.* **51**, 7966–7974 (2022).

149. H. Ullah, T. S. Alomar, S. Hussain, F. K. Shehzad, K. S. Munawar, N. Al Masoud, M. Ammar, H. M. Asif, M. Sohail, M. Ajmal, Polyoxometalate based ionic liquids reinforced on magnetic nanoparticles: A sustainable solution for microplastics and heavy metal ions elimination from water. *Microchem. J.* **204**, 110941 (2024).

150. L. Wang, W. Wang, X. Xu, M. Abassian, Efficient and selective photoreduction of carbon dioxide to methane using nanopolyoxomolybdate supported by Ar-g-C_3N_4 on dendritic fibrous nanotitanium. *Catal. Lett.* **154**, 3402–3417 (2024).

151. M. Abasian, R. Zhiani, A. Motavalizadehkakhky, H. Eshghi, J. Mehrzad, Hydrogenation of CO_2 to formate using nanopolyoxomolybdate supported onto dendritic fibrous nanosilica. *Inorg. Chem. Commun.* **151**, 110580 (2023).

152. B. Xiao, S. M. Sadeghzadeh, Sustainable oxidation of olefins and sulfides employing nanopolyoxomolybdate supported by ionic liquid on dendritic fibrous nanosilica. *J. Mol. Liq.* **363**, 119921 (2022).

153. Z. Huang, S. Liu, Q. Geng, H. Zeng, Y. Li, S. Xu, S. M. Sadeghzadeh, Sustainable production of biodiesel using nanocluster giant lemon nanopolyoxomolybdate supported on carbon nanotubes by ionic liquid. *Inorg. Chem. Commun.* **142**, 109714 (2022).

154. Y. Wang, J. Li, X. Sang, W. Chen, Z. Su, E. Wang, A $TiO_2@\{Mo_{368}\}$ composite: Synthesis, characterization, and application in dye-sensitized solar cells. *J. Coord. Chem.* **67**, 3873–3883 (2014).

155. P. Tang, R. Liu, X. Li, X. Yuan, Y. Wang, J. Hao, Huge electron sponge of polyoxometalate toward advanced lithium-ion storage. *Langmuir* **40**, 13860–13869 (2024).

156. P. Tang, Y. Yuan, C. Sun, Honeycomb films of polyoxomolybdate-surfactant hybrids and electrochemical detection of hydroquinone. *J. Disper. Sci. Technol.* **44**, 2322–2329 (2023).

157. C. Bao, X. Liu, X. Shao, X. Ren, Y. Zhang, X. Sun, D. Fan, Q. Wei, H. Ju, Cardiac troponin I photoelectrochemical sensor: $\{Mo_{368}\}$ as electrode donor for Bi_2S_3 and Au co-sensitized FeOOH composite. *Biosens. Bioelectron.* **157**, 112157 (2020).

158. D. G. Kurth, P. Lehmann, D. Volkmer, H. Cölfen, M. J. Koop, A. Müller, A. Du Chesne, Surfactant-encapsulated clusters (SECs): $(DODA)_{20}(NH_4)[H_3Mo_{57}V_6(NO)_6O_{183}(H_2O)_{18}]$, a case study. *Chem. Eur. J.* **6**, 385–393 (2000).

159. F. Caruso, D. G. Kurth, D. Volkmer, M. J. Koop, A. Müller, Ultrathin molybdenum polyoxometalate−polyelectrolyte multilayer films. *Langmuir* **14**, 3462–3465 (1998).

160. A. Rezaeifard, A. Khoshyan, M. Jafarpour, M. Pourtahmas, Selective aerobic benzylic C–H oxidation co-catalyzed by *N*-hydroxyphthalimide and keplerate {$Mo_{72}V_{30}$} nanocluster. *RSC Adv.* **7**, 15754–15761 (2017).

161. F. Jalilian, B. Yadollahi, M. R. Farsani, S. Tangestaninejad, H. A. Rudbari, R. Habibi, Catalytic performance of keplerate polyoxomolybdates in green epoxidation of alkenes with hydrogen peroxide. *RSC Adv.* **5**, 70424–70428 (2015).

162. F. Jalilian, B. Yadollahi, M. R. Farsani, S. Tangestaninejad, H. A. Rudbari, R. Habibi, New perspective to Keplerate polyoxomolybdates: Green oxidation of sulfides with hydrogen peroxide in water. *Catal. Commun.* **66**, 107–110 (2015).

163. C.-C. Lin, C.-T. Hsu, W. Liu, S.-C. Huang, M.-H. Lin, U. Kortz, A. S. Mougharbel, T.-Y. Chen, C.-W. Hu, J.-F. Lee, C.-C. Wang, Y.-F. Liao, L.-J. Li, L. Li, S. Peng, U. Stimming, H.-Y. Chen, In operando x-ray studies of high-performance lithium-ion storage in keplerate-type polyoxometalate anodes. *ACS Appl. Mater. Interfaces* **12**, 40296–40309 (2020).

164. C. Huang, Y. Chai, Y. Jiang, J. Forth, P. D. Ashby, M. M. L. Arras, K. Hong, G. S. Smith, P. Yin, T. P. Russell, The interfacial assembly of polyoxometalate nanoparticle surfactants. *Nano Lett.* **18**, 2525–2529 (2018).

165. W.-J. Liu, G. Yu, M. Zhang, R.-H. Li, L.-Z. Dong, H.-S. Zhao, Y.-J. Chen, Z.-F. Xin, S.-L. Li, Y.-Q. Lan, Investigation of the enhanced lithium battery storage in a polyoxometalate model: From solid spheres to hollow balls. *Small Methods* **2**, 1800154 (2018).

166. M. Clemente-León, E. Coronado, C. J. Gómez-García, C. Mingotaud, S. Ravaine, G. Romualdo-Torres, P. Delhaès, Polyoxometalate monolayers in langmuir–blodgett films. *Chem. A Eur. J.* **11**, 3979–3987 (2005).

167. Z. Garazhian, A. Rezaeifard, M. Jafarpour, A. Farrokhi, {$Mo_{72}Fe_{30}$} nanoclusters for the visible-light-driven photocatalytic degradation of organic dyes. *ACS Appl. Nano Mater.* **3**, 648–657 (2020).

168. R. Mokhtari, A. Rezaeifard, M. Jafarpour, A. Farrokhi, Visible-light driven catalase-like activity of blackberry-shaped {$Mo_{72}Fe_{30}$} nanovesicles: Combined kinetic and mechanistic studies. *Cat. Sci. Technol.* **8**, 4645–4656 (2018).

169. A. Rezaeifard, R. Mokhtari, Z. Garazhian, M. Jafarpour, K. V. Grzhegorzhevskii, Tetrahedral Keggin core tunes the visible light-assisted catalase-like activity of icosahedral Keplerate shell. *Inorg. Chem.* **61**, 7878–7889 (2022).

170. M. Nikookar, A. Rezaeifard, K. V. Grzhegorzhevskii, M. Jafarpour, R. Khani, Melem nanorectangular prism-modified {$Mo_{72}Fe_{30}$} nanocapsule as a visible-light-assisted photocatalyst for catalase-like activity. *ACS Appl. Nano Mater.* **5**, 7917–7931 (2022).

171. H. Taghiyar, B. Yadollahi, New perspective to catalytic epoxidation of olefins by Keplerate containing Keggin polyoxometalates. *Polyhedron* **156**, 98–104 (2018).

172. R. Fareghi-Alamdari, S. M. Hafshejani, H. Taghiyar, B. Yadollahi, M. R. Farsani, Recyclable, green and efficient epoxidation of olefins in water with hydrogen peroxide catalyzed by polyoxometalate nanocapsule. *Catal. Commun.* **78**, 64–67 (2016).

173. R. Kaushik, R. Sakla, D. A. Jose, A. Ghosh, Giant iron polyoxometalate that works as a catalyst for water oxidation. *New J. Chem.* **44**, 3764–3770 (2020).

174. H. K. Kolli, D. Jana, S. K. Das, Nanoblackberries of {$W_{72}Fe_{33}$} and {$Mo_{72}Fe_{30}$}: Electrocatalytic water reduction. *Inorg. Chem.* **60**, 15569–15582 (2021).

175. H. Taghiyar, B. Yadollahi, Keggin polyoxometalates encapsulated in molybdenum-iron-type keplerate nanoball as efficient and cost-effective catalysts in the oxidative desulfurization of sulfides. *Sci. Total Environ.* **708**, 134860 (2020).

176. E. Nikbakht, B. Yadollahi, M. Riahi Farsani, Green oxidation of alcohols in water by a polyoxometalate nano capsule as catalyst. *Inorg. Chem. Commun.* **55**, 135–138 (2015).

177. K. Krishnamoorthy, P. Pazhamalai, R. Swaminathan, V. Mohan, S.-J. Kim, Unravelling the bi-functional electrocatalytic properties of {$Mo_{72}Fe_{30}$} polyoxometalate nanostructures for

overall water splitting using scanning electrochemical microscope and electrochemical gating methods. *Adv. Sci.* **11**, 2401073 (2024).

178. S.-C. Huang, C.-C. Lin, C.-T. Hsu, C.-H. Guo, T.-Y. Chen, Y.-F. Liao, H.-Y. Chen, Keplerate-type polyoxometalate $\{Mo_{72}Fe_{30}\}$ nanoparticle anodes for high-energy lithium-ion batteries. *J. Mater. Chem. A* **8**, 21623–21633 (2020).

179. H. Sun, Q. Yang, J. Hao, Self-patterning porous films of giant vesicles of $\{Mo_{72}Fe_{30}\}(DODMA)_3$ complexes as frameworks. *Adv. Colloid Interface Sci.* **235**, 14–22 (2016).

180. A. Ostroushko, I. Gagarin, M. Tonkushina, K. Grzhegorzhevskii, O. Russkikh, Association of spherical porous nanocluster keplerate-type polyoxometalate $Mo_{72}Fe_{30}$ with biologically active substances. *J. Clust. Sci.* **29**, 111–120 (2018).

181. M. O. Tonkushina, K. V. Grzhegorzhevskii, A. A. Ermoshin, A. S. Tugbaeva, G. A. Kim, O. S. Taniya, I. D. Gagarin, A. A. Ostroushko, The electrostatic-mediated formation of a coordination complex: The trapping and release of an antitumor drug with an anthracycline core from $\{Mo_{72}Fe_{30}\}$-based ensembles. *ChemistrySelect* **7**, e202203684 (2022).

182. K. Grzhegorzhevskii, M. Tonkushina, P. Gushchin, I. Gagarin, A. Ermoshin, K. Belova, A. Prokofyeva, A. Ostroushko, A. Novikov, Association of keplerate-type polyoxometalate $\{Mo_{72}Fe_{30}\}$ with tetracycline: Nature of binding sites and antimicrobial action. *Inorganics* **11**, 9 (2023).

183. A. Rezaeifard, M. Jafarpour, R. Haddad, F. Feizpour, $\{Mo_{72}Cr_{30}\}$ nanocluster as a novel self-separating catalyst for hydrogen peroxide olefin epoxidation. *Catal. Commun.* **95**, 88–91 (2017).

184. T. Wang, T. Ji, W. Chen, X. Li, W. Guan, Y. Geng, X. Wang, Y. Li, Z. Kang, Polyoxometalate film simultaneously converts multiple low-value all-weather environmental energy to electricity. *Nano Energy* **68**, 104349 (2020).

185. D. Zhou, B. Li, Q. Zhao, X. Tang, T. Lan, H. Su, G. Yang, W. Xuan, Solvent-modulated assembly of peptide and cerium functionalized gigantic $\{Mo_{120}Ce_6\}_2$ dimers for high-efficiency photocatalytic oxidation. *Inorg. Chem. Front.* **11**, 2355–2364 (2024).

186. H. Lu, Z. Li, Z. Duan, Y. Liao, K. Liu, Y. Zhang, L. Fan, T. Xu, D. Yang, S. Wang, Y. Fu, H. Xiang, Y. Chen, G. Li, Photothermal catalytic reduction and bone tissue engineering towards a three-in-one therapy strategy for osteosarcoma. *Adv. Mater.* **36**, e2408016 (2024).

187. G. Jin, S.-M. Wang, W.-L. Chen, C. Qin, Z.-M. Su, E.-B. Wang, A photovoltaic system composed of a Keplerate-type polyoxometalate and a water-soluble poly(p-phenylenevinylene) derivative. *J Mater Chem A* **1**, 6727–6730 (2013).

188. H. Haddadi, E. M. Korani, S. M. Hafshejani, M. R. Farsani, Highly selective oxidation of sulfides to sulfones by H_2O_2 catalyzed by porous capsules. *J. Clust. Sci.* **26**, 1913–1922 (2015).

189. M. Bugnola, R. Carmieli, R. Neumann, Aerobic electrochemical oxygenation of light hydrocarbons catalyzed by an iron–tungsten oxide molecular capsule. *ACS Catal.* **8**, 3232–3236 (2018).

190. M. Bugnola, K. Shen, E. Haviv, R. Neumann, Reductive electrochemical activation of molecular oxygen catalyzed by an iron-tungstate oxide capsule: Reactivity studies consistent with compound I type oxidants. *ACS Catal.* **10**, 4227–4237 (2020).

191. D. C. Crans, B. Baruah, N. E. Levinger, Oxovanadates: A novel probe for studying lipid-water interfaces. *Biomed. Pharmacother.* **60**, 174–181 (2006).

192. E. Heath, O. W. Howarth, Vanadium-51 and oxygen-17 nuclear magnetic resonance study of vanadate(V) equilibria and kinetics. *J. Chem. Soc. Dalton Trans.* **5**, 1105–1110 (1981).

193. L. Biancalana, G. Tuci, F. Piccinelli, F. Marchetti, M. Bortoluzzi, G. Pampaloni, Vanadium(V) oxoanions in basic water solution: A simple oxidative system for the one-pot selective conversion of L-proline to pyrroline-2-carboxylate. *Dalton Trans.* **46**, 15059–15069 (2017).

194. E. Sánchez-Lara, S. Treviño, B. L. Sánchez-Gaytán, E. Sánchez-Mora, M. Eugenia Castro, F. J. Meléndez-Bustamante, M. A. Méndez-Rojas, E. González-Vergara, Decavanadate salts of cytosine and metformin: A combined experimental-theoretical study of potential metallodrugs against diabetes and cancer. *Front. Chem.* **6**, 2296–2646 (2018).

195. C. Slebodnick, V. L. Pecoraro, Solvent effects on ^{51}V NMR chemical shifts: Characterization of vanadate and peroxovanadate complexes in mixed water/acetonitrile solvent. *Inorg. Chim. Acta* **283**, 37–43 (1998).

196. S. Himeno, K. Kawasaki, M. Hashimoto, Preparation and characterization of an α-Wells–Dawson-type $[V_2Mo_{18}O_{62}]^{6-}$ complex. *Bull. Chem. Soc. Jpn.* **81**, 1465–1471 (2008).

197. M. Nabavi, F. Taulelle, C. Sanchez, M. Verdaguer, Xanes and ^{51}V NMR study of vanadium-oxygen compounds. *J. Phys. Chem. Solid* **51**, 1375–1382 (1990).

198. M. A. Fedotov, R. I. Maksimovskaya, NMR structural aspects of the chemistry of V, Mo, W polyoxometalates. *J. Struct. Chem.* **47**, 952–978 (2006).

199. V. Bondareva, T. Andrushkevich, G. Aleshina, R. I. Maksimovskaya, L. M. Plyasova, L. S. Dovlitova, E. B. Burgina, The formation of an active component in V-Mo-Nb-O catalysts of ethane oxidation and ammoxidation. *React. Kinet. Catal. Lett.* **88**, 183–191 (2006).

200. L. Krivosudsky, A. Roller, A. Rompel, Regioselective synthesis and characterization of monovanadium-substituted β-octamolybdate $[VMo_7O_{26}]^{5-}$. *Acta Crystallogr.* **C75**, 872–876 (2019).

201. R. I. Maksimovskaya, N. N. Chumachenko, ^{51}V and ^{17}O NMR studies of the mixed metal polyanions in aqueous V—Mo solutions. *Polyhedron* **6**, 1813–1821 (1987).

202. O. W. Howarth, L. Pettersson, I. Andersson, Aqueous molybdovanadates at high Mo: V ratio. *J. Chem. Soc. Dalton Trans.* **7**, 1799–1812 (1991).

203. D. Hou, K. S. Hagen, C. L. Hill, Pentadecavanadate, $V_{15}O_{42}{}^{9-}$, a new highly condensed fully oxidized isopolyvanadate with kinetic stability in water. *J. Chem. Soc. Chem. Commun.* **4**, 426–428 (1993).

204. A. A. Zhdanov, O. V. Shuvaeva, A study of complex phosphovanadomolybdates $[PV_xMo_{12-x}O_{40}]^{-(3+x)}$ by reversed-phase HPLC and capillary zone electrophoresis. *J. Anal. Chem.* **70**, 757–764 (2015).

205. D. V. Evtuguin, C. P. Neto, J. Rocha, J. D. P. de Jesus, Oxidative delignification in the presence of molybdovanadophosphate heteropolyanions: Mechanism and kinetic studies. *Appl. Catal. Gen.* **167**, 123–139 (1998).

206. V. F. Odyakov, E. G. Zhizhina, New process for preparing aqueous solutions of Mo-V-phosphoric heteropoly acids. *Russ. J. Inorg. Chem.* **54**, 361–367 (2009).

207. P. Csermely, A. Martonosi, G. C. Levy, A. J. Ejchart, ^{51}V-n.m.r. analysis of the binding of vanadium(V)oligoanions to sarcoplasmic reticulum. *Biochem. J.* **230**, 807–815 (1985).

208. P. J. Domaille, 1- and 2-Dimensional tungsten-183 and vanadium-51 NMR characterization of isopolymetakates and heteropolymetalates. *J. Am. Chem. Soc.* **106**, 7677–7687 (1984).

209. E. Ishikawa, D. Kihara, Y. Togawa, C. Ookawa, Cyclooctene epoxidation by hydrogen peroxide in the presence of vanadium-substituted lindqvist-type polyoxotungstate $[VW_5O_{19}]^{3-}$. *Eur. J. Inorg. Chem.* **2019**, 402–409 (2019).

210. C. Wang, L. Weng, Y. Ren, C. Du, B. Yue, M. Gu, H. He, Mixed-addenda lindqvist-type polyoxoanion $[V_2W_4O_{19}]^{4-}$-supported copper complexes. *Z. Anorg. Allg. Chem.* **637**, 472–477 (2011).

211. R. I. Maksimovskaya, A. K. Il'yasova, D. U. Begalieva, D. F. Takezhanova, Identification of mixed vanadium-tungsten polyoxocomplexes in aqueous solutions by ^{17}O and ^{51}V NMR. *Russ. Chem. Bull.* **33**, 1977–1982 (1984).

212. C. M. Flynn Jr., M. T. Pope, S. O'Donnel, Tungstovanadate heteropoly complexes. V. The ion $H_2W_{11}VVO_{40}{}^{7-}$ and the oxidation and reduction of tungstovanadates. *Inorg. Chem.* **13**, 831–833 (1974).

213. I. Andersson, J. J. Hastings, O. W. Howarth, L. Pettersson, Aqueous tungstovanadate equilibria. *J. Chem. Soc. Dalton Trans.* **13**, 2705–2711 (1996).

214. S. E. O'Donnel, M. T. Pope, Applications of vanadium-51 and phosphorus-31 nuclear magnetic resonance spectroscopy to the study of iso- and hetero-polyvanadates. *J. Chem. Soc. Dalton Trans.* **21**, 2290–2297 (1976).

215. K. Y. S. Ng, E. Gulari, Spectroscopic and scattering investigation of isopoly-molybdate and tungstate solutions. *Polyhedron* **3**, 1001–1011 (1984).

216. J. Twu, P. K. Dutta, Raman spectroscopic studies of intercalated molybdate ions in layered metal hydroxides. *Chem. Mater.* **4**, 398–401 (1992).

217. W. P. Griffith, P. J. Lesniak, Raman studies on species in aqueous solutions. Part III. Vanadates, molybdates, and tungstates. *J. Chem. Soc. A* **0**, 1066–1071 (1969).

218. J. Noack, F. Rosowski, R. Schlögl, A. Trunschke, Speciation of molybdates under hydrothermal conditions. *Z. Anorg. Allg. Chem.* **640**, 2730–2736 (2014).

219. J. Aveston, E. W. Anacker, J. S. Johnson, Hydrolysis of molybdenum(VI). Ultracentrifugation, acidity measurements, and raman spectra of polymolybdates. *Inorg. Chem.* **3**, 735–746 (1964).

220. S. Himeno, H. Niiya, T. Ueda, Raman studies on the identification of isopolymolybdates in aqueous solution. *Bull. Chem. Soc. Jpn.* **70**, 631–637 (1997).

221. M. Dieterle, G. Mestl, Raman spectroscopy of molybdenum oxides Part II. Resonance Raman spectroscopic characterization of the molybdenum oxides Mo_4O_{11} and MoO_2. *Phys. Chem. Chem. Phys.* **4**, 822–826 (2002).

222. R. Umair Khan, I. Khan, B. Ali, R. Muhammad, A. Samad, A. Shah, K. Song, D. Wang, Structural, dielectric, optical, and electrochemical performance of $Li_4Mo_5O_{17}$ for ULTCC applications. *Mater. Res. Bull.* **160**, 112142 (2023).

223. M. Green, Y. Li, Z. Peng, X. Chen, Dielectric, magnetic, and microwave absorption properties of polyoxometalate-based materials. *J. Magn. Magn. Mater.* **497**, 165974 (2020).

224. C. Rocchiccioli-Deltcheff, R. Thouvenot, M. Fouassier, Vibrational investigations of polyoxometallates. 1. Valence force field of $Mo_6O_{19}^{2-}$ based on total isotopic substitution (oxygen-18, molybdenum-92, molybdenum-100). *Inorg. Chem.* **21**, 30–35 (1982).

225. S. Jung, Speciation of molybdenum- and vanadium-based polyoxometalate species in aqueous medium and gas-phase and its consequences for M1 structured MoV oxide synthesis, thesis, Technical University Berlin, Berlin, Germany. (2018).

226. K. V. Grzhegorzhevskii, P. S. Zelenovskiy, O. V. Koryakova, A. A. Ostroushko, Thermal destruction of giant polyoxometalate nanoclusters: A vibrational spectroscopy study. *Inorg. Chim. Acta* **489**, 287–300 (2019).

227. A. Müller, A. M. Todea, J. van Slageren, M. Dressel, H. Bögge, M. Schmidtmann, M. Luban, L. Engelhardt, M. Rusu, Triangular geometrical and magnetic motifs uniquely linked on a spherical capsule surface. *Angew. Chem. Int. Ed. Engl.* **44**, 3857–3861 (2005).

228. P. F. Avila, T. J. Ripplinger, D. J. Kemper, J. L. Domine, C. D. Jordan, Features of vibrational and electronic structures of decavanadate revealed by resonance raman spectroscopy and density functional theory. *J. Phys. Chem. Lett.* **10**, 6032–6037 (2019).

229. M. Aureliano, C. Andre Ohlin, M. O. Vieira, M. P. M. Marques, W. H. Casey, L. A. E. Batista de Carvalho, Characterization of decavanadate and decaniobate solutions by Raman spectroscopy. *Dalton Trans.* **45**, 7391–7399 (2016).

230. A. M. Amado, M. Aureliano, P. J. A. Riberio-Claro, J. J. C. Teixeira-Dias, Combined Raman and ^{51}V NMR spectroscopic study of vanadium (V) oligomerization in aqueous alkaline solutions. *J. Raman Spectrosc.* **24**, 699–703 (1993).

231. P. Häufe, Raman-spectrophotometric determination of the tungstate anion and its isopolyanions in aqueous systems. *Z. Anal. Chem.* **310**, 388–391 (1982).

232. A. F. Redkin, G. V. Bondarenko, Raman spectra of tungsten-bearing solutions. *J. Solution Chem.* **39**, 1549–1561 (2010).

233. M. Picquart, S. Castro-Garsia, J. Lilage, C. Julien, E. Haro-Poniatowski, Structural studies during gelation of WO_3 investigated by in-situ Raman spectroscopy. *J. Sol. Gel Sci. Technol.* **18**, 199–206 (2000).

234. L. Fan, J. Cao, C. Hu, What can electrospray mass spectrometry of paratungstates in an equilibrating mixture tell us? *RSC Adv.* **5**, 83377–83382 (2015).

235. D. Wesolowski, S. E. Drummond, R. E. Mesme, H. Ohmoto, Hydrolysis equilibria of tungsten (VI) in aqueous sodium chloride solutions to 300°C. *Inorg. Chem.* **23**, 1120–1132 (1984).

236. J. J. Cruywagen, I. F. J. van der Merwe, Tungsten(VI) equilibria: A potentiometric and calorimetric investigation. *J. Chem. Soc. Dalton Trans.* **7**, 1701–1705 (1987).

237. J.-C. Raabe, F. Jameel, M. Stein, J. Albert, M. J. Poller, Heteroelements in polyoxometalates: A study on the influence of different group 15 elements on polyoxometalate formation. *Dalton Trans.* **53**, 454–466 (2024).

238. E. Majzik, F. P. Franguelli, G. Lendvay, L. Trif, C. Németh, A. Farkas, S. Klébert, L. Bereczki, I. M. Szilágyi, L. Kótai, Deuteration and vibrational spectra of dimethylammonium paratungstate-B hydrates. *Z. Anorg. Allg. Chem.* **647**, 593–598 (2021).

239. D. L. Collins-Wildman, M. Kim, K. P. Sullivan, A. M. Plonka, A. I. Frenkel, D. G. Musaev, C. L. Hill, Buffer-induced acceleration and inhibition in polyoxometalate-catalyzed organophosphorusester hydrolysis. *ACS Catal.* **8**, 7068–7076 (2018).
